# An alternative for proteinase K-heat-sensitive protease from fungus *Onygena corvina* for biotechnology: cloning, engineering, expression, characterization and special application for protein sequencing

**DOI:** 10.1186/s12934-020-01392-3

**Published:** 2020-06-24

**Authors:** Piotr M. Skowron, Daria Krefft, Robert Brodzik, Paulina Kasperkiewicz, Marcin Drag, Klaus-Peter Koller

**Affiliations:** 1 Department of Molecular Biotechnology, Faculty of Chemistry, University of Gdansk, Wita Stwosza 63 Street, 80-308 Gdansk, Poland; 2 BLIRT S.A., Trzy Lipy 3/1.38 Street, 80-172 Gdansk, Poland; 3 Department of Chemical Biology and Bioimaging, Wroclaw University of Science and Technology, Wyb. Wyspianskiego 27 Street, 50-370 Wroclaw, Poland; 4 Institute for Molecular Bio Science, University Frankfurt, Max-von-Laue-Str. 9, 60438 Frankfurt, Germany

**Keywords:** Serine protease, *Onygena corvina*, *Pichia pastoris*, Expression, Protease, Protein degradation, Protein sequencing, Proteinase K, Food processing, Waste processing

## Abstract

**Background:**

A neutral, heat-sensitive serine protease (NHSSP) originating from the feather-degrading fungus *Onygena corvina* (*O. corvina*) was described and defined as an alkaline serine protease of the subtilisin type S8 family, exhibiting an enzymatic activity at neutral pH. Generally, broad specificity proteases, such as proteinase K or trypsin, have found numerous applications in research and biotechnology.

**Results:**

We report the cloning and expression in the yeast PichiaPink™ system, as well as purification, and characterization of the NHSSP. Recombinant, His_6_-tagged NHSSP was efficiently expressed from an optimized, synthetic gene and purified using a simple protocol based on ammonium sulfate fractionation and hydrophobic interaction chromatography. The enzyme shows atypical C-terminal processing, the coded preproprotein undergoes signal peptide removal and maturation through the clipping of a propeptide section and 10 amino acids (aa) from the C-terminus, including the His_6_-tag. The deletion variant has been constructed, devoid of the C-terminal ORF segment, thus eliminating the need for C-terminal processing. Both NHSSP variants exhibit very similar enzymatic characteristics. The purified enzymes were characterized to determine the optimal proteolytic conditions. We revealed that the mature NHSSP is reproducibly active over a wide pH range from neutral to mild acidic (pH of 5.0 to 8.5), with an optimum at pH 6.8, and at temperatures of 15 to 50 °C with an optimum at 38–42 °C. Interestingly, we demonstrated that the protease can be fully deactivated by a moderate increase in temperature of about 15 °C from the optimum to over 50 °C. The protease was partially sensitive to serine protease inhibitors, and not inhibited by chelating or reducing agents and detergents. SDS induced autolysis of NHSSP, which points to a high stimulation of its proteolytic activity.

**Conclusions:**

The NHSSP was produced as a recombinant protein with high efficiency. Compared to proteinase K, the most common serine protease used, NHSSP shows an approx. twofold higher specific activity. Protein sequencing can be a valuable technical application for the protease. The protein coverage is significantly higher in comparison to trypsin and reaches about 84–100% for β-lactoglobulin (BLG), antibody (mAb) light and heavy chains. Furthermore, the option to perform digestions at neutral to slightly acidic pH-values down to pH 5.0 avoids modification of peptides, e.g. due to deamidation.

## Background

Proteases comprise one of the largest family of hydrolytic enzymes. They catalyse the hydrolysis of peptide bonds and thus proteases are involved in protein turnover, as well as numerous physiological pathways throughout all lifeforms [1, 2]. Since proteases cleave critically important cellular components, they are inherently very toxic to the cell if not properly controlled. Thus, for example, serine proteases are synthesized as inactive “zymogenic precursors” or “zymogens” in the form of a pre-proenzyme, which are activated by the removal of a signal sequence (secretion signal peptide or pre-peptide) and pro-sequence (pro-peptide) to yield an active mature enzyme [2, 3]. This activation process may result from the autocatalytic processing of the serine protease zymogen or through the presence of other proteolytic activities. Numerous alkaline serine proteases (EC 3.4.21) and genes encoding such enzymes have been isolated from eukaryotic organisms, including yeast and fungi. Typically, mature serine proteases have a molecular mass around 25 to 30 kDa [4] and are generally active at neutral or alkaline pH, with an optimum between pH 7.0 and 11.0, and have broad substrate specificity. Moreover, this group includes enzymes that are active and stable at pH 9.0 to pH 11.0 or even at pH 10.0 to 12.5 [5] with an isoelectric point of about pH 9.0. These represent the largest subgroup of commercially used serine proteases. The molecular masses of alkaline serine proteases range between 15 and 35 kDa. The temperature optima of the natural serine proteases are around 15–60 °C, depending in most part on the ecological niche of the producing organism [4]. These enzymes are also very important for biochemistry research, medicine, and manufacturing applications [1, 2]. In particular, proteases active at cold or moderate temperatures are finding increasing applications in food processing, cleaning, industrial conversion processes, protein bio-waste removal and medicinal uses, such as drug production or wound healing [4, 6]. A substantial effort has been undertaken to identify enzymes from natural sources or to generate variants of naturally occurring enzymes by mutagenesis to exhibit features that may better meet the needs of specific industrial applications [7]. Due to ecological and economical needs, which may also be manifested by governmental regulations, there is always the need to reduce waste production and save energy. However, there is still a need for the provision of alternative proteases that effectively degrade proteinaceous material whereby the protease is active at moderate temperatures and at a neutral or slightly acidic pH value. The fungus *O. corvina* produces a vast variety of proteases when grown on a very problematic to digest protein substrate—feathers [8]. One of these proteases (GenBank Accession No. KP290860.1, UniProtKB Accession No. A0A0B4VM82) [9, 10] was expressed as a His_6_-tagged protein after cDNA cloning and described as an alkaline S-8 protease based on bioinformatics analysis. However, detailed enzyme characteristics were missing. We engineered and cloned the synthetic gene, expressed it as a His_6_-tagged protein in the PichiaPink™ system and purified the mature enzyme. Surprisingly, a few aspects turned out to be new and challenged the bioinformatics analysis: the enzyme turns out to be a neutral heat-sensitive protease of the S-1 type. It follows a very unusual post-translational N- and C-terminal processing to produce the mature form. The synthetic truncated gene version expression confirmed the processing. Both the full length and truncated enzyme are highly suitable for studies on peptide mapping using MS/MS analysis and for protein sequencing. There, in overnight digestions, the enzyme exhibits only a slight preference for phenylalanine (F) and bulky neutral amino acids (aa), but instead, cleaves after almost all aa residues except proline (P). It leaves, however, clear and reproducible fragment patterns. The very high sequence coverage makes it attractive for protein sequencing using nanoLC-ESI–MS/MS—a routine method for analysis. In addition, the use of a neutral to slightly acidic pH range for digestions offers the advantage to avoid protein modification via deamidation. These frequently occur as trypsin or other enzymes in the digestion assays require a basic environment for optimal results.

## Materials and methods

### Reagents, vectors, bacterial and yeast strains

All reagents and chemicals were obtained from Sigma-Aldrich (St. Louis, MO, USA), if not otherwise indicated. Synthetic DNA, vector pPink-HC, *Escherichia coli* (*E. coli*) competent cells TOP10, *Pichia pastoris* (*P. pastoris*)-based PichiaPink™ Expression System no MAN0000717 and PageRuler™ Prestained Protein Ladder (SM0671) were from Thermo Fisher Scientific Inc. (Wilmington, DE, USA). ExtractMe™ Purification Kits (EM01.1) and rabbit anti-His_6_ antibodies were from DNA Gdansk/BLIRT (Gdansk, Poland). DNA purification kits ‘Clean-Up’ and ‘Gel-Out’ were from A&A Biotechnology (Gdynia, Poland). Chemical synthesis of the optimized full-length NHSSP-His_6_ gene was performed by GeneArt™ gene synthesis (Thermo Fisher Scientific Inc., Waltham, MA, USA). Q5 Polymerase and NEB reaction buffer were from New England Biolabs (Ipswich, MA, USA). Restriction endonucleases, T4 DNA ligase, calf intestinal phosphatase were from Thermo Fisher Scientific Inc./Fermentas (Vilnus, Lithuania). Phenyl-Sepharose 6FastFlow (low sub) resin and Amersham™ LMW Calibration Kit For SDS Electrophoresis: 97–30 kDa were from GE Healthcare (Chicago, IL, USA). Vivaflow 10 kDa MWCO PES filtered cassette was from Sartorius (Göttingen, Germany). Sequencing grade trypsin was from SERVA Electrophoresis GmbH (Heidelberg, Germany). Endoproteinase Asp-N (11058541103) was from Roche Diagnostics (Warsaw, Poland). ProSorb PVDF cartridge was from Applied Biosystems (Foster City, CA, USA). Proteinase K was from Merck-Millipore (Darmstadt, Germany). Goat anti-rabbit-HRP ab6721 antibodies were from Abcam (Cambridge, UK). Chemiluminescent substrate for peroxidase was from Pierce (Rockford, IL, USA).

### Construction of the expression plasmids

#### Full length NHSSP-His_6_

Based on the aa sequence disclosed by Huang et al. [8] for protease 6877 of *O. corvina* (EMBL protein database, Accession No KP290860) [11], a protein with the native protease 6877 signal peptide (SP) and a C-terminal His_6_-tag was designed. The expression plasmid was constructed by cloning a 1219-bp fragment (Additional files 1, 2, 3) comprising the synthetic gene encoding full-length NHSSP-His_6_ from *O. corvina* into the multiple cloning site of the vector pPink-HC into sites EcoRI (5′-end) and KpnI (3′-end). Following digestion of the vector with EcoRI and KpnI, the vector was dephosporylated with calf intestinal phosphatase and the linearized vector was resolved on an agarose gel/TBE buffer electrophoresis and purified from the gel. The synthetic DNA was cut with EcoRI/KpnI, then purified and ligated with T4 DNA ligase. *E. coli* TOP10 competent cells were electroporated with the ligation mixture, incubated in SOC medium for 1 h in 37 °C and plated on LA medium containing 100 µg/ml ampicillin, then followed incubation for 16 h at 30 °C and 37 °C. The generated plasmids pPink-HC-NHSSP (Additional files 4, 5, 6) were propagated in TOP10 *E. coli* cells and purified using a ExtractMe™ MidiPrep Purification Kit. The integrity of the expression cassette encoding preproNHSSP-His_6_ in pPink-HC-NHSSP was confirmed by DNA sequencing using a forward primer 5′-GACTGGTTCCAATTGACAAGC-3′ specific to the vector’s AOX1 promoter and reverse primer 5′-GCGTGAATGTAAGCGTGAC specific to the CYC1 terminator. The clones with the correct gene sequence were selected and plasmid DNAs were propagated, purified and further used for *P. pastoris* transformation according to the PichiaPink™ Expression System no MAN0000717 User Manual (UM). The expression construct PichiaPink™ Strain 1 clone 101, designated as *Pichia pastoris* NHSSP was deposited at the Deutsche Sammlung von Mikroorganismen und Zellkulturen (DSMZ) under accession number DSM 32492.

#### Deletion protein variant NHSSP

Using the pPink-HC-NHSSP construct as a template, a PCR reaction was performed to obtain the C-terminally truncated version of the NHSSP-His_6_ gene, devoid of 4 C-terminal aa and the His_6_-tag (Additional files 7, 8, 9). The reaction was conducted in 50 µl volume and contained 30 ng of template DNA, 0.5 mM forward primer 5′-GCCAGAATTCATGGGTTGTATCAAGGTTATC-3′, 0.5 mM reverce primer 5′-GCCGGGTACCTCATTAGGAACCGTTATACAACAATCTGTTTG-3′, 0.2 mM of each dNTPs, the NEB reaction buffer and Q5 Polymerase. The cycling profile included: 98 °C for 30 s, 30 cycles of 98 °C for 10 s, 65 °C for 25 s and 72 °C for 25 s, and a final step of 72 °C for 2 min. The resulting PCR product was purified using a Clean-Up kit, digested with EcoRI/KpnI and ligated to EcoRI/KpnI-linearized and purified pPink-HC vector. Generated plasmids pPink-HC-del-NHSSP (Additional files 10, 11, 12) were propagated in *E. coli* TOP10 cells and purified using a ExtractMe™ MidiPrep Purification Kit. The integrity of the expression cassette encoding C-terminally truncated NHSSP (del-NHSSP) in pPink-HC-del-NHSSP was confirmed by DNA sequencing using a forward primer 5′-GACTGGTTCCAATTGACAAGC-3′ specific to the vector’s AOX1 promoter and reverse primer 5′-GCGTGAATGTAAGCGTGAC specific to CYC1 terminator. The clones with the correct gene sequence were selected and plasmid DNAs were propagated, purified and further used for *P. pastoris* transformation.

### Pichia transformation and clonal selection

pPink-HC-NHSSP or pPink-HC-del-NHSSP was linearized with AflII restriction endonuclease prior to *Pichia* transformation. The PichiaPink™ Strain 1 (ade2) was used for transformation. Competent PichiaPink™ cells were prepared according to the UM. 80 µl of competent cells were pre-incubated with 10 µg of plasmid DNA on ice for 5 min. Electroporation was performed with a Gene Pulser Xcell™ Electroporation System (BioRad, Hercules, CA) in 2 mm gap cuvettes using the Yeast Predefined Program (V = 2000 V, C = 25 µF, R = 200 Ω, t = 5 ms). Immediately after the pulse, 1 ml of ice-cold YPDS medium was added to the cells and incubated at 30 °C for 4 h. 100 µl or 200 µl of cells were plated on MGY plates and grown for 3–4 days at 30 °C. White colonies were selected for the screening of transgene expression.

### Expression analysis in micro-scale and upscaling

**Fig. 1:**
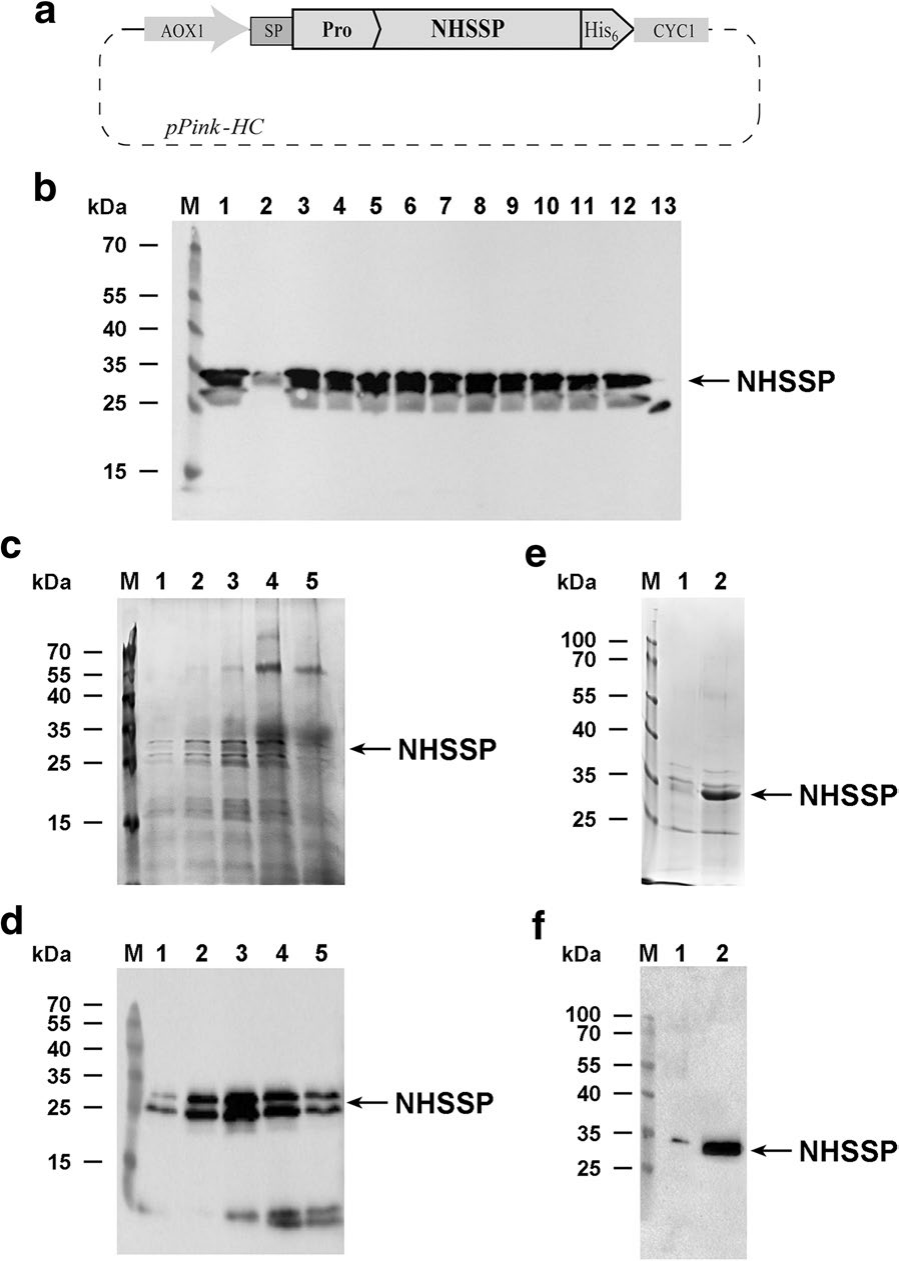
Production of NHSSP in PichiaPink™ system. **a** preproNHSSP-His_6_-coding synthetic gene was assembled in yeast expression vector pPink-HC, forming plasmid pPink-HC-NHSSP. The final expression cassette contains: the alcohol oxidase promoter AOX1, driving the coding sequence of NHSSP; the secretion signal peptide (SP), pro-peptide (Pro), the histidine tag (His_6_), the cytochrome C terminator from *Saccharomyces cerevisiae* (CYC1). **b** Western blot analysis of NHSSP expression in PichiaPink™ Strain 1, transformed with pPink-HC-NHSSP vector. Selected clones were grown in 24-well plates in 1 ml of BMMY medium. The synthesis of NHSSP protein was induced with methanol for 48 h. Then 15 µl of culture medium was mixed with 5 µl of Laemmli buffer, denatured and proteins were resolved on 12% SDS-PAGE, transferred to nitrocellulose membrane and probed with anti-His_6_ antibodies. Lanes 1, PageRuler™ Prestained Protein Ladder; lanes 2–13, expression culture supernatants from selected PichiaPink™ clones transformed with pPink-HC-NHSSP; lane 14, positive control for His_6_-tagged protein (25 kDa). **c** Scaled-up (fermentor) expression of selected pPink-HC-NHSSP clone. Cells were grown in 6 L batch of BSM medium on glycerol as a carbon source for 24 h. Then the culture was fed with methanol in cascade mode depending on oxygen level (maintained at minimum 30%) for another 100 h. Expression level of NHSSP in the culture medium was analysed on SDS-PAGE and **d** on Western blot. Lanes: lane M, PageRuler™ Prestained Protein Ladder; lanes 1–5, expression of NHSSP after 10, 32, 40, 80 and 100 h. **e** Fermenter-produced NHSSP was purified using ammonium sulfate fractionation and Phenyl-Sepharose chromatography. Fractions containing NHSSP were pooled and analyzed on SDS-PAGE and **f** on Western blot. Lanes M, PageRuler™ Prestained Protein Ladder; lanes 1, purified NHSSP

Recombinant cells carrying either pPink-HC-NHSSP or pPink-HC-del-NHSSP were grown in 24-well plates. 1.5 ml of BMGY medium was inoculated with a single white clone per well and grown at 30 °C in an orbital shaker at 250 rpm mixing. Every 24 h, cells were centrifuged at 1500×*g* for 5 min and resuspended in 1 ml of BMMY medium containing 0.5% methanol, according to the UM. 48 h cultured media were analysed for the presence of the recombinant protein on SDS-PAGE and Western blots using rabbit anti-His_6_ antibodies. Further, to fine tune the expression conditions, the pilot mid-scale expression was conducted in 1 L of Basal Salt Medium (BSM) according to the UM. The pH level was kept at 4.8 at the beginning of culturing, then at pH 5.5 after induction was adjusted and controlled with 10% ammonia. The initial temperature of fermentation of 30 °C was reduced to 28 °C after induction. Methanol supplementation to induce expression was carried out for 48-80 h. The expression level was analysed over time during fermentation (Fig. 1). Large scale expression was conducted in recombinant Strain 1 (ade2), carrying either the pPink-HC-NHSSP or pPink-HC-del-NHSSP in a Sartorius Biostat C bioreactor in 6-8 L batches in BSM using fermentation conditions as outlined in the UM and 0.5% (v/v) methanol as AOX1 promoter inductor.

### SDS-PAGE and Western blotting

Samples from *P. pastoris* expression were collected and analysed for recombinant protein content. 15 µl of culture medium were mixed simultaneously with 5 µl of 5× Laemmli buffer and 1 µl of 100 mM PMSF and denatured at 95 °C for 5 min. Immediately, proteins were loaded onto 12% SDS-PAGE and subjected to electrophoresis [12], followed by either staining using Coomassie Brilliant Blue R-250 or transfer to an Amersham Protran nitrocellulose membrane (GE Healfcare, Cat. No. 10600003) in BSN buffer (48 mM Tris, 39 mM glycine, pH 9.2, 20% methanol) at 25 V for 20 min, using a Trans-Blot^®^ SD Semi-Dry Transfer Cell System (BioRad, Hercules, CA). The membrane was blocked with 5% non-fat milk in PBS buffer and incubated with rabbit anti-His_6_ mAb for 1 h at room temperature at 1: 5000 dilution in PBS-T buffer, washed 3 times with PBS-T and incubated for 1 h at room temperature with goat anti-rabbit antibodies conjugated to horseradish peroxidase at 1:10,000 dilution in PBS-T. After washing, reactive protein bands were visualized with a chemiluminescent substrate for peroxidase. Detection with anti-His_6_ antibodies was possible due to the presence of trace amounts of C-terminally unprocessed His_6_-tagged NHSSP and/or cross-reaction with internal NHSSP aa segment NANNDGHGHGTH. 

### Protein purification

#### Analytical ammonium sulfate fractionation

BSM medium with secreted NHSSP from expressed pPink-HC-NHSSP clone was filtered through a 0.22 µm sterile filter and subjected to stepwise ammonium sulfate precipitation. Solid ammonium sulfate was added to a final concentration of 40% saturation, stirred at 4 °C for 1 h, and the resulting precipitate was removed by centrifugation at 18,000×*g* for 30 min and discarded. To the remaining supernatant, solid ammonium sulfate was further added to a final concentration of 80% and, after 1 h stirring, the precipitate was recovered by centrifugation at 18,000×*g* for 30 min. The protein pellet containing active NHSSP was resuspended in Resuspension buffer (50 mM buffer NaH_2_PO_4_/Na_2_HPO_4_, 50 mM NaCl, pH 6.6) and dialyzed overnight against resuspension buffer in a proportion of 1:250 (v/v).

#### Preparative purification using ammonium sulfate fractionation and hydrophobic chromatography

5 L of post expression BSM medium was 0.22 µm filtered and concentrated to 500 ml using a Vivaflow 10 kDa MWCO PES filtered cassette and 30× times buffer was exchanged to the final buffer A (25 mM Na_2_HPO_4_/NaH_2_PO_4_, pH 6.6, 50 mM NaCl). At the end of buffer exchange, solid (NH_4_)_2_SO_4_ was added to a final concentration of 2 M and the solution was filter-sterilized through a pore size of 0.22 µm. NHSSP solution was loaded onto 100 ml Phenyl-Sepharose 6 FastFlow (low sub) resin packed in a XK50 column. The loading flow rate was 10 ml/min. Unbound protein was washed from the column with 1 L (10 column volumes) of buffer A, supplemented with 2 M (NH_4_)_2_SO_4_. NHSSP was eluted with 400 ml (4 column volumes) of buffer B (25 mM NaH_2_PO_4_/Na_2_HPO_4_ pH 6.6, 50 mM NaCl) using the elution profile: 30%, 50%, 70%, 85%, and 100% buffer B. Fractions of 20 ml were collected and analyzed on SDS-PAGE and Western blot for content of NHSSP protein. Fractions containing NHSSP protein were pooled and combined. Purified NHSSP was stored at a concentration of 0.4 to 1 mg/ml in NaH_2_PO_4_/Na_2_HPO_4_, 100 mM NaCl, pH 6.6, glycerol 10% (for storage at − 80 °C) or glycerol 50% (for storage at − 20 °C) before further use. The same scheme was used for del-NHSSP purification, with minor modifications: (buffer A: 25 mM Tris–HCl pH 6.0, 50 mM NaCl, 1.6 M (NH_4_)_2_SO_4_; buffer B: 10 mM Tris–HCl pH 7.5, 50 mM NaCl, 5 mM CaCl_2_).

### Protein concentration determination

The protein concentration in solution was determined spectrophotometrically at 280 nm using factor A1%10 mm = 11.0 as predicted by DNASTAR Protean Package v.11 based on the aa composition of NHSSP. Photometric assays were performed using a Spectrophotometer NanoDrop™ ND-1000 (Thermo Fisher Scientific Inc.).

### Proteolytic activity assay

Methods for analysing the proteolytic activity of serine proteases are well-known, e.g. [13]. The protease activity of the enzyme was determined as previously described by Huang et al. [8] using azocasein as a substrate. The reaction mixture contained 100 μl diluted enzyme solution and 100 μl 0.5% (w/v) azocasein dissolved in 50 mM KH_2_PO_4_/K_2_HPO_4_ buffer (pH 6.5). The reaction was carried out at 42 °C for 50 min with constant agitation at 300 rpm using a TS-100 Thermo-Shaker, SC-20 (Biosan Ltd). After incubation, the reaction was stopped by adding 300 μl of 0.6 M trichloroacetic acid (TCA) and centrifuged at 10,000×*g* for 10 min to remove the substrate. 100 μl supernatant was transferred to a microtiter plate and the absorbance was measured at 450 nm using a plate reader. As a control, 100 μl 0.5% w/v azocasein dissolved in the same buffer as the sample was added to 300 μl of 0.6 M TCA before the addition of 100 μl of enzyme solution. Then, the control mixture was incubated at 42 °C for 30 min in the same way as the sample. For pH optimum determinations, the enzymatic hydrolysis of azocasein was measured in Britton-Robinson buffer [14] at 30 °C for 30 min. The reaction was stopped by the addition of TCA to a final concentration of 293 mM, the mixture was centrifuged as described above and the absorbance of the supernatant was measured. The temperature-dependent proteolytic activity (optimum and heat inactivation) of NHSSP was assayed as above, at a constant pH 6.5 in 50 mM KH_2_PO_4_/K_2_HPO_4_ buffer and temperatures ranging from 10 to 70 °C. Determination of protease inhibitor effects were conducted using azocasein, in Britton-Robinson buffer at 40 °C for 30 min. Determination of specific activity and comparison to proteinase K was conducted as above, except that the temperature used was 42 °C. The specific activity was calculated according to the manufacturer’s information (Merck-Millipore, Darmstadt, Germany) using the formula: Protease (Units/mg) = (140 × Abs. (440 nm) − 4) × 1 mg/concentration of enzyme in the assay/1000. Linear absorbance range was 0.1 to 1.0, with r = 0.99. The effect of divalent metal cations Mn^2+^, Co^2+^, Ni^2+^, Mg^2+^, Ca^2+^, Fe^2+^, Zn^2+^ used at the concentration of 5 mM, was conducted in buffers with increased pH, to avoid hydrolysis: HEPES pH 8.0 and/or Glycine–NaOH, pH 9.8. For comparative assays of NHSSP versus del-NHSSP, a different substrate was used—AZCL-Casein and readings were conducted at 590 nm.

### N-, C-terminal sequencing and peptide mass fingerprinting

To determine precisely the NHSSP N- and C-termini and the molecular weight upon secretion and maturation, the purified protein was subjected to N- and C-terminal sequencing and peptide mass fingerprinting. Aliquots containing 4 μg of the NHSSP, expressed from the pPink-HC-NHSSP clone, containing His_6_-tagged ORF coding for full lengths NHSSP, were reduced with dithiothreitol, alkylated with iodoacetamide and digested with either sequencing grade trypsin or endoproteinase Asp-N at 37 °C overnight. The peptides were analyzed by MALDI-TOF–MS. As matrix, alpha-cyano-4-hydroxycinnamic acid was used. The measured peptide masses were compared with the provided sequences using the software Mascot (Matrix Science, Boston, MA, USA). The identification threshold was set to p < 0.05; peptide mass tolerance was set to 100 ppm. Carbamidomethylated C were set as fixed modifications; oxidized M were set as variable modifications. Additionally, Mascot database searches using the SwissProt database with sequences of all species were performed. For N-terminal sequencing, 6 μg NHSSP sample was diluted in 100 μl 0.1% TCA and immediately adsorbed on a ProSorb PVDF cartridge, washed with 100 μl HPLC-grade water, air dried and transferred to the sequencer cartridge. Sequencing was performed on a Procise 494 Sequencer (Applied Biosystems, Foster City, CA, USA). The standard method “Pulsed-Liquid” was used. The sample was sequenced for 8 cycles of Edman degradation [15]. At the beginning of the sequencing run, a blank gradient and a standard mixture containing 10 pmol of the PTH-derivatives of each of the 19 proteinogenic aa (without C) was analyzed. The aa from each sequencing cycle were identified by their retention time and quantitated by comparison of peak heights with the standard chromatogram.

### Application of the NHSSP for protein sequencing

#### Digests with NHSSP

1 nmol of BLG (Applied Biosystems 400 979) and Rituximab (Mabthera Roche) was dissolved in 50 µl 8 M urea/0.4 M ammonium bicarbonate, pH 8.0 and reduced by the addition of 5 µl 45 mM dithiothreitol at 55 °C for 1 h. After cooling to room temperature, alkylation of cysteines was performed with 5 µl 100 mM iodoacetamide for 15 min in the dark. The reduced and alkylated samples were diluted with 1 × PBS to a urea concentration of 2 M. The digest with del-NHSSP (truncated form) in the ratio 1:50 was performed at 30 °C overnight.

For the pH studies 10 µl samples of BSA (10 µg) in water were distributed to nine vials and diluted with 10 µl 8 M urea/0.4 M ammonium bicarbonate, pH 8.0 and reduced by the addition of 2 µl 45 mM dithiothreitol at 55 °C for 30 min. After cooling to room temperature, alkylation of C was performed with 2 µl 100 mM iodoacetamide for 15 min in the dark. Three replicates of the reduced and alkylated samples were diluted with 50 µl of 1× PBS pH 6.5, three replicates were diluted with 50 µl of 1× PBS pH 6.0 and three replicates were diluted with 50 µl of 1 × PBS pH 5.0. The digest with NHSSP (full length form) in the ratio 1:50 was performed at 30 °C overnight.

#### Digests with trypsin

The digests with trypsin were essentially done as described above, however, each protein was dissolved in 50 µl 8 M urea/0.4 M ammonium bicarbonate, pH 8.5 resembling also the final pH in the digestion step.

#### Mass spectrometry with nanoLC-ESI–MS/MS

For the measurement with nanoLC-ESI–MS/MS about 0.25 µg of the trypsin digests and 0.5 µg NHSSP digests were applied. For measurement 500 nl (~ 0.5 µg) of each NHSSP digest were subjected to HPLC separation using an EASY-nLC1000 (Thermo Scientific) system with the following columns and chromatographic settings: the peptides were applied to a C18 column (Acclaim^®^ PepMap 100 pre-column, C18, 3 µm, 2 cm × 75 µm Nanoviper, Thermo Scientific) and subsequently separated using an analytical column (EASY-Spray column, 50 cm × 75 µm ID, PepMap C18 2 µm particles, 100 Å pore size, Thermo Scientific) by using a linear gradient (A: 0.1% formic acid in water, B: 0.1% formic acid in 100% ACN) at a flow rate of 200 nl/min. The gradient used was: 2-50% B in 30 min, 84% B 10 min.

Mass-spectrometric analysis was done on a LTQ Orbitrap XL mass-spectrometer (Thermo Scientific) which was coupled online to the HPLC-system. The mass spectrometer was operated in the so-called “data-dependent” mode where after each global scan the five most intense peptide signals were chosen automatically for MS/MS-analysis.

#### Mascot database searches

The LC–ESI–MS/MS data were used for database searches with the software Mascot (Matrix Science) using customer specific databases that contained the sequence of BSA, BLA and mAb respectively. Peptide mass tolerance was set to 50 ppm; fragment mass tolerance was set to 0.6 Da. Protein significance threshold was set to P < 0.05 and an ion score cut off of > 20 was applied for peptide identification. Masses with carbamidomethyl at C were set as fixed modification and oxidation of methionine (M) and pyroglutamate formation at N-terminal glutamine (Q) were set as variable modifications.

## Results

### Cloning and expression of full lengths NHSSP-His_6_

Huang et al. [8] revealed the sequences for a vast number of putative and/or verified proteases found in the genome of the feather-digesting fungus *O. corvina,* including the sequence for protease 6877 of *O. corvina* (EMBL protein database, Accession No. KP290860) [11]. A *P. pastoris*-optimised gene was designed de novo with the addition of a C-terminal His_6_-tag. The full length preproNHSSP-His_6_ has a calculated molecular weight of 39.9 kDa. In silico bioinformatics simulations and database comparisons (UniProt Knowledgebase, Accession No. A0A0B4VM82) [10] led to the determination of the 19 aa long native SP of protease 6877, potentially driving secretion of proprotein NHSSP outside *O. corvina* cells. This native SP has been used in recombinant constructs, as several other PichiaPink™ SPs tested in this work did not lead to the efficient secretion of NHSSP (not shown). The bioinformatics analysis revealed that aa 20–144 comprise a propeptide sequence and aa 145–401 the final mature, His_6_-tagged NHSSP (Additional files 1, 2, 3, 4, 5, 6). The pI for the mature NHSSP was calculated to be 8.7 [16]. The chemically synthesized DNA was cloned into the pPink-HC vector, forming a perfect fusion with the vector’s start codon. Expression construct pPink-HC-NHSSP (Fig. 1a, Additional files 4, 5, 6) drove expression by the methanol-inducible promoter of the alcohol oxidase AOX1 when transformed into the PichiaPink™ Strain 1 (ade2). Other tested strains included: Strain 2 (ade2, pep4), Strain 3 (ade2, prb1), and Strain 4 (ade2, prb1, pep4), which all gave lower expression levels (not shown). A series of expression experiments, from titer plate, through mid-scale to large fermenter scale, were conducted to identify the optimal conditions for a simple expression-purification protocol. The cultivation conditions in these formats are inherently different than in larger scales, nevertheless, these tests allowed the selection of the most efficient clone and narrowed down the culturing variables. During large-scale cultivation using 5 L fermenters, the peak expression was obtained 80 h after induction of the pPink-HC-NHSSP clone. Surprisingly, we were not able to absorb secreted, mature NHSSP to several tested metal affinity resins (not shown). Thus we suspected that the C-terminus of recombinant NHSSP, containing His_6_-tag, is either buried and inaccessible within the protein structure or C-terminus processing takes place. To eliminate the possibilit, that the clone spontaneously mutated to eliminate the 3′-portion of the coding ORF, we isolated the *P. pastoris* clone genomic DNA and positively verified it by sequencing (not shown).

### Protein engineering

To determine the mature NHSSP borders and calculate the actual molecular weight of the mature protein, we conducted N- and C-terminal sequencing, as well as peptide mass fingerprinting. The N-terminus of the isolated, mature NHSSP was determined to be Ala-Leu-Thr-Thr-Gln-Pro-Asn-Ala, while the C-terminus is suggested to be Leu-Leu-Tyr-Asn-Gly-Ser (Additional files 13, 14). Taking the N- and the C-terminal aa into account as the borders of the mature enzyme, a molecular weight of 28.4 kDa was calculated. These results revealed processing at the C-terminus, leading to the elimination of 4 C-terminal aa along with the His_6_-tag. To explore further the expression clone improvements for biotechnology production purposes, a deletion mutant clone was constructed. As based on the high expression clone pPink-HC-NHSSP, a modified construct was made by PCR amplification of the truncated coding ORF prepro-NHSSP-His_6_ and cloning into pPink-HC vector following the same procedure as for full length NHSSP-His_6_. Consequently, the truncated ORF was devoid of the coding His_6_-tag 6 aa along with 4 C-terminal NHSSP aa, thus eliminating the need for C-terminal processing during maturation. The remaining ORF portion coded for preproNHSSP, thus retaining the SP and propeptide aa sequences, thus it allowed N-terminus utilization for secretion to the media and further maturation.

### Protein purification, activity and stability

**Fig. 2:**
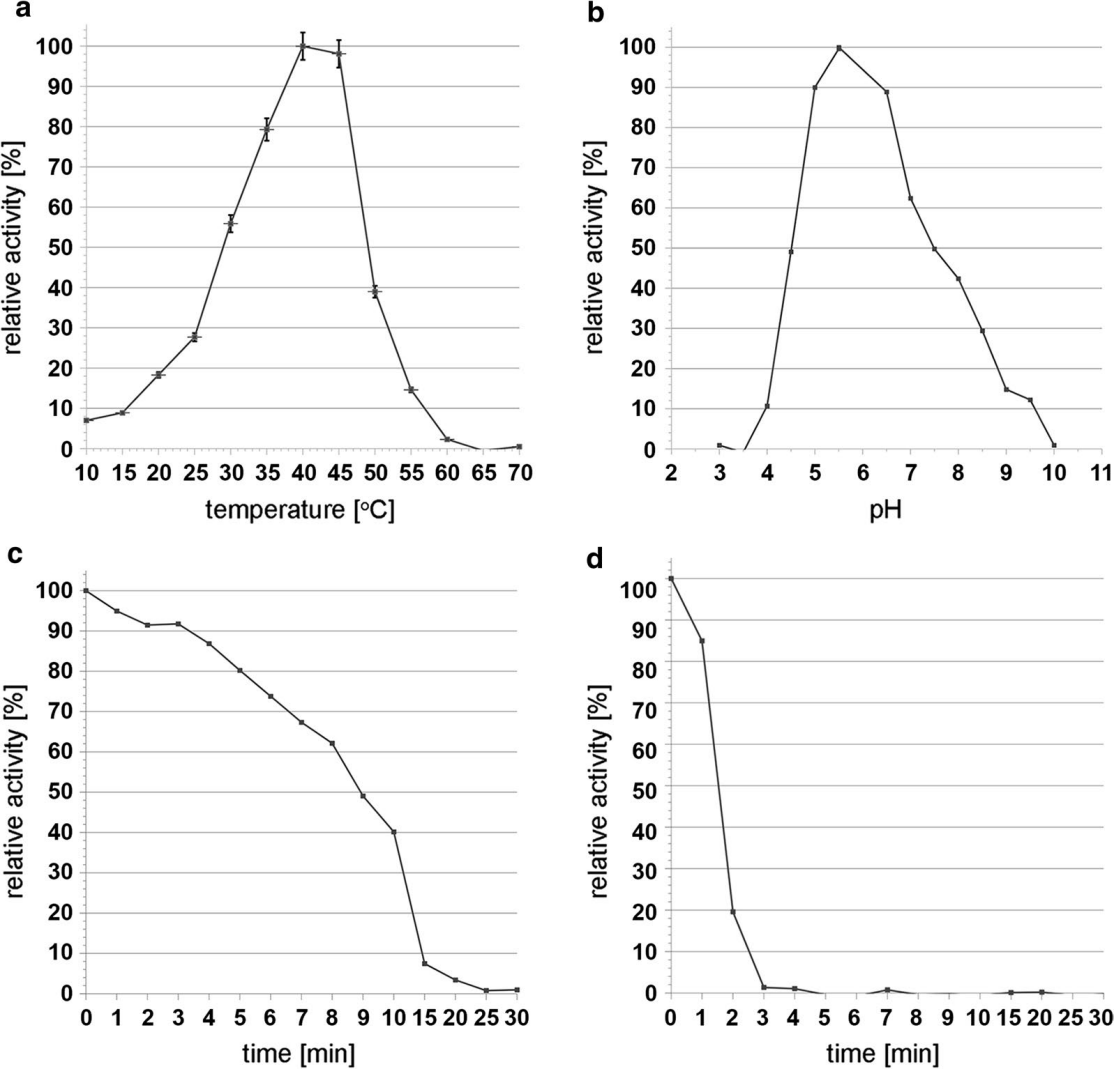
NHSSP temperature and pH activity dependence. **a** Temperature activity range and optimum. **b** Reaction pH activity range and optimum. **c** NHSSP inactivation time at 50 °C. **d** NHSSP inactivation time at 60 °C

Since the His_6_-tag was removed either during internal processing or through gene truncation, a simple protocol based on ammonium sulfate fractionation and hydrophobic affinity chromatography was developed instead of metal affinity chromatography for both NHSSP variants. Figure 1 summarizes NHSSP expression and purification expressed from the pPink-HC-NHSSP clone. The yield obtained was more than 300 mg of the mature NHSSP product present in the post-expression medium from the pPink-HC-NHSSP clone. The purified preparation of NHSSP was stable upon storage in phosphate-buffered 50% glycerol at − 80 °C for over 2 years with no detectable loss of activity. Calculations of the mature NHSSP apparent molecular weight were conducted on a series of SDS-PAGE gels (Fig. 1), resulting in values of up to 34 kDa, depending on gel concentration used. We estimate the purity of the enzyme to be about 95%, which was further used for the determination of critical reaction parameters, such as temperature and pH. Figure 2 shows the results of the assays at pH 6.5, indicating the mature NHSSP exhibits less than 10% maximum activity at tested temperature range extremes of 10 °C and 55 °C, with app. 20% or more activity in the range of 15–50 °C and optimum at 40 °C. At 35 °C to 45 °C at least approximately 40% of the activity was observed, while the optimal temperature range extended from 38 to 42 °C. A rapid decrease in activity was observed above 50-55 °C (Fig. 2a, d). Somewhat surprisingly, the pH activity range fell in the neutral to slightly acidic range, from at least pH 5.0 to pH 7.5, showing over 40% of the maximum activity at pH 6.8 at 40 °C. The pH optimum is between pH 5.4 and pH 7.3 (about 60% of the maximum activity), and the enzyme is most active between pH 6 and 7 (about 80% of the maximum activity) with an optimum at pH 6.8 (Fig. 2b). The upper temperature range was more closely investigated to evaluate the possibility of a rapid, relatively low temperature inactivation, which is of key importance in industrial applications. Two temperatures were tested: 50 °C and 60 °C. Unexpectedly, it was found that the enzyme is very heat-sensitive above a temperature of about 50 °C: at 50 °C it undergoes near complete inactivation within 15 min, while at 60 °C complete inactivation takes only 3 min (Fig. 2d).

**Fig. 3:**
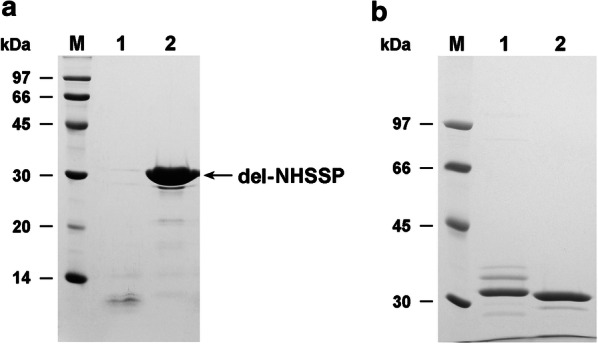
Purified NHSSP variants and SDS effect. **a** Purified del-NHSSP expressed by pPink-HC-del-NHSSP clone. Lane M, Amersham™ LMW Calibration Kit For SDS Electrophoresis; lane 1, NHSSP sample denatured by heating in Leammli electrophoresis loading buffer (with SDS); lane 2, NHSSP sample denatured by heating in Leammli electrophoresis loading buffer (with SDS) in the presence of 5 mM PMSF. **b** Very long electrophoresis run of purified NHSSP and del-NHSSP. Lane M, Amersham™ LMW Calibration Kit For SDS Electrophoresis; lane 1, NHSSP expressed by pPink-HC-NHSSP clone; lane 2, del-NHSSP expressed by pPink-HC-del-NHSSP clone

We tested the effect of several divalent metal cations: Mn^2+^, Co^2+^, Ni^2+^, Fe^2+^, Zn^2+^, Mg^2+^, Ca^2+^. Only Fe^2+^, Zn^2+^ showed minor stimulations of approx. 10–15% or less, close to the assay error, while Mn^2+^ and Ni^2+^ showed modest inhibitory effects (not shown). Considering the temperature and pH optima, the proteolytic activity of the NHSSP was evaluated in comparison to the widely used, broad specificity proteinase K. The comparative results of the specific activities of NHSSP, deletion derivative of NHSSP and proteinase K are given in Table 1. The mature NHSSPs show an approx. twofold higher specific activity. PMSF and SDS were identified as inhibitors of NHSSP, whereas EDTA, TCEP, Triton-X100, urea, DTT and β-mercaptoethanol did not significantly inhibit protease activity (Table 2). However, even though the effect of SDS at first glance seemed inhibitory (Table 2), in fact it is so highly stimulating to NHSSP to the point of rapid autodegradation, which can be mistaken for an inhibitory effect during activity assays (Fig. 3).

**Table 1:** Specific activities of NHSSPs compared to proteinase K

Protease specific activity (units/mg)		
Protease	Average	Standard deviation
NHSSP	63.9	7.6
del-NHSSP	44.3	6.5
proteinase K	31.9	5.8

**Table 2:** Effect of protease inhibitors and denaturing agents on the enzymatic activity of NHSSP

Reagent	Concentration	Relative activity (%)
none	–	100
SDS	1%	8
Triton	1%	98
Urea	4 M	95
DTT	5 mM	105
β-Mercaptoethanol	5 mM	99
PMSF	1 mM	40
EDTA	1 mM	100
TCEP	1 mM	97

### Comparative analysis of truncated del-NHSSP

**Fig. 4:**
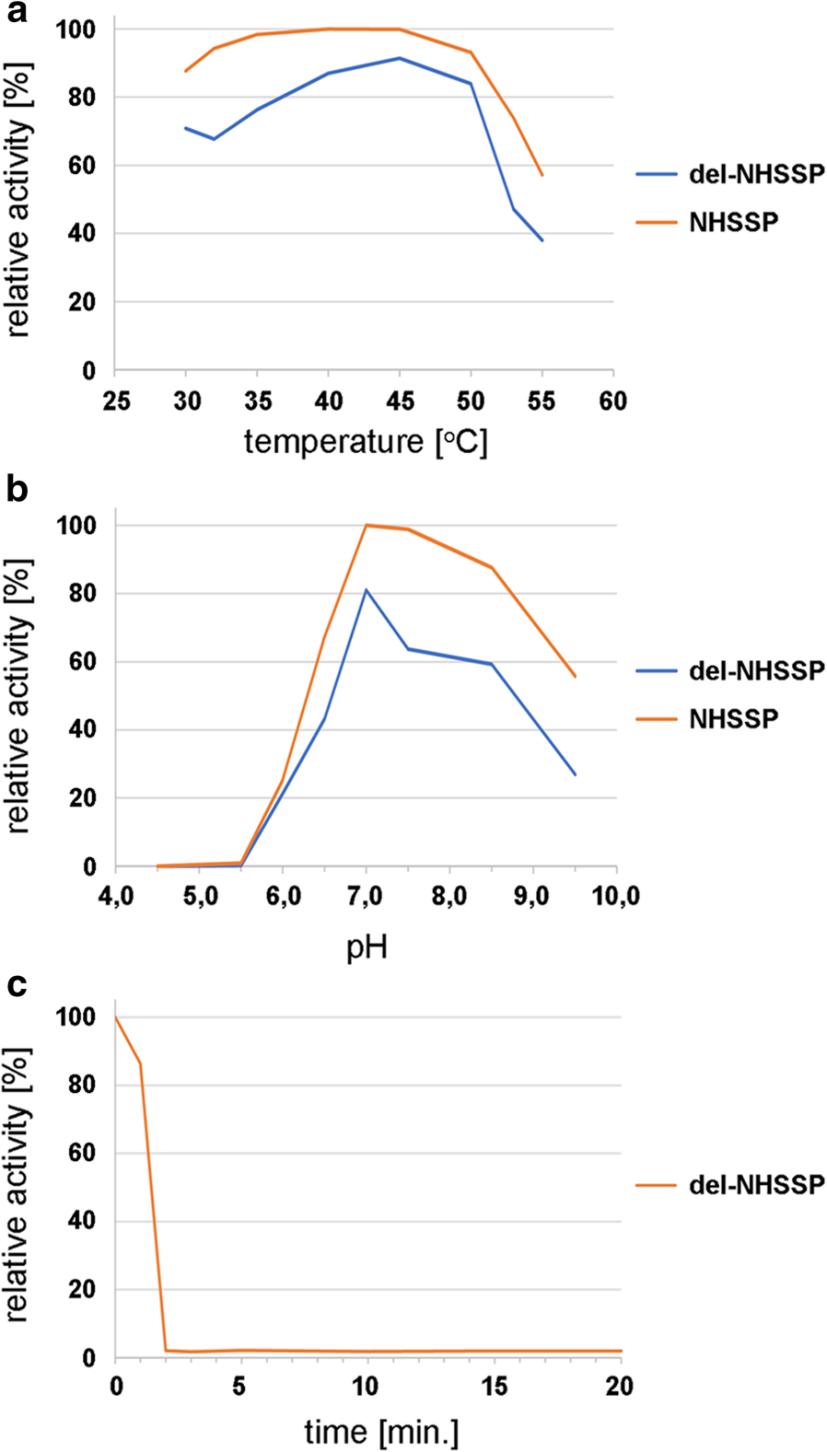
Comparison of range and optimal temperature and pH for activity of recombinant NHSSP and del-NHSSP. **a** Temperature effect. **b** pH effect. **c** del-NHSSP inactivation time at 60 °C

Engineering of the original expression clone pPink-HC-NHSSP eliminated the autoprocessing of the secreted protease at the C-terminus. Apparently, this is associated with a shorter time needed to obtain the maximum yield of the engineered C-deletion derivative of NHSSP. Peak expression from pPink-HC-del-NHSSP clone appeared considerably faster, after approx. 30 h post induction with methanol. After this time, the yield decreased. Following the original ammonium sulfate/Phenyl-Sepharose purification scheme with minor modifications, the truncated del-NHSSP was purified, as shown in Fig. 3. Lane 1 in Fig. 3a shows heavy degradation of purified del-NHSSP in the samples during incubation with SDS in preparation of the SDS-PAGE, when no PMSF was added. 5 mM PMSF effectively prevented SDS-induced autolysis of NHSSP protein (lane 2). Figure 3b shows a comparative electrophoresis run of NHSSP and del-NHSSP. We solved the problem of SDS sensitivity by applying TCA-precipitation before SDS treatment. There is a subtle difference in migration, only visible on very long electrophoresis runs. The purity of the obtained del-NHSSP preparation exceeded 95%. To verify the retention of the catalytic parameters of del-NHSSP, key factors like temperature and pH optima and inactivation temperatures were analysed in parallel with NHSSP using a slightly different substrate (AZCL-Casein) than for the initial analysis of NHSSP. The purpose of substrate change was to verify that the obtained results are not significantly affected by the substrate type used. Figure 4 shows that the results obtained are strikingly similar both for NHSSP and del-NHSSP: a broad range of maximum activity was observed between 35 and 50 °C and rapid decrease of activity above 50 °C. Complete inactivation of del-NHSSP at 60 °C takes only 2 min. The peak of enzymatic activity (90%) was observed at pH 7.0–7.5 and a broad spectrum of optimum enzymatic activity between pH 6.5 up to 9.0 with activity of least 50%. The specific activity of del-NHSSP is app. 70% of the NHSSP (Table 1).

### Functional applications of the NHSSP in protein sequencing

**Fig. 5:**
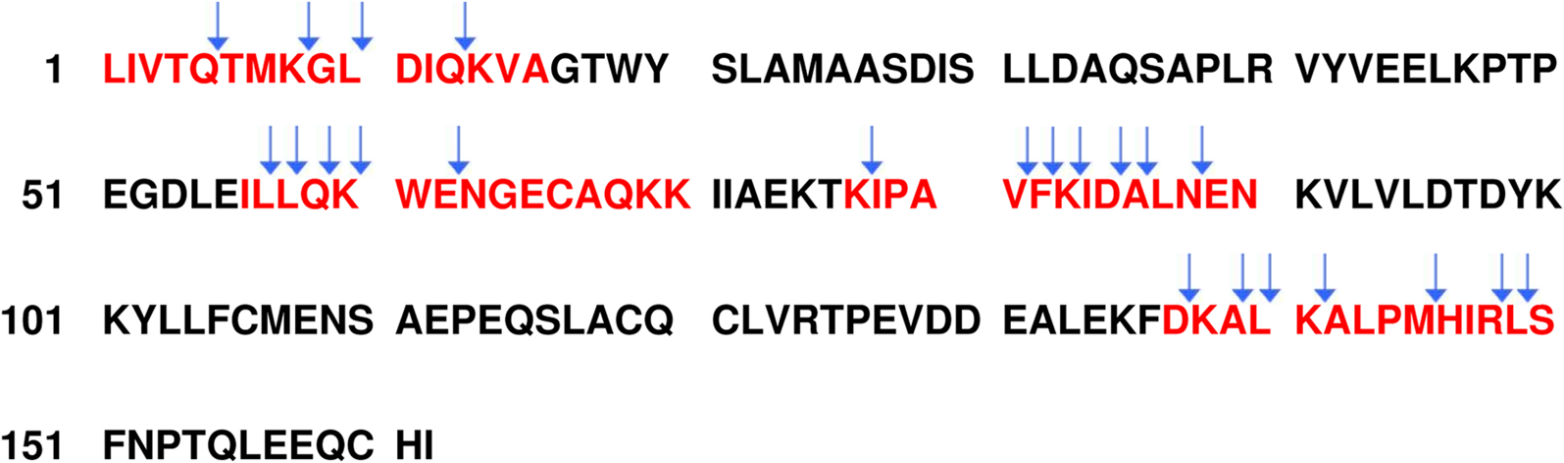
Cleavage sites within the allergenic epitopes of BLG. The cleavage site locations were obtained after cleavage of the protein at pH 6.0 and seen in nanoLC–ESI–MS/MS analysis. Arrows indicate the cleavage sites given in the fragment table for the digested protein of the Mascot Search results (allergenic region highlighted in red) (Additional file 15-5)

From studies regarding the sequence specificity [M. Drag, personal communication, 17] of the protease using synthetic peptides as substrates and short incubation times (5 min), we know about the superior enzyme kinetics, as well as some preference for cleavage on the C-side of phenylalanine (F) and of bulky aa by NHSSP. In addition, initial digestion experiments like BSA, BLG and monoclonal antibodies light and heavy chains (mAb LC and mAb HC), as well as longer incubation times (overnight), indicated a clear cleavage pattern if analysed by HPLC-MALDI (Fig. 5, Tables 3, 4, 5, 6, Additional files 15, 16, 17, 18). To test the suitability of the NHSSP enzyme for the characterization and confirmation of protein sequences, we digested these substrates and used LC–ESI–MS/MS to allow for the optimal resolution of peptide fragments giving rise to high sequence coverage, as smaller peptides will be detected more easily. In addition, we included digestion with trypsin as a reference.

**Table 3:** Sequence coverage obtained with different proteins and enzymes

Enzyme	Sequence coverage (nanoLC-ESI–MS/MS analytics)^a^			
	BSA (%)	BLG (%)	mAb HC (%)	mAb LC (%)
Trypsin	93	76	64	71
NHSSP	91	100	84	96

^a^Specificity R, K for trypsin and no specificity for NHSSP; ion cut-off 20

**Table 4:** Number of residues in test proteins (BLG and mAb) exhibiting NHSSP cleavage

Residue	BLG		mAb heavy chain		mAb light chain	
	# residues	% of possible residues (%)	# residues	% of possible residues (%)	# residues	% of possible residues (%)
A	*5*	*33.3*	1	4.2	1	6.7
C*	1	20.0	0	0.0	0	0.0
D	0	0.0	2	11.8	0	0.0
E	1	6.3	1	5.0	2	18.2
F	3	75.0	3	23.1	*4*	*57.1*
G	0	0.0	4	12.9	2	15.4
H	0	0.0	2	20.0	0	0.0
I	0	0.0	0	0.0	0	0.0
K	2	13.3	8	22.2	3	23.1
L	*12*	*54.5*	*8*	*25.0*	*4*	*30.8*
M	2	50.0	0	0.0	1	100
N	0	0.0	*6*	*31.6*	2	28.6
P	0	0.0	0	0.0	0	0.0
Q	*5*	*55.6*	4	22.2	*4*	*33.3*
R	1	33.3	0	0.0	1	16.7
S	2	28.6	7	13.2	7	20.0
T	0	0.0	3	7.9	3	17.6
V	1	11.1	3	7.0	1	6.3
W	0	0.0	1	11.1	*1*	25.0
Y	2	50.0	*6*	*27.3*	*6*	*66.7*

The aa that were found with a certain preference are in italics

If the minimum aa residue number was not reached, they are underlined

**Table 5:** Sequence coverage obtained for NHSSP at different pH conditions

Condition	Sequence coverage^a^			Average seq. coverage (%)	rSD (%)
	Replicate 1 (%)	Replicate 2 (%)	Replicate 3 (%)		
pH 6.5	6	6	11	8	30.7
pH 6.0	6	1	7	8	22.2
pH 5.0	6	3	3	4	35.4

^a^Cleavage specificity after F, Y and W, reference protein-BSA

**Table 6:** Sequence coverage obtained for NHSSP at different pH conditions with no enzyme specificity

Condition	Sequence coverage^a^			Average seq. coverage (%)	rSD (%)
	Replicate 1 (%)	Replicate 2 (%)	Replicate 3 (%)		
pH 6.5	72	81	97	83	12.4
pH 6.0	82	85	82	83	1.7
pH 5.0	86	70	76	77	8.5

^a^Reference protein-BSA

#### Sequence coverage

Mascot database searches of the nanoLC–ESI–MS/MS data of the enzymatic digests of BLG and mAb resulted in good sequence coverage of all proteins. However, the digestion with trypsin did result in clearly lower sequence coverage for BLG and mAb (Additional files 15, 16, 17, 18, and Table 3). The lower sequence coverage obtained with trypsin was not a result of less material applied to the instrument as confirmed by visualization of the mass spectra. Since trypsin cleaves with high specificity after arginine (R)  and lysine (K), a lower number of peptides with masses in the measurement range of the mass spectrometer (200–2000 m/z) can be expected, dependent on the sequence.

#### Cleavage specificity

From previous experiments, it was already known that NHSSP cleaves rather non-specifically than preferentially after aromatic aa residues as initially assumed. To gain further data about the potential specificity, or at least preferential cleavage sites, the detected peptides were analysed with regard to the C-terminal aa. It is believed that the cleavage motif would be defined as the C-terminal of a certain aa residue (like trypsin, chymotrypsin, endoprotease Glu-C or Lys-C) rather than N-terminal of a certain aa residue (like endoprotease Asp-N).

Preference for a certain aa was assumed if cleavage was detected after more than 25% of these aa in the protein sequence. Additionally, the total number of residues where cleavage was detected had to be more than three, in order to diminish the error. The aa that have been found with a certain preference are given in italics in the tables. If the minimum aa residue number was not reached, they are underlined.

The results seen in Table 4 suggest that NHSSP may have a certain preference for L and (tyrosine) Y within the tested proteins although we rather conclude that the protease seems to be rather non-specific in the choice of aa. Cleavage was detected after almost all aa (Additional files 15, 16, 17, 18, 19, 20; Fig. 5; Tables 3, 4, 5, 6). Nevertheless, the application of optimized cleavage conditions did not result in totally shredded proteins with only peptides below the detected range. Remarkably, a good distribution of differentially sized peptides was detected that resulted in high sequence coverage.

#### Determination of the optimal pH range

As the determination of the pH optimum showed a broad activity centring around pH 7.0, we were curious about the performance at lower pH. Therefore, the activity of the enzyme was assessed using BSA under more acidic cleavage conditions (pH 6.5, 6.0 and 5.0) that are a prerequisite for the characterization of posttranslational modifications of proteins, since the introduction of artificial protein modifications (such as deamidations) during sample handling is significantly reduced below pH 7.0.

Applying no enzyme specificity in the Mascot data base searches yielded in a high sequence coverage, with values > 70% at all tested pH conditions as seen in Tables 5 and 6 whereas searches with enzyme cleavage specificity after F, Y and tryptophan (W) resulted in only low sequence coverage (3–11%, data not shown). The very similar results obtained for all experiments indicate that there was no obvious correlation between pH conditions and sequence coverage. However, some variation was detected between the three replicates with identical cleavage conditions as expressed in values of relative standard deviation (rSD) up to 12.4% if searches were conducted with no enzyme specificity. These values were even higher with cleavage specificity after F, Y and W, due to the low number of assigned peptides.

It was found that for none of the aa F, leucine (L) and serine (S) that were previously observed as preferential cleavage sites of NHSSP with BSA were affected by a decrease of pH. In contrast, cleavage after Y, which appears in Table 4 to be one of the few preferred cleavage sites, occurred less often at lower pH conditions. Other aa that appeared less often cleaved at pH conditions close to pH 5.0 are alanine (A), F, K and threonine (T), but these aa did not appear to be preferential cleavage sites, therefore the observed changes could well be coincidence. No cleavage apparently occurs on the C-terminal side of proline (P) under all pH conditions tested.

## Discussion

The cloning, expression, protein engineering, purification and characterization have been undertaken to provide a novel enzymatic tool for proteolytic applications in industry and research. The NHSSP may be used in detergent, protein, brewing, bakery, meat, photographic, leather, dairy and pharmaceutical industries [4]. It may further be used as an alternative to chemicals to convert fibrous protein waste to useful biomass, protein concentrate or aa [6]. Work conducted by Huang et al. [8], and Lange et al. [18] had indicated that feather-digesting fungus *O. corvina* possesses numerous proteases, which can potentially offer advantages over currently used ones, especially proteinase K. We have cloned and expressed in the yeast *P. pastoris* 5 putative proteases genes from *O. corvina* (not shown), out of which protease 6877 (NHSSP) displayed unique features as described above. The choice of the PichiaPink™ host was critically important, as for industrial production purposes it is desirable to obtain enzymes secreted from cells, which are then easily removed by centrifugation or filtration. In addition, similar protein modification pathways in yeast and fungus *O. corvina* are expected to be functional. A first variant of the synthetic, optimized protease 6877 gene yielded high expression and enzymatic activity of the secreted NHSSP, when cloned in PichiaPink™. However, the results of N- and C-terminal aa sequencing of mature NHSSP-His_6_ revealed an unexpected type of processing of the pre-proprotein, where 10 aa from C-terminus were removed. As serine proteases are generally expressed as inactive precursors, which are activated by removal of the signal sequence and the pro-sequence to yield an active mature enzyme [3], the activation process needs to be conducted by autocatalysis or through the presence of another protease. As we observed full processing of the recombinant NHSSP, the maturation has to be conducted by the enzyme itself. The NHSSP has a calculated molecular mass of 28.36 kDa. This value is significantly lower than the apparent molecular weight of app. 34 kDa, as determined by SDS-PAGE analysis. However, since the NHSSP did not bind to affinity resins exposing chelated Ni^2+^ ion, its N- and C-terminal aa sequence was verified by aa sequencing and peptide mass fingerprinting, it is safe to conclude that the molecular weight discrepancy comes from atypical NHSSP migration on denaturing SDS-PAGE gels. It is known, that some proteins migrate on SDS-PAGE gels not precisely proportionally to their molecular weight, which causes even 10–20% error in molecular weight estimations. Alternatively, the possibility cannot be excluded that the mature NHSSP undergoes post-translational modifications, such as glycosylation, in *P. pastoris*. Since the C-terminal processing has eliminated the possibility for application of immobilized metal affinity chromatography in the NHSSP purification, we developed a simple, easily scalable purification protocol, including ammonium sulfate fractionation and hydrophobic chromatography. As an alternative to self-cleavage of the C-terminus by NHSSP, the shortening of the enzyme may also be achieved by truncating the encoding gene. Thus, a deletion derivative clone was made, mimicking the C-terminal processing. Both enzyme variants were highly active, with strikingly similar temperature and pH activity curves, as well as inactivation temperatures. There were differences between the two variants: (i) expression cultures had different expression kinetics, namely the full length NHSSP clone kept accumulating the mature NHSSP protein over two times longer than the truncated NHSSP clone. This could be attributed to faster maturation, where only the propeptide needed to be clipped off, thus the active truncated NHSSP was subjected to faster autodigestion during its prolonged presence in the culture medium. This feature however is beneficial for industrial production, as it allows a significantly reduction in cultivation time and costs; (ii) The truncated NHSSP migrated as an apparently minimally shorter protein on SDS-PAGE gels. The difference was subtle and visible only on much prolonged runs, completed just before the proteins bands were about to leave the gels. The nature of that is not known, maybe attributed to different proteins conformations in the gels or del-NHSSP is further processed, resulting in just a few more aa removed. The determined reaction parameters for both NHSSP variants, e.g.: activity at neutral to acidic pH of 5.0 to 8.5, with optimum at 6.8, temperature activity range of 15 to 50 °C and fast deactivation above 50 °C, are particularly attractive for both industrial and scientific applications.

In order to gain access to such applications we tested the cleavage properties and performance of the enzyme by digestion of standard proteins like BSA, BLG or monoclonal antibodies and by analysis of the fragment pattern. The enzyme showed a high cleavage efficiency resulting in superior sequence coverage for example in comparison to trypsin. There is no apparent cleavage specificity under conditions of overnight digestions. The performance appears to be robust over a broad pH range with still good yields at pH 5.0. The apparent lack of cleavage specificity may not qualify the enzyme for standard peptide mass fingerprinting, since high enzyme specificity is needed for robust protein identification based on peptide masses only.

In contrast, as a tool for sequence confirmation and peptide mapping of proteins the NHSSP can be very useful. The lack of specificity and therefore cleavage of any kind of protein independent of its sequence is advantageous over other proteases, for example such as trypsin or chymotrypsin. LC–ESI–MS/MS measurement allows protein and peptide identification based on peptide and fragment ions, therefore no cleavage specificity is required. Another advantage of the enzyme is the very high reproducibility of cleavage resulting in highly comparable fragment patterns as shown in the nanoLC chromatograms.

Additionally, the enzyme appears to be beneficial for the analysis of posttranslational modifications such as deamidations since it works with high efficiency in buffers below pH 7.0. This enables a detailed analysis of the defined residues with regard to their modifications with minimal risk of introducing artificial modification of the protein, as it is commonly known to happen in buffers with higher pH. This feature may be very helpful in the identification of modifications, for example in the quality control of GMP material of clinical samples of antibodies and other proteinaceous medical entities.

The excellent sequence coverage and interpretation of the fragment pattern obtained for lactoglobulin suggests an interesting application for the enzyme. A side effect, for example of chemical preparation of milk hydrolysates from whey as used in baby food, food substitutes in clinics or for sportsman and astronauts, is the appearance of bitter peptides. One of the best characterized is the pentapeptide with the sequence N-IPAVF-C [19] derived from the most abundant protein in whey, lactoglobulin. This pentapeptide locates at position 78–82 in the sequence of lactoglobulin. Although this pentapeptide or possibly its smaller fragments are too small for detection with the experimental setting of the mass analysis used in this report we could detect a series of 26 longer fragments between amino acid 70 and 82 that contain the pentapeptide. The obtained fragment pattern suggests that the enzyme releases fragments that mask the bitter sequence and therefore hydrolysates derived from digestion with NHSSP may not exhibit a bitter taste. We are currently testing this application option. As the enzyme is rapidly inactivated at temperatures above 50 °C it will consequently not survive a routine pasteurization step done at 72–75 °C. This may support the application in the processing of milk proteins.

An additional application aspect can be derived from the analysis of the fragment patterns of BLG obtained from the sequencing approach. The enzyme cleaves the protein in areas that are known to exhibit allergenic epitopes. Essentially four regions were shown to bind to IgE antibodies mapping around amino acids 1–16, 56–70, 76–90 and 136–150, respectively [20]. Figure 5 shows the cleavage sites used by NHSSP in the antigenic region of BLG. Confirmed cleavage sites are seen at position 5, 8, 10, 13 within antigenic peptide 1, at position 57, 58, 59, 60, 62, for antigenic peptide region 2, at position 77, 81, 82, 83, 85, 86, 88 for antigenic peptide region 3 and at position 137, 139, 140, 141, 145, 146, 148, 149 for antigenic region 4. As the enzymes cleaved BLG within all known allergenic regions we therefore conclude that the appearance of allergenic peptides in milk hydrolysates can be avoided by application of NHSSP at least if they are generated from BLG, the most abundant milk protein. We are currently testing the other main milk proteins like different α-lactalbumins and different caseins.

## Conclusions

1. The gene coding for protease 6877 (NHSSP) from feather-degrading fungus *O. corvina* has been cloned and expressed in yeast and an efficient purification protocol was developed.
2. The enzyme underwent an atypical maturation process, including clipping of both N- and C-termini.
3. The purified enzyme exhibited unique features, offering advantages over currently used ones, especially proteinase K, thus it is useful as a novel enzymatic tool for proteolytic applications in industry and research.
4. The NHSSP features include: activity at neutral to acidic pH of 5.0 to 8.5, with optimum at 6.8, temperature activity range of 15 to 50 °C, fast deactivation above 50 °C, no cleavage specificity.
5. In protein analyses the enzyme exhibited a high cleavage efficiency and no apparent cleavage specificity, resulting in superior sequence coverage compared to trypsin. The performance is robust over a broad pH range.
6. The NHSSP is useful in a number of applications, such as: protein sequencing, LC–MS analysis, posttranslational modifications analysis, quality control of GMP material, food processing, waste treatment, among others.

## Supplementary information

**Additional file 1.** Synthetic DNA fragment map, sequence, translation and features, coding for optimized full length SP-PRO-NHSSP-His_6_ gene. Secretion peptide native—marked in green, propeptide—marked in yellow, His_6_-tag—marked in red.

**Additional file 2.** Synthetic DNA fragment map and features, comprising optimized full length SP-PRO-NHSSP-His_6_ gene. Secretion peptide native—marked in green, propeptide—marked in yellow, His_6_-tag—marked in red.

**Additional file 3.** Synthetic DNA fragment sequence, translation and features, comprising optimized full length SP-PRO-NHSSP-His_6_ gene. Secretion peptide native—marked in green, propeptide—marked in yellow, His_6_-tag—marked in red.

**Additional file 4.** pPink-HC-NHSSP plasmid construct sequence, translation and features. Secretion peptide native—marked in green, propeptide—marked in yellow, His_6_-tag—marked in red.

**Additional file 5.** pPink-HC-NHSSP plasmid construct map and features. Secretion peptide native—marked in green, propeptide—marked in yellow, His_6_-tag—marked in red.

**Additional file 6.** pPink-HC-NHSSP plasmid sequence and features. Secretion peptide native—marked in green, propeptide—marked in yellow, His_6_-tag—marked in red.

**Additional file 7.** Synthetic DNA fragment map, sequence, translation and features, coding for optimized SP-PRO-NHSSP gene with deleted C-terminal 4 aa and His_6_-tag. Secretion peptide native—marked in green, propeptide—marked in yellow.

**Additional file 8.** Synthetic DNA fragment map and features, comprising optimized SP-PRO-NHSSP gene with deleted C-terminal 4 aa and His_6_-tag. Secretion peptide native—marked in green, propeptide—marked in yellow.

**Additional file 9.** Synthetic DNA fragment sequence, translation and features, comprising optimized SP-PRO-NHSSP gene with deleted C-terminal 4 aa and His_6_-tag. Secretion peptide native—marked in green, propeptide—marked in yellow.

**Additional file 10.** pPink-HC-del-NHSSP plasmid construct sequence, translation and features. Secretion peptide native—marked in green, propeptide—marked in yellow.

**Additional file 11.** pPink-HC-del-NHSSP plasmid construct map and features. Secretion peptide native—marked in green, propeptide—marked in yellow.

**Additional file 12.** pPink-HC-del-NHSSP plasmid sequence and features. Secretion peptide native—marked in green, propeptide—marked in yellow.

**Additional file 13.** MASCOT Search Results of mature NHSSP.

**Additional file 14.** N- and C-terminal sequencing results of mature NHSSP.

**Additional file 15.** BLG digest with trypsin and matched protein sequence. BLG digest with NHSSP and matched protein sequence. Monoclonal mAb digest with trypsin and matched protein sequences. Monoclonal mAb digest with NHSSP and matched protein sequences. Cleavage sites around the bitter peptide region of BLG.

**Additional file 16.** MASCOT Search Results of NHSSP-cleaved BLG.

**Additional file 17.** MASCOT Search Results of NHSSP-cleaved monoclonal mAb heavy chain.

**Additional file 18.** MASCOT Search Results of NHSSP-cleaved monoclonal mAb light chain.

**Additional file 19.** HPLC elution profiles of NHSSP-cleaved BLG.

**Additional file 20.** HPLC elution profiles of NHSSP-cleaved monoclonal mAb.

## Data Availability

The raw data required to reproduce these findings are available to download as Additional files of this paper. The processed data required to reproduce these findings are available to download as Additional files of this paper.
