# Supplementary figures and images for: An alternative for proteinase K-heat-sensitive protease from fungus Onygena corvina for biotechnology: cloning, engineering, expression, characterization and special application for protein sequencing
Source: Microb Cell Fact. 2020 Jun 24;19:135. doi: 10.1186/s12934-020-01392-3 (PMC7313183; doi:10.1186/s12934-020-01392-3)

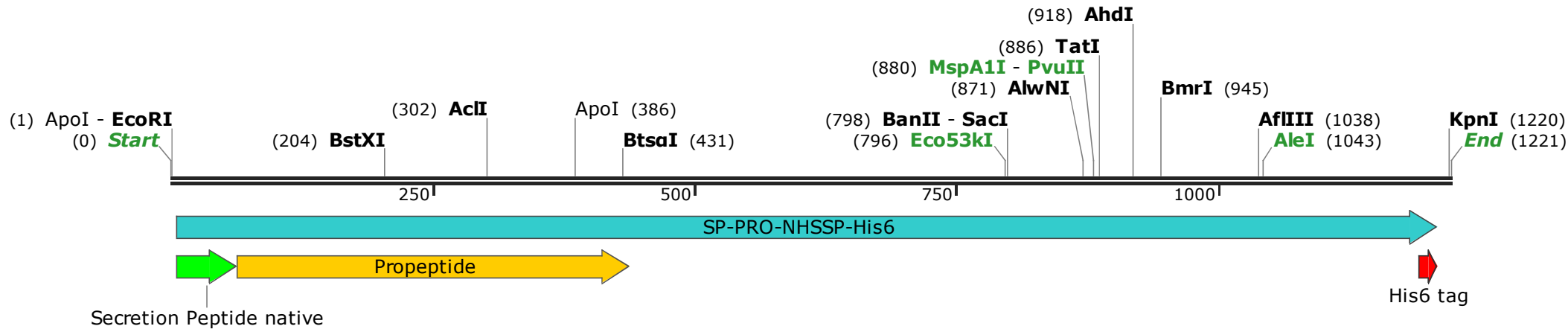

**SP-PRO-NHSSP-His6**  
1221 bp

Supplement: Supplementary file 2 — Additional file 2. Synthetic DNA fragment map and features, comprising optimized full length SP-PRO-NHSSP-His6 gene. Secretion peptide native—marked in green, propeptide—marked in yellow, His6-tag—marked in red. [file 12934_2020_1392_MOESM2_ESM.pdf]

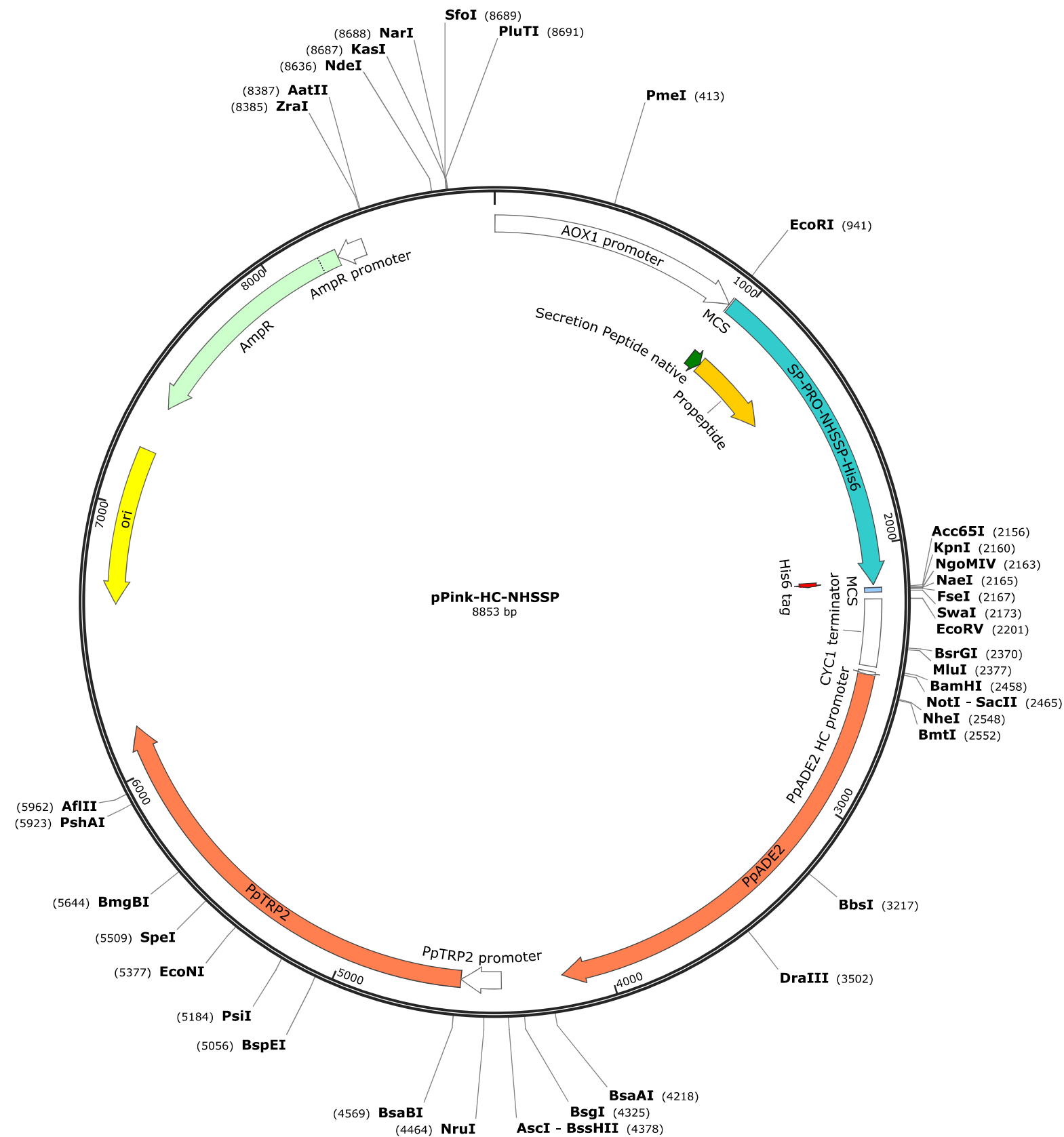

Supplement: Supplementary file 5 — Additional file 5. pPink-HC-NHSSP plasmid construct map and features. Secretion peptide native—marked in green, propeptide—marked in yellow, His6-tag—marked in red. [file 12934_2020_1392_MOESM5_ESM.pdf]

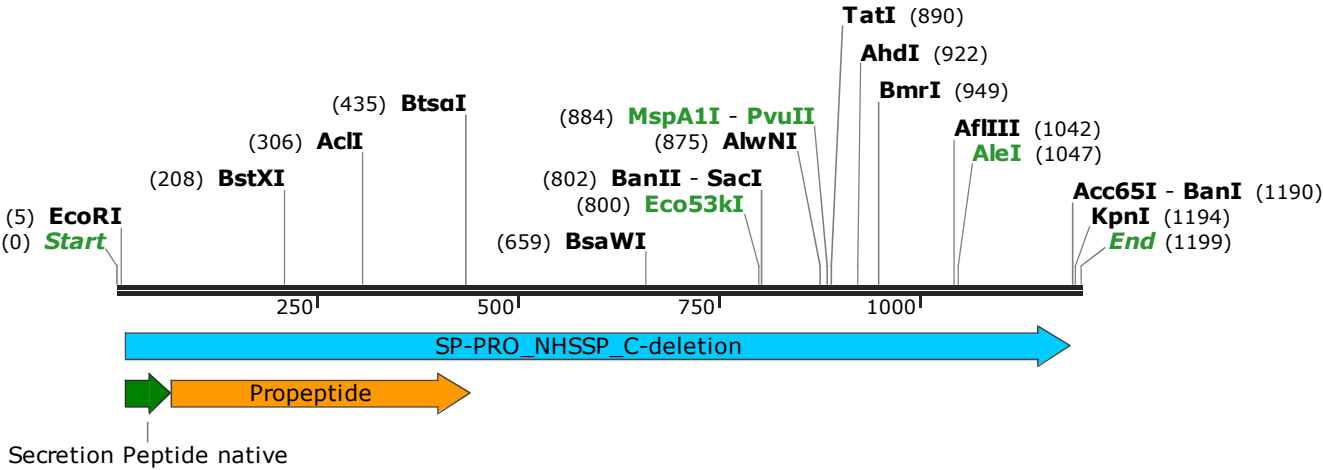

**SP-PRO-NHSSP\_C-deletion**  
1199 bp

Supplement: Supplementary file 8 — Additional file 8. Synthetic DNA fragment map and features, comprising optimized SP-PRO-NHSSP gene with deleted C-terminal 4 aa and His6-tag. Secretion peptide native—marked in green, propeptide—marked in yellow. [file 12934_2020_1392_MOESM8_ESM.pdf]

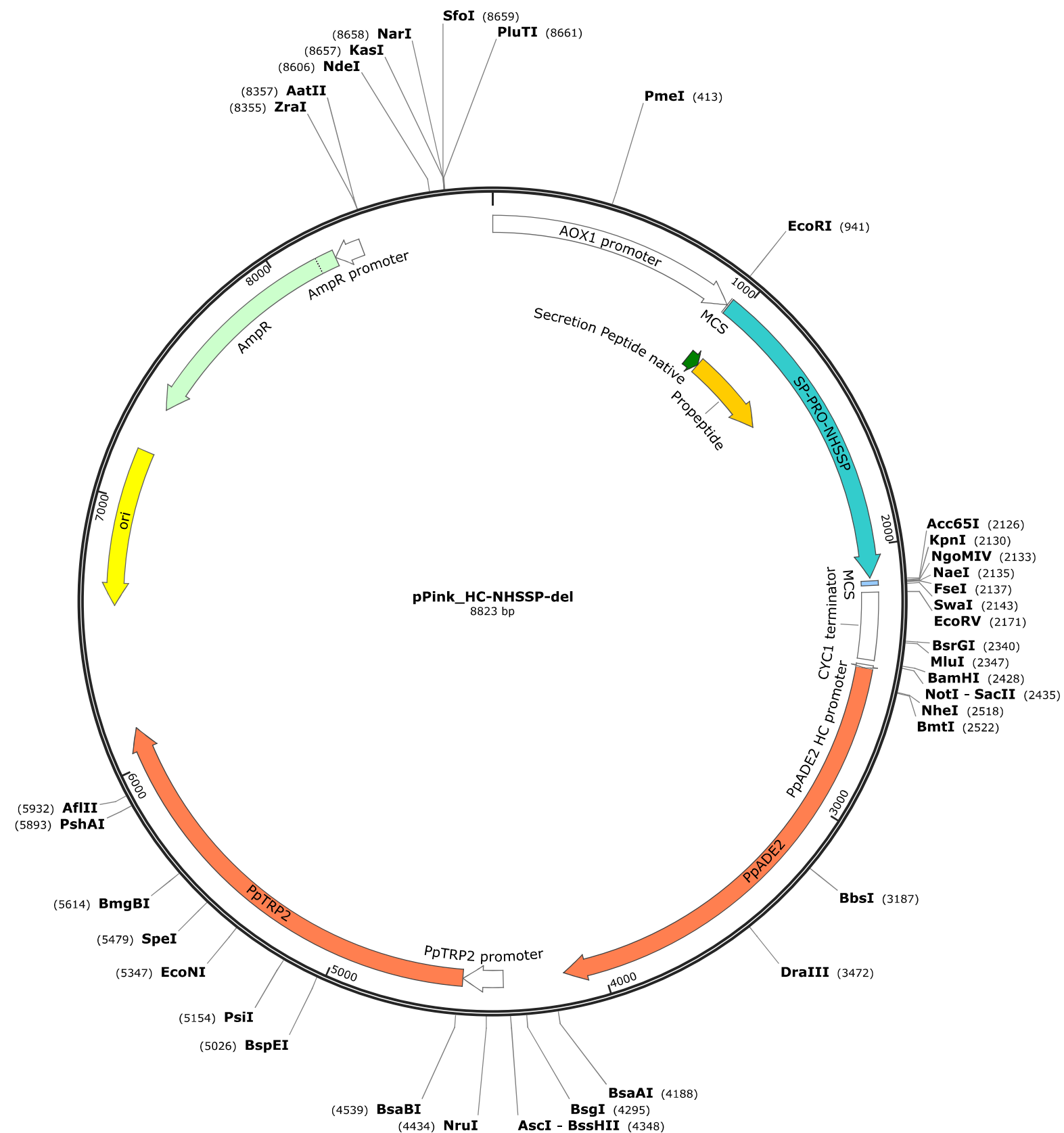

Supplement: Supplementary file 11 — Additional file 11. pPink-HC-del-NHSSP plasmid construct map and features. Secretion peptide native—marked in green, propeptide—marked in yellow. [file 12934_2020_1392_MOESM11_ESM.pdf]

XIC (base peak), m/z: 200.0000 - 2000.0000

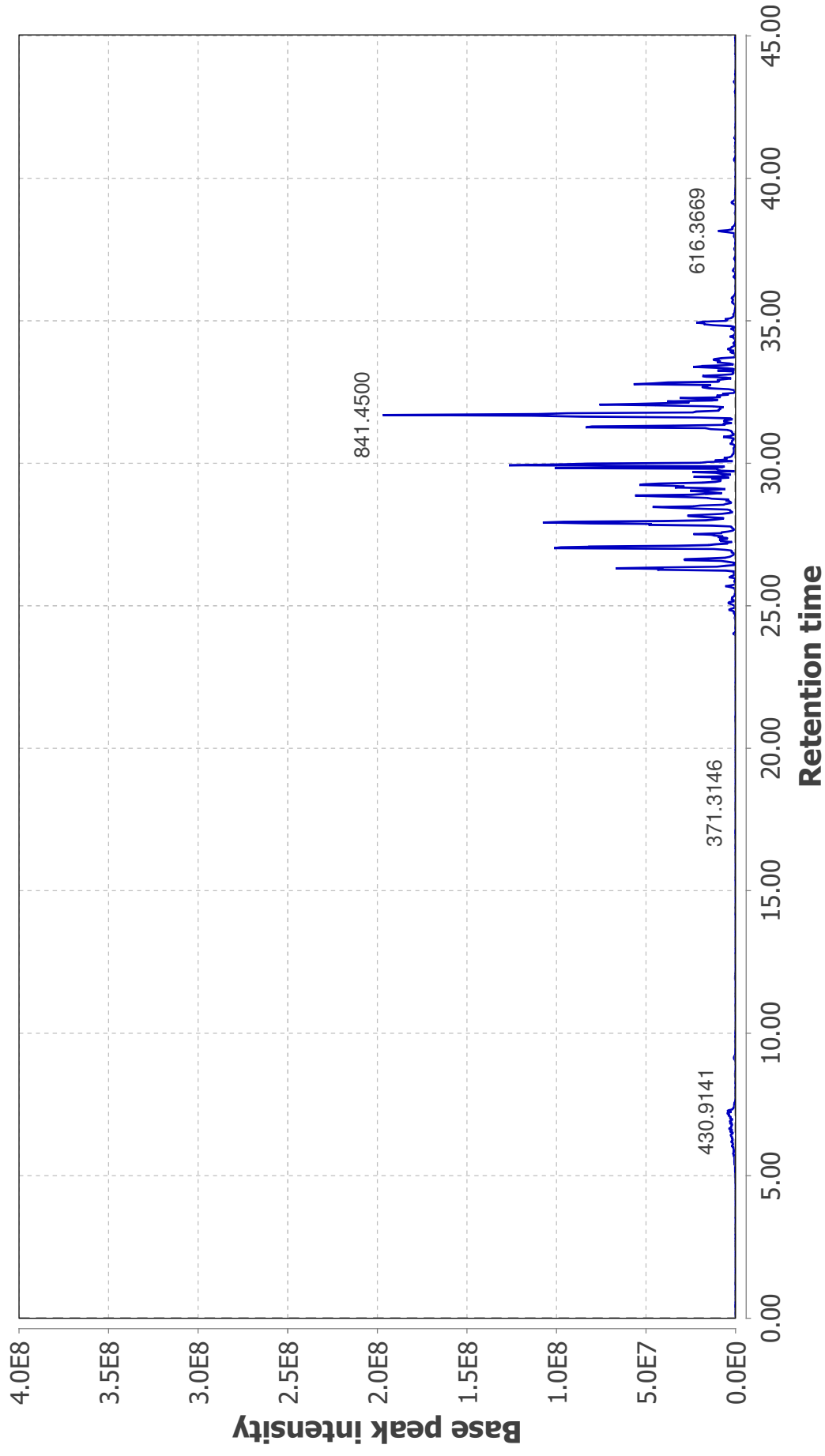

— 8375\_BLG\_HL1\_1210nl.raw

Supplement: Supplementary file 19 — Additional file 19. HPLC elution profiles of NHSSP-cleaved BLG. [file 12934_2020_1392_MOESM19_ESM.pdf]

**XIC (base peak), m/z: 200.0000 - 2000.0000**

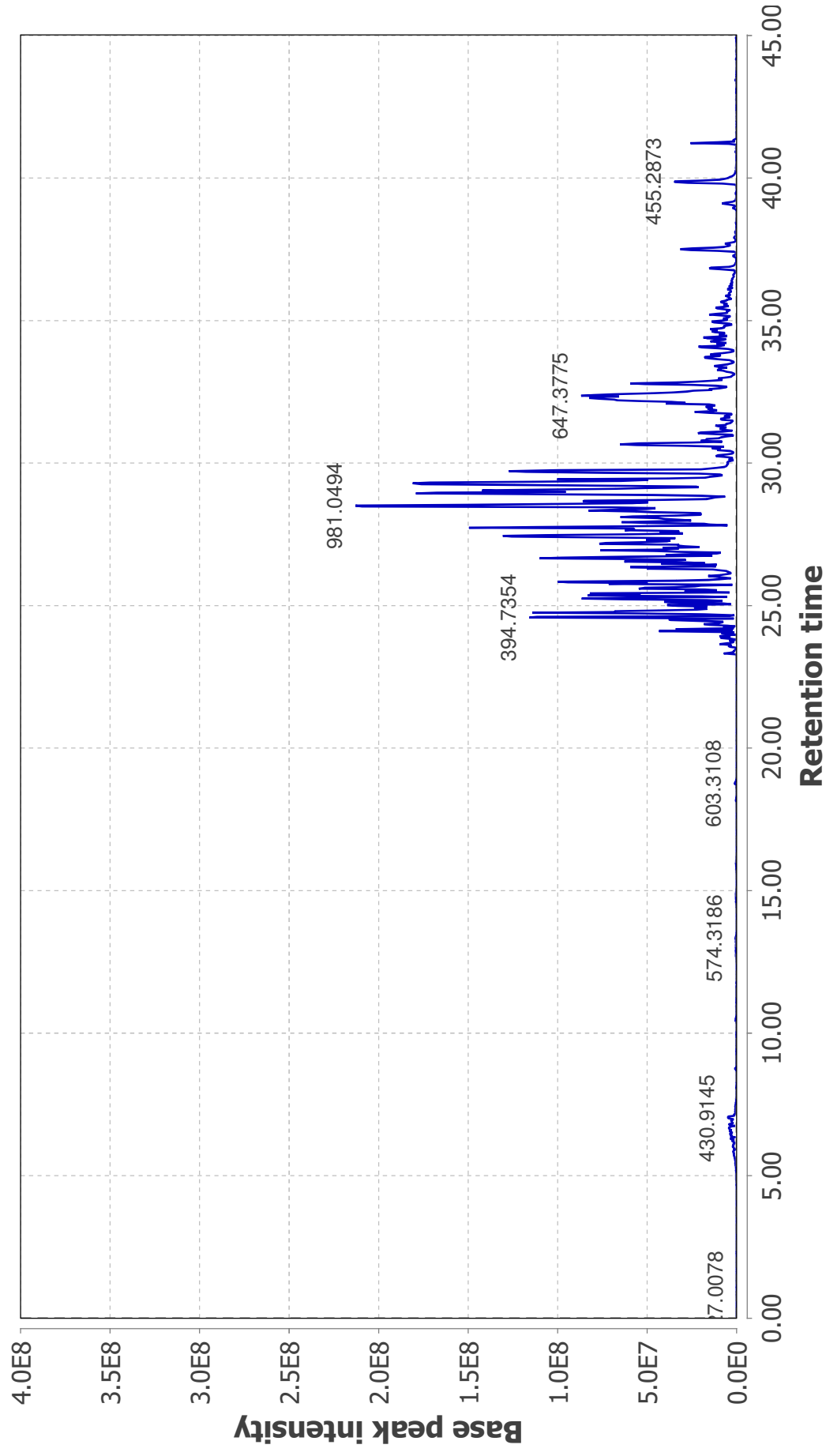

— 8375\_mAB\_HL1\_1070nl.raw

Supplement: Supplementary file 20 — Additional file 20. HPLC elution profiles of NHSSP-cleaved monoclonal mAb. [file 12934_2020_1392_MOESM20_ESM.pdf]
