# Supplementary material for: An alternative for proteinase K-heat-sensitive protease from fungus Onygena corvina for biotechnology: cloning, engineering, expression, characterization and special application for protein sequencing
Source: Microb Cell Fact. 2020 Jun 24;19:135. doi: 10.1186/s12934-020-01392-3 (PMC7313183; doi:10.1186/s12934-020-01392-3)
Supplement: Supplementary file 3 — Additional file 3. Synthetic DNA fragment sequence, translation and features, comprising optimized full length SP-PRO-NHSSP-His6 gene. Secretion peptide native—marked in green, propeptide—marked in yellow, His6-tag—marked in red. [file 12934_2020_1392_MOESM3_ESM.pdf]

Start (0)

ApoI
EcoRI

5′ GAATTCATGGGTTGTATCAAGGTTATCTCCGTTTTCTTGGCTGCTGTTGCTGCTGTTGACGCTAGAGCTTTTTTC 75
3′ CTTAAGTACCCAACATAGTTCCAATAGAGGCAAAAGAACCGACGACAACGACGACAACTGCGATCTCGAAAAAAG

1 5 10 15 20
M G C I K V I S V F L A A V A A V D A R A F F
SP-PRO-NHSSP-His6
Secretion Peptide native
Propeptide

CACAACAAGGGTGGTAACGACATCATCCCAAACTCCTACATCGTTGTTATGAAGGACGGTATCTCCACTCAAGAG 150
GTGTTGTTCCCACCATTGCTGTAGTAGGGTTTGAGGATGTAGCAACAATACTTCCTGCCATAGAGGTGAGTTCTC

25 30 35 40 45
H N K G G N D I I P N S Y I V V M K D G I S T Q E
SP-PRO-NHSSP-His6
Propeptide

BstXI

TTCGAGTCCCACATTTCTTCCGTTTCTGCTACTCACGCTAAGAGATCCACTGAGTTGGTTGGTCACAAGGATTCC 225
AAGCTCAGGGTGTAAAGAAGGCAAAGACGATGAGTGCGATTCTCTAGGTGACTCAACCAACCAGTGTTCCTAAGG

50 55 60 65 70
F E S H I S S V S A T H A K R S T E L V G H K D S
SP-PRO-NHSSP-His6
Propeptide

TTCAACATCAACGGTTGGAGAGCTTACAACGGTCACTTCGATGCTGCTACTTTGGAGTCCATTTTGAACGACGAC 300
AAGTTGTAGTTGCCAACCTCTCGAATGTTGCCAGTGAAGCTACGACGATGAAACCTCAGGTAAAACTTGCTGCTG

75 80 85 90 95
F N I N G W R A Y N G H F D A A T L E S I L N D D
SP-PRO-NHSSP-His6
Propeptide

AclI

AACGTTAAGTACGTTGAGCACGACAGAGTTGTTAAGATCTCCGCTTTGACTACTCAGCCAAACGCTCCATCTTGG 375
TTGCAATTCATGCAACTCGTGCTGTCTCAACAATTCTAGAGGCGAAACTGATGAGTCGGTTTGCGAGGTAGAACC

100 105 110 115 120
N V K Y V E H D R V V K I S A L T T Q P N A P S W
SP-PRO-NHSSP-His6
Propeptide

ApoI **BtsαI**

GGTTTGGGTAGAATTTCTCACAGATCCCCAGGTAACAAGGACTTCGTTTACGATGACACTGCTGGTCAGGGTATC 450
CCAAACCCATCTTAAAGAGTGTCTAGGGGTCCATTGTTCCTGAAGCAAATGCTACTGTGACGACCAGTCCCATAG

125 130 135 140 145
G L G R I S H R S P G N K D F V Y D D T A G Q G I
SP-PRO-NHSSP-His6
Propeptide

ACTATCTACGGTGTTGACACTGGAATCGACATCAACCACCCAGATTTTGGTGGTAGAGCTAGATGGGGAACTAAC 525
TGATAGATGCCACAACTGTGACCTTAGCTGTAGTTGGTGGGTCTAAAACCACCATCTCGATCTACCCCTTGATTG

150 155 160 165 170
T I Y G V D T G I D I N H P D F G G R A R W G T N
SP-PRO-NHSSP-His6

ACTGTTGACAACGCTAACAACGACGGTCATGGTCATGGTACTCATACTGCTGGTACTTTCGCTGGTAACGCTTAC 600
TGACAACTGTTGCGATTGTTGCTGCCAGTACCAGTACCATGAGTATGACGACCATGAAAGCGACCATTGCGAATG

175 180 185 190 195
T V D N A N N D G H G H G T H T A G T F A G N A Y
SP-PRO-NHSSP-His6

```
GGTATTGCTAAGAAGGCTTCCGTTGTTGCTGTTAAGGTTTTGTCTGCTTCTGGTTCCGGTTCCAACGCTGGTGTT   675
CCATAACGATTCTTCCGAAGGCAACAACGACAATTCCAAAACAGACGAAGACCAAGGCCAAGGTTGCGACCACAA
   200            205            210            215            220
G  I  A  K  K  A  S  V  V  A  V  K  V  L  S  A  S  G  S  G  S  N  A  G  V
SP-PRO-NHSSP-His6

ATTAAGGGTATCGACTGGTGTGTTACAGACGCTAGATCTAAGGGTGCTTTGGGTAAGGCTGCTTTGAACTTGTCT   750
TAATTCCCATAGCTGACCACACAATGTCTGCGATCTAGATTCCCACGAAACCCATTCCGACGAAACTTGAACAGA
   225            230            235            240            245
I  K  G  I  D  W  C  V  T  D  A  R  S  K  G  A  L  G  K  A  A  L  N  L  S
SP-PRO-NHSSP-His6

                                                                  SacI
                                                        Eco53kI   BanII
TTGGGTGGTGGTTTCAACCAGGCTACAAACGACGCTGTTACTAGAGCTCAGACTGCTGGAATCTTCGTTGCTGTT   825
AACCCACCACCAAAGTTGGTCCGATGTTTGCTGCGACAATGATCTCGAGTCTGACGACCTTAGAAGCAACGACAA
   250            255            260            265            270
L  G  G  G  F  N  Q  A  T  N  D  A  V  T  R  A  Q  T  A  G  I  F  V  A  V
SP-PRO-NHSSP-His6

                                                                  PvuII
                                                   AlwNI          MspA1I TatI
GCTGCTGGTAACGATAACAAGGACGCTAGAAACTACTCTCCAGCTTCTGCTCCAGCTGTTTGTACTGTTGCTTCC   900
CGACGACCATTGCTATTGTTCCTGCGATCTTTGATGAGAGGTCGAAGACGAGGTCGACAAACATGACAACGAAGG
   275            280            285            290            295
A  A  G  N  D  N  K  D  A  R  N  Y  S  P  A  S  A  P  A  V  C  T  V  A  S
SP-PRO-NHSSP-His6

                    AhdI                         BmrI
TCCACTATCGACGACCAAAAGTCCTCTTTCTCTAACTGGGGTTCCATCGTTGACATCTACGCTCCAGGTTCCAAC   975
AGGTGATAGCTGCTGGTTTTCAGGAGAAAGAGATTGACCCCAAGGTAGCAACTGTAGATGCGAGGTCCAAGGTTG
   300            305            310            315            320
S  T  I  D  D  Q  K  S  S  F  S  N  W  G  S  I  V  D  I  Y  A  P  G  S  N
SP-PRO-NHSSP-His6

                                                                  AflIII AleI
ATTATTTCCGATGCTCCAGGTGGTGGTGTTAGAACTATGTCTGGTACTTCTATGGCTTCCCCACACGTTTGTGGT   1050
TAATAAAGGCTACGAGGTCCACCACCACAATCTTGATACAGACCATGAAGATACCGAAGGGGTGTGCAAACACCA
   325            330            335            340            345
I  I  S  D  A  P  G  G  G  V  R  T  M  S  G  T  S  M  A  S  P  H  V  C  G
SP-PRO-NHSSP-His6

GCTGGTGCTGCTATGTTGGCTCAAGGTGTTCCAGTTGGTCAGGTTTGTGACAGATTGAAGCAGATCGGTAACGCT   1125
CGACCACGACGATACAACCGAGTTCCACAAGGTCAACCAGTCCAAACACTGTCTAACTTCGTCTAGCCATTGCGA
   350            355            360            365            370
A  G  A  A  M  L  A  Q  G  V  P  V  G  Q  V  C  D  R  L  K  Q  I  G  N  A
SP-PRO-NHSSP-His6

GTTGTTAGAAACCCAGGTACTTCCACTACAAACAGATTGTTGTATAACGGTTCCGGTCAGGGTTCTCATCATCAC   1200
CAACAATCTTTGGGTCCATGAAGGTGATGTTTGTCTAACAACATATTGCCAAGGCCAGTCCCAAGAGTAGTAGTG
   375            380            385            390            395
V  V  R  N  P  G  T  S  T  T  N  R  L  L  Y  N  G  S  G  Q  G  S  H  H  H
SP-PRO-NHSSP-His6
                                                                  His6 tag
```

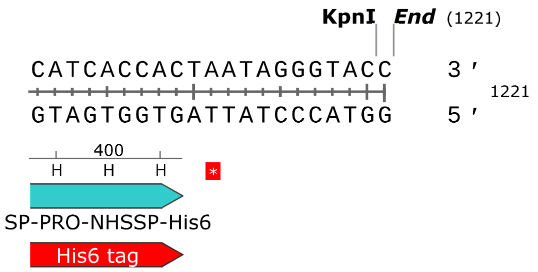
KpnI
End (1221)
CATCACCACTAATAGGGTACC 3′
1221
GTAGTGGTGATTATCCCATGG 5′
400
H H H
*
SP-PRO-NHSSP-His6
His6 tag
