# Supplementary material for: An alternative for proteinase K-heat-sensitive protease from fungus Onygena corvina for biotechnology: cloning, engineering, expression, characterization and special application for protein sequencing
Source: Microb Cell Fact. 2020 Jun 24;19:135. doi: 10.1186/s12934-020-01392-3 (PMC7313183; doi:10.1186/s12934-020-01392-3)
Supplement: Supplementary file 6 — Additional file 6. pPink-HC-NHSSP plasmid sequence and features. Secretion peptide native—marked in green, propeptide—marked in yellow, His6-tag—marked in red. [file 12934_2020_1392_MOESM6_ESM.pdf]

5′ AGATCTAACATCCAAAGACGAAAGGTTGAATGAAACCTTTTTGCCATCCGACATCCACAGGTCCATTCTCACACA 75
3′ TCTAGATTGTAGGTTTCTGCTTTCCAACTTACTTTGGAAAAACGGTAGGCTGTAGGTGTCCAGGTAAGAGTGTGT
AOX1 promoter

TAAGTGCCAAACGCAACAGGAGGGGATACACTAGCAGCAGACCGTTGCAAACGCAGGACCTCCACTCCTCTTCTC 150
ATTCACGGTTTGCGTTGTCCTCCCCTATGTGATCGTCGTCTGGCAACGTTTGCGTCCTGGAGGTGAGGAGAAGAG
AOX1 promoter

CTCAACACCCACTTTTGCCATCGAAAAACCAGCCCAGTTATTGGGCTTGATTGGAGCTCGCTCATTCCAATTCCT 225
GAGTTGTGGGTGAAAACGGTAGCTTTTTGGTCGGGTCAATAACCCGAACTAACCTCGAGCGAGTAAGGTTAAGGA
AOX1 promoter

TCTATTAGGCTACTAACACCATGACTTTATTAGCCTGTCTATCCTGGCCCCCCTGGCGAGGTTCATGTTTGTTTA 300
AGATAATCCGATGATTGTGGTACTGAAATAATCGGACAGATAGGACCGGGGGGACCGCTCCAAGTACAAACAAAT
AOX1 promoter

TTTCCGAATGCAACAAGCTCCGCATTACACCCGAACATCACTCCAGATGAGGGCTTTCTGAGTGTGGGGTCAAAT 375
AAAGGCTTACGTTGTTCGAGGCGTAATGTGGGCTTGTAGTGAGGTCTACTCCCGAAAGACTCACACCCCAGTTTA
AOX1 promoter

PmeI
AGTTTCATGTTCCCCAAATGGCCCAAAACTGACAGTTTAAACGCTGTCTTGGAACCTAATATGACAAAAGCGTGA 450
TCAAAGTACAAGGGGTTTACCGGGTTTTGACTGTCAAATTTGCGACAGAACCTTGGATTATACTGTTTTCGCACT
AOX1 promoter

TCTCATCCAAGATGAACTAAGTTTGGTTCGTTGAAATGCTAACGGCCAGTTGGTCAAAAAGAAACTTCCAAAAGT 525
AGAGTAGGTTCTACTTGATTCAAACCAAGCAACTTTACGATTGCCGGTCAACCAGTTTTTCTTTGAAGGTTTTCA
AOX1 promoter

CGGCATACCGTTTGTCTTGTTTGGTATTGATTGACGAATGCTCAAAAATAATCTCATTAATGCTTAGCGCAGTCT 600
GCCGTATGGCAAACAGAACAAACCATAACTAACTGCTTACGAGTTTTTATTAGAGTAATTACGAATCGCGTCAGA
AOX1 promoter

CTCTATCGCTTCTGAACCCCGGTGCACCTGTGCCGAAACGCAAATGGGGAAACACCCGCTTTTTGGATGATTATG 675
GAGATAGCGAAGACTTGGGGCCACGTGGACACGGCTTTGCGTTTACCCCTTTGTGGGCGAAAAACCTACTAATAC
AOX1 promoter

CATTGTCTCCACATTGTATGCTTCCAAGATTCTGGTGGGAATACTGCTGATAGCCTAACGTTCATGATCAAAATT 750
GTAACAGAGGTGTAACATACGAAGGTTCTAAGACCACCCTTATGACGACTATCGGATTGCAAGTACTAGTTTTAA
AOX1 promoter

TAACTGTTCTAACCCCTACTTGACAGCAATATATAAACAGAAGGAAGCTGCCCTGTCTTAAACCTTTTTTTTTAT 825
ATTGACAAGATTGGGGATGAACTGTCGTTATATATTTGTCTTCCTTCGACGGGACAGAATTTGGAAAAAAAAATA
AOX1 promoter

CATCATTATTAGCTTACTTTCATAATTGCGACTGGTTCCAATTGACAAGCTTTTGATTTTAACGACTTTTAACGA 900
GTAGTAATAATCGAATGAAAGTATTAACGCTGACCAAGGTTAACTGTTCGAAAACTAAAATTGCTGAAAATTGCT
AOX1 promoter

EcoRI

CAACTTGAGAAGATCAAAAAACAACTAATTATTCGAAACGGAATTCATGGGTTGTATCAAGGTTATCTCCGTTTT 975
GTTGAACTCTTCTAGTTTTTTGTTGATTAATAAGCTTTGCCTTAAGTACCCAACATAGTTCCAATAGAGGCAAAA

1 5 10
M G C I K V I S V F

AOX1 promoter | MCS | SP-PRO-NHSSP-His6

Secretion Peptide native

CTTGGCTGCTGTTGCTGCTGTTGACGCTAGAGCTTTTTTCCACAACAAGGGTGGTAACGACATCATCCCAAACTC 1050
GAACCGACGACAACGACGACAACTGCGATCTCGAAAAAAGGTGTTGTTCCCACCATTGCTGTAGTAGGGTTTGAG

15 20 25 30 35
L A A V A A V D A R A F F H N K G G N D I I P N S

SP-PRO-NHSSP-His6

Secretion Peptide native | Propeptide

CTACATCGTTGTTATGAAGGACGGTATCTCCACTCAAGAGTTCGAGTCCCACATTTCTTCCGTTTCTGCTACTCA 1125
GATGTAGCAACAATACTTCCTGCCATAGAGGTGAGTTCTCAAGCTCAGGGTGTAAAGAAGGCAAAGACGATGAGT

40 45 50 55 60
Y I V V M K D G I S T Q E F E S H I S S V S A T H

SP-PRO-NHSSP-His6

Propeptide

CGCTAAGAGATCCACTGAGTTGGTTGGTCACAAGGATTCCTTCAACATCAACGGTTGGAGAGCTTACAACGGTCA 1200
GCGATTCTCTAGGTGACTCAACCAACCAGTGTTCCTAAGGAAGTTGTAGTTGCCAACCTCTCGAATGTTGCCAGT

65 70 75 80 85
A K R S T E L V G H K D S F N I N G W R A Y N G H

SP-PRO-NHSSP-His6

Propeptide

CTTCGATGCTGCTACTTTGGAGTCCATTTTGAACGACGACAACGTTAAGTACGTTGAGCACGACAGAGTTGTTAA 1275
GAAGCTACGACGATGAAACCTCAGGTAAAACTTGCTGCTGTTGCAATTCATGCAACTCGTGCTGTCTCAACAATT

90 95 100 105 110
F D A A T L E S I L N D D N V K Y V E H D R V V K

SP-PRO-NHSSP-His6

Propeptide

GATCTCCGCTTTGACTACTCAGCCAAACGCTCCATCTTGGGGTTTGGGTAGAATTTCTCACAGATCCCCAGGTAA 1350
CTAGAGGCGAAACTGATGAGTCGGTTTGCGAGGTAGAACCCCAAACCCATCTTAAAGAGTGTCTAGGGGTCCATT

115 120 125 130 135
I S A L T T Q P N A P S W G L G R I S H R S P G N

SP-PRO-NHSSP-His6

Propeptide

CAAGGACTTCGTTTACGATGACACTGCTGGTCAGGGTATCACTATCTACGGTGTTGACACTGGAATCGACATCAA 1425
GTTCCTGAAGCAAATGCTACTGTGACGACCAGTCCCATAGTGATAGATGCCACAACTGTGACCTTAGCTGTAGTT

140 145 150 155 160
K D F V Y D D T A G Q G I T I Y G V D T G I D I N

SP-PRO-NHSSP-His6

Propeptide

CCACCCAGATTTTGGTGGTAGAGCTAGATGGGGAACTAACACTGTTGACAACGCTAACAACGACGGTCATGGTCA 1500
GGTGGGTCTAAAACCACCATCTCGATCTACCCCTTGATTGTGACAACTGTTGCGATTGTTGCTGCCAGTACCAGT

165 170 175 180 185
H P D F G G R A R W G T N T V D N A N N D G H G H

SP-PRO-NHSSP-His6

TGGTACTCATACTGCTGGTACTTTCGCTGGTAACGCTTACGGTATTGCTAAGAAGGCTTCCGTTGTTGCTGTTAA 1575
ACCATGAGTATGACGACCATGAAAGCGACCATTGCGAATGCCATAACGATTCTTCCGAAGGCAACAACGACAATT

190 195 200 205 210
G T H T A G T F A G N A Y G I A K K A S V V A V K

SP-PRO-NHSSP-His6

```
GGTTTTGTCTGCTTCTGGTTCCGGTTCCAACGCTGGTGTTATTAAGGGTATCGACTGGTGTGTTACAGACGCTAG   1650
CCAAAACAGACGAAGACCAAGGCCAAGGTTGCGACCACAATAATTCCCATAGCTGACCACACAATGTCTGCGATC
          215            220            225            230            235
 V  L  S  A  S  G  S  G  S  N  A  G  V  I  K  G  I  D  W  C  V  T  D  A  R
SP-PRO-NHSSP-His6

ATCTAAGGGTGCTTTGGGTAAGGCTGCTTTGAACTTGTCTTTGGGTGGTGGTTTCAACCAGGCTACAAACGACGC   1725
TAGATTCCCACGAAACCCATTCCGACGAAACTTGAACAGAAACCCACCACCAAAGTTGGTCCGATGTTTGCTGCG
          240            245            250            255            260
 S  K  G  A  L  G  K  A  A  L  N  L  S  L  G  G  G  F  N  Q  A  T  N  D  A
SP-PRO-NHSSP-His6

TGTTACTAGAGCTCAGACTGCTGGAATCTTCGTTGCTGTTGCTGCTGGTAACGATAACAAGGACGCTAGAAACTA   1800
ACAATGATCTCGAGTCTGACGACCTTAGAAGCAACGACAACGACGACCATTGCTATTGTTCCTGCGATCTTTGAT
          265            270            275            280            285
 V  T  R  A  Q  T  A  G  I  F  V  A  V  A  A  G  N  D  N  K  D  A  R  N  Y
SP-PRO-NHSSP-His6

CTCTCCAGCTTCTGCTCCAGCTGTTTGTACTGTTGCTTCCTCCACTATCGACGACCAAAAGTCCTCTTTCTCTAA   1875
GAGAGGTCGAAGACGAGGTCGACAAACATGACAACGAAGGAGGTGATAGCTGCTGGTTTTCAGGAGAAAGAGATT
          290            295            300            305            310
 S  P  A  S  A  P  A  V  C  T  V  A  S  S  T  I  D  D  Q  K  S  S  F  S  N
SP-PRO-NHSSP-His6

CTGGGGTTCCATCGTTGACATCTACGCTCCAGGTTCCAACATTATTTCCGATGCTCCAGGTGGTGGTGTTAGAAC   1950
GACCCCAAGGTAGCAACTGTAGATGCGAGGTCCAAGGTTGTAATAAAGGCTACGAGGTCCACCACCACAATCTTG
          315            320            325            330            335
 W  G  S  I  V  D  I  Y  A  P  G  S  N  I  I  S  D  A  P  G  G  G  V  R  T
SP-PRO-NHSSP-His6

TATGTCTGGTACTTCTATGGCTTCCCCACACGTTTGTGGTGCTGGTGCTGCTATGTTGGCTCAAGGTGTTCCAGT   2025
ATACAGACCATGAAGATACCGAAGGGGTGTGCAAACACCACGACCACGACGATACAACCGAGTTCCACAAGGTCA
          340            345            350            355            360
 M  S  G  T  S  M  A  S  P  H  V  C  G  A  G  A  A  M  L  A  Q  G  V  P  V
SP-PRO-NHSSP-His6

TGGTCAGGTTTGTGACAGATTGAAGCAGATCGGTAACGCTGTTGTTAGAAACCCAGGTACTTCCACTACAAACAG   2100
ACCAGTCCAAACACTGTCTAACTTCGTCTAGCCATTGCGACAACAATCTTTGGGTCCATGAAGGTGATGTTTGTC
          365            370            375            380            385
 G  Q  V  C  D  R  L  K  Q  I  G  N  A  V  V  R  N  P  G  T  S  T  T  N  R
SP-PRO-NHSSP-His6

                                                        NgoMIV
                                                                 NaeI
                                                   KpnI
                                                Acc65I        FseI      SwaI
ATTGTTGTATAACGGTTCCGGTCAGGGTTCTCATCATCACCATCACCACTAATAGGGTACCGGCCGGCCATTTAA   2175
TAACAACATATTGCCAAGGCCAGTCCCAAGAGTAGTAGTGGTAGTGGTGATTATCCCATGGCCGGCCGGTAAATT
          390            395            400
 L  L  Y  N  G  S  G  Q  G  S  H  H  H  H  H  H     *
SP-PRO-NHSSP-His6                                            MCS
                              His6 tag

                         EcoRV
ATACAGGCCCCTTTTCCTTTGTCGATATCATGTAATTAGTTATGTCACGCTTACATTCACGCCCTCCTCCCACAT   2250
TATGTCCGGGGAAAAGGAAACAGCTATAGTACATTAATCAATACAGTGCGAATGTAAGTGCGGGAGGAGGGTGTA
MCS                                         CYC1 terminator

CCGCTCTAACCGAAAAGGAAGGAGTTAGACAACCTGAAGTCTAGGTCCCTATTTATTTTTTTTAATAGTTATGTT   2325
GGCGAGATTGGCTTTTCCTTCCTCAATCTGTTGGACTTCAGATCCAGGGATAAATAAAAAAAATTATCAATACAA
CYC1 terminator
```

```
                                             BsrGI        MluI
AGTATTAAGAACGTTATTTATATTTCAAATTTTTCTTTTTTTTCTGTACAAACGCGTGTACGCATGTAACATTAT   2400
TCATAATTCTTGCAATAAATATAAAGTTTAAAAAGAAAAAAAAGACATGTTTGCGCACATGCGTACATTGTAATA
CYC1 terminator

                                                           NotI
                                                   BamHI   SacII
ACTGAAAACCTTGCTTGAGAAGGTTTTGGGACGCTCGAAGGCTTTAATTTGCAAGCTGGATCCGCGGCCGCCTTC   2475
TGACTTTTGGAACGAACTCTTCCAAAACCCTGCGAGCTTCCGAAATTAAACGTTCGACCTAGGCGCCGGCGGAAG
CYC1 terminator                                                     PpADE2 HC promoter

                                                                         NheI
CAAACTCTCATGGATTCTCAGGTAATAGGTATTCTAGGAGGAGGCCAGCTAGGCCGAATGATTGTTGAGGCCGCT   2550
GTTTGAGAGTACCTAAGAGTCCATTATCCATAAGATCCTCCTCCGGTCGATCCGGCTTACTAACAACTCCGGCGA
1 5 10 15 20
M D S Q V I G I L G G G Q L G R M I V E A A
PpADE2 HC promoter          PpADE2

 BmtI
AGCAGGCTCAATATCAAGACCGTGATTCTTGATGATGGTTTTTCACCTGCTAAGCACATTAATGCTGCGCAAGAC   2625
TCGTCCGAGTTATAGTTCTGGCACTAAGAACTACTACCAAAAAGTGGACGATTCGTGTAATTACGACGCGTTCTG
25 30 35 40 45
S R L N I K T V I L D D G F S P A K H I N A A Q D
PpADE2

CACATCGACGGATCATTCAAAGATGAGGAGGCTATCGCCAAGTTAGCTGCCAAATGTGATGTTCTCACTGTAGAG   2700
GTGTAGCTGCCTAGTAAGTTTCTACTCCTCCGATAGCGGTTCAATCGACGGTTTACACTACAAGAGTGACATCTC
50 55 60 65 70
H I D G S F K D E E A I A K L A A K C D V L T V E
PpADE2

ATTGAGCATGTCAACACAGATGCTCTAAAGAGAGTTCAAGACAGAACTGGAATCAAGATATATCCTTTACCAGAG   2775
TAACTCGTACAGTTGTGTCTACGAGATTTCTCTCAAGTTCTGTCTTGACCTTAGTTCTATATAGGAAATGGTCTC
75 80 85 90 95
I E H V N T D A L K R V Q D R T G I K I Y P L P E
PpADE2

ACAATCGAACTAATCAAGGATAAGTACTTGCAAAAGGAACATTTGATCAAGCACAACATTTCGGTGACAAAGTCT   2850
TGTTAGCTTGATTAGTTCCTATTCATGAACGTTTTCCTTGTAAACTAGTTCGTGTTGTAAAGCCACTGTTTCAGA
100 105 110 115 120
T I E L I K D K Y L Q K E H L I K H N I S V T K S
PpADE2

CAGGGTATAGAATCTAATGAAAAGGCGCTGCTTTTGTTTGGAGAAGAGAATGGATTTCCATATCTGTTGAAGTCC   2925
GTCCCATATCTTAGATTACTTTTCCGCGACGAAAACAAACCTCTTCTCTTACCTAAAGGTATAGACAACTTCAGG
125 130 135 140 145
Q G I E S N E K A L L L F G E E N G F P Y L L K S
PpADE2

CGGACTATGGCTTATGATGGAAGAGGCAATTTTGTAGTGGAGTCTAAAGAGGACATCAGTAAGGCATTAGAGTTC   3000
GCCTGATACCGAATACTACCTTCTCCGTTAAAACATCACCTCAGATTTCTCCTGTAGTCATTCCGTAATCTCAAG
150 155 160 165 170
R T M A Y D G R G N F V V E S K E D I S K A L E F
PpADE2
```

TTGAAAGATCGTCCATTGTATGCCGAGAAGTTTGCTCCTTTTGTTAAAGAATTAGCGGTAATGGTTGTGAGATCA 3075
AACTTTCTAGCAGGTAACATACGGCTCTTCAAACGAGGAAAACAATTTCTTAATCGCCATTACCAACACTCTAGT
175 180 185 190 195
L K D R P L Y A E K F A P F V K E L A V M V V R S
PpADE2

CTGGAAGGCGAAGTATTCTCCTACCCAACCGTAGAAACTGTGCACAAGGACAATATCTGTCATATTGTGTATGCT 3150
GACCTTCCGCTTCATAAGAGGATGGGTTGGCATCTTTGACACGTGTTCCTGTTATAGACAGTATAACACATACGA
200 205 210 215 220
L E G E V F S Y P T V E T V H K D N I C H I V Y A
PpADE2

BbsI

CCGGCCAGAGTTAATGACACCATCCAAAAGAAAGCTCAAATATTAGCTGAAAACACTGTGAAGACTTTCCCAGGC 3225
GGCCGGTCTCAATTACTGTGGTAGGTTTTCTTTCGAGTTTATAATCGACTTTTGTGACACTTCTGAAAGGGTCCG
225 230 235 240 245
P A R V N D T I Q K K A Q I L A E N T V K T F P G
PpADE2

GCTGGAATCTTCGGAGTTGAGATGTTCCTATTGTCTGATGGAGAACTTCTTGTAAATGAGATTGCTCCAAGGCCC 3300
CGACCTTAGAAGCCTCAACTCTACAAGGATAACAGACTACCTCTTGAAGAACATTTACTCTAACGAGGTTCCGGG
250 255 260 265 270
A G I F G V E M F L L S D G E L L V N E I A P R P
PpADE2

CACAATTCTGGTCACTATACAATCGATGCATGTGTAACATCTCAGTTCGAAGCACATGTAAGAGCCATAACTGGT 3375
GTGTTAAGACCAGTGATATGTTAGCTACGTACACATTGTAGAGTCAAGCTTCGTGTACATTCTCGGTATTGACCA
275 280 285 290 295
H N S G H Y T I D A C V T S Q F E A H V R A I T G
PpADE2

CTGCCAATGCCACTAGATTTCACCAAACTATCTACTTCCAACACCAACGCTATTATGCTCAATGTTTTGGGTGCT 3450
GACGGTTACGGTGATCTAAAGTGGTTTGATAGATGAAGGTTGTGGTTGCGATAATACGAGTTACAAAACCCACGA
300 305 310 315 320
L P M P L D F T K L S T S N T N A I M L N V L G A
PpADE2

DraIII

GAAAAATCTCACGGGGAATTAGAGTTTTGTAGAAGAGCCTTAGAAACACCCGGTGCTTCTGTATATCTGTACGGA 3525
CTTTTTAGAGTGCCCCTTAATCTCAAAACATCTTCTCGGAATCTTTGTGGGCCACGAAGACATATAGACATGCCT
325 330 335 340 345
E K S H G E L E F C R R A L E T P G A S V Y L Y G
PpADE2

AAGACCACCCGATTGGCTCGTAAGATGGGTCATATCAACATAATAGGATCTTCCATGTTGGAAGCAGAACAAAAG 3600
TTCTGGTGGGCTAACCGAGCATTCTACCCAGTATAGTTGTATTATCCTAGAAGGTACAACCTTCGTCTTGTTTTC
350 355 360 365 370
K T T R L A R K M G H I N I I G S S M L E A E Q K
PpADE2

TTAGAGTACATTCTAGAAGAATCAACCCACTTACCATCCAGTACTGTATCAGCTGACACTAAACCGTTGGTTGGA 3675
AATCTCATGTAAGATCTTCTTAGTTGGGTGAATGGTAGGTCATGACATAGTCGACTGTGATTTGGCAACCAACCT
375 380 385 390 395
L E Y I L E E S T H L P S S T V S A D T K P L V G
PpADE2

GTTATCATGGGTTCAGACTCTGATCTACCTGTGATTTCGAAAGGTTGCGATATTTTAAAACAGTTTGGTGTTCCA 3750
CAATAGTACCCAAGTCTGAGACTAGATGGACACTAAAGCTTTCCAACGCTATAAAATTTTGTCAAACCACAAGGT
400 405 410 415 420
V I M G S D S D L P V I S K G C D I L K Q F G V P
PpADE2

TTCGAAGTTACTATTGTCTCTGCTCATAGAACACCACAGAGAATGACCAGATATGCCTTTGAAGCCGCTAGTAGA 3825
AAGCTTCAATGATAACAGAGACGAGTATCTTGTGGTGTCTCTTACTGGTCTATACGGAAACTTCGGCGATCATCT
425 430 435 440 445
F E V T I V S A H R T P Q R M T R Y A F E A A S R
PpADE2

GGTATCAAGGCTATCATTGCAGGTGCTGGTGGTGCTGCTCATCTTCCAGGAATGGTTGCTGCCATGACTCCGTTG 3900
CCATAGTTCCGATAGTAACGTCCACGACCACCACGACGAGTAGAAGGTCCTTACCAACGACGGTACTGAGGCAAC
450 455 460 465 470
G I K A I I A G A G G A A H L P G M V A A M T P L
PpADE2

CCAGTCATTGGTGTTCCTGTCAAGGGCTCTACGTTGGATGGTGTAGACTCGCTACACTCGATTGTCCAAATGCCT 3975
GGTCAGTAACCACAAGGACAGTTCCCGAGATGCAACCTACCACATCTGAGCGATGTGAGCTAACAGGTTTACGGA
475 480 485 490 495
P V I G V P V K G S T L D G V D S L H S I V Q M P
PpADE2

AGAGGTGTTCCTGTGGCTACGGTTGCTATCAACAACGCCACCAATGCCGCTCTGTTGGCCATCAGGATTTTAGGT 4050
TCTCCACAAGGACACCGATGCCAACGATAGTTGTTGCGGTGGTTACGGCGAGACAACCGGTAGTCCTAAAATCCA
500 505 510 515 520
R G V P V A T V A I N N A T N A A L L A I R I L G
PpADE2

ACAATTGACCACAAATGGCAAAAGGAAATGTCCAAGTATATGAATGCAATGGAGACCGAAGTGTTGGGGAAGGCA 4125
TGTTAACTGGTGTTTACCGTTTTCCTTTACAGGTTCATATACTTACGTTACCTCTGGCTTCACAACCCCTTCCGT
525 530 535 540 545
T I D H K W Q K E M S K Y M N A M E T E V L G K A
PpADE2

TCCAACTTGGAATCTGAAGGGTATGAATCCTATTTGAAGAATCGTCTTTGAATTTAGTATTGTTTTTTAATAGAT 4200
AGGTTGAACCTTAGACTTCCCATACTTAGGATAAACTTCTTAGCAGAAACTTAAATCATAACAAAAAATTATCTA
550 555 560
S N L E S E G Y E S Y L K N R L *
PpADE2

BsaAI
GTATATATAATAGTACACGTAACTTATCTATTCCATTCATAATTTTATTTTAAAGGTTCGGTAGAAATTTGTCCT 4275
CATATATATTATCATGTGCATTGAATAGATAAGGTAAGTATTAAAATAAAATTTCCAAGCCATCTTTAAACAGGA

BsgI
CCAAAAAGTTGGTTAGAGCCTGGCAGTTTTGATAGGCATTATTATAGATTGGGTAATATTTACCCTGCACCTGGA 4350
GGTTTTTCAACCAATCTCGGACCGTCAAAACTATCCGTAATAATATCTAACCCATTATAAATGGGACGTGGACCT

AscI
BssHII
GGAACTTTGCAAAGAGCCTCATGTGCGGCGCGCCAGGCCATAATGGCCAAACGGTTTCTCAATTACTATATACTA 4425
CCTTGAAACGTTTCTCGGAGTACACGCCGCGCGGTCCGGTATTACCGGTTTGCCAAAGAGTTAATGATATATGAT
PpTRP2 promoter

NruI
CTAACCATTTACCTGTAGCGTATTTCTTTTCCCTCTTCGCGAAAGCTCAAGGGCATCTTCTTGACTCATGAAAAA 4500
GATTGGTAAATGGACATCGCATAAAGAAAAGGGAGAAGCGCTTTCGAGTTCCCGTAGAAGAACTGAGTACTTTTT
PpTRP2 promoter

BsaBI

TATCTGGATTTCTTCTGACAGATCATCACCCTTGAGCCCAACTCTCTAGCCTATGAGTGTAAGTGATAGTCATCT 4575
ATAGACCTAAAGAAGACTGTCTAGTAGTGGGAACTCGGGTTGAGAGATCGGATACTCACATTCACTATCAGTAGA

1 5
M S V S D S H L

PpTRP2 promoter
PpTRP2

TGCAACAGATTATTTTGGAACGCAACTAACAAAGCAGATACACCCTTCAGCAGAATCCTTTCTGGATATTGTGAA 4650
ACGTTGTCTAATAAAACCTTGCGTTGATTGTTTCGTCTATGTGGGAAGTCGTCTTAGGAAAGACCTATAACACTT

10 15 20 25 30
A T D Y F G T Q L T K Q I H P S A E S F L D I V K

PpTRP2

GAATGATCGCCAAAGTCACAGTCCTGAGACAGTTCCTAATCTTTACCCCATTTACAAGTTCATCCAATCAGACTT 4725
CTTACTAGCGGTTTCAGTGTCAGGACTCTGTCAAGGATTAGAAATGGGGTAAATGTTCAAGTAGGTTAGTCTGAA

35 40 45 50 55
N D R Q S H S P E T V P N L Y P I Y K F I Q S D F

PpTRP2

CTTAACGCCTCATCTGGCTTATATCAAGCTTACCAACAGTTCAGAAACTCCCAGTCCAAGTTTCTTGCTTGAAAG 4800
GAATTGCGGAGTAGACCGAATATAGTTCGAATGGTTGTCAAGTCTTTGAGGGTCAGGTTCAAAGAACGAACTTTC

60 65 70 75 80
L T P H L A Y I K L T N S S E T P S P S F L L E S

PpTRP2

TGCGAAGAATGGTGACACCGTTGACAGGTACACCTTTATGGGACATTCCCCCAGAAAAATAATCAAGACTGGGCC 4875
ACGCTTCTTACCACTGTGGCAACTGTCCATGTGGAAATACCCTGTAAGGGGGTCTTTTTATTAGTTCTGACCCGG

85 90 95 100 105
A K N G D T V D R Y T F M G H S P R K I I K T G P

PpTRP2

TTTAGAGGGTGCTGAAGTTGACCCCTTGGTGCTTCTGGAAAAAGAACTGAAGGGCACCAGACAAGCGCAACTTCC 4950
AAATCTCCCACGACTTCAACTGGGGAACCACGAAGACCTTTTTCTTGACTTCCCGTGGTCTGTTCGCGTTGAAGG

110 115 120 125 130
L E G A E V D P L V L L E K E L K G T R Q A Q L P

PpTRP2

TGGTATTCCTCGTCTAAGTGGTGGTGCCATAGGATACATCTCGTACGATTGTATTAAGTACTTTGAACCAAAAAC 5025
ACCATAAGGAGCAGATTCACCACCACGGTATCCTATGTAGAGCATGCTAACATAATTCATGAAACTTGGTTTTTG

135 140 145 150 155
G I P R L S G G A I G Y I S Y D C I K Y F E P K T

PpTRP2

BspEI

TGAAAGAAAACTGAAAGATGTTTTGCAACTTCCGGAAGCAGCTTTGATGTTGTTCGACACGATCGTGGCTTTTGA 5100
ACTTTCTTTTGACTTTCTACAAAACGTTGAAGGCCTTCGTCGAAACTACAACAAGCTGTGCTAGCACCGAAAACT

160 165 170 175 180
E R K L K D V L Q L P E A A L M L F D T I V A F D

PpTRP2

CAATGTTTATCAAAGATTCCAGGTAATTGGAAACGTTTCTCTATCCGTTGATGACTCGGACGAAGCTATTCTTGA 5175
GTTACAAATAGTTTCTAAGGTCCATTAACCTTTGCAAAGAGATAGGCAACTACTGAGCCTGCTTCGATAAGAACT

185 190 195 200 205
N V Y Q R F Q V I G N V S L S V D D S D E A I L E

PpTRP2

PsiI

GAAATATTATAAGACAAGAGAAGAAGTGGAAAAGATCAGTAAAGTGGTATTTGACAATAAAACTGTTCCCTACTA 5250
CTTTATAATATTCTGTTCTCTTCTTCACCTTTTCTAGTCATTTCACCATAAACTGTTATTTTGACAAGGGATGAT

210 215 220 225 230
K Y Y K T R E E V E K I S K V V F D N K T V P Y Y

PpTRP2

```
TGAACAGAAAGATATTATTCAAGGCCAAACGTTCACCTCTAATATTGGTCAGGAAGGGTATGAAAACCATGTTCG   5325
ACTTGTCTTTCTATAATAAGTTCCGGTTTGCAAGTGGAGATTATAACCAGTCCTTCCCATACTTTTGGTACAAGC
  235  240  245  250  255
 E Q K D I I Q G Q T F T S N I G Q E G Y E N H V R
PpTRP2
```

```
                                      EcoNI
CAAGCTGAAAGAACATATTCTGAAAGGAGACATCTTCCAAGCTGTTCCCTCTCAAAGGGTAGCCAGGCCGACCTC   5400
GTTCGACTTTCTTGTATAAGACTTTCCTCTGTAGAAGGTTCGACAAGGGAGAGTTTCCCATCGGTCCGGCTGGAG
  260  265  270  275  280
 K L K E H I L K G D I F Q A V P S Q R V A R P T S
PpTRP2
```

```
ATTGCACCCTTTCAACATCTATCGTCATTTGAGAACTGTCAATCCTTCTCCATACATGTTCTATATTGACTATCT   5475
TAACGTGGGAAAGTTGTAGATAGCAGTAAACTCTTGACAGTTAGGAAGAGGTATGTACAAGATATAACTGATAGA
  285  290  295  300  305
 L H P F N I Y R H L R T V N P S P Y M F Y I D Y L
PpTRP2
```

```
                           SpeI
AGACTTCCAAGTTGTTGGTGCTTCACCTGAATTACTAGTTAAATCCGACAACAACAACAAAATCATCACACATCC   5550
TCTGAAGGTTCAACAACCACGAAGTGGACTTAATGATCAATTTAGGCTGTTGTTGTTGTTTTAGTAGTGTGTAGG
  310  315  320  325  330
 D F Q V V G A S P E L L V K S D N N N K I I T H P
PpTRP2
```

```
TATTGCTGGAACTCTTCCCAGAGGTAAAACTATCGAAGAGGACGACAATTATGCTAAGCAATTGAAGTCGTCTTT   5625
ATAACGACCTTGAGAAGGGTCTCCATTTTGATAGCTTCTCCTGCTGTTAATACGATTCGTTAACTTCAGCAGAAA
  335  340  345  350  355
 I A G T L P R G K T I E E D D N Y A K Q L K S S L
PpTRP2
```

```
               BmgBI
GAAAGACAGGGCCGAGCACGTCATGCTGGTAGATTTGGCCAGAAATGATATTAACCGTGTGTGTGAGCCCACCAG   5700
CTTTCTGTCCCGGCTCGTGCAGTACGACCATCTAAACCGGTCTTTACTATAATTGGCACACACACTCGGGTGGTC
  360  365  370  375  380
 K D R A E H V M L V D L A R N D I N R V C E P T S
PpTRP2
```

```
TACCACGGTTGATCGTTTATTGACTGTGGAGAGATTTTCTCATGTGATGCATCTTGTGTCAGAAGTCAGTGGAAC   5775
ATGGTGCCAACTAGCAAATAACTGACACCTCTCTAAAAGAGTACACTACGTAGAACACAGTCTTCAGTCACCTTG
  385  390  395  400  405
 T T V D R L L T V E R F S H V M H L V S E V S G T
PpTRP2
```

```
ATTGAGACCAAACAAGACTCGCTTCGATGCTTTCAGATCCATTTTCCCAGCAGGAACCGTCTCCGGTGCTCCGAA   5850
TAACTCTGGTTTGTTCTGAGCGAAGCTACGAAAGTCTAGGTAAAAGGGTCGTCCTTGGCAGAGGCCACGAGGCTT
  410  415  420  425  430
 L R P N K T R F D A F R S I F P A G T V S G A P K
PpTRP2
```

```
                                                                         PshAI
GGTAAGAGCAATGCAACTCATAGGAGAATTGGAAGGAGAAAAGAGAGGTGTTTATGCGGGGGCCGTAGGACACTG   5925
CCATTCTCGTTACGTTGAGTATCCTCTTAACCTTCCTCTTTTCTCTCCACAAATACGCCCCCGGCATCCTGTGAC
  435  440  445  450  455
 V R A M Q L I G E L E G E K R G V Y A G A V G H W
PpTRP2
```

AflII

GTCGTACGATGGAAAATCGATGGACACATGTATTGCCTTAAGAACAATGGTCGTCAAGGACGGTGTCGCTTACCT 6000
CAGCATGCTACCTTTTAGCTACCTGTGTACATAACGGAATTCTTGTTACCAGCAGTTCCTGCCACAGCGAATGGA

460 465 470 475 480
S Y D G K S M D T C I A L R T M V V K D G V A Y L
PpTRP2

TCAAGCCGGAGGTGGAATTGTCTACGATTCTGACCCCTATGACGAGTACATCGAAACCATGAACAAAATGAGATC 6075
AGTTCGGCCTCCACCTTAACAGATGCTAAGACTGGGGATACTGCTCATGTAGCTTTGGTACTTGTTTTACTCTAG

485 490 495 500 505
Q A G G G I V Y D S D P Y D E Y I E T M N K M R S
PpTRP2

CAACAATAACACCATCTTGGAGGCTGAGAAAATCTGGACCGATAGGTTGGCCAGAGACGAGAATCAAAGTGAATC 6150
GTTGTTATTGTGGTAGAACCTCCGACTCTTTTAGACCTGGCTATCCAACCGGTCTCTGCTCTTAGTTTCACTTAG

510 515 520 525 530
N N N T I L E A E K I W T D R L A R D E N Q S E S
PpTRP2

CGAAGAAAACGATCAATGAACGGAGGACGTAAGTAGGAATTTATGGTTTGGCCATAATGGCCTAGCTTGGCGTAA 6225
GCTTCTTTTGCTAGTTACTTGCCTCCTGCATTCATCCTTAAATACCAAACCGGTATTACCGGATCGAACCGCATT

535
E E N D Q *
PpTRP2

TCATGGTCATAGCTGTTTCCTGTGTGAAATTGTTATCCGCTCACAATTCCACACAACATACGAGCCGGAAGCATA 6300
AGTACCAGTATCGACAAAGGACACACTTTAACAATAGGCGAGTGTTAAGGTGTGTTGTATGCTCGGCCTTCGTAT

AAGTGTAAAGCCTGGGGTGCCTAATGAGTGAGCTAACTCACATTAATTGCGTTGCGCTCACTGCCCGCTTTCCAG 6375
TTCACATTTCGGACCCCACGGATTACTCACTCGATTGAGTGTAATTAACGCAACGCGAGTGACGGGCGAAAGGTC

TCGGGAAACCTGTCGTGCCAGCTGCATTAATGAATCGGCCAACGCGCGGGGAGAGGCGGTTTGCGTATTGGGCGC 6450
AGCCCTTTGGACAGCACGGTCGACGTAATTACTTAGCCGGTTGCGCGCCCCTCTCCGCCAAACGCATAACCCGCG

TCTTCCGCTTCCTCGCTCACTGACTCGCTGCGCTCGGTCGTTCGGCTGCGGCGAGCGGTATCAGCTCACTCAAAG 6525
AGAAGGCGAAGGAGCGAGTGACTGAGCGACGCGAGCCAGCAAGCCGACGCCGCTCGCCATAGTCGAGTGAGTTTC

GCGGTAATACGGTTATCCACAGAATCAGGGGATAACGCAGGAAAGAACATGTGAGCAAAAGGCCAGCAAAAGGCC 6600
CGCCATTATGCCAATAGGTGTCTTAGTCCCCTATTGCGTCCTTTCTTGTACACTCGTTTTCCGGTCGTTTTCCGG

AGGAACCGTAAAAAGGCCGCGTTGCTGGCGTTTTTCCATAGGCTCCGCCCCCCTGACGAGCATCACAAAAATCGA 6675
TCCTTGGCATTTTTCCGGCGCAACGACCGCAAAAAGGTATCCGAGGCGGGGGGACTGCTCGTAGTGTTTTTAGCT
ori

CGCTCAAGTCAGAGGTGGCGAAACCCGACAGGACTATAAAGATACCAGGCGTTTCCCCCTGGAAGCTCCCTCGTG 6750
GCGAGTTCAGTCTCCACCGCTTTGGGCTGTCCTGATATTTCTATGGTCCGCAAAGGGGGACCTTCGAGGGAGCAC
ori

CGCTCTCCTGTTCCGACCCTGCCGCTTACCGGATACCTGTCCGCCTTTCTCCCTTCGGGAAGCGTGGCGCTTTCT 6825
GCGAGAGGACAAGGCTGGGACGGCGAATGGCCTATGGACAGGCGGAAAGAGGGAAGCCCTTCGCACCGCGAAAGA
ori

CATAGCTCACGCTGTAGGTATCTCAGTTCGGTGTAGGTCGTTCGCTCCAAGCTGGGCTGTGTGCACGAACCCCCC 6900
GTATCGAGTGCGACATCCATAGAGTCAAGCCACATCCAGCAAGCGAGGTTCGACCCGACACACGTGCTTGGGGGG
ori

GTTCAGCCCGACCGCTGCGCCTTATCCGGTAACTATCGTCTTGAGTCCAACCCGGTAAGACACGACTTATCGCCA 6975
CAAGTCGGGCTGGCGACGCGGAATAGGCCATTGATAGCAGAACTCAGGTTGGGCCATTCTGTGCTGAATAGCGGT
< ori

CTGGCAGCAGCCACTGGTAACAGGATTAGCAGAGCGAGGTATGTAGGCGGTGCTACAGAGTTCTTGAAGTGGTGG 7050
GACCGTCGTCGGTGACCATTGTCCTAATCGTCTCGCTCCATACATCCGCCACGATGTCTCAAGAACTTCACCACC
< ori

CCTAACTACGGCTACACTAGAAGGACAGTATTTGGTATCTGCGCTCTGCTGAAGCCAGTTACCTTCGGAAAAAGA 7125
GGATTGATGCCGATGTGATCTTCCTGTCATAAACCATAGACGCGAGACGACTTCGGTCAATGGAAGCCTTTTTCT
< ori

GTTGGTAGCTCTTGATCCGGCAAACAAACCACCGCTGGTAGCGGTGGTTTTTTTGTTTGCAAGCAGCAGATTACG 7200
CAACCATCGAGAACTAGGCCGTTTGTTTGGTGGCGACCATCGCCACCAAAAAAACAAACGTTCGTCGTCTAATGC
< ori

CGCAGAAAAAAAGGATCTCAAGAAGATCCTTTGATCTTTTCTACGGGGTCTGACGCTCAGTGGAACGAAAACTCA 7275
GCGTCTTTTTTTCCTAGAGTTCTTCTAGGAAACTAGAAAAGATGCCCCAGACTGCGAGTCACCTTGCTTTTGAGT
< ori

CGTTAAGGGATTTTGGTCATGAGATTATCAAAAAGGATCTTCACCTAGATCCTTTTAAATTAAAAATGAAGTTTT 7350
GCAATTCCCTAAAACCAGTACTCTAATAGTTTTTCCTAGAAGTGGATCTAGGAAAATTTAATTTTTACTTCAAAA

AAATCAATCTAAAGTATATATGAGTAAACTTGGTCTGACAGTTACCAATGCTTAATCAGTGAGGCACCTATCTCA 7425
TTTAGTTAGATTTCATATATACTCATTTGAACCAGACTGTCAATGGTTACGAATTAGTCACTCCGTGGATAGAGT
285 280
* W H K I L S A G I E
AmpR

GCGATCTGTCTATTTCGTTCATCCATAGTTGCCTGACTCCCCGTCGTGTAGATAACTACGATACGGGAGGGCTTA 7500
CGCTAGACAGATAAAGCAAGTAGGTATCAACGGACTGAGGGGCAGCACATCTATTGATGCTATGCCCTCCCGAAT
275 270 265 260 255
A I Q R N R E D M T A Q S G T T Y I V V I R S P K
< AmpR

CCATCTGGCCCCAGTGCTGCAATGATACCGCGAGACCCACGCTCACCGGCTCCAGATTTATCAGCAATAAACCAG 7575
GGTAGACCGGGGTCACGACGTTACTATGGCGCTCTGGGTGCGAGTGGCCGAGGTCTAAATAGTCGTTATTTGGTC
250 245 240 235 230
G D P G L A A I I G R S G R E G A G S K D A I F W
< AmpR

CCAGCCGGAAGGGCCGAGCGCAGAAGTGGTCCTGCAACTTTATCCGCCTCCATCCAGTCTATTAATTGTTGCCGG 7650
GGTCGGCCTTCCCGGCTCGCGTCTTCACCAGGACGTTGAAATAGGCGGAGGTAGGTCAGATAATTAACAACGGCC
225 220 215 210 205
G A P L A S R L L P G A V K D A E M W D I L Q Q R
< AmpR

GAAGCTAGAGTAAGTAGTTCGCCAGTTAATAGTTTGCGCAACGTTGTTGCCATTGCTACAGGCATCGTGGTGTCA 7725
CTTCGATCTCATTCATCAAGCGGTCAATTATCAAACGCGTTGCAACAACGGTAACGATGTCCGTAGCACCACAGT
200 195 190 185 180
S A L T L L E G T L L K R L T T A M A V P M T T D
< AmpR

CGCTCGTCGTTTGGTATGGCTTCATTCAGCTCCGGTTCCCAACGATCAAGGCGAGTTACATGATCCCCCATGTTG 7800
GCGAGCAGCAAACCATACCGAAGTAAGTCGAGGCCAAGGGTTGCTAGTTCCGCTCAATGTACTAGGGGGTACAAC

175 170 165 160 155
R E D N P I A E N L E P E W R D L R T V H D G M N

AmpR

TGCAAAAAAGCGGTTAGCTCCTTCGGTCCTCCGATCGTTGTCAGAAGTAAGTTGGCCGCAGTGTTATCACTCATG 7875
ACGTTTTTTCGCCAATCGAGGAAGCCAGGAGGCTAGCAACAGTCTTCATTCAACCGGCGTCACAATAGTGAGTAC

150 145 140 135 130
H L F A T L E K P G G I T T L L L N A A T N D S M

AmpR

GTTATGGCAGCACTGCATAATTCTCTTACTGTCATGCCATCCGTAAGATGCTTTTCTGTGACTGGTGAGTACTCA 7950
CAATACCGTCGTGACGTATTAAGAGAATGACAGTACGGTAGGCATTCTACGAAAAGACACTGACCACTCATGAGT

125 120 115 110 105
T I A A S C L E R V T M G D T L H K E T V P S Y E

AmpR

ACCAAGTCATTCTGAGAATAGTGTATGCGGCGACCGAGTTGCTCTTGCCCGGCGTCAATACGGGATAATACCGCG 8025
TGGTTCAGTAAGACTCTTATCACATACGCCGCTGGCTCAACGAGAACGGGCCGCAGTTATGCCCTATTATGGCGC

100 95 90 85 80
V L D N Q S Y H I R R G L Q E Q G A D I R S L V A

AmpR

CCACATAGCAGAACTTTAAAAGTGCTCATCATTGGAAAACGTTCTTCGGGGCGAAAACTCTCAAGGATCTTACCG 8100
GGTGTATCGTCTTGAAATTTTCACGAGTAGTAACCTTTTGCAAGAAGCCCCGCTTTTGAGAGTTCCTAGAATGGC

75 70 65 60 55
G C L L V K F T S M M P F R E E P R F S E L I K G

AmpR

CTGTTGAGATCCAGTTCGATGTAACCCACTCGTGCACCCAACTGATCTTCAGCATCTTTTACTTTCACCAGCGTT 8175
GACAACTCTAGGTCAAGCTACATTGGGTGAGCACGTGGGTTGACTAGAAGTCGTAGAAAATGAAAGTGGTCGCAA

50 45 40 35 30
S N L D L E I Y G V R A G L Q D E A D K V K V L T

AmpR

TCTGGGTGAGCAAAAACAGGAAGGCAAAATGCCGCAAAAAAGGGAATAAGGGCGACACGGAAATGTTGAATACTC 8250
AGACCCACTCGTTTTTGTCCTTCCGTTTTACGGCGTTTTTTCCCTTATTCCCGCTGTGCCTTTACAACTTATGAG

25 20 15 10 5
E P H A F V P L C F A A F F P I L A V R F H Q I S

signal sequence

AmpR

ATACTCTTCCTTTTTCAATATTATTGAAGCATTTATCAGGGTTATTGTCTCATGAGCGGATACATATTTGAATGT 8325
TATGAGAAGGAAAAAGTTATAATAACTTCGTAAATAGTCCCAATAACAGAGTACTCGCCTATGTATAAACTTACA

1
M

AmpR promoter

AmpR

ZraI **AatII**

ATTTAGAAAAATAAACAAATAGGGGTTCCGCGCACATTTCCCCGAAAAGTGCCACCTGACGTCTAAGAAACCATT 8400
TAAATCTTTTTATTTGTTTATCCCCAAGGCGCGTGTAAAGGGGCTTTTCACGGTGGACTGCAGATTCTTTGGTAA

AmpR promoter

ATTATCATGACATTAACCTATAAAAATAGGCGTATCACGAGGCCCTTTCGTCTCGCGCGTTTCGGTGATGACGGT 8475
TAATAGTACTGTAATTGGATATTTTTATCCGCATAGTGCTCCGGGAAAGCAGAGCGCGCAAAGCCACTACTGCCA

```
GAAAACCTCTGACACATGCAGCTCCCGGAGACGGTCACAGCTTGTCTGTAAGCGGATGCCGGGAGCAGACAAGCC
                                                                            8550
CTTTTGGAGACTGTGTACGTCGAGGGCCTCTGCCAGTGTCGAACAGACATTCGCCTACGGCCCTCGTCTGTTCGG

CGTCAGGGCGCGTCAGCGGGTGTTGGCGGGTGTCGGGGCTGGCTTAACTATGCGGCATCAGAGCAGATTGTACTG
                                                                            8625
GCAGTCCCGCGCAGTCGCCCACAACCGCCCACAGCCCCGACCGAATTGATACGCCGTAGTCTCGTCTAACATGAC

           NdeI                                         KasI NarI SfoI PluTI
AGAGTGCACCATATGCGGTGTGAAATACCGCACAGATGCGTAAGGAGAAAATACCGCATCAGGCGCCATTCGCCA
                                                                            8700
TCTCACGTGGTATACGCCACACTTTATGGCGTGTCTACGCATTCCTCTTTTATGGCGTAGTCCGCGGTAAGCGGT

TTCAGGCTGCGCAACTGTTGGGAAGGGCGATCGGTGCGGGCCTCTTCGCTATTACGCCAGCTGGCGAAAGGGGGA
                                                                            8775
AAGTCCGACGCGTTGACAACCCTTCCCGCTAGCCACGCCCGGAGAAGCGATAATGCGGTCGACCGCTTTCCCCCT

TGTGCTGCAAGGCGATTAAGTTGGGTAACGCCAGGGTTTTCCCAGTCACGACGTTGTAAAACGACGGCCAGTGAA
                                                                            8850
ACACGACGTTCCGCTAATTCAACCCATTGCGGTCCCAAAAGGGTCAGTGCTGCAACATTTTGCTGCCGGTCACTT

TTG    3′
   ...    8853
AAC    5′
```
