# Supplementary material for: An alternative for proteinase K-heat-sensitive protease from fungus Onygena corvina for biotechnology: cloning, engineering, expression, characterization and special application for protein sequencing
Source: Microb Cell Fact. 2020 Jun 24;19:135. doi: 10.1186/s12934-020-01392-3 (PMC7313183; doi:10.1186/s12934-020-01392-3)
Supplement: Supplementary file 9 — Additional file 9. Synthetic DNA fragment sequence, translation and features, comprising optimized SP-PRO-NHSSP gene with deleted C-terminal 4 aa and His6-tag. Secretion peptide native—marked in green, propeptide—marked in yellow. [file 12934_2020_1392_MOESM9_ESM.pdf]

*Start* (0)

**EcoRI**

5′ GCCAGAATTCATGGGTTGTATCAAGGTTATCTCCGTTTTCTTGGCTGCTGTTGCT 55
3′ CGGTCTTAAGTACCCAACATAGTTCCAATAGAGGCAAAAGAACCGACGACAACGA

1 5 10 15
M G C I K V I S V F L A A V A
SP-PRO_NHSSP_C-deletion

1 5 10 15
M G C I K V I S V F L A A V A
Secretion Peptide native

GCTGTTGACGCTAGAGCTTTTTTCCACAACAAGGGTGGTAACGACATCATCCCAA 110
CGACAACTGCGATCTCGAAAAAAGGTGTTGTTCCCACCATTGCTGTAGTAGGGTT

20 25 30
A V D A R A F F H N K G G N D I I P
SP-PRO_NHSSP_C-deletion

A V D A R A F F H N K G G N D I I P
(in frame with Secretion Peptide native)
Secretion Peptide native

Propeptide

ACTCCTACATCGTTGTTATGAAGGACGGTATCTCCACTCAAGAGTTCGAGTCCCA 165
TGAGGATGTAGCAACAATACTTCCTGCCATAGAGGTGAGTTCTCAAGCTCAGGGT

35 40 45 50
N S Y I V V M K D G I S T Q E F E S H
SP-PRO_NHSSP_C-deletion

N S Y I V V M K D G I S T Q E F E S H
(in frame with Secretion Peptide native)

Propeptide

**BstXI**

CATTTCTTCCGTTTCTGCTACTCACGCTAAGAGATCCACTGAGTTGGTTGGTCAC 220
GTAAAGAAGGCAAAGACGATGAGTGCGATTCTCTAGGTGACTCAACCAACCAGTG

55 60 65 70
I S S V S A T H A K R S T E L V G H
SP-PRO_NHSSP_C-deletion

I S S V S A T H A K R S T E L V G H
(in frame with Secretion Peptide native)

Propeptide

```
AAGGATTCCTTCAACATCAACGGTTGGAGAGCTTACAACGGTCACTTCGATGCTG   275
TTCCTAAGGAAGTTGTAGTTGCCAACCTCTCGAATGTTGCCAGTGAAGCTACGAC
          75        80        85
K  D  S  F  N  I  N  G  W  R  A  Y  N  G  H  F  D  A
SP-PRO_NHSSP_C-deletion
K  D  S  F  N  I  N  G  W  R  A  Y  N  G  H  F  D  A
(in frame with Secretion Peptide native)
Propeptide

                     AclI
CTACTTTGGAGTCCATTTTGAACGACGACAACGTTAAGTACGTTGAGCACGACAG   330
GATGAAACCTCAGGTAAAACTTGCTGCTGTTGCAATTCATGCAACTCGTGCTGTC
   90        95        100       105
A  T  L  E  S  I  L  N  D  D  N  V  K  Y  V  E  H  D  R
SP-PRO_NHSSP_C-deletion
A  T  L  E  S  I  L  N  D  D  N  V  K  Y  V  E  H  D  R
(in frame with Secretion Peptide native)
Propeptide

AGTTGTTAAGATCTCCGCTTTGACTACTCAGCCAAACGCTCCATCTTGGGGTTTG   385
TCAACAATTCTAGAGGCGAAACTGATGAGTCGGTTTGCGAGGTAGAACCCCAAAC
      110       115       120       125
V  V  K  I  S  A  L  T  T  Q  P  N  A  P  S  W  G  L
SP-PRO_NHSSP_C-deletion
V  V  K  I  S  A  L  T  T  Q  P  N  A  P  S  W  G  L
(in frame with Secretion Peptide native)
Propeptide

                                          BtsαI
GGTAGAATTTCTCACAGATCCCCAGGTAACAAGGACTTCGTTTACGATGACACTG   440
CCATCTTAAAGAGTGTCTAGGGGTCCATTGTTCCTGAAGCAAATGCTACTGTGAC
          130       135       140
G  R  I  S  H  R  S  P  G  N  K  D  F  V  Y  D  D  T
SP-PRO_NHSSP_C-deletion
G  R  I  S  H  R  S  P  G  N  K  D  F  V  Y  D  D  T
(in frame with Secretion Peptide native)
Propeptide

CTGGTCAGGGTATCACTATCTACGGTGTTGACACTGGAATCGACATCAACCACCC   495
GACCAGTCCCATAGTGATAGATGCCACAACTGTGACCTTAGCTGTAGTTGGTGGG
   145       150       155       160
A  G  Q  G  I  T  I  Y  G  V  D  T  G  I  D  I  N  H  P
SP-PRO_NHSSP_C-deletion
A  G  Q  G  I  T  I  Y  G  V  D  T  G  I  D  I  N  H  P
(in frame with Secretion Peptide native)
Propeptide
```

AGATTTTGGTGGTAGAGCTAGATGGGGAACTAACACTGTTGACAACGCTAACAAC 550
TCTAAAACCACCATCTCGATCTACCCCTTGATTGTGACAACTGTTGCGATTGTTG

165 170 175 180
D F G G R A R W G T N T V D N A N N
SP-PRO_NHSSP_C-deletion

D F G G R A R W G T N T V D N A N N
(in frame with Secretion Peptide native)

GACGGTCATGGTCATGGTACTCATACTGCTGGTACTTTCGCTGGTAACGCTTACG 605
CTGCCAGTACCAGTACCATGAGTATGACGACCATGAAAGCGACCATTGCGAATGC

185 190 195
D G H G H G T H T A G T F A G N A Y
SP-PRO_NHSSP_C-deletion

D G H G H G T H T A G T F A G N A Y
(in frame with Secretion Peptide native)

**BsaWI**

GTATTGCTAAGAAGGCTTCCGTTGTTGCTGTTAAGGTTTTGTCTGCTTCTGGTTC 660
CATAACGATTCTTCCGAAGGCAACAACGACAATTCCAAAACAGACGAAGACCAAG

200 205 210 215
G I A K K A S V V A V K V L S A S G S
SP-PRO_NHSSP_C-deletion

G I A K K A S V V A V K V L S A S G S
(in frame with Secretion Peptide native)

CGGTTCCAACGCTGGTGTTATTAAGGGTATCGACTGGTGTGTTACAGACGCTAGA 715
GCCAAGGTTGCGACCACAATAATTCCCATAGCTGACCACACAATGTCTGCGATCT

220 225 230 235
G S N A G V I K G I D W C V T D A R
SP-PRO_NHSSP_C-deletion

G S N A G V I K G I D W C V T D A R
(in frame with Secretion Peptide native)

TCTAAGGGTGCTTTGGGTAAGGCTGCTTTGAACTTGTCTTTGGGTGGTGGTTTCA 770
AGATTCCCACGAAACCCATTCCGACGAAACTTGAACAGAAACCCACCACCAAAGT

240 245 250
S K G A L G K A A L N L S L G G G F
SP-PRO_NHSSP_C-deletion

S K G A L G K A A L N L S L G G G F
(in frame with Secretion Peptide native)

Eco53kI SacI BanII

ACCAGGCTACAAACGACGCTGTTACTAGAGCTCAGACTGCTGGAATCTTCGTTGC 825
TGGTCCGATGTTTGCTGCGACAATGATCTCGAGTCTGACGACCTTAGAAGCAACG

255 260 265 270
N Q A T N D A V T R A Q T A G I F V A
SP-PRO_NHSSP_C-deletion
N Q A T N D A V T R A Q T A G I F V A
(in frame with Secretion Peptide native)

AlwNI

TGTTGCTGCTGGTAACGATAACAAGGACGCTAGAAACTACTCTCCAGCTTCTGCT 880
ACAACGACGACCATTGCTATTGTTCCTGCGATCTTTGATGAGAGGTCGAAGACGA

275 280 285 290
V A A G N D N K D A R N Y S P A S A
SP-PRO_NHSSP_C-deletion
V A A G N D N K D A R N Y S P A S A
(in frame with Secretion Peptide native)

PvuII MspA1I TatI AhdI

CCAGCTGTTTGTACTGTTGCTTCCTCCACTATCGACGACCAAAAGTCCTCTTTCT 935
GGTCGACAAACATGACAACGAAGGAGGTGATAGCTGCTGGTTTTCAGGAGAAAGA

295 300 305
P A V C T V A S S T I D D Q K S S F
SP-PRO_NHSSP_C-deletion
P A V C T V A S S T I D D Q K S S F
(in frame with Secretion Peptide native)

BmrI

CTAACTGGGGTTCCATCGTTGACATCTACGCTCCAGGTTCCAACATTATTTCCGA 990
GATTGACCCCAAGGTAGCAACTGTAGATGCGAGGTCCAAGGTTGTAATAAAGGCT

310 315 320 325
S N W G S I V D I Y A P G S N I I S D
SP-PRO_NHSSP_C-deletion
S N W G S I V D I Y A P G S N I I S D
(in frame with Secretion Peptide native)

AflIII

TGCTCCAGGTGGTGGTGTTAGAACTATGTCTGGTACTTCTATGGCTTCCCCACAC 1045
ACGAGGTCCACCACCACAATCTTGATACAGACCATGAAGATACCGAAGGGGTGTG

330 335 340 345
A P G G G V R T M S G T S M A S P H
SP-PRO_NHSSP_C-deletion
A P G G G V R T M S G T S M A S P H
(in frame with Secretion Peptide native)

AleI

GTTTGTGGTGCTGGTGCTGCTATGTTGGCTCAAGGTGTTCCAGTTGGTCAGGTTT 1100
CAAACACCACGACCACGACGATACAACCGAGTTCCACAAGGTCAACCAGTCCAAA

350 355 360
V C G A G A A M L A Q G V P V G Q V
SP-PRO_NHSSP_C-deletion

V C G A G A A M L A Q G V P V G Q V
(in frame with Secretion Peptide native)

GTGACAGATTGAAGCAGATCGGTAACGCTGTTGTTAGAAACCCAGGTACTTCCAC 1155
CACTGTCTAACTTCGTCTAGCCATTGCGACAACAATCTTTGGGTCCATGAAGGTG

365 370 375 380
C D R L K Q I G N A V V R N P G T S T
SP-PRO_NHSSP_C-deletion

C D R L K Q I G N A V V R N P G T S T
(in frame with Secretion Peptide native)

**BanI**
**Acc65I** **KpnI** *End* (1199)

TACAAACAGATTGTTGTATAACGGTTCCTAATGAGGTACCCGGC 3′
1199
ATGTTTGTCTAACAACATATTGCCAAGGATTACTCCATGGGCCG 5′

385 390
T N R L L Y N G S * *
SP-PRO_NHSSP_C-deletion

T N R L L Y N G S *
(in frame with Secretion Peptide native)
