# Supplementary material for: An alternative for proteinase K-heat-sensitive protease from fungus Onygena corvina for biotechnology: cloning, engineering, expression, characterization and special application for protein sequencing
Source: Microb Cell Fact. 2020 Jun 24;19:135. doi: 10.1186/s12934-020-01392-3 (PMC7313183; doi:10.1186/s12934-020-01392-3)
Supplement: Supplementary file 12 — Additional file 12. pPink-HC-del-NHSSP plasmid sequence and features. Secretion peptide native—marked in green, propeptide—marked in yellow. [file 12934_2020_1392_MOESM12_ESM.pdf]

```
5′ AGATCTAACATCCAAAGACGAAAGGTTGAATGAAACCTTTTTGCCATCCGACATCCACAGGTCCATTCTCACACA  75
3′ TCTAGATTGTAGGTTTCTGCTTTCCAACTTACTTTGGAAAAACGGTAGGCTGTAGGTGTCCAGGTAAGAGTGTGT
   AOX1 promoter

TAAGTGCCAAACGCAACAGGAGGGGATACACTAGCAGCAGACCGTTGCAAACGCAGGACCTCCACTCCTCTTCTC  150
ATTCACGGTTTGCGTTGTCCTCCCCTATGTGATCGTCGTCTGGCAACGTTTGCGTCCTGGAGGTGAGGAGAAGAG
   AOX1 promoter

CTCAACACCCACTTTTGCCATCGAAAAACCAGCCCAGTTATTGGGCTTGATTGGAGCTCGCTCATTCCAATTCCT  225
GAGTTGTGGGTGAAAACGGTAGCTTTTTGGTCGGGTCAATAACCCGAACTAACCTCGAGCGAGTAAGGTTAAGGA
   AOX1 promoter

TCTATTAGGCTACTAACACCATGACTTTATTAGCCTGTCTATCCTGGCCCCCCTGGCGAGGTTCATGTTTGTTTA  300
AGATAATCCGATGATTGTGGTACTGAAATAATCGGACAGATAGGACCGGGGGGACCGCTCCAAGTACAAACAAAT
   AOX1 promoter

TTTCCGAATGCAACAAGCTCCGCATTACACCCGAACATCACTCCAGATGAGGGCTTTCTGAGTGTGGGGTCAAAT  375
AAAGGCTTACGTTGTTCGAGGCGTAATGTGGGCTTGTAGTGAGGTCTACTCCCGAAAGACTCACACCCCAGTTTA
   AOX1 promoter

                                         PmeI
AGTTTCATGTTCCCCAAATGGCCCAAAACTGACAGTTTAAACGCTGTCTTGGAACCTAATATGACAAAAGCGTGA  450
TCAAAGTACAAGGGGTTTACCGGGTTTTGACTGTCAAATTTGCGACAGAACCTTGGATTATACTGTTTTCGCACT
   AOX1 promoter

TCTCATCCAAGATGAACTAAGTTTGGTTCGTTGAAATGCTAACGGCCAGTTGGTCAAAAAGAAACTTCCAAAAGT  525
AGAGTAGGTTCTACTTGATTCAAACCAAGCAACTTTACGATTGCCGGTCAACCAGTTTTTCTTTGAAGGTTTTCA
   AOX1 promoter

CGGCATACCGTTTGTCTTGTTTGGTATTGATTGACGAATGCTCAAAAATAATCTCATTAATGCTTAGCGCAGTCT  600
GCCGTATGGCAAACAGAACAAACCATAACTAACTGCTTACGAGTTTTTATTAGAGTAATTACGAATCGCGTCAGA
   AOX1 promoter

CTCTATCGCTTCTGAACCCCGGTGCACCTGTGCCGAAACGCAAATGGGGAAACACCCGCTTTTTGGATGATTATG  675
GAGATAGCGAAGACTTGGGGCCACGTGGACACGGCTTTGCGTTTACCCCTTTGTGGGCGAAAAACCTACTAATAC
   AOX1 promoter

CATTGTCTCCACATTGTATGCTTCCAAGATTCTGGTGGGAATACTGCTGATAGCCTAACGTTCATGATCAAAATT  750
GTAACAGAGGTGTAACATACGAAGGTTCTAAGACCACCCTTATGACGACTATCGGATTGCAAGTACTAGTTTTAA
   AOX1 promoter

TAACTGTTCTAACCCCTACTTGACAGCAATATATAAACAGAAGGAAGCTGCCCTGTCTTAAACCTTTTTTTTTAT  825
ATTGACAAGATTGGGGATGAACTGTCGTTATATATTTGTCTTCCTTCGACGGGACAGAATTTGGAAAAAAAAATA
   AOX1 promoter

CATCATTATTAGCTTACTTTCATAATTGCGACTGGTTCCAATTGACAAGCTTTTGATTTTAACGACTTTTAACGA  900
GTAGTAATAATCGAATGAAAGTATTAACGCTGACCAAGGTTAACTGTTCGAAAACTAAAATTGCTGAAAATTGCT
   AOX1 promoter
```

EcoRI

CAACTTGAGAAGATCAAAAAACAACTAATTATTCGAAACGGAATTCATGGGTTGTATCAAGGTTATCTCCGTTTT 975
GTTGAACTCTTCTAGTTTTTTGTTGATTAATAAGCTTTGCCTTAAGTACCCAACATAGTTCCAATAGAGGCAAAA

1 5 10
M G C I K V I S V F

AOX1 promoter
MCS
SP-PRO-NHSSP
Secretion Peptide native

CTTGGCTGCTGTTGCTGCTGTTGACGCTAGAGCTTTTTTCCACAACAAGGGTGGTAACGACATCATCCCAAACTC 1050
GAACCGACGACAACGACGACAACTGCGATCTCGAAAAAAGGTGTTGTTCCCACCATTGCTGTAGTAGGGTTTGAG

15 20 25 30 35
L A A V A A V D A R A F F H N K G G N D I I P N S

SP-PRO-NHSSP
Secretion Peptide native
Propeptide

CTACATCGTTGTTATGAAGGACGGTATCTCCACTCAAGAGTTCGAGTCCCACATTTCTTCCGTTTCTGCTACTCA 1125
GATGTAGCAACAATACTTCCTGCCATAGAGGTGAGTTCTCAAGCTCAGGGTGTAAAGAAGGCAAAGACGATGAGT

40 45 50 55 60
Y I V V M K D G I S T Q E F E S H I S S V S A T H

SP-PRO-NHSSP
Propeptide

CGCTAAGAGATCCACTGAGTTGGTTGGTCACAAGGATTCCTTCAACATCAACGGTTGGAGAGCTTACAACGGTCA 1200
GCGATTCTCTAGGTGACTCAACCAACCAGTGTTCCTAAGGAAGTTGTAGTTGCCAACCTCTCGAATGTTGCCAGT

65 70 75 80 85
A K R S T E L V G H K D S F N I N G W R A Y N G H

SP-PRO-NHSSP
Propeptide

CTTCGATGCTGCTACTTTGGAGTCCATTTTGAACGACGACAACGTTAAGTACGTTGAGCACGACAGAGTTGTTAA 1275
GAAGCTACGACGATGAAACCTCAGGTAAAACTTGCTGCTGTTGCAATTCATGCAACTCGTGCTGTCTCAACAATT

90 95 100 105 110
F D A A T L E S I L N D D N V K Y V E H D R V V K

SP-PRO-NHSSP
Propeptide

GATCTCCGCTTTGACTACTCAGCCAAACGCTCCATCTTGGGGTTTGGGTAGAATTTCTCACAGATCCCCAGGTAA 1350
CTAGAGGCGAAACTGATGAGTCGGTTTGCGAGGTAGAACCCCAAACCCATCTTAAAGAGTGTCTAGGGGTCCATT

115 120 125 130 135
I S A L T T Q P N A P S W G L G R I S H R S P G N

SP-PRO-NHSSP
Propeptide

CAAGGACTTCGTTTACGATGACACTGCTGGTCAGGGTATCACTATCTACGGTGTTGACACTGGAATCGACATCAA 1425
GTTCCTGAAGCAAATGCTACTGTGACGACCAGTCCCATAGTGATAGATGCCACAACTGTGACCTTAGCTGTAGTT

140 145 150 155 160
K D F V Y D D T A G Q G I T I Y G V D T G I D I N

SP-PRO-NHSSP
Propeptide

CCACCCAGATTTTGGTGGTAGAGCTAGATGGGGAACTAACACTGTTGACAACGCTAACAACGACGGTCATGGTCA 1500
GGTGGGTCTAAAACCACCATCTCGATCTACCCCTTGATTGTGACAACTGTTGCGATTGTTGCTGCCAGTACCAGT

165 170 175 180 185
H P D F G G R A R W G T N T V D N A N N D G H G H

SP-PRO-NHSSP

TGGTACTCATACTGCTGGTACTTTCGCTGGTAACGCTTACGGTATTGCTAAGAAGGCTTCCGTTGTTGCTGTTAA 1575
ACCATGAGTATGACGACCATGAAAGCGACCATTGCGAATGCCATAACGATTCTTCCGAAGGCAACAACGACAATT

190 195 200 205 210
G T H T A G T F A G N A Y G I A K K A S V V A V K

SP-PRO-NHSSP

GGTTTTGTCTGCTTCTGGTTCCGGTTCCAACGCTGGTGTTATTAAGGGTATCGACTGGTGTGTTACAGACGCTAG 1650
CCAAAACAGACGAAGACCAAGGCCAAGGTTGCGACCACAATAATTCCCATAGCTGACCACACAATGTCTGCGATC
215 220 225 230 235
V L S A S G S G S N A G V I K G I D W C V T D A R
SP-PRO-NHSSP

ATCTAAGGGTGCTTTGGGTAAGGCTGCTTTGAACTTGTCTTTGGGTGGTGGTTTCAACCAGGCTACAAACGACGC 1725
TAGATTCCCACGAAACCCATTCCGACGAAACTTGAACAGAAACCCACCACCAAAGTTGGTCCGATGTTTGCTGCG
240 245 250 255 260
S K G A L G K A A L N L S L G G G F N Q A T N D A
SP-PRO-NHSSP

TGTTACTAGAGCTCAGACTGCTGGAATCTTCGTTGCTGTTGCTGCTGGTAACGATAACAAGGACGCTAGAAACTA 1800
ACAATGATCTCGAGTCTGACGACCTTAGAAGCAACGACAACGACGACCATTGCTATTGTTCCTGCGATCTTTGAT
265 270 275 280 285
V T R A Q T A G I F V A V A A G N D N K D A R N Y
SP-PRO-NHSSP

CTCTCCAGCTTCTGCTCCAGCTGTTTGTACTGTTGCTTCCTCCACTATCGACGACCAAAAGTCCTCTTTCTCTAA 1875
GAGAGGTCGAAGACGAGGTCGACAAACATGACAACGAAGGAGGTGATAGCTGCTGGTTTTCAGGAGAAAGAGATT
290 295 300 305 310
S P A S A P A V C T V A S S T I D D Q K S S F S N
SP-PRO-NHSSP

CTGGGGTTCCATCGTTGACATCTACGCTCCAGGTTCCAACATTATTTCCGATGCTCCAGGTGGTGGTGTTAGAAC 1950
GACCCCAAGGTAGCAACTGTAGATGCGAGGTCCAAGGTTGTAATAAAGGCTACGAGGTCCACCACCACAATCTTG
315 320 325 330 335
W G S I V D I Y A P G S N I I S D A P G G G V R T
SP-PRO-NHSSP

TATGTCTGGTACTTCTATGGCTTCCCCACACGTTTGTGGTGCTGGTGCTGCTATGTTGGCTCAAGGTGTTCCAGT 2025
ATACAGACCATGAAGATACCGAAGGGGTGTGCAAACACCACGACCACGACGATACAACCGAGTTCCACAAGGTCA
340 345 350 355 360
M S G T S M A S P H V C G A G A A M L A Q G V P V
SP-PRO-NHSSP

TGGTCAGGTTTGTGACAGATTGAAGCAGATCGGTAACGCTGTTGTTAGAAACCCAGGTACTTCCACTACAAACAG 2100
ACCAGTCCAAACACTGTCTAACTTCGTCTAGCCATTGCGACAACAATCTTTGGGTCCATGAAGGTGATGTTTGTC
365 370 375 380 385
G Q V C D R L K Q I G N A V V R N P G T S T T N R
SP-PRO-NHSSP

NgoMIV **NaeI**
KpnI
Acc65I **FseI** **SwaI** **EcoRV**
ATTGTTGTATAACGGTTCCTAATGAGGTACCGGCCGGCCATTTAAATACAGGCCCCTTTTCCTTTGTCGATATCA 2175
TAACAACATATTGCCAAGGATTACTCCATGGCCGGCCGGTAAATTTATGTCCGGGGAAAAGGAAACAGCTATAGT
390
L L Y N G S *
SP-PRO-NHSSP MCS
CYC1 terminator

TGTAATTAGTTATGTCACGCTTACATTCACGCCCTCCTCCCACATCCGCTCTAACCGAAAAGGAAGGAGTTAGAC 2250
ACATTAATCAATACAGTGCGAATGTAAGTGCGGGAGGAGGGTGTAGGCGAGATTGGCTTTTCCTTCCTCAATCTG
CYC1 terminator

AACCTGAAGTCTAGGTCCCTATTTATTTTTTTTAATAGTTATGTTAGTATTAAGAACGTTATTTATATTTCAAAT 2325
TTGGACTTCAGATCCAGGGATAAATAAAAAAAATTATCAATACAATCATAATTCTTGCAATAAATATAAAGTTTA
CYC1 terminator

BsrGI MluI

TTTTCTTTTTTTTTCTGTACAAACGCGTGTACGCATGTAACATTATACTGAAAACCTTGCTTGAGAAGGTTTTGGG 2400
AAAAGAAAAAAAAAGACATGTTTGCGCACATGCGTACATTGTAATATGACTTTTGGAACGAACTCTTCCAAAACCC

CYC1 terminator

BamHI NotI SacII

ACGCTCGAAGGCTTTAATTTGCAAGCTGGATCCGCGGCCGCCTTCCAAACTCTCATGGATTCTCAGGTAATAGGT 2475
TGCGAGCTTCCGAAATTAAACGTTCGACCTAGGCGCCGGCGGAAGGTTTGAGAGTACCTAAGAGTCCATTATCCA

1 5
M D S Q V I G

CYC1 terminator PpADE2 HC promoter PpADE2

NheI BmtI

ATTCTAGGAGGAGGCCAGCTAGGCCGAATGATTGTTGAGGCCGCTAGCAGGCTCAATATCAAGACCGTGATTCTT 2550
TAAGATCCTCCTCCGGTCGATCCGGCTTACTAACAACTCCGGCGATCGTCCGAGTTATAGTTCTGGCACTAAGAA

10 15 20 25 30
I L G G G Q L G R M I V E A A S R L N I K T V I L

PpADE2

GATGATGGTTTTTCACCTGCTAAGCACATTAATGCTGCGCAAGACCACATCGACGGATCATTCAAAGATGAGGAG 2625
CTACTACCAAAAAGTGGACGATTCGTGTAATTACGACGCGTTCTGGTGTAGCTGCCTAGTAAGTTTCTACTCCTC

35 40 45 50 55
D D G F S P A K H I N A A Q D H I D G S F K D E E

PpADE2

GCTATCGCCAAGTTAGCTGCCAAATGTGATGTTCTCACTGTAGAGATTGAGCATGTCAACACAGATGCTCTAAAG 2700
CGATAGCGGTTCAATCGACGGTTTACACTACAAGAGTGACATCTCTAACTCGTACAGTTGTGTCTACGAGATTTC

60 65 70 75 80
A I A K L A A K C D V L T V E I E H V N T D A L K

PpADE2

AGAGTTCAAGACAGAACTGGAATCAAGATATATCCTTTACCAGAGACAATCGAACTAATCAAGGATAAGTACTTG 2775
TCTCAAGTTCTGTCTTGACCTTAGTTCTATATAGGAAATGGTCTCTGTTAGCTTGATTAGTTCCTATTCATGAAC

85 90 95 100 105
R V Q D R T G I K I Y P L P E T I E L I K D K Y L

PpADE2

CAAAAGGAACATTTGATCAAGCACAACATTTCGGTGACAAAGTCTCAGGGTATAGAATCTAATGAAAAGGCGCTG 2850
GTTTTCCTTGTAAACTAGTTCGTGTTGTAAAGCCACTGTTTCAGAGTCCCATATCTTAGATTACTTTTCCGCGAC

110 115 120 125 130
Q K E H L I K H N I S V T K S Q G I E S N E K A L

PpADE2

CTTTTGTTTGGAGAAGAGAATGGATTTCCATATCTGTTGAAGTCCCGGACTATGGCTTATGATGGAAGAGGCAAT 2925
GAAAACAAACCTCTTCTCTTACCTAAAGGTATAGACAACTTCAGGGCCTGATACCGAATACTACCTTCTCCGTTA

135 140 145 150 155
L L F G E E N G F P Y L L K S R T M A Y D G R G N

PpADE2

TTTGTAGTGGAGTCTAAAGAGGACATCAGTAAGGCATTAGAGTTCTTGAAAGATCGTCCATTGTATGCCGAGAAG 3000
AAACATCACCTCAGATTTCTCCTGTAGTCATTCCGTAATCTCAAGAACTTTCTAGCAGGTAACATACGGCTCTTC

160 165 170 175 180
F V V E S K E D I S K A L E F L K D R P L Y A E K

PpADE2

TTTGCTCCTTTTGTTAAAGAATTAGCGGTAATGGTTGTGAGATCACTGGAAGGCGAAGTATTCTCCTACCCAACC 3075
AAACGAGGAAAACAATTTCTTAATCGCCATTACCAACACTCTAGTGACCTTCCGCTTCATAAGAGGATGGGTTGG

185 190 195 200 205
F A P F V K E L A V M V V R S L E G E V F S Y P T

PpADE2

GTAGAAACTGTGCACAAGGACAATATCTGTCATATTGTGTATGCTCCGGCCAGAGTTAATGACACCATCCAAAAG 3150
CATCTTTGACACGTGTTCCTGTTATAGACAGTATAACACATACGAGGCCGGTCTCAATTACTGTGGTAGGTTTTC

210 215 220 225 230
V E T V H K D N I C H I V Y A P A R V N D T I Q K
PpADE2

BbsI

AAAGCTCAAATATTAGCTGAAAACACTGTGAAGACTTTCCCAGGCGCTGGAATCTTCGGAGTTGAGATGTTCCTA 3225
TTTCGAGTTTATAATCGACTTTTGTGACACTTCTGAAAGGGTCCGCGACCTTAGAAGCCTCAACTCTACAAGGAT

235 240 245 250 255
K A Q I L A E N T V K T F P G A G I F G V E M F L
PpADE2

TTGTCTGATGGAGAACTTCTTGTAAATGAGATTGCTCCAAGGCCCCACAATTCTGGTCACTATACAATCGATGCA 3300
AACAGACTACCTCTTGAAGAACATTTACTCTAACGAGGTTCCGGGGTGTTAAGACCAGTGATATGTTAGCTACGT

260 265 270 275 280
L S D G E L L V N E I A P R P H N S G H Y T I D A
PpADE2

TGTGTAACATCTCAGTTCGAAGCACATGTAAGAGCCATAACTGGTCTGCCAATGCCACTAGATTTCACCAAACTA 3375
ACACATTGTAGAGTCAAGCTTCGTGTACATTCTCGGTATTGACCAGACGGTTACGGTGATCTAAAGTGGTTTGAT

285 290 295 300 305
C V T S Q F E A H V R A I T G L P M P L D F T K L
PpADE2

TCTACTTCCAACACCAACGCTATTATGCTCAATGTTTTGGGTGCTGAAAAATCTCACGGGGAATTAGAGTTTTGT 3450
AGATGAAGGTTGTGGTTGCGATAATACGAGTTACAAAACCCACGACTTTTTAGAGTGCCCCTTAATCTCAAAACA

310 315 320 325 330
S T S N T N A I M L N V L G A E K S H G E L E F C
PpADE2

DraIII

AGAAGAGCCTTAGAAACACCCGGTGCTTCTGTATATCTGTACGGAAAGACCACCCGATTGGCTCGTAAGATGGGT 3525
TCTTCTCGGAATCTTTGTGGGCCACGAAGACATATAGACATGCCTTTCTGGTGGGCTAACCGAGCATTCTACCCA

335 340 345 350 355
R R A L E T P G A S V Y L Y G K T T R L A R K M G
PpADE2

CATATCAACATAATAGGATCTTCCATGTTGGAAGCAGAACAAAAGTTAGAGTACATTCTAGAAGAATCAACCCAC 3600
GTATAGTTGTATTATCCTAGAAGGTACAACCTTCGTCTTGTTTTCAATCTCATGTAAGATCTTCTTAGTTGGGTG

360 365 370 375 380
H I N I I G S S M L E A E Q K L E Y I L E E S T H
PpADE2

TTACCATCCAGTACTGTATCAGCTGACACTAAACCGTTGGTTGGAGTTATCATGGGTTCAGACTCTGATCTACCT 3675
AATGGTAGGTCATGACATAGTCGACTGTGATTTGGCAACCAACCTCAATAGTACCCAAGTCTGAGACTAGATGGA

385 390 395 400 405
L P S S T V S A D T K P L V G V I M G S D S D L P
PpADE2

GTGATTTCGAAAGGTTGCGATATTTTAAAACAGTTTGGTGTTCCATTCGAAGTTACTATTGTCTCTGCTCATAGA 3750
CACTAAAGCTTTCCAACGCTATAAAATTTTGTCAAACCACAAGGTAAGCTTCAATGATAACAGAGACGAGTATCT

410 415 420 425 430
V I S K G C D I L K Q F G V P F E V T I V S A H R
PpADE2

ACACCACAGAGAATGACCAGATATGCCTTTGAAGCCGCTAGTAGAGGTATCAAGGCTATCATTGCAGGTGCTGGT 3825
TGTGGTGTCTCTTACTGGTCTATACGGAAACTTCGGCGATCATCTCCATAGTTCCGATAGTAACGTCCACGACCA

435 440 445 450 455
T P Q R M T R Y A F E A A S R G I K A I I A G A G
PpADE2

GGTGCTGCTCATCTTCCAGGAATGGTTGCTGCCATGACTCCGTTGCCAGTCATTGGTGTTCCTGTCAAGGGCTCT 3900
CCACGACGAGTAGAAGGTCCTTACCAACGACGGTACTGAGGCAACGGTCAGTAACCACAAGGACAGTTCCCGAGA
460 465 470 475 480
G A A H L P G M V A A M T P L P V I G V P V K G S
PpADE2

ACGTTGGATGGTGTAGACTCGCTACACTCGATTGTCCAAATGCCTAGAGGTGTTCCTGTGGCTACGGTTGCTATC 3975
TGCAACCTACCACATCTGAGCGATGTGAGCTAACAGGTTTACGGATCTCCACAAGGACACCGATGCCAACGATAG
485 490 495 500 505
T L D G V D S L H S I V Q M P R G V P V A T V A I
PpADE2

AACAACGCCACCAATGCCGCTCTGTTGGCCATCAGGATTTTAGGTACAATTGACCACAAATGGCAAAAGGAAATG 4050
TTGTTGCGGTGGTTACGGCGAGACAACCGGTAGTCCTAAAATCCATGTTAACTGGTGTTTACCGTTTTCCTTTAC
510 515 520 525 530
N N A T N A A L L A I R I L G T I D H K W Q K E M
PpADE2

TCCAAGTATATGAATGCAATGGAGACCGAAGTGTTGGGGAAGGCATCCAACTTGGAATCTGAAGGGTATGAATCC 4125
AGGTTCATATACTTACGTTACCTCTGGCTTCACAACCCCTTCCGTAGGTTGAACCTTAGACTTCCCATACTTAGG
535 540 545 550 555
S K Y M N A M E T E V L G K A S N L E S E G Y E S
PpADE2

BsaAI

TATTTGAAGAATCGTCTTTGAATTTAGTATTGTTTTTTAATAGATGTATATATAATAGTACACGTAACTTATCTA 4200
ATAAACTTCTTAGCAGAAACTTAAATCATAACAAAAAATTATCTACATATATATTATCATGTGCATTGAATAGAT
560
Y L K N R L *
PpADE2

TTCCATTCATAATTTTATTTTAAAGGTTCGGTAGAAATTTGTCCTCCAAAAAGTTGGTTAGAGCCTGGCAGTTTT 4275
AAGGTAAGTATTAAAATAAAATTTCCAAGCCATCTTTAAACAGGAGGTTTTTCAACCAATCTCGGACCGTCAAAA

BsgI **AscI** **BssHII**

GATAGGCATTATTATAGATTGGGTAATATTTACCCTGCACCTGGAGGAACTTTGCAAAGAGCCTCATGTGCGGCG 4350
CTATCCGTAATAATATCTAACCCATTATAAATGGGACGTGGACCTCCTTGAAACGTTTCTCGGAGTACACGCCGC

CGCCAGGCCATAATGGCCAAACGGTTTCTCAATTACTATATACTACTAACCATTTACCTGTAGCGTATTTCTTTT 4425
GCGGTCCGGTATTACCGGTTTGCCAAAGAGTTAATGATATATGATGATTGGTAAATGGACATCGCATAAAGAAAA
PpTRP2 promoter

NruI

CCCTCTTCGCGAAAGCTCAAGGGCATCTTCTTGACTCATGAAAAATATCTGGATTTCTTCTGACAGATCATCACC 4500
GGGAGAAGCGCTTTCGAGTTCCCGTAGAAGAACTGAGTACTTTTTATAGACCTAAAGAAGACTGTCTAGTAGTGG
PpTRP2 promoter

BsaBI

CTTGAGCCCAACTCTCTAGCCTATGAGTGTAAGTGATAGTCATCTTGCAACAGATTATTTTGGAACGCAACTAAC 4575
GAACTCGGGTTGAGAGATCGGATACTCACATTCACTATCAGTAGAACGTTGTCTAATAAAACCTTGCGTTGATTG
1 5 10 15
M S V S D S H L A T D Y F G T Q L T
PpTRP2 promoter
PpTRP2

```
AAAGCAGATACACCCTTCAGCAGAATCCTTTCTGGATATTGTGAAGAATGATCGCCAAAGTCACAGTCCTGAGAC     4650
TTTCGTCTATGTGGGAAGTCGTCTTAGGAAAGACCTATAACACTTCTTACTAGCGGTTTCAGTGTCAGGACTCTG
        20                      25                      30                      35                      40
  K   Q   I   H   P   S   A   E   S   F   L   D   I   V   K   N   D   R   Q   S   H   S   P   E   T
PpTRP2

AGTTCCTAATCTTTACCCCATTTACAAGTTCATCCAATCAGACTTCTTAACGCCTCATCTGGCTTATATCAAGCT     4725
TCAAGGATTAGAAATGGGGTAAATGTTCAAGTAGGTTAGTCTGAAGAATTGCGGAGTAGACCGAATATAGTTCGA
        45                      50                      55                      60                      65
  V   P   N   L   Y   P   I   Y   K   F   I   Q   S   D   F   L   T   P   H   L   A   Y   I   K   L
PpTRP2

TACCAACAGTTCAGAAACTCCCAGTCCAAGTTTCTTGCTTGAAAGTGCGAAGAATGGTGACACCGTTGACAGGTA     4800
ATGGTTGTCAAGTCTTTGAGGGTCAGGTTCAAAGAACGAACTTTCACGCTTCTTACCACTGTGGCAACTGTCCAT
        70                      75                      80                      85                      90
  T   N   S   S   E   T   P   S   P   S   F   L   L   E   S   A   K   N   G   D   T   V   D   R   Y
PpTRP2

CACCTTTATGGGACATTCCCCCAGAAAAATAATCAAGACTGGGCCTTTAGAGGGTGCTGAAGTTGACCCCTTGGT     4875
GTGGAAATACCCTGTAAGGGGGTCTTTTTATTAGTTCTGACCCGGAAATCTCCCACGACTTCAACTGGGGAACCA
        95                      100                     105                     110                     115
  T   F   M   G   H   S   P   R   K   I   I   K   T   G   P   L   E   G   A   E   V   D   P   L   V
PpTRP2

GCTTCTGGAAAAAGAACTGAAGGGCACCAGACAAGCGCAACTTCCTGGTATTCCTCGTCTAAGTGGTGGTGCCAT     4950
CGAAGACCTTTTTCTTGACTTCCCGTGGTCTGTTCGCGTTGAAGGACCATAAGGAGCAGATTCACCACCACGGTA
        120                     125                     130                     135                     140
  L   L   E   K   E   L   K   G   T   R   Q   A   Q   L   P   G   I   P   R   L   S   G   G   A   I
PpTRP2

AGGATACATCTCGTACGATTGTATTAAGTACTTTGAACCAAAAACTGAAAGAAAACTGAAAGATGTTTTGCAACT     5025
TCCTATGTAGAGCATGCTAACATAATTCATGAAACTTGGTTTTTGACTTTCTTTTGACTTTCTACAAAACGTTGA
        145                     150                     155                     160                     165
  G   Y   I   S   Y   D   C   I   K   Y   F   E   P   K   T   E   R   K   L   K   D   V   L   Q   L
PpTRP2

 BspEI
TCCGGAAGCAGCTTTGATGTTGTTCGACACGATCGTGGCTTTTGACAATGTTTATCAAAGATTCCAGGTAATTGG     5100
AGGCCTTCGTCGAAACTACAACAAGCTGTGCTAGCACCGAAAACTGTTACAAATAGTTTCTAAGGTCCATTAACC
        170                     175                     180                     185                     190
  P   E   A   A   L   M   L   F   D   T   I   V   A   F   D   N   V   Y   Q   R   F   Q   V   I   G
PpTRP2

                                                               PsiI
AAACGTTTCTCTATCCGTTGATGACTCGGACGAAGCTATTCTTGAGAAATATTATAAGACAAGAGAAGAAGTGGA     5175
TTTGCAAAGAGATAGGCAACTACTGAGCCTGCTTCGATAAGAACTCTTTATAATATTCTGTTCTCTTCTTCACCT
        195                     200                     205                     210                     215
  N   V   S   L   S   V   D   D   S   D   E   A   I   L   E   K   Y   Y   K   T   R   E   E   V   E
PpTRP2

AAAGATCAGTAAAGTGGTATTTGACAATAAAACTGTTCCCTACTATGAACAGAAAGATATTATTCAAGGCCAAAC     5250
TTTCTAGTCATTTCACCATAAACTGTTATTTTGACAAGGGATGATACTTGTCTTTCTATAATAAGTTCCGGTTTG
        220                     225                     230                     235                     240
  K   I   S   K   V   V   F   D   N   K   T   V   P   Y   Y   E   Q   K   D   I   I   Q   G   Q   T
PpTRP2

GTTCACCTCTAATATTGGTCAGGAAGGGTATGAAAACCATGTTCGCAAGCTGAAAGAACATATTCTGAAAGGAGA     5325
CAAGTGGAGATTATAACCAGTCCTTCCCATACTTTTGGTACAAGCGTTCGACTTTCTTGTATAAGACTTTCCTCT
        245                     250                     255                     260                     265
  F   T   S   N   I   G   Q   E   G   Y   E   N   H   V   R   K   L   K   E   H   I   L   K   G   D
PpTRP2
```

EcoNI

CATCTTCCAAGCTGTTCCCTCTCAAAGGGTAGCCAGGCCGACCTCATTGCACCCTTTCAACATCTATCGTCATTT 5400
GTAGAAGGTTCGACAAGGGAGAGTTTCCCATCGGTCCGGCTGGAGTAACGTGGGAAAGTTGTAGATAGCAGTAAA

270 275 280 285 290
I F Q A V P S Q R V A R P T S L H P F N I Y R H L
PpTRP2

GAGAACTGTCAATCCTTCTCCATACATGTTCTATATTGACTATCTAGACTTCCAAGTTGTTGGTGCTTCACCTGA 5475
CTCTTGACAGTTAGGAAGAGGTATGTACAAGATATAACTGATAGATCTGAAGGTTCAACAACCACGAAGTGGACT

295 300 305 310 315
R T V N P S P Y M F Y I D Y L D F Q V V G A S P E
PpTRP2

SpeI

ATTACTAGTTAAATCCGACAACAACAACAAAATCATCACACATCCTATTGCTGGAACTCTTCCCAGAGGTAAAAC 5550
TAATGATCAATTTAGGCTGTTGTTGTTGTTTTAGTAGTGTGTAGGATAACGACCTTGAGAAGGGTCTCCATTTTG

320 325 330 335 340
L L V K S D N N N K I I T H P I A G T L P R G K T
PpTRP2

BmgBI

TATCGAAGAGGACGACAATTATGCTAAGCAATTGAAGTCGTCTTTGAAAGACAGGGCCGAGCACGTCATGCTGGT 5625
ATAGCTTCTCCTGCTGTTAATACGATTCGTTAACTTCAGCAGAAACTTTCTGTCCCGGCTCGTGCAGTACGACCA

345 350 355 360 365
I E E D D N Y A K Q L K S S L K D R A E H V M L V
PpTRP2

AGATTTGGCCAGAAATGATATTAACCGTGTGTGTGAGCCCACCAGTACCACGGTTGATCGTTTATTGACTGTGGA 5700
TCTAAACCGGTCTTTACTATAATTGGCACACACACTCGGGTGGTCATGGTGCCAACTAGCAAATAACTGACACCT

370 375 380 385 390
D L A R N D I N R V C E P T S T T V D R L L T V E
PpTRP2

GAGATTTTCTCATGTGATGCATCTTGTGTCAGAAGTCAGTGGAACATTGAGACCAAACAAGACTCGCTTCGATGC 5775
CTCTAAAAGAGTACACTACGTAGAACACAGTCTTCAGTCACCTTGTAACTCTGGTTTGTTCTGAGCGAAGCTACG

395 400 405 410 415
R F S H V M H L V S E V S G T L R P N K T R F D A
PpTRP2

TTTCAGATCCATTTTCCCAGCAGGAACCGTCTCCGGTGCTCCGAAGGTAAGAGCAATGCAACTCATAGGAGAATT 5850
AAAGTCTAGGTAAAAGGGTCGTCCTTGGCAGAGGCCACGAGGCTTCCATTCTCGTTACGTTGAGTATCCTCTTAA

420 425 430 435 440
F R S I F P A G T V S G A P K V R A M Q L I G E L
PpTRP2

PshAI

GGAAGGAGAAAAGAGAGGTGTTTATGCGGGGGCCGTAGGACACTGGTCGTACGATGGAAAATCGATGGACACATG 5925
CCTTCCTCTTTTCTCTCCACAAATACGCCCCCGGCATCCTGTGACCAGCATGCTACCTTTTAGCTACCTGTGTAC

445 450 455 460 465
E G E K R G V Y A G A V G H W S Y D G K S M D T C
PpTRP2

AflII

TATTGCCTTAAGAACAATGGTCGTCAAGGACGGTGTCGCTTACCTTCAAGCCGGAGGTGGAATTGTCTACGATTC 6000
ATAACGGAATTCTTGTTACCAGCAGTTCCTGCCACAGCGAATGGAAGTTCGGCCTCCACCTTAACAGATGCTAAG

470 475 480 485 490
I A L R T M V V K D G V A Y L Q A G G G I V Y D S
PpTRP2

```
TGACCCCTATGACGAGTACATCGAAACCATGAACAAAATGAGATCCAACAATAACACCATCTTGGAGGCTGAGAA  6075
ACTGGGGATACTGCTCATGTAGCTTTGGTACTTGTTTTACTCTAGGTTGTTATTGTGGTAGAACCTCCGACTCTT
   495            500            505            510            515
 D  P  Y  D  E  Y  I  E  T  M  N  K  M  R  S  N  N  N  T  I  L  E  A  E  K
PpTRP2 >

AATCTGGACCGATAGGTTGGCCAGAGACGAGAATCAAAGTGAATCCGAAGAAAACGATCAATGAACGGAGGACGT  6150
TTAGACCTGGCTATCCAACCGGTCTCTGCTCTTAGTTTCACTTAGGCTTCTTTTGCTAGTTACTTGCCTCCTGCA
   520            525            530            535
 I  W  T  D  R  L  A  R  D  E  N  Q  S  E  S  E  E  N  D  Q  *
PpTRP2 >

AAGTAGGAATTTATGGTTTGGCCATAATGGCCTAGCTTGGCGTAATCATGGTCATAGCTGTTTCCTGTGTGAAAT  6225
TTCATCCTTAAATACCAAACCGGTATTACCGGATCGAACCGCATTAGTACCAGTATCGACAAAGGACACACTTTA

TGTTATCCGCTCACAATTCCACACAACATACGAGCCGGAAGCATAAAGTGTAAAGCCTGGGGTGCCTAATGAGTG  6300
ACAATAGGCGAGTGTTAAGGTGTGTTGTATGCTCGGCCTTCGTATTTCACATTTCGGACCCCACGGATTACTCAC

AGCTAACTCACATTAATTGCGTTGCGCTCACTGCCCGCTTTCCAGTCGGGAAACCTGTCGTGCCAGCTGCATTAA  6375
TCGATTGAGTGTAATTAACGCAACGCGAGTGACGGGCGAAAGGTCAGCCCTTTGGACAGCACGGTCGACGTAATT

TGAATCGGCCAACGCGCGGGGAGAGGCGGTTTGCGTATTGGGCGCTCTTCCGCTTCCTCGCTCACTGACTCGCTG  6450
ACTTAGCCGGTTGCGCGCCCCTCTCCGCCAAACGCATAACCCGCGAGAAGGCGAAGGAGCGAGTGACTGAGCGAC

CGCTCGGTCGTTCGGCTGCGGCGAGCGGTATCAGCTCACTCAAAGGCGGTAATACGGTTATCCACAGAATCAGGG  6525
GCGAGCCAGCAAGCCGACGCCGCTCGCCATAGTCGAGTGAGTTTCCGCCATTATGCCAATAGGTGTCTTAGTCCC

GATAACGCAGGAAAGAACATGTGAGCAAAAGGCCAGCAAAAGGCCAGGAACCGTAAAAAGGCCGCGTTGCTGGCG  6600
CTATTGCGTCCTTTCTTGTACACTCGTTTTCCGGTCGTTTTCCGGTCCTTGGCATTTTTCCGGCGCAACGACCGC

TTTTTCCATAGGCTCCGCCCCCCTGACGAGCATCACAAAAATCGACGCTCAAGTCAGAGGTGGCGAAACCCGACA  6675
AAAAAGGTATCCGAGGCGGGGGGACTGCTCGTAGTGTTTTTAGCTGCGAGTTCAGTCTCCACCGCTTTGGGCTGT
< ori

GGACTATAAAGATACCAGGCGTTTCCCCCTGGAAGCTCCCTCGTGCGCTCTCCTGTTCCGACCCTGCCGCTTACC  6750
CCTGATATTTCTATGGTCCGCAAAGGGGGACCTTCGAGGGAGCACGCGAGAGGACAAGGCTGGGACGGCGAATGG
< ori

GGATACCTGTCCGCCTTTCTCCCTTCGGGAAGCGTGGCGCTTTCTCATAGCTCACGCTGTAGGTATCTCAGTTCG  6825
CCTATGGACAGGCGGAAAGAGGGAAGCCCTTCGCACCGCGAAAGAGTATCGAGTGCGACATCCATAGAGTCAAGC
< ori

GTGTAGGTCGTTCGCTCCAAGCTGGGCTGTGTGCACGAACCCCCCGTTCAGCCCGACCGCTGCGCCTTATCCGGT  6900
CACATCCAGCAAGCGAGGTTCGACCCGACACACGTGCTTGGGGGGCAAGTCGGGCTGGCGACGCGGAATAGGCCA
< ori

AACTATCGTCTTGAGTCCAACCCGGTAAGACACGACTTATCGCCACTGGCAGCAGCCACTGGTAACAGGATTAGC  6975
TTGATAGCAGAACTCAGGTTGGGCCATTCTGTGCTGAATAGCGGTGACCGTCGTCGGTGACCATTGTCCTAATCG
< ori

AGAGCGAGGTATGTAGGCGGTGCTACAGAGTTCTTGAAGTGGTGGCCTAACTACGGCTACACTAGAAGGACAGTA  7050
TCTCGCTCCATACATCCGCCACGATGTCTCAAGAACTTCACCACCGGATTGATGCCGATGTGATCTTCCTGTCAT
< ori
```

TTTGGTATCTGCGCTCTGCTGAAGCCAGTTACCTTCGGAAAAAGAGTTGGTAGCTCTTGATCCGGCAAACAAACC 7125
AAACCATAGACGCGAGACGACTTCGGTCAATGGAAGCCTTTTTCTCAACCATCGAGAACTAGGCCGTTTGTTTGG
ori

ACCGCTGGTAGCGGTGGTTTTTTTGTTTGCAAGCAGCAGATTACGCGCAGAAAAAAAGGATCTCAAGAAGATCCT 7200
TGGCGACCATCGCCACCAAAAAAACAAACGTTCGTCGTCTAATGCGCGTCTTTTTTTCCTAGAGTTCTTCTAGGA
ori

TTGATCTTTTCTACGGGGTCTGACGCTCAGTGGAACGAAAACTCACGTTAAGGGATTTTGGTCATGAGATTATCA 7275
AACTAGAAAAGATGCCCCAGACTGCGAGTCACCTTGCTTTTGAGTGCAATTCCCTAAAACCAGTACTCTAATAGT

AAAAGGATCTTCACCTAGATCCTTTTAAATTAAAAATGAAGTTTTAAATCAATCTAAAGTATATATGAGTAAACT 7350
TTTTCCTAGAAGTGGATCTAGGAAAATTTAATTTTTACTTCAAAATTTAGTTAGATTTCATATATACTCATTTGA

TGGTCTGACAGTTACCAATGCTTAATCAGTGAGGCACCTATCTCAGCGATCTGTCTATTTCGTTCATCCATAGTT 7425
ACCAGACTGTCAATGGTTACGAATTAGTCACTCCGTGGATAGAGTCGCTAGACAGATAAAGCAAGTAGGTATCAA
285 280 275 270
* W H K I L S A G I E A I Q R N R E D M T
AmpR

GCCTGACTCCCCGTCGTGTAGATAACTACGATACGGGAGGGCTTACCATCTGGCCCCAGTGCTGCAATGATACCG 7500
CGGACTGAGGGGCAGCACATCTATTGATGCTATGCCCTCCCGAATGGTAGACCGGGGTCACGACGTTACTATGGC
265 260 255 250 245
A Q S G T T Y I V V I R S P K G D P G L A A I I G
AmpR

CGAGACCCACGCTCACCGGCTCCAGATTTATCAGCAATAAACCAGCCAGCCGGAAGGGCCGAGCGCAGAAGTGGT 7575
GCTCTGGGTGCGAGTGGCCGAGGTCTAAATAGTCGTTATTTGGTCGGTCGGCCTTCCCGGCTCGCGTCTTCACCA
240 235 230 225 220
R S G R E G A G S K D A I F W G A P L A S R L L P
AmpR

CCTGCAACTTTATCCGCCTCCATCCAGTCTATTAATTGTTGCCGGGAAGCTAGAGTAAGTAGTTCGCCAGTTAAT 7650
GGACGTTGAAATAGGCGGAGGTAGGTCAGATAATTAACAACGGCCCTTCGATCTCATTCATCAAGCGGTCAATTA
215 210 205 200 195
G A V K D A E M W D I L Q Q R S A L T L L E G T L
AmpR

AGTTTGCGCAACGTTGTTGCCATTGCTACAGGCATCGTGGTGTCACGCTCGTCGTTTGGTATGGCTTCATTCAGC 7725
TCAAACGCGTTGCAACAACGGTAACGATGTCCGTAGCACCACAGTGCGAGCAGCAAACCATACCGAAGTAAGTCG
190 185 180 175 170
L K R L T T A M A V P M T T D R E D N P I A E N L
AmpR

TCCGGTTCCCAACGATCAAGGCGAGTTACATGATCCCCCATGTTGTGCAAAAAAGCGGTTAGCTCCTTCGGTCCT 7800
AGGCCAAGGGTTGCTAGTTCCGCTCAATGTACTAGGGGGTACAACACGTTTTTTCGCCAATCGAGGAAGCCAGGA
165 160 155 150 145
E P E W R D L R T V H D G M N H L F A T L E K P G
AmpR

CCGATCGTTGTCAGAAGTAAGTTGGCCGCAGTGTTATCACTCATGGTTATGGCAGCACTGCATAATTCTCTTACT 7875
GGCTAGCAACAGTCTTCATTCAACCGGCGTCACAATAGTGAGTACCAATACCGTCGTGACGTATTAAGAGAATGA
140 135 130 125 120
G I T T L L L N A A T N D S M T I A A S C L E R V
AmpR

GTCATGCCATCCGTAAGATGCTTTTCTGTGACTGGTGAGTACTCAACCAAGTCATTCTGAGAATAGTGTATGCGG 7950
CAGTACGGTAGGCATTCTACGAAAAGACACTGACCACTCATGAGTTGGTTCAGTAAGACTCTTATCACATACGCC
115 110 105 100 95
T M G D T L H K E T V P S Y E V L D N Q S Y H I R
AmpR

CGACCGAGTTGCTCTTGCCCGGCGTCAATACGGGATAATACCGCGCCACATAGCAGAACTTTAAAAGTGCTCATC 8025
GCTGGCTCAACGAGAACGGGCCGCAGTTATGCCCTATTATGGCGCGGTGTATCGTCTTGAAATTTTCACGAGTAG
90 85 80 75 70
R G L Q E Q G A D I R S L V A G C L L V K F T S M
AmpR

ATTGGAAAACGTTCTTCGGGGCGAAAACTCTCAAGGATCTTACCGCTGTTGAGATCCAGTTCGATGTAACCCACT 8100
TAACCTTTTGCAAGAAGCCCCGCTTTTGAGAGTTCCTAGAATGGCGACAACTCTAGGTCAAGCTACATTGGGTGA
65 60 55 50 45
M P F R E E P R F S E L I K G S N L D L E I Y G V
AmpR

CGTGCACCCAACTGATCTTCAGCATCTTTTACTTTCACCAGCGTTTCTGGGTGAGCAAAAACAGGAAGGCAAAAT 8175
GCACGTGGGTTGACTAGAAGTCGTAGAAAATGAAAGTGGTCGCAAAGACCCACTCGTTTTTGTCCTTCCGTTTTA
40 35 30 25 20
R A G L Q D E A D K V K V L T E P H A F V P L C F
signal sequence
AmpR

GCCGCAAAAAAGGGAATAAGGGCGACACGGAAATGTTGAATACTCATACTCTTCCTTTTTCAATATTATTGAAGC 8250
CGGCGTTTTTTCCCTTATTCCCGCTGTGCCTTTACAACTTATGAGTATGAGAAGGAAAAAGTTATAATAACTTCG
15 10 5 1
A A F F P I L A V R F H Q I S M
signal sequence
AmpR promoter
AmpR

ATTTATCAGGGTTATTGTCTCATGAGCGGATACATATTTGAATGTATTTAGAAAAATAAACAAATAGGGGTTCCG 8325
TAAATAGTCCCAATAACAGAGTACTCGCCTATGTATAAACTTACATAAATCTTTTTATTTGTTTATCCCCAAGGC
AmpR promoter

ZraI **AatII**

CGCACATTTCCCCGAAAAGTGCCACCTGACGTCTAAGAAACCATTATTATCATGACATTAACCTATAAAAATAGG 8400
GCGTGTAAAGGGGCTTTTCACGGTGGACTGCAGATTCTTTGGTAATAATAGTACTGTAATTGGATATTTTTATCC
AmpR promoter

CGTATCACGAGGCCCTTTCGTCTCGCGCGTTTCGGTGATGACGGTGAAAACCTCTGACACATGCAGCTCCCGGAG 8475
GCATAGTGCTCCGGGAAAGCAGAGCGCGCAAAGCCACTACTGCCACTTTTGGAGACTGTGTACGTCGAGGGCCTC

ACGGTCACAGCTTGTCTGTAAGCGGATGCCGGGAGCAGACAAGCCCGTCAGGGCGCGTCAGCGGGTGTTGGCGGG 8550
TGCCAGTGTCGAACAGACATTCGCCTACGGCCCTCGTCTGTTCGGGCAGTCCCGCGCAGTCGCCCACAACCGCCC

NdeI

TGTCGGGGCTGGCTTAACTATGCGGCATCAGAGCAGATTGTACTGAGAGTGCACCATATGCGGTGTGAAATACCG 8625
ACAGCCCCGACCGAATTGATACGCCGTAGTCTCGTCTAACATGACTCTCACGTGGTATACGCCACACTTTATGGC

KasI **NarI** **SfoI** **PluTI**

CACAGATGCGTAAGGAGAAAATACCGCATCAGGCGCCATTCGCCATTCAGGCTGCGCAACTGTTGGGAAGGGCGA 8700
GTGTCTACGCATTCCTCTTTTATGGCGTAGTCCGCGGTAAGCGGTAAGTCCGACGCGTTGACAACCCTTCCCGCT

```
TCGGTGCGGGCCTCTTCGCTATTACGCCAGCTGGCGAAAGGGGGATGTGCTGCAAGGCGATTAAGTTGGGTAACG
                                                                                8775
AGCCACGCCCGGAGAAGCGATAATGCGGTCGACCGCTTTCCCCCTACACGACGTTCCGCTAATTCAACCCATTGC

CCAGGGTTTTCCCAGTCACGACGTTGTAAAACGACGGCCAGTGAATTG    3′
                                                ...   8823
GGTCCCAAAAGGGTCAGTGCTGCAACATTTTGCTGCCGGTCACTTAAC    5′
```
