# Supplementary material for: An alternative for proteinase K-heat-sensitive protease from fungus Onygena corvina for biotechnology: cloning, engineering, expression, characterization and special application for protein sequencing
Source: Microb Cell Fact. 2020 Jun 24;19:135. doi: 10.1186/s12934-020-01392-3 (PMC7313183; doi:10.1186/s12934-020-01392-3)
Supplement: Supplementary file 13 — Additional file 13. MASCOT Search Results of mature NHSSP. [file 12934_2020_1392_MOESM13_ESM.pdf]

MASCOT Search Results

Protein View: 349

Prot-1 [agafab 20160316]

**Database:**	small-www
**Score:**	9207
**Nominal mass (M_r):**	39892
**Calculated pI:**	7.90

Sequence similarity is available as an NCBI BLAST search of 349 against nr.

Search parameters

**MS data file:**	\\212.87.29.88\orbita\FA\03-marzec2016\60324460bro_Prot-1.raw
**Enzyme:**	Trypsin: cuts C-term side of KR unless next residue is P.
**Fixed modifications:**	Methylthio (C)
**Variable modifications:**	Oxidation (M)

Protein sequence coverage: 35%

Matched peptides shown in ***bold red***.

```
  1 RAFFHNKGGN DIIPNSYIVV MKDGISTQEF ESHISSVSAT HAKRSTELVG
 51 HKDSFNINGW RAYNGHFDAA TLESILNDDN VKYVEHDRVV KISALTTQPN
101 APSWGLGRIS HRSPGNKDFV YDDTAGQGIT IYGVDTGIDI NHPDFGGRAR
151 WGTNTVDNAN NDGHGHGTHT AGTFAGNAYG IAKKASVVAV KVLSASGSGS
201 NAGVIKGIDW CVTDARSKGA LGKAALNLSL GGGFNQATND AVTRAQTAGI
251 FVAVAAGNDN KDARNYSPAS APAVCTVASS TIDDQKSSFS NWGSIVDIYA
301 PGSNIISDAP GGGVRTMSGT SMASPHVCGA GAAMLAQGVP VGQVCDRLKQ
351 IGNAVVRNPG TSTTNRLLYN GSGQGSHHHH HH
```

Unformatted sequence string: 382 residues (for pasting into other applications).

Sort peptides by ◉ Residue Number ○ Increasing Mass ○ Decreasing Mass

Show predicted peptides also

Query	Start	-	End	Observed	Mr(expt)	Mr(calc)	ppm	M	Score	Expect	Rank	U	Peptide
4310	109	-	117	498.2726	994.5306	994.5308	-0.16		177	2.1e-08	1 Score > 13 indicates identity	U	R.ISHRSPGNK.D
4311	109	-	117	332.5175	994.5307	994.5308	-0.14		137	0.00021	1 Score > 13 indicates identity	U	R.ISHRSPGNK.D

Query	Start	-	End	Observed	Mr(expt)	Mr(calc)	ppm	M	Score	Expect	Rank	U	Peptide
4312	109	-	117	332.5175	994.5307	994.5308	-0.14	1	38	0.00014	1 Score **> 13** indicates **identity**	U	R.ISHRSPGNK.D
4313	109	-	117	332.5175	994.5307	994.5308	-0.14	1	38	0.00017	1 Score **> 13** indicates **identity**	U	R.ISHRSPGNK.D
4314	109	-	117	498.2728	994.5311	994.5308	0.30	1	81	7.9e-09	1 Score **> 13** indicates **identity**	U	R.ISHRSPGNK.D
4315	109	-	117	498.2731	994.5317	994.5308	0.89	1	71	7.3e-08	1 Score **> 13** indicates **identity**	U	R.ISHRSPGNK.D
24868	151	-	183	832.3790	3325.4869	3325.4886	-0.50	0	49	1.4e-05	1 Score **> 13** indicates **identity**	U	R.WGTNTVDNANNDGHGHGTHTAGTFAGNAYGIAK.K

Query	Start	-	End	Observed	Mr(expt)	Mr(calc)	ppm	M	Score	Expect	Rank	U	Peptide
24870	151	-	183	832.3800	3325.4909	3325.4886	0.72	0	47	2.1e-05	1Score **> 13** indicates **identity**	U	R.WGTNTVDNANNDGHGHGTHTAGTFAGNAYGIAK.K
24871	151	-	183	832.3801	3325.4915	3325.4886	0.88	0	45	2.9e-05	1Score **> 13** indicates **identity**	U	R.WGTNTVDNANNDGHGHGTHTAGTFAGNAYGIAK.K
24873	151	-	183	832.3803	3325.4923	3325.4886	1.11	0	44	4.4e-05	1Score **> 13** indicates **identity**	U	R.WGTNTVDNANNDGHGHGTHTAGTFAGNAYGIAK.K
24874	151	-	183	832.3809	3325.4947	3325.4886	1.83	0	56	2.8e-06	1Score **> 13** indicates **identity**	U	R.WGTNTVDNANNDGHGHGTHTAGTFAGNAYGIAK.K
24875	151	-	183	832.3811	3325.4952	3325.4886	1.99	0	42	7.1e-05	1Score **> 13** indicates **identity**	U	R.WGTNTVDNANNDGHGHGTHTAGTFAGNAYGIAK.K

Query	Start	-	End	Observed	Mr(expt)	Mr(calc)	ppm	M	Score	Expect	Rank	U	Peptide
1519	184	-	191	401.2636	800.5127	800.5120	0.86	1	86	2.6e-09	1Score **> 13** indicates **identity**	U	K.KASVVAVK.V
9860	192	-	206	673.8683	1345.7220	1345.7201	1.37	0	148	1.5e-15	1Score **> 13** indicates **identity**	U	K.VLSASGSGSNAGVIK.G
9861	192	-	206	673.8685	1345.7224	1345.7201	1.69	0	126	2.3e-13	1Score **> 13** indicates **identity**	U	K.VLSASGSGSNAGVIK.G
19039	224	-	244	697.3573	2089.0500	2089.0552	-2.49	0	42	6.8e-05	1Score **> 13** indicates **identity**	U	K.AALNLSLGGGFNQATNDAVTR.A
19040	224	-	244	697.3576	2089.0509	2089.0552	-2.09	0	38	0.00016	1Score **> 13** indicates **identity**	U	K.AALNLSLGGGFNQATNDAVTR.A

Query	Start	–	End	Observed	Mr(expt)	Mr(calc)	ppm	M	Score	Expect	Rank	U	Peptide
19042	224	–	244	1045.5330	2089.0514	2089.0552	-1.81	0	117	2.1e-12	1	**Score > 13** indicates **identity**	UK.AALNLSLGGGFNQATNDAVTR.A
19043	224	–	244	1045.5334	2089.0522	2089.0552	-1.42	0	112	6.1e-12	1	**Score > 13** indicates **identity**	UK.AALNLSLGGGFNQATNDAVTR.A
19044	224	–	244	697.3585	2089.0537	2089.0552	-0.71	0	32	0.00063	1	**Score > 13** indicates **identity**	UK.AALNLSLGGGFNQATNDAVTR.A
19045	224	–	244	697.3589	2089.0549	2089.0552	-0.14	0	112	7e-12	1	**Score > 13** indicates **identity**	UK.AALNLSLGGGFNQATNDAVTR.A
19046	224	–	244	1045.5348	2089.0550	2089.0552	-0.083	0	107	2.1e-11	1	**Score > 13** indicates **identity**	UK.AALNLSLGGGFNQATNDAVTR.A

Query	Start	–	End	Observed	Mr(expt)	Mr(calc)	ppm	M	Score	Expect	Rank	U	Peptide
19047	224	–	244	697.3591	2089.0556	2089.0552	0.16	0	122	6.2e-13	1 Score **> 13** indicates **identity**	U	K.AALNLSLGGGFNQATNDAVTR.A
19048	224	–	244	1045.5351	2089.0556	2089.0552	0.20	0	180	1e-18	1 Score **> 13** indicates **identity**	U	K.AALNLSLGGGFNQATNDAVTR.A
19049	224	–	244	697.3592	2089.0559	2089.0552	0.31	0	117	1.8e-12	1 Score **> 13** indicates **identity**	U	K.AALNLSLGGGFNQATNDAVTR.A
19050	224	–	244	697.3593	2089.0562	2089.0552	0.46	0	82	6.1e-09	1 Score **> 13** indicates **identity**	U	K.AALNLSLGGGFNQATNDAVTR.A
19051	224	–	244	1045.5354	2089.0562	2089.0552	0.49	0	110	1.1e-11	1 Score **> 13** indicates **identity**	U	K.AALNLSLGGGFNQATNDAVTR.A

Query	Start	–	End	Observed	Mr(expt)	Mr(calc)	ppm	M	Score	Expect	Rank	U	Peptide
19052	224	–	244	697.3595	2089.0565	2089.0552	0.62	0	109	1.4e-11	1 **Score > 13** indicates **identity**	U	K.AALNLSLGGGFNQATNDAVTR.A
19053	224	–	244	1045.5356	2089.0566	2089.0552	0.68	0	180	9.5e-19	1 **Score > 13** indicates **identity**	U	K.AALNLSLGGGFNQATNDAVTR.A
19054	224	–	244	697.3595	2089.0567	2089.0552	0.69	0	56	2.6e-06	1 **Score > 13** indicates **identity**	U	K.AALNLSLGGGFNQATNDAVTR.A
19055	224	–	244	1045.5358	2089.0570	2089.0552	0.87	0	180	1e-18	1 **Score > 13** indicates **identity**	U	K.AALNLSLGGGFNQATNDAVTR.A
19056	224	–	244	697.3599	2089.0577	2089.0552	1.20	0	108	1.7e-11	1 **Score > 13** indicates **identity**	U	K.AALNLSLGGGFNQATNDAVTR.A

Query	Start	-	End	Observed	Mr(expt)	Mr(calc)	ppm	M	Score	Expect	Rank	U	Peptide
19057	224	-	244	697.3599	2089.0578	2089.0552	1.21	0	119	1.1e-12	1Score **> 13** indicates **identity**		UK.AALNLSLGGGFNQATNDAVTR.A
19058	224	-	244	697.3599	2089.0578	2089.0552	1.25	0	107	2.2e-11	1Score **> 13** indicates **identity**		UK.AALNLSLGGGFNQATNDAVTR.A
19059	224	-	244	1045.5362	2089.0578	2089.0552	1.26	0	176	2.6e-18	1Score **> 13** indicates **identity**		UK.AALNLSLGGGFNQATNDAVTR.A
19060	224	-	244	697.3599	2089.0580	2089.0552	1.33	0	121	7.5e-13	1Score **> 13** indicates **identity**		UK.AALNLSLGGGFNQATNDAVTR.A
19061	224	-	244	1045.5363	2089.0580	2089.0552	1.35	0	89	1.2e-09	1Score **> 13** indicates **identity**		UK.AALNLSLGGGFNQATNDAVTR.A

Query	Start	-	End	Observed	Mr(expt)	Mr(calc)	ppm	M	Score	Expect	Rank	U	Peptide
19062	224	-	244	1045.5363	2089.0580	2089.0552	1.35	0	160	9.7e-17	1 Score **> 13** indicates **identity**	U	K.AALNLSLGGGFNQATNDAVTR.A
19063	224	-	244	1045.5364	2089.0582	2089.0552	1.45	0	111	8.6e-12	1 Score **> 13** indicates **identity**	U	K.AALNLSLGGGFNQATNDAVTR.A
19064	224	-	244	697.3601	2089.0583	2089.0552	1.48	0	114	3.7e-12	1 Score **> 13** indicates **identity**	U	K.AALNLSLGGGFNQATNDAVTR.A
19065	224	-	244	697.3601	2089.0585	2089.0552	1.56	0	116	2.4e-12	1 Score **> 13** indicates **identity**	U	K.AALNLSLGGGFNQATNDAVTR.A
19066	224	-	244	697.3602	2089.0588	2089.0552	1.71	0	116	2.3e-12	1 Score **> 13** indicates **identity**	U	K.AALNLSLGGGFNQATNDAVTR.A

Query	Start	-	End	Observed	Mr(expt)	Mr(calc)	ppm	M	Score	Expect	Rank	U	Peptide
19067	224	-	244	697.3602	2089.0589	2089.0552	1.77	0	111	7.4e-12	1 Score **> 13** indicates **identity**		UK.AALNLSLGGGFNQATNDAVTR.A
19068	224	-	244	1045.5368	2089.0590	2089.0552	1.83	0	52	6.7e-06	1 Score **> 13** indicates **identity**		UK.AALNLSLGGGFNQATNDAVTR.A
19069	224	-	244	1045.5368	2089.0590	2089.0552	1.83	0	180	1e-18	1 Score **> 13** indicates **identity**		UK.AALNLSLGGGFNQATNDAVTR.A
19071	224	-	244	697.3605	2089.0596	2089.0552	2.07	0	40	9.3e-05	1 Score **> 13** indicates **identity**		UK.AALNLSLGGGFNQATNDAVTR.A
19072	224	-	244	1045.5371	2089.0596	2089.0552	2.12	0	161	8.7e-17	1 Score **> 13** indicates **identity**		UK.AALNLSLGGGFNQATNDAVTR.A

Query	Start	-	End	Observed	Mr(expt)	Mr(calc)	ppm	M	Score	Expect	Rank	U	Peptide
19073	224	-	244	1045.5375	2089.0604	2089.0552	2.50	0	170	1.1e-17	1	Score **> 13** indicates **identity**	UK.AALNLSLGGGFNQATNDAVTR.A
19074	224	-	244	1045.5375	2089.0604	2089.0552	2.50	0	176	2.6e-18	1	Score **> 13** indicates **identity**	UK.AALNLSLGGGFNQATNDAVTR.A
19075	224	-	244	1045.5375	2089.0604	2089.0552	2.50	0	164	4.4e-17	1	Score **> 13** indicates **identity**	UK.AALNLSLGGGFNQATNDAVTR.A
19076	224	-	244	697.3608	2089.0605	2089.0552	2.55	0	102	6.4e-11	1	Score **> 13** indicates **identity**	UK.AALNLSLGGGFNQATNDAVTR.A
19077	224	-	244	697.3608	2089.0605	2089.0552	2.55	0	95	3.4e-10	1	Score **> 13** indicates **identity**	UK.AALNLSLGGGFNQATNDAVTR.A

Query	Start	–	End	Observed	Mr(expt)	Mr(calc)	ppm	M	Score	Expect	Rank	U	Peptide
19078	224	–	244	1045.5376	2089.0606	2089.0552	2.60	0	104	3.7e-11	1Score **> 13** indicates **identity**	U	K.AALNLSLGGGFNQATNDAVTR.A
19079	224	–	244	1045.5378	2089.0610	2089.0552	2.79	0	173	4.9e-18	1Score **> 13** indicates **identity**	U	K.AALNLSLGGGFNQATNDAVTR.A
19080	224	–	244	697.3610	2089.0612	2089.0552	2.86	0	72	6.5e-08	1Score **> 13** indicates **identity**	U	K.AALNLSLGGGFNQATNDAVTR.A
19081	224	–	244	697.3610	2089.0613	2089.0552	2.89	0	109	1.2e-11	1Score **> 13** indicates **identity**	U	K.AALNLSLGGGFNQATNDAVTR.A
19082	224	–	244	697.3611	2089.0614	2089.0552	2.95	0	98	1.4e-10	1Score **> 13** indicates **identity**	U	K.AALNLSLGGGFNQATNDAVTR.A

Query	Start	-	End	Observed	Mr(expt)	Mr(calc)	ppm	M	Score	Expect	Rank	U	Peptide
19083	224	-	244	1045.5381	2089.0616	2089.0552	3.08	0	168	1.4e-17	1 Score **> 13** indicates **identity**		UK.AALNLSLGGGFNQATNDAVTR.A
19084	224	-	244	1045.5385	2089.0624	2089.0552	3.46	0	85	3.5e-09	1 Score **> 13** indicates **identity**		UK.AALNLSLGGGFNQATNDAVTR.A
19085	224	-	244	1045.5385	2089.0624	2089.0552	3.46	0	114	4.4e-12	1 Score **> 13** indicates **identity**		UK.AALNLSLGGGFNQATNDAVTR.A
19086	224	-	244	697.3615	2089.0626	2089.0552	3.52	0	127	1.9e-13	1 Score **> 13** indicates **identity**		UK.AALNLSLGGGFNQATNDAVTR.A
19087	224	-	244	1045.5386	2089.0626	2089.0552	3.55	0	159	1.2e-16	1 Score **> 13** indicates **identity**		UK.AALNLSLGGGFNQATNDAVTR.A

Query	Start	–	End	Observed	Mr(expt)	Mr(calc)	ppm	M	Score	Expect	Rank	U	Peptide
19088	224	–	244	697.3617	2089.0632	2089.0552	3.80	0	95	3.4e-10	1 Score **> 13** indicates **identity**	U	K.AALNLSLGGGFNQATNDAVTR.A
19089	224	–	244	1045.5389	2089.0632	2089.0552	3.84	0	161	8.5e-17	1 Score **> 13** indicates **identity**	U	K.AALNLSLGGGFNQATNDAVTR.A
19090	224	–	244	697.3618	2089.0635	2089.0552	3.95	0	105	3.4e-11	1 Score **> 13** indicates **identity**	U	K.AALNLSLGGGFNQATNDAVTR.A
19091	224	–	244	697.3619	2089.0638	2089.0552	4.10	0	90	9.3e-10	1 Score **> 13** indicates **identity**	U	K.AALNLSLGGGFNQATNDAVTR.A
19092	224	–	244	1045.5397	2089.0648	2089.0552	4.61	0	152	6.3e-16	1 Score **> 13** indicates **identity**	U	K.AALNLSLGGGFNQATNDAVTR.A

Query	Start	-	End	Observed	Mr(expt)	Mr(calc)	ppm	M	Score	Expect	Rank	U	Peptide
19093	224	-	244	1045.5404	2089.0662	2089.0552	5.28	0	170	9.3e-18	1Score **> 13** indicates **identity**	U	K.AALNLSLGGGFNQATNDAVTR.A
19094	224	-	244	697.3632	2089.0676	2089.0552	5.94	0	82	6.3e-09	1Score **> 13** indicates **identity**	U	K.AALNLSLGGGFNQATNDAVTR.A
19095	224	-	244	1045.5431	2089.0716	2089.0552	7.86	0	142	6.6e-15	1Score **> 13** indicates **identity**	U	K.AALNLSLGGGFNQATNDAVTR.A
19096	224	-	244	1045.5449	2089.0752	2089.0552	9.59	0	135	3.3e-14	1Score **> 13** indicates **identity**	U	K.AALNLSLGGGFNQATNDAVTR.A
23711	287	-	315	1462.2128	2922.4110	2922.4148	-1.29	0	148	1.7e-15	1Score **> 13** indicates **identity**	U	K.SSFSNWGSIVDIYAPGSNIISDAPGGGVR.T

Query	Start	–	End	Observed	Mr(expt)	Mr(calc)	ppm	M	Score	Expect	Rank		U	Peptide
23712	287	–	315	1462.2134	2922.4122	2922.4148	-0.88	0	137	2.2e-14	1	Score **> 13** indicates **identity**	U	K.SSFSNWGSIVDIYAPGSNIISDAPGGGVR.T
23713	287	–	315	1462.2151	2922.4156	2922.4148	0.29	0	127	1.9e-13	1	Score **> 13** indicates **identity**	U	K.SSFSNWGSIVDIYAPGSNIISDAPGGGVR.T
23714	287	–	315	975.1468	2922.4187	2922.4148	1.33	0	128	1.6e-13	1	Score **> 13** indicates **identity**	U	K.SSFSNWGSIVDIYAPGSNIISDAPGGGVR.T
6304	348	–	357	549.3426	1096.6707	1096.6717	-0.85	1	77	2.2e-08	1	Score **> 13** indicates **identity**	U	R.LKQIGNAVVR.N
6305	348	–	357	366.5642	1096.6707	1096.6717	-0.84	1	94	4e-10	1	Score **> 13** indicates **identity**	U	R.LKQIGNAVVR.N

Query	Start	–	End	Observed	Mr(expt)	Mr(calc)	ppm	M	Score	Expect	Rank	U	Peptide
6306	348	–	357	549.3427	1096.6707	1096.6717	-0.83		174	3.9e-08	1Score **> 13** indicates **identity**	U	R.LKQIGNAVVR.N
6307	348	–	357	366.5642	1096.6709	1096.6717	-0.73		194	4e-10	1Score **> 13** indicates **identity**	U	R.LKQIGNAVVR.N
6308	348	–	357	549.3427	1096.6709	1096.6717	-0.67		177	2.2e-08	1Score **> 13** indicates **identity**	U	R.LKQIGNAVVR.N
6309	348	–	357	366.5643	1096.6712	1096.6717	-0.46		194	3.9e-10	1Score **> 13** indicates **identity**	U	R.LKQIGNAVVR.N
6310	348	–	357	366.5643	1096.6712	1096.6717	-0.43		194	3.9e-10	1Score **> 13** indicates **identity**	U	R.LKQIGNAVVR.N

Query	Start	–	End	Observed	Mr(expt)	Mr(calc)	ppm	M	Score	Expect	Rank	U	Peptide
6311	348	–	357	366.5644	1096.6713	1096.6717	-0.37		194	3.9e-10	1Score **> 13** indicates **identity**	U	R.LKQIGNAVVR.N
6312	348	–	357	549.3430	1096.6715	1096.6717	-0.12		174	3.8e-08	1Score **> 13** indicates **identity**	U	R.LKQIGNAVVR.N
6313	348	–	357	549.3431	1096.6717	1096.6717	0.0064		176	2.3e-08	1Score **> 13** indicates **identity**	U	R.LKQIGNAVVR.N
6314	348	–	357	549.3431	1096.6717	1096.6717	0.061		177	2.2e-08	1Score **> 13** indicates **identity**	U	R.LKQIGNAVVR.N
6315	348	–	357	366.5646	1096.6721	1096.6717	0.37		194	3.9e-10	1Score **> 13** indicates **identity**	U	R.LKQIGNAVVR.N

Query	Start	-	End	Observed	Mr(expt)	Mr(calc)	ppm	M	Score	Expect	Rank	U	Peptide
6316	348	-	357	549.3433	1096.6721	1096.6717	0.44		175	2.8e-08	1Score **> 13** indicates **identity**	U	R.LKQIGNAVVR.N
6317	348	-	357	366.5647	1096.6723	1096.6717	0.61		194	4e-10	1Score **> 13** indicates **identity**	U	R.LKQIGNAVVR.N
6318	348	-	357	549.3436	1096.6725	1096.6717	0.81		174	3.8e-08	1Score **> 13** indicates **identity**	U	R.LKQIGNAVVR.N
6319	348	-	357	549.3437	1096.6729	1096.6717	1.12		174	4.2e-08	1Score **> 13** indicates **identity**	U	R.LKQIGNAVVR.N
6320	348	-	357	549.3439	1096.6733	1096.6717	1.47		166	2.8e-07	1Score **> 13** indicates **identity**	U	R.LKQIGNAVVR.N

Query	Start	-	End	Observed	Mr(expt)	Mr(calc)	ppm	M	Score	Expect	Rank	U	Peptide
6322	348	-	357	549.3439	1096.6733	1096.6717	1.54	1	74	4.3e-08	1Score **> 13** indicates **identity**	U	R.LKQIGNAVVR.N
6323	348	-	357	549.3440	1096.6735	1096.6717	1.65	1	68	1.5e-07	1Score **> 13** indicates **identity**	U	R.LKQIGNAVVR.N
6324	348	-	357	549.3443	1096.6741	1096.6717	2.19	1	55	3.4e-06	1Score **> 13** indicates **identity**	U	R.LKQIGNAVVR.N
6325	348	-	357	549.3448	1096.6750	1096.6717	3.09	1	49	1.1e-05	1Score **> 13** indicates **identity**	U	R.LKQIGNAVVR.N
2210	350	-	357	428.7521	855.4897	855.4926	-3.46	0	56	2.5e-06	1Score **> 13** indicates **identity**	U	K.QIGNAVVR.N

Query	Start	-	End	Observed	Mr(expt)	Mr(calc)	ppm	M	Score	Expect	Rank	U	Peptide
2213	350	-	357	428.7525	855.4904	855.4926	-2.66	0	56	2.5e-06	1Score **> 13** indicates **identity**		UK.QIGNAVVR.N
2214	350	-	357	428.7525	855.4904	855.4926	-2.59	0	56	2.5e-06	1Score **> 13** indicates **identity**		UK.QIGNAVVR.N
2215	350	-	357	428.7525	855.4905	855.4926	-2.45	0	56	2.5e-06	1Score **> 13** indicates **identity**		UK.QIGNAVVR.N
2217	350	-	357	428.7527	855.4908	855.4926	-2.10	0	51	8.6e-06	1Score **> 13** indicates **identity**		UK.QIGNAVVR.N
2218	350	-	357	428.7529	855.4911	855.4926	-1.75	0	56	2.6e-06	1Score **> 13** indicates **identity**		UK.QIGNAVVR.N

Query	Start	-	End	Observed	Mr(expt)	Mr(calc)	ppm	M	Score	Expect	Rank	U	Peptide
2219	350	–	357	428.7529	855.4912	855.4926	-1.70	0	56	2.6e-06	1Score **> 13** indicates **identity**	U	K.QIGNAVVR.N
2222	350	–	357	428.7530	855.4914	855.4926	-1.42	0	50	9e-06	1Score **> 13** indicates **identity**	U	K.QIGNAVVR.N
2223	350	–	357	428.7530	855.4915	855.4926	-1.35	0	58	1.5e-06	1Score **> 13** indicates **identity**	U	K.QIGNAVVR.N
2224	350	–	357	428.7530	855.4915	855.4926	-1.31	0	58	1.7e-06	1Score **> 13** indicates **identity**	U	K.QIGNAVVR.N
2225	350	–	357	428.7531	855.4917	855.4926	-1.12	0	56	2.6e-06	1Score **> 13** indicates **identity**	U	K.QIGNAVVR.N

Query	Start	-	End	Observed	Mr(expt)	Mr(calc)	ppm	M	Score	Expect	Rank	U	Peptide
2226	350	-	357	428.7534	855.4921	855.4926	-0.58	0	58	1.7e-06	1 Score **> 13** indicates **identity**	U	K.QIGNAVVR.N
2227	350	-	357	428.7534	855.4923	855.4926	-0.37	0	53	4.9e-06	1 Score **> 13** indicates **identity**	U	K.QIGNAVVR.N
2228	350	-	357	428.7535	855.4924	855.4926	-0.23	0	58	1.5e-06	1 Score **> 13** indicates **identity**	U	K.QIGNAVVR.N
16290	350	-	366	595.6517	1783.9332	1783.9289	2.41	1	65	3.2e-07	1 Score **> 13** indicates **identity**	U	K.QIGNAVVRNPGTSTTNR.L

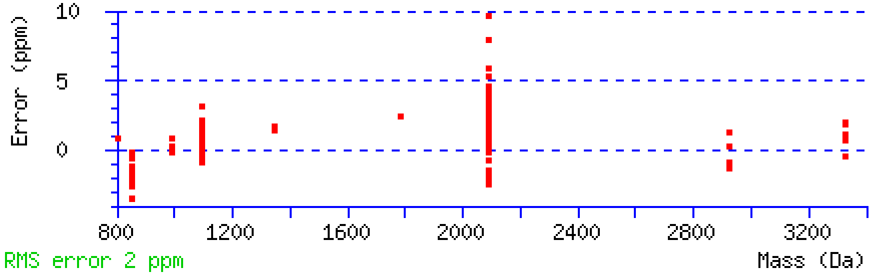

Mascot: http://www.matrixscience.com/
