# Supplementary material for: An alternative for proteinase K-heat-sensitive protease from fungus Onygena corvina for biotechnology: cloning, engineering, expression, characterization and special application for protein sequencing
Source: Microb Cell Fact. 2020 Jun 24;19:135. doi: 10.1186/s12934-020-01392-3 (PMC7313183; doi:10.1186/s12934-020-01392-3)
Supplement: Supplementary file 14 — Additional file 14. N- and C-terminal sequencing results of mature NHSSP. [file 12934_2020_1392_MOESM14_ESM.pdf]

Applied Biosystems 4700 Proteomics Analyzer 430510

4700 Reflector Spec #1 MC[BP = 1568.8, 3009]

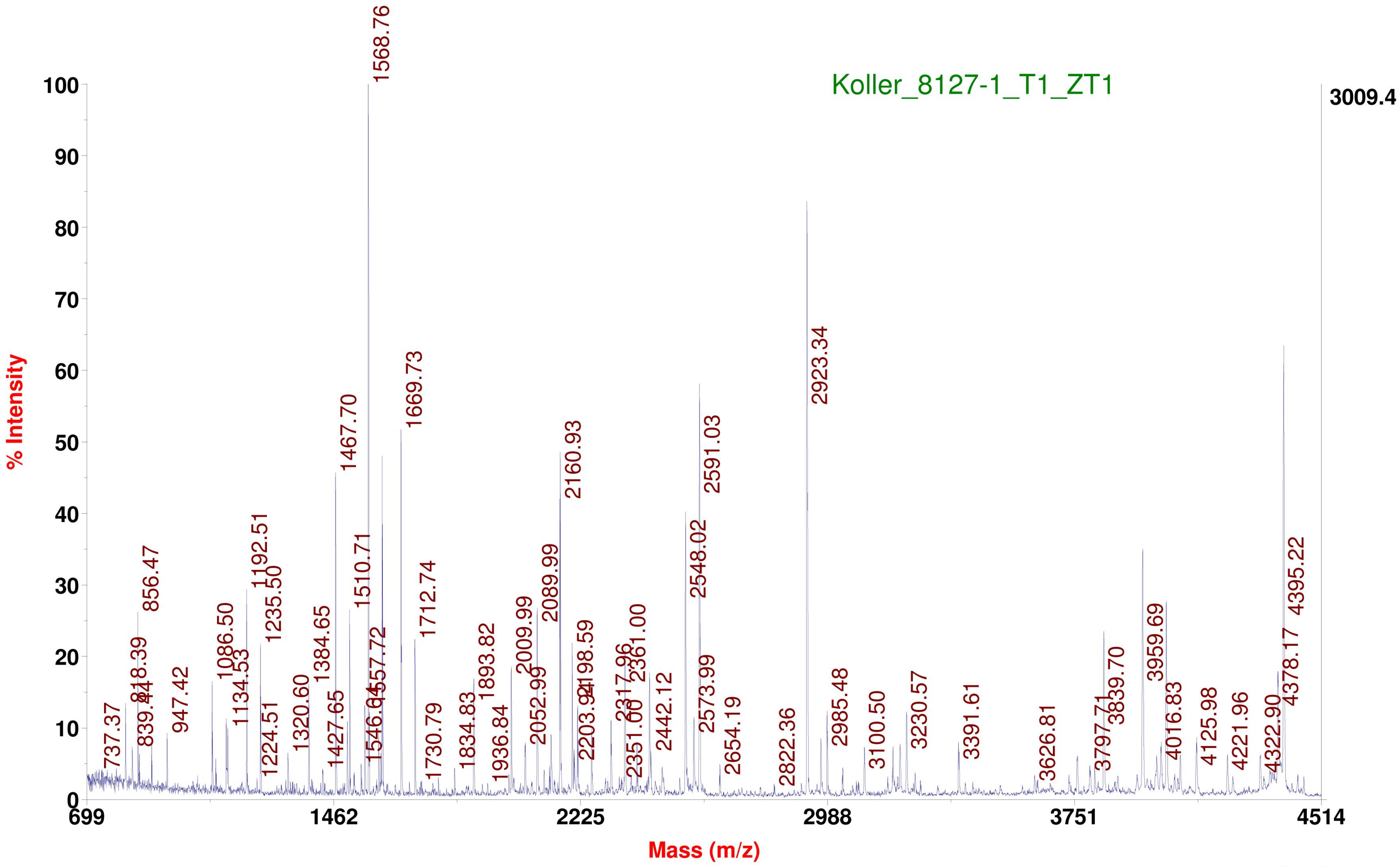

\\server3\User\Kunden_Projekte\Koller_8127\Kunden_2016\Div_160818\P9_MS_1.t2d

Printed: 10:24, November 30, 2016

red arrow - N - terminal peptide

blue arrow - C - terminal peptide

MATRIX SCIENCE Mascot Search Results

User :
Email :
Search title : **P9_MS_1.t2d - Koller_8127-1_T1_ZT1**
Database : **Steffi 1 (14 sequences; 4130 residues)**
Timestamp : **1 Dec 2016 at 07:48:00 GMT**
Top Score : **79 for Protease_1_94-372,**

Probability Based Mowse Score

Ions score is -10*Log(P), where P is the probability that the observed match is a random event.
Protein scores greater than 24 are significant (p<0.05).

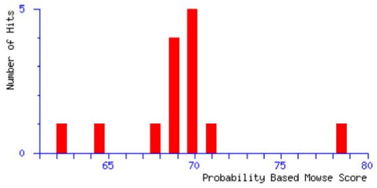

Protein Summary Report

Format As | Protein Summary | Help

Significance threshold p< 0.05 Max. number of hits AUTO

Re-Search All | Search Unmatched

Index

	Accession	Mass	Score	Description
1.	Protease 1 94-372	28361	79	
2.	Protease 1 94-366	27714	70	
3.	Protease 1 94-367	27827	70	
4.	Protease 1 94-368	27940	70	
5.	Protease 1 94-369	28103	70	
6.	Protease 1 94-370	28217	70	
7.	Protease 1 94-371	28274	70	
8.	Protease 1 94-373	28418	69	
9.	Protease 1 94-374	28546	69	
10.	Protease 1 94-375	28603	69	
11.	Protease 1 94-376	28690	69	
12.	Protease 1 94-382	29512	68	
13.	Protease 1 full length 1-382	39937	64	
14.	Protease 1 full length plus Signal peptide	41853	62	

Results List

1. Protease 1 94-372 **Mass:** 28361 **Score:** **79** **Expect:** 2e-007 **Queries matched:** 12

Observed	Mr(expt)	Mr(calc)	Delta	Start	End	Miss	Peptide
856.4697	855.4624	855.4926	-0.0302	257 -	264	0	K.QIGNAVVR.N
947.4228	946.4155	946.4468	-0.0313	265 -	273	0	R.NPGTSTTNR.L
1097.6431	1096.6358	1096.6716	-0.0358	255 -	264	1	R.LKQIGNAVVR.N
1192.5066	1191.4993	1191.5342	-0.0349	114 -	123	0	K.GIDWCVTDAR.S
1346.6834	1345.6761	1345.7201	-0.0440	99 -	113	0	K.VLSASGSGSNAGVIK.G
1568.7599	1567.7526	1567.8107	-0.0580	1 -	15	0	-.ALTTQPNAPSWGLGR.I
1594.6986	1593.6913	1593.7747	-0.0833	265 -	279	1	R.NPGTSTTNRLLYNGS.-
1784.8785	1783.8713	1783.9288	-0.0576	257 -	273	1	K.QIGNAVVRNPGTSTTNR.L
2089.9856	2088.9783	2089.0551	-0.0768	131 -	151	0	K.AALNLSLGGGFNQATNDAVTR.A
2923.3357	2922.3284	2922.4147	-0.0863	194 -	222	0	K.SSFSNWGSIVDIYAPGSNIISDAPGGGVR.T
3203.3560	3202.3487	3202.4449	-0.0962	223 -	254	0	R.TMSGTSMASPHVCGAGAAMLAQGVPVGQVCDR.L
3811.7322	3810.7249	3810.7760	-0.0511	20 -	55	1	R.SPGNKDFVYDDTAGQGITIYGVDTGIDINHPDFGGR.A

No match to: 724.4434, 733.4623, 737.3729, 744.4733, 746.4565, 749.4891, 755.4316, 760.4791, 762.5109, 768.4706, 786.5005, 790.5098, 798.4902, 818.3948, 839.4433, 858.4949, 859.4981, 861.3915, 899.4702, 1086.5004, 1129.5076, 1132.5085, 1133.4841, 1134.5315, 1224.5101, 1235.5029, 1313.6464, 1320.5973, 1334.6139, 1384.6472, 1393.6626, 1427.6527, 1433.7258, 1467.7009, 1503.6478, 1510.7076, 1524.6400, 1546.6444, 1557.7217, 1600.7336, 1605.7229, 1607.7161, 1611.7679, 1669.7307, 1712.7358, 1730.7920, 1767.8136, 1834.8348, 1893.8197, 1936.8419, 2002.8632, 2009.9857, 2052.9927, 2110.9190, 2127.9177, 2132.9998, 2142.9309, 2160.9346, 2198.0662, 2198.5911, 2203.9448, 2215.0576, 2258.0129, 2317.9634, 2351.0039, 2359.0432, 2360.9961, 2380.0496, 2398.1003, 2433.0029, 2436.9856, 2441.0759, 2442.1169, 2476.0034, 2547.0313, 2548.0212, 2553.1460, 2573.9905, 2591.0327, 2654.1919, 2822.3552, 2922.3638, 2966.3379, 2985.4771, 3033.4314, 3076.4116, 3083.3225, 3100.4958, 3173.6069, 3188.4954, 3210.4119, 3230.5667, 3253.4275, 3257.3911, 3274.5586, 3391.6094, 3626.8101, 3634.5938, 3733.6704, 3757.6819, 3797.7144, 3839.6951, 3959.6892, 4000.7944, 4005.7834, 4016.8291, 4032.7107, 4075.7166, 4125.9771, 4221.9639, 4322.8979, 4377.2358, 4378.1675, 4395.2231

2. Protease 1 94-366 **Mass:** 27714 **Score:** **70** **Expect:** 1.3e-006 **Queries matched:** 11

Observed	Mr(expt)	Mr(calc)	Delta	Start	End	Miss	Peptide
856.4697	855.4624	855.4926	-0.0302	257 -	264	0	K.QIGNAVVR.N
947.4228	946.4155	946.4468	-0.0313	265 -	273	0	R.NPGTSTTNR.-
1097.6431	1096.6358	1096.6716	-0.0358	255 -	264	1	R.LKQIGNAVVR.N
1192.5066	1191.4993	1191.5342	-0.0349	114 -	123	0	K.GIDWCVTDAR.S
1346.6834	1345.6761	1345.7201	-0.0440	99 -	113	0	K.VLSASGSGSNAGVIK.G
1568.7599	1567.7526	1567.8107	-0.0580	1 -	15	0	-.ALTTQPNAPSWGLGR.I
1784.8785	1783.8713	1783.9288	-0.0576	257 -	273	1	K.QIGNAVVRNPGTSTTNR.-

2089.9856	2088.9783	2089.0551	-0.0768	131	151	0	K.AALNLSLGGGFNQATNDAVTR.A
2923.3357	2922.3284	2922.4147	-0.0863	194	222	0	K.SSFSNWGSIVDIYAPGSNIISDAPGGGVR.T
3203.3560	3202.3487	3202.4449	-0.0962	223	254	0	R.TMSGTSMASPHVCGAGAAMLAQGVPVGQVCDR.L
3811.7322	3810.7249	3810.7760	-0.0511	20	55	1	R.SPGNKDFVYDDTAGQGITIYGVDTGIDINHPDFGGR.A

No match to: 724.4434, 733.4623, 737.3729, 744.4733, 746.4565, 749.4891, 755.4316, 760.4791, 762.5109, 768.4706, 786.5005, 790.5098, 798.4902, 818.3948, 839.4433, 858.4949, 859.4981, 861.3915, 899.4702, 1086.5004, 1129.5076, 1132.5085, 1133.4841, 1134.5315, 1224.5101, 1235.5029, 1313.6464, 1320.5973, 1334.6139, 1384.6472, 1393.6626, 1427.6527, 1433.7258, 1467.7009, 1503.6478, 1510.7076, 1524.6400, 1546.6444, 1557.7217, 1594.6986, 1600.7336, 1605.7229, 1607.7161, 1611.7679, 1669.7307, 1712.7358, 1730.7920, 1767.8136, 1834.8348, 1893.8197, 1936.8419, 2002.8632, 2009.9857, 2052.9927, 2110.9190, 2127.9177, 2132.9998, 2142.9309, 2160.9346, 2198.0662, 2198.5911, 2203.9448, 2215.0576, 2258.0129, 2317.9634, 2351.0039, 2359.0432, 2360.9961, 2380.0496, 2398.1003, 2433.0029, 2436.9856, 2441.0759, 2442.1169, 2476.0034, 2547.0313, 2548.0212, 2553.1460, 2573.9905, 2591.0327, 2654.1919, 2822.3552, 2922.3638, 2966.3379, 2985.4771, 3033.4314, 3076.4116, 3083.3225, 3100.4958, 3173.6069, 3188.4954, 3210.4119, 3230.5667, 3253.4275, 3257.3911, 3274.5586, 3391.6094, 3626.8101, 3634.5938, 3733.6704, 3757.6819, 3797.7144, 3839.6951, 3959.6892, 4000.7944, 4005.7834, 4016.8291, 4032.7107, 4075.7166, 4125.9771, 4221.9639, 4322.8979, 4377.2358, 4378.1675, 4395.2231

3. Protease 1 94-367 **Mass:** 27827 **Score:** 70 **Expect:** 1.3e-006 **Queries matched:** 11

Observed	Mr(expt)	Mr(calc)	Delta	Start	End	Miss	Peptide
856.4697	855.4624	855.4926	-0.0302	257	264	0	K.QIGNAVVR.N
947.4228	946.4155	946.4468	-0.0313	265	273	0	R.NPGTSTTNR.L
1097.6431	1096.6358	1096.6716	-0.0358	255	264	1	R.LKQIGNAVVR.N
1192.5066	1191.4993	1191.5342	-0.0349	114	123	0	K.GIDWCVTDAR.S
1346.6834	1345.6761	1345.7201	-0.0440	99	113	0	K.VLSASGSGSNAGVIK.G
1568.7599	1567.7526	1567.8107	-0.0580	1	15	0	-.ALTTQPNAPSWGLGR.I
1784.8785	1783.8713	1783.9288	-0.0576	257	273	1	K.QIGNAVVRNPGTSTTNR.L
2089.9856	2088.9783	2089.0551	-0.0768	131	151	0	K.AALNLSLGGGFNQATNDAVTR.A
2923.3357	2922.3284	2922.4147	-0.0863	194	222	0	K.SSFSNWGSIVDIYAPGSNIISDAPGGGVR.T
3203.3560	3202.3487	3202.4449	-0.0962	223	254	0	R.TMSGTSMASPHVCGAGAAMLAQGVPVGQVCDR.L
3811.7322	3810.7249	3810.7760	-0.0511	20	55	1	R.SPGNKDFVYDDTAGQGITIYGVDTGIDINHPDFGGR.A

No match to: 724.4434, 733.4623, 737.3729, 744.4733, 746.4565, 749.4891, 755.4316, 760.4791, 762.5109, 768.4706, 786.5005, 790.5098, 798.4902, 818.3948, 839.4433, 858.4949, 859.4981, 861.3915, 899.4702, 1086.5004, 1129.5076, 1132.5085, 1133.4841, 1134.5315, 1224.5101, 1235.5029, 1313.6464, 1320.5973, 1334.6139, 1384.6472, 1393.6626, 1427.6527, 1433.7258, 1467.7009, 1503.6478, 1510.7076, 1524.6400, 1546.6444, 1557.7217, 1594.6986, 1600.7336, 1605.7229, 1607.7161, 1611.7679, 1669.7307, 1712.7358, 1730.7920, 1767.8136, 1834.8348, 1893.8197, 1936.8419, 2002.8632, 2009.9857, 2052.9927, 2110.9190, 2127.9177, 2132.9998, 2142.9309, 2160.9346, 2198.0662, 2198.5911, 2203.9448, 2215.0576, 2258.0129, 2317.9634, 2351.0039, 2359.0432, 2360.9961, 2380.0496, 2398.1003, 2433.0029, 2436.9856, 2441.0759, 2442.1169, 2476.0034, 2547.0313, 2548.0212, 2553.1460, 2573.9905, 2591.0327, 2654.1919, 2822.3552, 2922.3638, 2966.3379, 2985.4771, 3033.4314, 3076.4116, 3083.3225, 3100.4958, 3173.6069, 3188.4954, 3210.4119, 3230.5667, 3253.4275, 3257.3911, 3274.5586, 3391.6094, 3626.8101, 3634.5938, 3733.6704, 3757.6819, 3797.7144, 3839.6951, 3959.6892, 4000.7944, 4005.7834, 4016.8291, 4032.7107, 4075.7166, 4125.9771, 4221.9639, 4322.8979, 4377.2358, 4378.1675, 4395.2231

4. Protease 1 94-368 **Mass:** 27940 **Score:** 70 **Expect:** 1.4e-006 **Queries matched:** 11

Observed	Mr(expt)	Mr(calc)	Delta	Start	End	Miss	Peptide
856.4697	855.4624	855.4926	-0.0302	257	264	0	K.QIGNAVVR.N
947.4228	946.4155	946.4468	-0.0313	265	273	0	R.NPGTSTTNR.L
1097.6431	1096.6358	1096.6716	-0.0358	255	264	1	R.LKQIGNAVVR.N
1192.5066	1191.4993	1191.5342	-0.0349	114	123	0	K.GIDWCVTDAR.S
1346.6834	1345.6761	1345.7201	-0.0440	99	113	0	K.VLSASGSGSNAGVIK.G
1568.7599	1567.7526	1567.8107	-0.0580	1	15	0	-.ALTTQPNAPSWGLGR.I
1784.8785	1783.8713	1783.9288	-0.0576	257	273	1	K.QIGNAVVRNPGTSTTNR.L
2089.9856	2088.9783	2089.0551	-0.0768	131	151	0	K.AALNLSLGGGFNQATNDAVTR.A
2923.3357	2922.3284	2922.4147	-0.0863	194	222	0	K.SSFSNWGSIVDIYAPGSNIISDAPGGGVR.T
3203.3560	3202.3487	3202.4449	-0.0962	223	254	0	R.TMSGTSMASPHVCGAGAAMLAQGVPVGQVCDR.L
3811.7322	3810.7249	3810.7760	-0.0511	20	55	1	R.SPGNKDFVYDDTAGQGITIYGVDTGIDINHPDFGGR.A

No match to: 724.4434, 733.4623, 737.3729, 744.4733, 746.4565, 749.4891, 755.4316, 760.4791, 762.5109, 768.4706, 786.5005, 790.5098, 798.4902, 818.3948, 839.4433, 858.4949, 859.4981, 861.3915, 899.4702, 1086.5004, 1129.5076, 1132.5085, 1133.4841, 1134.5315, 1224.5101, 1235.5029, 1313.6464, 1320.5973, 1334.6139, 1384.6472, 1393.6626, 1427.6527, 1433.7258, 1467.7009, 1503.6478, 1510.7076, 1524.6400, 1546.6444, 1557.7217, 1594.6986, 1600.7336, 1605.7229, 1607.7161, 1611.7679, 1669.7307, 1712.7358, 1730.7920, 1767.8136, 1834.8348, 1893.8197, 1936.8419, 2002.8632, 2009.9857, 2052.9927, 2110.9190, 2127.9177, 2132.9998, 2142.9309, 2160.9346, 2198.0662, 2198.5911, 2203.9448, 2215.0576, 2258.0129, 2317.9634, 2351.0039, 2359.0432, 2360.9961, 2380.0496, 2398.1003, 2433.0029, 2436.9856, 2441.0759, 2442.1169, 2476.0034, 2547.0313, 2548.0212, 2553.1460, 2573.9905, 2591.0327, 2654.1919, 2822.3552, 2922.3638, 2966.3379, 2985.4771, 3033.4314, 3076.4116, 3083.3225, 3100.4958, 3173.6069, 3188.4954, 3210.4119, 3230.5667, 3253.4275, 3257.3911, 3274.5586, 3391.6094, 3626.8101, 3634.5938, 3733.6704, 3757.6819, 3797.7144, 3839.6951, 3959.6892, 4000.7944, 4005.7834, 4016.8291, 4032.7107, 4075.7166, 4125.9771, 4221.9639, 4322.8979, 4377.2358, 4378.1675, 4395.2231

5. Protease 1 94-369 **Mass:** 28103 **Score:** 70 **Expect:** 1.4e-006 **Queries matched:** 11

Observed	Mr(expt)	Mr(calc)	Delta	Start	End	Miss	Peptide
856.4697	855.4624	855.4926	-0.0302	257	264	0	K.QIGNAVVR.N
947.4228	946.4155	946.4468	-0.0313	265	273	0	R.NPGTSTTNR.L
1097.6431	1096.6358	1096.6716	-0.0358	255	264	1	R.LKQIGNAVVR.N
1192.5066	1191.4993	1191.5342	-0.0349	114	123	0	K.GIDWCVTDAR.S
1346.6834	1345.6761	1345.7201	-0.0440	99	113	0	K.VLSASGSGSNAGVIK.G
1568.7599	1567.7526	1567.8107	-0.0580	1	15	0	-.ALTTQPNAPSWGLGR.I
1784.8785	1783.8713	1783.9288	-0.0576	257	273	1	K.QIGNAVVRNPGTSTTNR.L
2089.9856	2088.9783	2089.0551	-0.0768	131	151	0	K.AALNLSLGGGFNQATNDAVTR.A
2923.3357	2922.3284	2922.4147	-0.0863	194	222	0	K.SSFSNWGSIVDIYAPGSNIISDAPGGGVR.T
3203.3560	3202.3487	3202.4449	-0.0962	223	254	0	R.TMSGTSMASPHVCGAGAAMLAQGVPVGQVCDR.L
3811.7322	3810.7249	3810.7760	-0.0511	20	55	1	R.SPGNKDFVYDDTAGQGITIYGVDTGIDINHPDFGGR.A

No match to: 724.4434, 733.4623, 737.3729, 744.4733, 746.4565, 749.4891, 755.4316, 760.4791, 762.5109, 768.4706, 786.5005, 790.5098, 798.4902, 818.3948, 839.4433, 858.4949, 859.4981, 861.3915, 899.4702, 1086.5004, 1129.5076, 1132.5085, 1133.4841, 1134.5315, 1224.5101, 1235.5029, 1313.6464, 1320.5973, 1334.6139, 1384.6472, 1393.6626, 1427.6527, 1433.7258, 1467.7009, 1503.6478, 1510.7076, 1524.6400, 1546.6444, 1557.7217, 1594.6986, 1600.7336, 1605.7229, 1607.7161, 1611.7679, 1669.7307, 1712.7358, 1730.7920, 1767.8136, 1834.8348, 1893.8197, 1936.8419, 2002.8632, 2009.9857, 2052.9927, 2110.9190, 2127.9177, 2132.9998, 2142.9309, 2160.9346, 2198.0662, 2198.5911, 2203.9448, 2215.0576, 2258.0129, 2317.9634, 2351.0039, 2359.0432, 2360.9961, 2380.0496, 2398.1003, 2433.0029, 2436.9856, 2441.0759, 2442.1169, 2476.0034, 2547.0313, 2548.0212, 2553.1460, 2573.9905, 2591.0327, 2654.1919, 2822.3552, 2922.3638, 2966.3379, 2985.4771, 3033.4314, 3076.4116, 3083.3225, 3100.4958, 3173.6069, 3188.4954,

3210.4119, 3230.5667, 3253.4275, 3257.3911, 3274.5586, 3391.6094, 3626.8101, 3634.5938, 3733.6704, 3757.6819, 3797.7144, 3839.6951, 3959.6892, 4000.7944, 4005.7834, 4016.8291, 4032.7107, 4075.7166, 4125.9771, 4221.9639, 4322.8979, 4377.2358, 4378.1675, 4395.2231

6. Protease 1 94-370 **Mass:** 28217 **Score:** 70 **Expect:** 1.5e-006 **Queries matched:** 11

Observed	Mr(expt)	Mr(calc)	Delta	Start	End	Miss	Peptide
856.4697	855.4624	855.4926	-0.0302	257 -	264	0	K.QIGNAVVR.N
947.4228	946.4155	946.4468	-0.0313	265 -	273	0	R.NPGTSTTNR.L
1097.6431	1096.6358	1096.6716	-0.0358	255 -	264	1	R.LKQIGNAVVR.N
1192.5066	1191.4993	1191.5342	-0.0349	114 -	123	0	K.GIDWCVTDAR.S
1346.6834	1345.6761	1345.7201	-0.0440	99 -	113	0	K.VLSASGSGSNAGVIK.G
1568.7599	1567.7526	1567.8107	-0.0580	1 -	15	0	-.ALTTQPNAPSWGLGR.I
1784.8785	1783.8713	1783.9288	-0.0576	257 -	273	1	K.QIGNAVVRNPGTSTTNR.L
2089.9856	2088.9783	2089.0551	-0.0768	131 -	151	0	K.AALNLSLGGGFNQATNDAVTR.A
2923.3357	2922.3284	2922.4147	-0.0863	194 -	222	0	K.SSFSNWGSIVDIYAPGSNIISDAPGGGVR.T
3203.3560	3202.3487	3202.4449	-0.0962	223 -	254	0	R.TMSGTSMASPHVCGAGAAMLAQGVPVGQVCDR.L
3811.7322	3810.7249	3810.7760	-0.0511	20 -	55	1	R.SPGNKDFVYDDTAGQGITIYGVDTGIDINHPDFGGR.A

No match to: 724.4434, 733.4623, 737.3729, 744.4733, 746.4565, 749.4891, 755.4316, 760.4791, 762.5109, 768.4706, 786.5005, 790.5098, 798.4902, 818.3948, 839.4433, 858.4949, 859.4981, 861.3915, 899.4702, 1086.5004, 1129.5076, 1132.5085, 1133.4841, 1134.5315, 1224.5101, 1235.5029, 1313.6464, 1320.5973, 1334.6139, 1384.6472, 1393.6626, 1427.6527, 1433.7258, 1467.7009, 1503.6478, 1510.7076, 1524.6400, 1546.6444, 1557.7217, 1594.6986, 1600.7336, 1605.7229, 1607.7161, 1611.7679, 1669.7307, 1712.7358, 1730.7920, 1767.8136, 1834.8348, 1893.8197, 1936.8419, 2002.8632, 2009.9857, 2052.9927, 2110.9190, 2127.9177, 2132.9998, 2142.9309, 2160.9346, 2198.0662, 2198.5911, 2203.9448, 2215.0576, 2258.0129, 2317.9634, 2351.0039, 2359.0432, 2360.9961, 2380.0496, 2398.1003, 2433.0029, 2436.9856, 2441.0759, 2442.1169, 2476.0034, 2547.0313, 2548.0212, 2553.1460, 2573.9905, 2591.0327, 2654.1919, 2822.3552, 2922.3638, 2966.3379, 2985.4771, 3033.4314, 3076.4116, 3083.3225, 3100.4958, 3173.6069, 3188.4954, 3210.4119, 3230.5667, 3253.4275, 3257.3911, 3274.5586, 3391.6094, 3626.8101, 3634.5938, 3733.6704, 3757.6819, 3797.7144, 3839.6951, 3959.6892, 4000.7944, 4005.7834, 4016.8291, 4032.7107, 4075.7166, 4125.9771, 4221.9639, 4322.8979, 4377.2358, 4378.1675, 4395.2231

7. Protease 1 94-371 **Mass:** 28274 **Score:** 70 **Expect:** 1.5e-006 **Queries matched:** 11

Observed	Mr(expt)	Mr(calc)	Delta	Start	End	Miss	Peptide
856.4697	855.4624	855.4926	-0.0302	257 -	264	0	K.QIGNAVVR.N
947.4228	946.4155	946.4468	-0.0313	265 -	273	0	R.NPGTSTTNR.L
1097.6431	1096.6358	1096.6716	-0.0358	255 -	264	1	R.LKQIGNAVVR.N
1192.5066	1191.4993	1191.5342	-0.0349	114 -	123	0	K.GIDWCVTDAR.S
1346.6834	1345.6761	1345.7201	-0.0440	99 -	113	0	K.VLSASGSGSNAGVIK.G
1568.7599	1567.7526	1567.8107	-0.0580	1 -	15	0	-.ALTTQPNAPSWGLGR.I
1784.8785	1783.8713	1783.9288	-0.0576	257 -	273	1	K.QIGNAVVRNPGTSTTNR.L
2089.9856	2088.9783	2089.0551	-0.0768	131 -	151	0	K.AALNLSLGGGFNQATNDAVTR.A
2923.3357	2922.3284	2922.4147	-0.0863	194 -	222	0	K.SSFSNWGSIVDIYAPGSNIISDAPGGGVR.T
3203.3560	3202.3487	3202.4449	-0.0962	223 -	254	0	R.TMSGTSMASPHVCGAGAAMLAQGVPVGQVCDR.L
3811.7322	3810.7249	3810.7760	-0.0511	20 -	55	1	R.SPGNKDFVYDDTAGQGITIYGVDTGIDINHPDFGGR.A

No match to: 724.4434, 733.4623, 737.3729, 744.4733, 746.4565, 749.4891, 755.4316, 760.4791, 762.5109, 768.4706, 786.5005, 790.5098, 798.4902, 818.3948, 839.4433, 858.4949, 859.4981, 861.3915, 899.4702, 1086.5004, 1129.5076, 1132.5085, 1133.4841, 1134.5315, 1224.5101, 1235.5029, 1313.6464, 1320.5973, 1334.6139, 1384.6472, 1393.6626, 1427.6527, 1433.7258, 1467.7009, 1503.6478, 1510.7076, 1524.6400, 1546.6444, 1557.7217, 1594.6986, 1600.7336, 1605.7229, 1607.7161, 1611.7679, 1669.7307, 1712.7358, 1730.7920, 1767.8136, 1834.8348, 1893.8197, 1936.8419, 2002.8632, 2009.9857, 2052.9927, 2110.9190, 2127.9177, 2132.9998, 2142.9309, 2160.9346, 2198.0662, 2198.5911, 2203.9448, 2215.0576, 2258.0129, 2317.9634, 2351.0039, 2359.0432, 2360.9961, 2380.0496, 2398.1003, 2433.0029, 2436.9856, 2441.0759, 2442.1169, 2476.0034, 2547.0313, 2548.0212, 2553.1460, 2573.9905, 2591.0327, 2654.1919, 2822.3552, 2922.3638, 2966.3379, 2985.4771, 3033.4314, 3076.4116, 3083.3225, 3100.4958, 3173.6069, 3188.4954, 3210.4119, 3230.5667, 3253.4275, 3257.3911, 3274.5586, 3391.6094, 3626.8101, 3634.5938, 3733.6704, 3757.6819, 3797.7144, 3839.6951, 3959.6892, 4000.7944, 4005.7834, 4016.8291, 4032.7107, 4075.7166, 4125.9771, 4221.9639, 4322.8979, 4377.2358, 4378.1675, 4395.2231

8. Protease 1 94-373 **Mass:** 28418 **Score:** 69 **Expect:** 1.6e-006 **Queries matched:** 11

Observed	Mr(expt)	Mr(calc)	Delta	Start	End	Miss	Peptide
856.4697	855.4624	855.4926	-0.0302	257 -	264	0	K.QIGNAVVR.N
947.4228	946.4155	946.4468	-0.0313	265 -	273	0	R.NPGTSTTNR.L
1097.6431	1096.6358	1096.6716	-0.0358	255 -	264	1	R.LKQIGNAVVR.N
1192.5066	1191.4993	1191.5342	-0.0349	114 -	123	0	K.GIDWCVTDAR.S
1346.6834	1345.6761	1345.7201	-0.0440	99 -	113	0	K.VLSASGSGSNAGVIK.G
1568.7599	1567.7526	1567.8107	-0.0580	1 -	15	0	-.ALTTQPNAPSWGLGR.I
1784.8785	1783.8713	1783.9288	-0.0576	257 -	273	1	K.QIGNAVVRNPGTSTTNR.L
2089.9856	2088.9783	2089.0551	-0.0768	131 -	151	0	K.AALNLSLGGGFNQATNDAVTR.A
2923.3357	2922.3284	2922.4147	-0.0863	194 -	222	0	K.SSFSNWGSIVDIYAPGSNIISDAPGGGVR.T
3203.3560	3202.3487	3202.4449	-0.0962	223 -	254	0	R.TMSGTSMASPHVCGAGAAMLAQGVPVGQVCDR.L
3811.7322	3810.7249	3810.7760	-0.0511	20 -	55	1	R.SPGNKDFVYDDTAGQGITIYGVDTGIDINHPDFGGR.A

No match to: 724.4434, 733.4623, 737.3729, 744.4733, 746.4565, 749.4891, 755.4316, 760.4791, 762.5109, 768.4706, 786.5005, 790.5098, 798.4902, 818.3948, 839.4433, 858.4949, 859.4981, 861.3915, 899.4702, 1086.5004, 1129.5076, 1132.5085, 1133.4841, 1134.5315, 1224.5101, 1235.5029, 1313.6464, 1320.5973, 1334.6139, 1384.6472, 1393.6626, 1427.6527, 1433.7258, 1467.7009, 1503.6478, 1510.7076, 1524.6400, 1546.6444, 1557.7217, 1594.6986, 1600.7336, 1605.7229, 1607.7161, 1611.7679, 1669.7307, 1712.7358, 1730.7920, 1767.8136, 1834.8348, 1893.8197, 1936.8419, 2002.8632, 2009.9857, 2052.9927, 2110.9190, 2127.9177, 2132.9998, 2142.9309, 2160.9346, 2198.0662, 2198.5911, 2203.9448, 2215.0576, 2258.0129, 2317.9634, 2351.0039, 2359.0432, 2360.9961, 2380.0496, 2398.1003, 2433.0029, 2436.9856, 2441.0759, 2442.1169, 2476.0034, 2547.0313, 2548.0212, 2553.1460, 2573.9905, 2591.0327, 2654.1919, 2822.3552, 2922.3638, 2966.3379, 2985.4771, 3033.4314, 3076.4116, 3083.3225, 3100.4958, 3173.6069, 3188.4954, 3210.4119, 3230.5667, 3253.4275, 3257.3911, 3274.5586, 3391.6094, 3626.8101, 3634.5938, 3733.6704, 3757.6819, 3797.7144, 3839.6951, 3959.6892, 4000.7944, 4005.7834, 4016.8291, 4032.7107, 4075.7166, 4125.9771, 4221.9639, 4322.8979, 4377.2358, 4378.1675, 4395.2231

9. Protease 1 94-374 **Mass:** 28546 **Score:** 69 **Expect:** 1.7e-006 **Queries matched:** 11

Observed	Mr(expt)	Mr(calc)	Delta	Start	End	Miss	Peptide
856.4697	855.4624	855.4926	-0.0302	257 -	264	0	K.QIGNAVVR.N
947.4228	946.4155	946.4468	-0.0313	265 -	273	0	R.NPGTSTTNR.L
1097.6431	1096.6358	1096.6716	-0.0358	255 -	264	1	R.LKQIGNAVVR.N
1192.5066	1191.4993	1191.5342	-0.0349	114 -	123	0	K.GIDWCVTDAR.S
1346.6834	1345.6761	1345.7201	-0.0440	99 -	113	0	K.VLSASGSGSNAGVIK.G
1568.7599	1567.7526	1567.8107	-0.0580	1 -	15	0	-.ALTTQPNAPSWGLGR.I
1784.8785	1783.8713	1783.9288	-0.0576	257 -	273	1	K.QIGNAVVRNPGTSTTNR.L

2089.9856	2088.9783	2089.0551	-0.0768	131	- 151	0	K.AALNLSLGGGFNQATNDAVTR.A
2923.3357	2922.3284	2922.4147	-0.0863	194	- 222	0	K.SSFSNWGSIVDIYAPGSNIISDAPGGGVR.T
3203.3560	3202.3487	3202.4449	-0.0962	223	- 254	0	R.TMSGTSMASPHVCGAGAAMLAQGVPVGQVCDR.L
3811.7322	3810.7249	3810.7760	-0.0511	20	- 55	1	R.SPGNKDFVYDDTAGQGITIYGVDTGIDINHPDFGGR.A

No match to: 724.4434, 733.4623, 737.3729, 744.4733, 746.4565, 749.4891, 755.4316, 760.4791, 762.5109, 768.4706, 786.5005, 790.5098, 798.4902, 818.3948, 839.4433, 858.4949, 859.4981, 861.3915, 899.4702, 1086.5004, 1129.5076, 1132.5085, 1133.4841, 1134.5315, 1224.5101, 1235.5029, 1313.6464, 1320.5973, 1334.6139, 1384.6472, 1393.6626, 1427.6527, 1433.7258, 1467.7009, 1503.6478, 1510.7076, 1524.6400, 1546.6444, 1557.7217, 1594.6986, 1600.7336, 1605.7229, 1607.7161, 1611.7679, 1669.7307, 1712.7358, 1730.7920, 1767.8136, 1834.8348, 1893.8197, 1936.8419, 2002.8632, 2009.9857, 2052.9927, 2110.9190, 2127.9177, 2132.9998, 2142.9309, 2160.9346, 2198.0662, 2198.5911, 2203.9448, 2215.0576, 2258.0129, 2317.9634, 2351.0039, 2359.0432, 2360.9961, 2380.0496, 2398.1003, 2433.0029, 2436.9856, 2441.0759, 2442.1169, 2476.0034, 2547.0313, 2548.0212, 2553.1460, 2573.9905, 2591.0327, 2654.1919, 2822.3552, 2922.3638, 2966.3379, 2985.4771, 3033.4314, 3076.4116, 3083.3225, 3100.4958, 3173.6069, 3188.4954, 3210.4119, 3230.5667, 3253.4275, 3257.3911, 3274.5586, 3391.6094, 3626.8101, 3634.5938, 3733.6704, 3757.6819, 3797.7144, 3839.6951, 3959.6892, 4000.7944, 4005.7834, 4016.8291, 4032.7107, 4075.7166, 4125.9771, 4221.9639, 4322.8979, 4377.2358, 4378.1675, 4395.2231

10. Protease 1 94-375 **Mass:** 28603 **Score:** 69 **Expect:** 1.8e-006 **Queries matched:** 11

Observed	Mr(expt)	Mr(calc)	Delta	Start	End	Miss	Peptide
856.4697	855.4624	855.4926	-0.0302	257	- 264	0	K.QIGNAVVR.N
947.4228	946.4155	946.4468	-0.0313	265	- 273	0	R.NPGTSTTNR.L
1097.6431	1096.6358	1096.6716	-0.0358	255	- 264	1	R.LKQIGNAVVR.N
1192.5066	1191.4993	1191.5342	-0.0349	114	- 123	0	K.GIDWCVTDAR.S
1346.6834	1345.6761	1345.7201	-0.0440	99	- 113	0	K.VLSASGSGSNAGVIK.G
1568.7599	1567.7526	1567.8107	-0.0580	1	- 15	0	-.ALTTQPNAPSWGLGR.I
1784.8785	1783.8713	1783.9288	-0.0576	257	- 273	1	K.QIGNAVVRNPGTSTTNR.L
2089.9856	2088.9783	2089.0551	-0.0768	131	- 151	0	K.AALNLSLGGGFNQATNDAVTR.A
2923.3357	2922.3284	2922.4147	-0.0863	194	- 222	0	K.SSFSNWGSIVDIYAPGSNIISDAPGGGVR.T
3203.3560	3202.3487	3202.4449	-0.0962	223	- 254	0	R.TMSGTSMASPHVCGAGAAMLAQGVPVGQVCDR.L
3811.7322	3810.7249	3810.7760	-0.0511	20	- 55	1	R.SPGNKDFVYDDTAGQGITIYGVDTGIDINHPDFGGR.A

No match to: 724.4434, 733.4623, 737.3729, 744.4733, 746.4565, 749.4891, 755.4316, 760.4791, 762.5109, 768.4706, 786.5005, 790.5098, 798.4902, 818.3948, 839.4433, 858.4949, 859.4981, 861.3915, 899.4702, 1086.5004, 1129.5076, 1132.5085, 1133.4841, 1134.5315, 1224.5101, 1235.5029, 1313.6464, 1320.5973, 1334.6139, 1384.6472, 1393.6626, 1427.6527, 1433.7258, 1467.7009, 1503.6478, 1510.7076, 1524.6400, 1546.6444, 1557.7217, 1594.6986, 1600.7336, 1605.7229, 1607.7161, 1611.7679, 1669.7307, 1712.7358, 1730.7920, 1767.8136, 1834.8348, 1893.8197, 1936.8419, 2002.8632, 2009.9857, 2052.9927, 2110.9190, 2127.9177, 2132.9998, 2142.9309, 2160.9346, 2198.0662, 2198.5911, 2203.9448, 2215.0576, 2258.0129, 2317.9634, 2351.0039, 2359.0432, 2360.9961, 2380.0496, 2398.1003, 2433.0029, 2436.9856, 2441.0759, 2442.1169, 2476.0034, 2547.0313, 2548.0212, 2553.1460, 2573.9905, 2591.0327, 2654.1919, 2822.3552, 2922.3638, 2966.3379, 2985.4771, 3033.4314, 3076.4116, 3083.3225, 3100.4958, 3173.6069, 3188.4954, 3210.4119, 3230.5667, 3253.4275, 3257.3911, 3274.5586, 3391.6094, 3626.8101, 3634.5938, 3733.6704, 3757.6819, 3797.7144, 3839.6951, 3959.6892, 4000.7944, 4005.7834, 4016.8291, 4032.7107, 4075.7166, 4125.9771, 4221.9639, 4322.8979, 4377.2358, 4378.1675, 4395.2231

11. Protease 1 94-376 **Mass:** 28690 **Score:** 69 **Expect:** 1.8e-006 **Queries matched:** 11

Observed	Mr(expt)	Mr(calc)	Delta	Start	End	Miss	Peptide
856.4697	855.4624	855.4926	-0.0302	257	- 264	0	K.QIGNAVVR.N
947.4228	946.4155	946.4468	-0.0313	265	- 273	0	R.NPGTSTTNR.L
1097.6431	1096.6358	1096.6716	-0.0358	255	- 264	1	R.LKQIGNAVVR.N
1192.5066	1191.4993	1191.5342	-0.0349	114	- 123	0	K.GIDWCVTDAR.S
1346.6834	1345.6761	1345.7201	-0.0440	99	- 113	0	K.VLSASGSGSNAGVIK.G
1568.7599	1567.7526	1567.8107	-0.0580	1	- 15	0	-.ALTTQPNAPSWGLGR.I
1784.8785	1783.8713	1783.9288	-0.0576	257	- 273	1	K.QIGNAVVRNPGTSTTNR.L
2089.9856	2088.9783	2089.0551	-0.0768	131	- 151	0	K.AALNLSLGGGFNQATNDAVTR.A
2923.3357	2922.3284	2922.4147	-0.0863	194	- 222	0	K.SSFSNWGSIVDIYAPGSNIISDAPGGGVR.T
3203.3560	3202.3487	3202.4449	-0.0962	223	- 254	0	R.TMSGTSMASPHVCGAGAAMLAQGVPVGQVCDR.L
3811.7322	3810.7249	3810.7760	-0.0511	20	- 55	1	R.SPGNKDFVYDDTAGQGITIYGVDTGIDINHPDFGGR.A

No match to: 724.4434, 733.4623, 737.3729, 744.4733, 746.4565, 749.4891, 755.4316, 760.4791, 762.5109, 768.4706, 786.5005, 790.5098, 798.4902, 818.3948, 839.4433, 858.4949, 859.4981, 861.3915, 899.4702, 1086.5004, 1129.5076, 1132.5085, 1133.4841, 1134.5315, 1224.5101, 1235.5029, 1313.6464, 1320.5973, 1334.6139, 1384.6472, 1393.6626, 1427.6527, 1433.7258, 1467.7009, 1503.6478, 1510.7076, 1524.6400, 1546.6444, 1557.7217, 1594.6986, 1600.7336, 1605.7229, 1607.7161, 1611.7679, 1669.7307, 1712.7358, 1730.7920, 1767.8136, 1834.8348, 1893.8197, 1936.8419, 2002.8632, 2009.9857, 2052.9927, 2110.9190, 2127.9177, 2132.9998, 2142.9309, 2160.9346, 2198.0662, 2198.5911, 2203.9448, 2215.0576, 2258.0129, 2317.9634, 2351.0039, 2359.0432, 2360.9961, 2380.0496, 2398.1003, 2433.0029, 2436.9856, 2441.0759, 2442.1169, 2476.0034, 2547.0313, 2548.0212, 2553.1460, 2573.9905, 2591.0327, 2654.1919, 2822.3552, 2922.3638, 2966.3379, 2985.4771, 3033.4314, 3076.4116, 3083.3225, 3100.4958, 3173.6069, 3188.4954, 3210.4119, 3230.5667, 3253.4275, 3257.3911, 3274.5586, 3391.6094, 3626.8101, 3634.5938, 3733.6704, 3757.6819, 3797.7144, 3839.6951, 3959.6892, 4000.7944, 4005.7834, 4016.8291, 4032.7107, 4075.7166, 4125.9771, 4221.9639, 4322.8979, 4377.2358, 4378.1675, 4395.2231

12. Protease 1 94-382 **Mass:** 29512 **Score:** 68 **Expect:** 2.2e-006 **Queries matched:** 11

Observed	Mr(expt)	Mr(calc)	Delta	Start	End	Miss	Peptide
856.4697	855.4624	855.4926	-0.0302	257	- 264	0	K.QIGNAVVR.N
947.4228	946.4155	946.4468	-0.0313	265	- 273	0	R.NPGTSTTNR.L
1097.6431	1096.6358	1096.6716	-0.0358	255	- 264	1	R.LKQIGNAVVR.N
1192.5066	1191.4993	1191.5342	-0.0349	114	- 123	0	K.GIDWCVTDAR.S
1346.6834	1345.6761	1345.7201	-0.0440	99	- 113	0	K.VLSASGSGSNAGVIK.G
1568.7599	1567.7526	1567.8107	-0.0580	1	- 15	0	-.ALTTQPNAPSWGLGR.I
1784.8785	1783.8713	1783.9288	-0.0576	257	- 273	1	K.QIGNAVVRNPGTSTTNR.L
2089.9856	2088.9783	2089.0551	-0.0768	131	- 151	0	K.AALNLSLGGGFNQATNDAVTR.A
2923.3357	2922.3284	2922.4147	-0.0863	194	- 222	0	K.SSFSNWGSIVDIYAPGSNIISDAPGGGVR.T
3203.3560	3202.3487	3202.4449	-0.0962	223	- 254	0	R.TMSGTSMASPHVCGAGAAMLAQGVPVGQVCDR.L
3811.7322	3810.7249	3810.7760	-0.0511	20	- 55	1	R.SPGNKDFVYDDTAGQGITIYGVDTGIDINHPDFGGR.A

No match to: 724.4434, 733.4623, 737.3729, 744.4733, 746.4565, 749.4891, 755.4316, 760.4791, 762.5109, 768.4706, 786.5005, 790.5098, 798.4902, 818.3948, 839.4433, 858.4949, 859.4981, 861.3915, 899.4702, 1086.5004, 1129.5076, 1132.5085, 1133.4841, 1134.5315, 1224.5101, 1235.5029, 1313.6464, 1320.5973, 1334.6139, 1384.6472, 1393.6626, 1427.6527, 1433.7258, 1467.7009, 1503.6478, 1510.7076, 1524.6400, 1546.6444, 1557.7217, 1594.6986, 1600.7336, 1605.7229, 1607.7161, 1611.7679, 1669.7307, 1712.7358, 1730.7920, 1767.8136, 1834.8348, 1893.8197, 1936.8419, 2002.8632, 2009.9857, 2052.9927, 2110.9190, 2127.9177, 2132.9998, 2142.9309, 2160.9346, 2198.0662, 2198.5911, 2203.9448, 2215.0576, 2258.0129, 2317.9634, 2351.0039, 2359.0432, 2360.9961, 2380.0496, 2398.1003, 2433.0029, 2436.9856, 2441.0759, 2442.1169, 2476.0034, 2547.0313, 2548.0212, 2553.1460, 2573.9905, 2591.0327, 2654.1919,

2822.3552, 2922.3638, 2966.3379, 2985.4771, 3033.4314, 3076.4116, 3083.3225, 3100.4958, 3173.6069, 3188.4954, 3210.4119, 3230.5667, 3253.4275, 3257.3911, 3274.5586, 3391.6094, 3626.8101, 3634.5938, 3733.6704, 3757.6819, 3797.7144, 3839.6951, 3959.6892, 4000.7944, 4005.7834, 4016.8291, 4032.7107, 4075.7166, 4125.9771, 4221.9639, 4322.8979, 4377.2358, 4378.1675, 4395.2231

13. Protease 1 full length 1-382 **Mass:** 39937 **Score:** 64 **Expect:** 5.2e-006 **Queries matched:** 12

Observed	Mr(expt)	Mr(calc)	Delta	Start	End	Miss	Peptide
818.3948	817.3876	817.3718	0.0157	83 -	88	0	K.YVEHDR.V
856.4697	855.4624	855.4926	-0.0302	350 -	357	0	K.QIGNAVVR.N
947.4228	946.4155	946.4468	-0.0313	358 -	366	0	R.NPGTSTTNR.L
1097.6431	1096.6358	1096.6716	-0.0358	348 -	357	1	R.LKQIGNAVVR.N
1192.5066	1191.4993	1191.5342	-0.0349	207 -	216	0	K.GIDWCVTDAR.S
1346.6834	1345.6761	1345.7201	-0.0440	192 -	206	0	K.VLSASGSGSNAGVIK.G
1784.8785	1783.8713	1783.9288	-0.0576	350 -	366	1	K.QIGNAVVRNPGTSTTNR.L
2089.9856	2088.9783	2089.0551	-0.0768	224 -	244	0	K.AALNLSLGGGFNQATNDAVTR.A
2380.0496	2379.0423	2379.2044	-0.1622	2 -	22	1	R.AFFHNKGGNDIIPNSYIVVMK.D + Oxidation (M)
2923.3357	2922.3284	2922.4147	-0.0863	287 -	315	0	K.SSFSNWGSIVDIYAPGSNIISDAPGGGVR.T
3203.3560	3202.3487	3202.4449	-0.0962	316 -	347	0	R.TMSGTSMASPHVCGAGAAMLAQGVPVGQVCDR.L
3811.7322	3810.7249	3810.7760	-0.0511	113 -	148	1	R.SPGNKDFVYDDTAGQGITIYGVDTGIDINHPDFGGR.A

No match to: 724.4434, 733.4623, 737.3729, 744.4733, 746.4565, 749.4891, 755.4316, 760.4791, 762.5109, 768.4706, 786.5005, 790.5098, 798.4902, 839.4433, 858.4949, 859.4981, 861.3915, 899.4702, 1086.5004, 1129.5076, 1132.5085, 1133.4841, 1134.5315, 1224.5101, 1235.5029, 1313.6464, 1320.5973, 1334.6139, 1384.6472, 1393.6626, 1427.6527, 1433.7258, 1467.7009, 1503.6478, 1510.7076, 1524.6400, 1546.6444, 1557.7217, 1568.7599, 1594.6986, 1600.7336, 1605.7229, 1607.7161, 1611.7679, 1669.7307, 1712.7358, 1730.7920, 1767.8136, 1834.8348, 1893.8197, 1936.8419, 2002.8632, 2009.9857, 2052.9927, 2110.9190, 2127.9177, 2132.9998, 2142.9309, 2160.9346, 2198.0662, 2198.5911, 2203.9448, 2215.0576, 2258.0129, 2317.9634, 2351.0039, 2359.0432, 2360.9961, 2398.1003, 2433.0029, 2436.9856, 2441.0759, 2442.1169, 2476.0034, 2547.0313, 2548.0212, 2553.1460, 2573.9905, 2591.0327, 2654.1919, 2822.3552, 2922.3638, 2966.3379, 2985.4771, 3033.4314, 3076.4116, 3083.3225, 3100.4958, 3173.6069, 3188.4954, 3210.4119, 3230.5667, 3253.4275, 3257.3911, 3274.5586, 3391.6094, 3626.8101, 3634.5938, 3733.6704, 3757.6819, 3797.7144, 3839.6951, 3959.6892, 4000.7944, 4005.7834, 4016.8291, 4032.7107, 4075.7166, 4125.9771, 4221.9639, 4322.8979, 4377.2358, 4378.1675, 4395.2231

14. Protease 1 full length plus Signal peptide **Mass:** 41853 **Score:** 62 **Expect:** 8.2e-006 **Queries matched:** 12

Observed	Mr(expt)	Mr(calc)	Delta	Start	End	Miss	Peptide
818.3948	817.3876	817.3718	0.0157	102 -	107	0	K.YVEHDR.V
856.4697	855.4624	855.4926	-0.0302	369 -	376	0	K.QIGNAVVR.N
947.4228	946.4155	946.4468	-0.0313	377 -	385	0	R.NPGTSTTNR.L
1097.6431	1096.6358	1096.6716	-0.0358	367 -	376	1	R.LKQIGNAVVR.N
1192.5066	1191.4993	1191.5342	-0.0349	226 -	235	0	K.GIDWCVTDAR.S
1346.6834	1345.6761	1345.7201	-0.0440	211 -	225	0	K.VLSASGSGSNAGVIK.G
1784.8785	1783.8713	1783.9288	-0.0576	369 -	385	1	K.QIGNAVVRNPGTSTTNR.L
2089.9856	2088.9783	2089.0551	-0.0768	243 -	263	0	K.AALNLSLGGGFNQATNDAVTR.A
2380.0496	2379.0423	2379.2044	-0.1622	21 -	41	1	R.AFFHNKGGNDIIPNSYIVVMK.D + Oxidation (M)
2923.3357	2922.3284	2922.4147	-0.0863	306 -	334	0	K.SSFSNWGSIVDIYAPGSNIISDAPGGGVR.T
3203.3560	3202.3487	3202.4449	-0.0962	335 -	366	0	R.TMSGTSMASPHVCGAGAAMLAQGVPVGQVCDR.L
3811.7322	3810.7249	3810.7760	-0.0511	132 -	167	1	R.SPGNKDFVYDDTAGQGITIYGVDTGIDINHPDFGGR.A

No match to: 724.4434, 733.4623, 737.3729, 744.4733, 746.4565, 749.4891, 755.4316, 760.4791, 762.5109, 768.4706, 786.5005, 790.5098, 798.4902, 839.4433, 858.4949, 859.4981, 861.3915, 899.4702, 1086.5004, 1129.5076, 1132.5085, 1133.4841, 1134.5315, 1224.5101, 1235.5029, 1313.6464, 1320.5973, 1334.6139, 1384.6472, 1393.6626, 1427.6527, 1433.7258, 1467.7009, 1503.6478, 1510.7076, 1524.6400, 1546.6444, 1557.7217, 1568.7599, 1594.6986, 1600.7336, 1605.7229, 1607.7161, 1611.7679, 1669.7307, 1712.7358, 1730.7920, 1767.8136, 1834.8348, 1893.8197, 1936.8419, 2002.8632, 2009.9857, 2052.9927, 2110.9190, 2127.9177, 2132.9998, 2142.9309, 2160.9346, 2198.0662, 2198.5911, 2203.9448, 2215.0576, 2258.0129, 2317.9634, 2351.0039, 2359.0432, 2360.9961, 2398.1003, 2433.0029, 2436.9856, 2441.0759, 2442.1169, 2476.0034, 2547.0313, 2548.0212, 2553.1460, 2573.9905, 2591.0327, 2654.1919, 2822.3552, 2922.3638, 2966.3379, 2985.4771, 3033.4314, 3076.4116, 3083.3225, 3100.4958, 3173.6069, 3188.4954, 3210.4119, 3230.5667, 3253.4275, 3257.3911, 3274.5586, 3391.6094, 3626.8101, 3634.5938, 3733.6704, 3757.6819, 3797.7144, 3839.6951, 3959.6892, 4000.7944, 4005.7834, 4016.8291, 4032.7107, 4075.7166, 4125.9771, 4221.9639, 4322.8979, 4377.2358, 4378.1675, 4395.2231

Search Parameters

Type of search : Peptide Mass Fingerprint
Enzyme : Trypsin
Fixed modifications : Carbamidomethyl (C)
Variable modifications : Oxidation (M)
Mass values : Monoisotopic
Protein Mass : Unrestricted
Peptide Mass Tolerance : ± 100 ppm
Peptide Charge State : 1+
Max Missed Cleavages : 1
Number of queries : 126

Mascot: http://www.matrixscience.com/

Mascot Search Results

Protein View

Match to: **Protease_1_94-372** Score: **79** Expect: **2e-007**

Nominal mass (M_r): **28361**; Calculated pI value: **8.70**
NCBI BLAST search of Protease_1_94-372 against nr
Unformatted sequence string for pasting into other applications

Fixed modifications: Carbamidomethyl (C)
Variable modifications: Oxidation (M)
Cleavage by Trypsin: cuts C-term side of KR unless next residue is P
Number of mass values searched: **126**
Number of mass values matched: **12**
Sequence Coverage: **65%**

Matched peptides shown in **Bold Red**

```
  1 ALTTQPNAPS WGLGRISHRS PGNKDFVYDD TAGQGITIYG VDTGIDINHP
 51 DFGGRARWGT NTVDNANNDG HGHGTHTAGT FAGNAYGIAK KASVVAVKVL
101 SASGSGSNAG VIKGIDWCVT DARSKGALGK AALNLSLGGG FNQATNDAVT
151 RAQTAGIFVA VAAGNDNKDA RNYSPASAPA VCTVASSTID DQKSSFSNWG
201 SIVDIYAPGS NIISDAPGGG VRTMSGTSMA SPHVCGAGAA MLAQGVPVGQ
251 VCDRLKQIGN AVVRNPGTST TNRLLYNGS
```

Show predicted peptides also

Sort Peptides By ◉Residue Number ○Increasing Mass ○Decreasing Mass

Start - End	Observed	Mr(expt)	Mr(calc)	Delta	Miss	Sequence
1 - 15	1568.7599	1567.7526	1567.8107	-0.0580	0	-.ALTTQPNAPSWGLGR.I
20 - 55	3811.7322	3810.7249	3810.7760	-0.0511	1	R.SPGNKDFVYDDTAGQGITIYGVDTGIDINHPDFGGR.A
99 - 113	1346.6834	1345.6761	1345.7201	-0.0440	0	K.VLSASGSGSNAGVIK.G
114 - 123	1192.5066	1191.4993	1191.5342	-0.0349	0	K.GIDWCVTDAR.S
131 - 151	2089.9856	2088.9783	2089.0551	-0.0768	0	K.AALNLSLGGGFNQATNDAVTR.A
194 - 222	2923.3357	2922.3284	2922.4147	-0.0863	0	K.SSFSNWGSIVDIYAPGSNIISDAPGGGVR.T
223 - 254	3203.3560	3202.3487	3202.4449	-0.0962	0	R.TMSGTSMASPHVCGAGAAMLAQGVPVGQVCDR.L
255 - 264	1097.6431	1096.6358	1096.6716	-0.0358	1	R.LKQIGNAVVR.N
257 - 264	856.4697	855.4624	855.4926	-0.0302	0	K.QIGNAVVR.N
257 - 273	1784.8785	1783.8713	1783.9288	-0.0576	1	K.QIGNAVVRNPGTSTTNR.L
265 - 273	947.4228	946.4155	946.4468	-0.0313	0	R.NPGTSTTNR.L
265 - 279	1594.6986	1593.6913	1593.7747	-0.0833	1	R.NPGTSTTNRLLYNGS.-

No match to: 724.4434, 733.4623, 737.3729, 744.4733, 746.4565, 749.4891, 755.4316, 760.4791, 762.5109, 768.4706, 786.5005, 790.5098, 798.4902, 818.

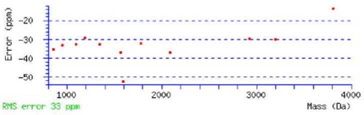

Mascot: http://www.matrixscience.com/

Mascot Search Results

Peptide View

MS/MS Fragmentation of **ALTTQPNAPSWGLGR**
Found in **Protease_1_94-366**

Match to Query 1: 1567.752724 from(1568.760000,1+)

Click mouse within plot area to zoom in by factor of two about that point
Or, Plot from 0 to 1600 Da

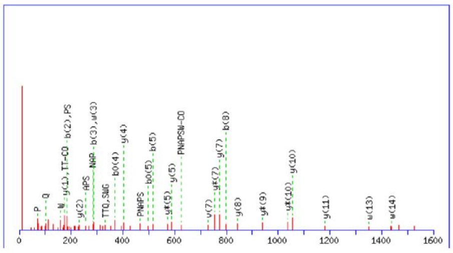

Monoisotopic mass of neutral peptide Mr(calc): 1567.8107
Fixed modifications: Carbamidomethyl (C)
Ions Score: 56 **Expect:** 1.5e-006
Matches (Bold Red): 35/256 fragment ions using 39 most intense peaks

#	Immon.	a	a*	a^0	b	b*	b^0	Seq.	v	w	w'	y	y*	y^0	#
1	44.0495	44.0495			72.0444			A							15
2	86.0964	157.1335			**185.1284**			L	1439.7026	**1438.7073**		1497.7808	1480.7543	1479.7703	14
3	74.0600	258.1812		240.1706	**286.1761**		268.1656	T	1338.6549	**1351.6753**	1353.6546	1384.6968	1367.6702	1366.6862	13
4	74.0600	359.2289		341.2183	387.2238		**369.2132**	T	1237.6072	1250.6276	1252.6069	1283.6491	1266.6225	1265.6385	12
5	**101.0709**	487.2875	470.2609	469.2769	**515.2824**	498.2558	**497.2718**	Q	1109.5486	1108.5534		**1182.6014**	1165.5749	1164.5908	11
6	**70.0651**	584.3402	567.3137	566.3297	612.3351	595.3086	594.3246	P	1012.4959	1011.5006		**1054.5428**	**1037.5163**	1036.5323	10
7	87.0553	698.3832	681.3566	680.3726	726.3781	709.3515	708.3675	N	898.4530	897.4577		957.4901	**940.4635**	939.4795	9
8	44.0495	769.4203	752.3937	751.4097	**797.4152**	780.3886	779.4046	A	827.4158			**843.4471**	826.4206	825.4366	8
9	**70.0651**	866.4730	849.4465	848.4625	894.4679	877.4414	876.4574	P	**730.3631**	729.3678		**772.4100**	**755.3835**	754.3995	7
10	60.0444	953.5051	936.4785	935.4945	981.5000	964.4734	963.4894	S	643.3311	642.3358		675.3573	658.3307	657.3467	6
11	**159.0917**	1139.5844	1122.5578	1121.5738	1167.5793	1150.5527	1149.5687	W	457.2517			**588.3252**	**571.2987**		5
12	30.0338	1196.6058	1179.5793	1178.5953	1224.6007	1207.5742	1206.5902	G				**402.2459**	385.2194		4
13	86.0964	1309.6899	1292.6633	1291.6793	1337.6848	1320.6583	1319.6742	L	287.1462	**286.1510**		345.2245	328.1979		3
14	30.0338	1366.7114	1349.6848	1348.7008	1394.7063	1377.6797	1376.6957	G				**232.1404**	215.1139		2
15	129.1135							R	74.0237	73.0284		**175.1190**	158.0924		1

Seq	ya	yb	Seq	ya	yb	Seq	ya	yb
LT	187.1441	215.1390	LTT	288.1918	316.1867	LTTQ	416.2504	444.2453
LTTQP	513.3031	541.2980	LTTQPN	627.3461	655.3410	LTTQPNA	698.3832	726.3781
TT	**175.1077**	203.1026	TTQ	303.1663	**331.1612**	TTQP	400.2191	428.2140
TTQPN	514.2620	542.2569	TTQPNA	585.2991	613.2940	TTQPNAP	682.3519	710.3468
TQ	202.1186	230.1135	TQP	299.1714	327.1663	TQPN	413.2143	441.2092
TQPNA	484.2514	512.2463	TQPNAP	581.3042	609.2991	TQPNAPS	668.3362	696.3311
QP	198.1237	226.1186	QPN	312.1666	340.1615	QPNA	383.2037	411.1987
QPNAP	480.2565	508.2514	QPNAPS	567.2885	595.2834	PN	184.1081	212.1030
PNA	255.1452	**283.1401**	PNAP	352.1979	380.1928	PNAPS	439.2300	**467.2249**
PNAPSW	**625.3093**	653.3042	PNAPSWG	682.3307	710.3256	NA	158.0924	186.0873
NAP	255.1452	**283.1401**	NAPS	342.1772	370.1721	NAPSW	528.2565	556.2514
NAPSWG	585.2780	613.2729	NAPSWGL	698.3620	726.3569	AP	141.1022	169.0971
APS	228.1343	**256.1292**	APSW	414.2136	442.2085	APSWG	471.2350	499.2299
APSWGL	584.3191	612.3140	APSWGLG	641.3406	669.3355	PS	157.0972	**185.0921**
PSW	343.1765	371.1714	PSWG	400.1979	428.1928	PSWGL	513.2820	541.2769
PSWGLG	570.3034	598.2984	SW	246.1237	274.1186	SWG	303.1452	**331.1401**
SWGL	416.2292	444.2241	SWGLG	473.2507	501.2456	WG	216.1131	244.1080
WGL	329.1972	357.1921	WGLG	386.2187	414.2136	GL	143.1179	171.1128
GLG	200.1393	228.1343	LG	143.1179	171.1128			

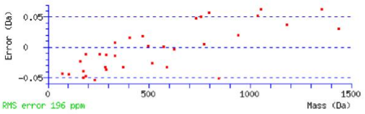

NCBI **BLAST** search of ALTTQPNAPSWGLGR
(Parameters: blastp, nr protein database, expect=20000, no filter, PAM30)
Other BLAST web gateways

Mascot: http://www.matrixscience.com/
