# Supplementary material for: An alternative for proteinase K-heat-sensitive protease from fungus Onygena corvina for biotechnology: cloning, engineering, expression, characterization and special application for protein sequencing
Source: Microb Cell Fact. 2020 Jun 24;19:135. doi: 10.1186/s12934-020-01392-3 (PMC7313183; doi:10.1186/s12934-020-01392-3)
Supplement: Supplementary file 15 — Additional file 15. BLG digest with trypsin and matched protein sequence. BLG digest with NHSSP and matched protein sequence. Monoclonal mAb digest with trypsin and matched protein sequences. Monoclonal mAb digest with NHSSP and matched protein sequences. Cleavage sites around the bitter peptide region of BLG. [file 12934_2020_1392_MOESM15_ESM.pdf]

Figure Supplementary 15-1: BLG digest with trypsin and matched protein sequence

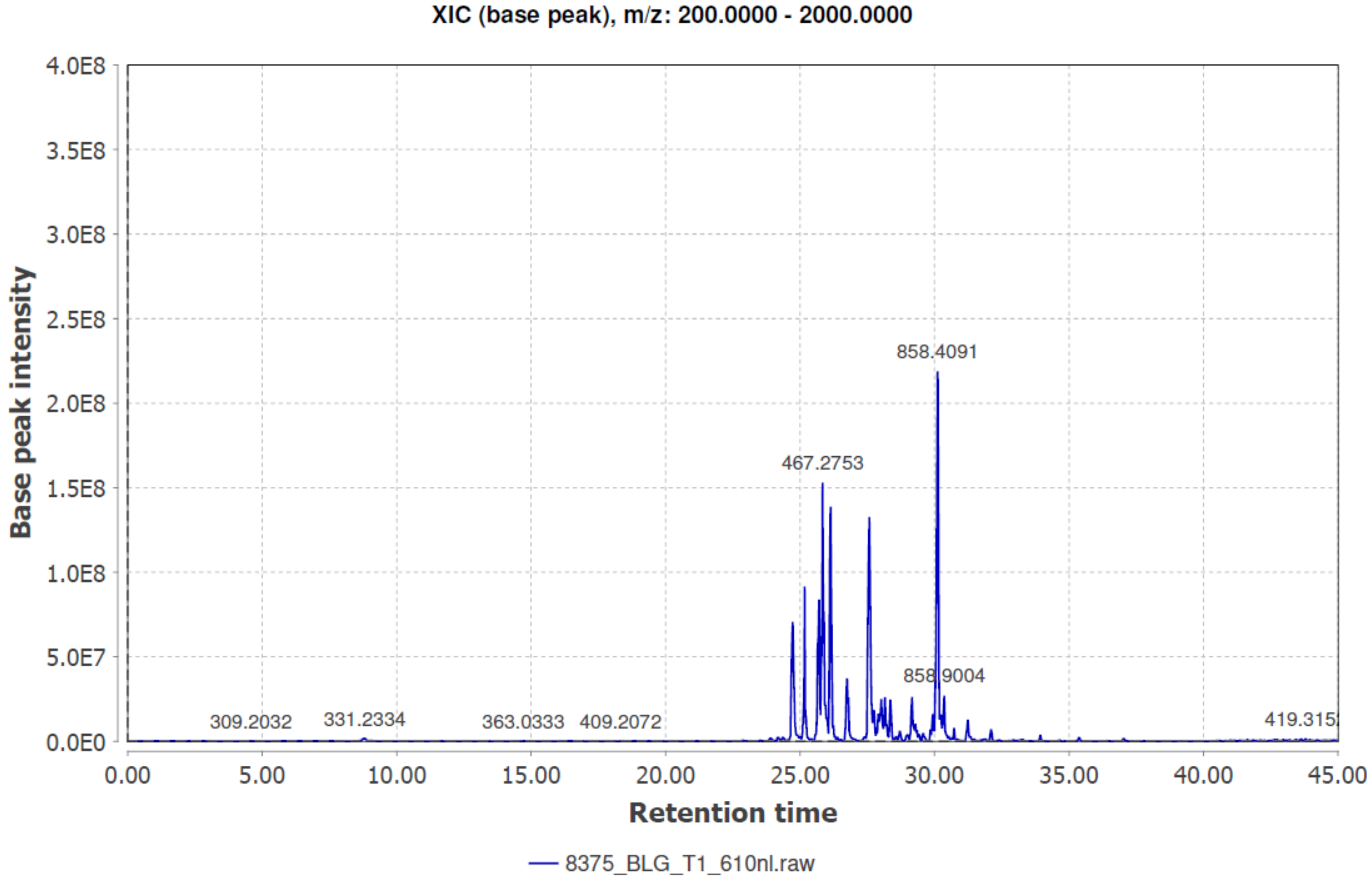


```
Sequence Coverage: 76%

Matched peptides shown in Bold Red

  1 LIVTQTMKGL DIQKVAGTWY SLAMAASDIS LLDAQSAPLR VYVEELKPTP
 51 EGDLEILLQK WENGECAQKK IIAEKTKIPA VFKIDALNEN KVLVLDTDYK
101 KYLLFCMENS AEPEQSLACQ CLVRTPEVDD EALEKFDKAL KALPMHIRLS
151 FNPTQLEEQC HI
```

Figure Supplementary 15-2: BLG digest with NHSSP-protease and matched protein sequence

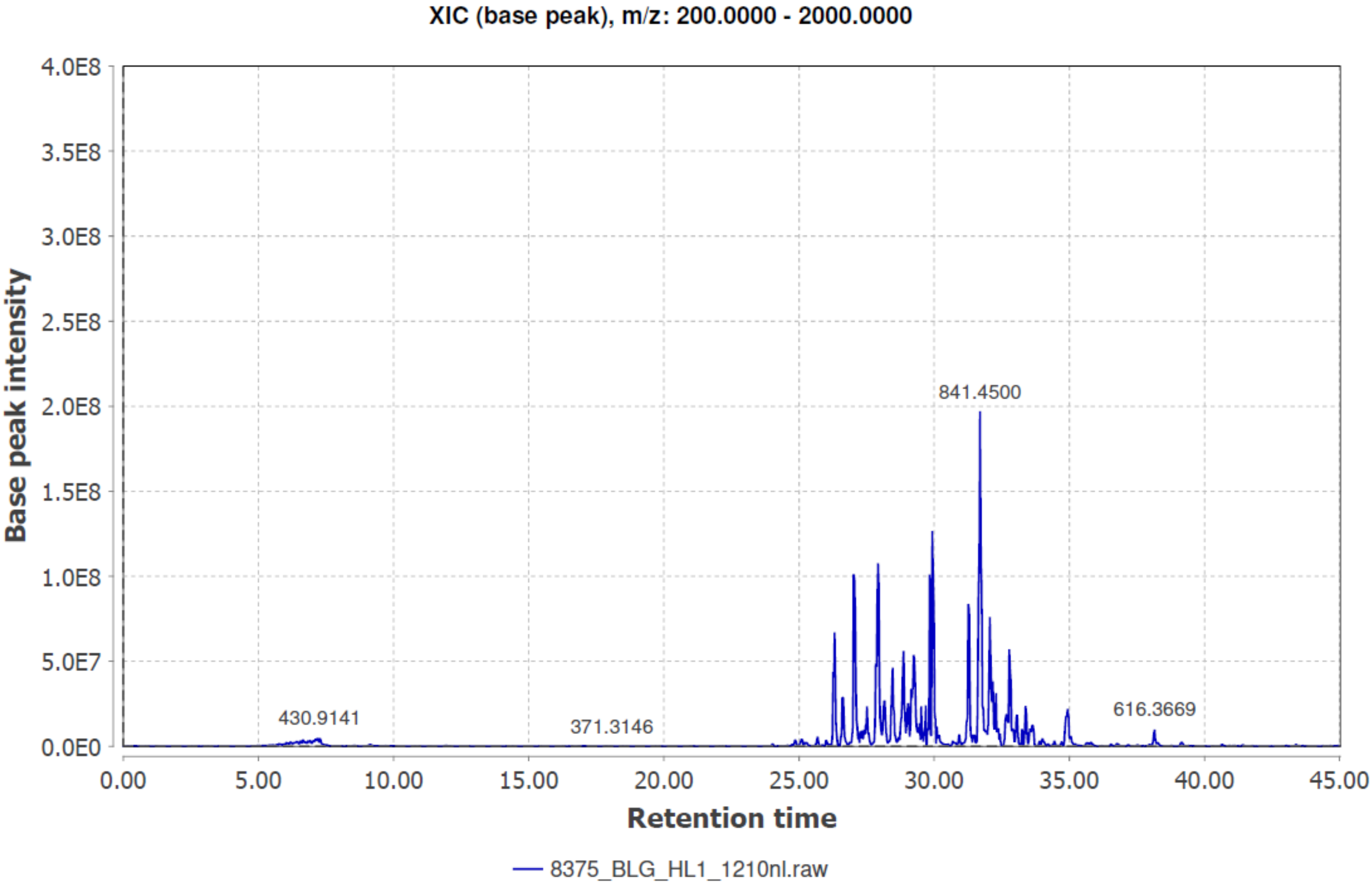

Sequence Coverage: **100%**

Matched peptides shown in **Bold Red**

```
  1 LIVTQTMKGL DIQKVAGTWY SLAMAASDIS LLDAQSAPLR VYVEELKPTP
 51 EGDLEILLQK WENGECAQKK IIAEKTKIPA VFKIDALNEN KVLVLDTDYK
101 KYLLFCMENS AEPEQSLACQ CLVRTPEVDD EALEKFDKAL KALPMHIRLS
151 FNPTQLEEQC HI
```

Figure Supplementary 15-3: mAB digest with trypsin and matched protein sequences

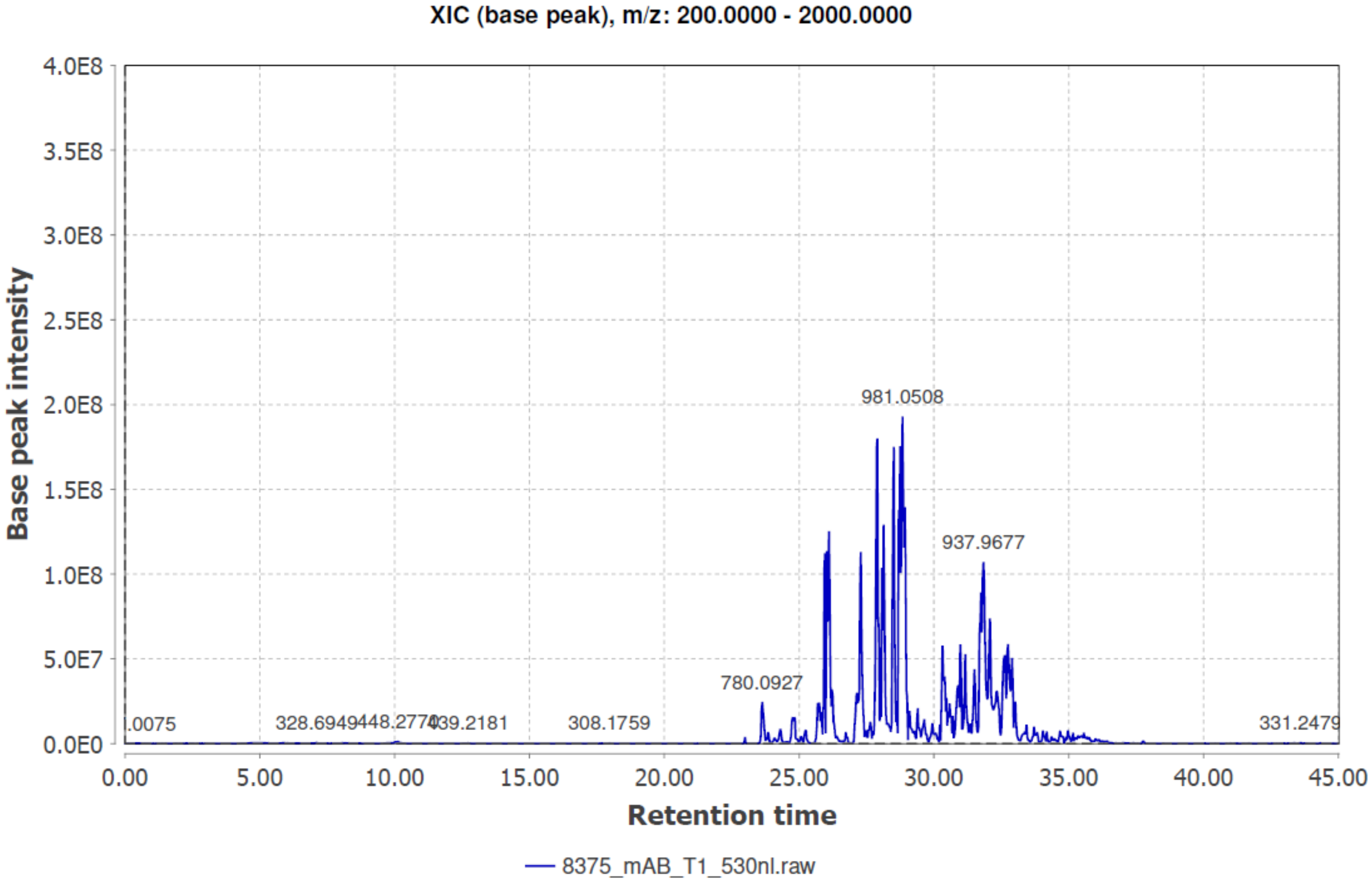

<u>mAB heavy chain:</u>

Sequence Coverage: **64%**

Matched peptides shown in **Bold Red**

1 QVQLQQPGAE LVKPGASVKM SCK**ASGYTFT** **SYNMHWVK**QT PGR**GLEWIGA**
51 **IYPGNGDTSY** **NQK**FK**GKATL** **TADKSSSTAY** **MQLSSLTSED** **SAVYYCARS**T
101 YYGGDWYFNV WGAGTTVTVS AASTK**GPSVF** **PLAPSSKSTS** **GGTAALGCLV**
151 **K**DYFPEPVTV SWNSGALTSG VHTFPAVLQS SGLYSLSSVV TVPSSSLGTQ
201 TYICNVNHKP SNTKVDKKVE PK**SCDKTHTC** **PPCPAPELLG** **GPSVFLFPPK**
251 **PKDTLMISRT** **PEVTCVVVDV** **SHEDPEVKFN** **WYVDGVEVHN** **AK**TKPREEQY
301 NSTYR**VVSVL** **TVLHQDWLNG** **KEYK**CKVSNK **ALPAPIEKTI** SKAKGQPREP
351 **QVYTLPPSRD** **ELTKNQVSLT** **CLVKGFYPSD** **IAVEWESNGQ** **PENNYKTTPP**
401 **VLDSDGSFFL** **YSK**LTVDKSR **WQQGNVFSCS** **VMHEALHNHY** **TQKSLSLSPG**
451 **K**

<u>mAB light chain:</u>

Sequence Coverage: **71%**

Matched peptides shown in **Bold Red**

1 QIVLSQSPAI LSASPGEK**VT** **MTCR**ASSSVS YIHWFQQKPG SSPKPWIYAT
51 SNLASGVPVR **FSGSGSGTSY** **SLTISRVEAE** **DAATYYCQQW** **TSNPPTFGGG**
101 **TKLEIKRTVA** **APSVFIFPPS** **DEQLKSGTAS** **VVCLLNNFYP** **R**EAKVQWK**VD**
151 **NALQSGNSQE** **SVTEQDSKDS** **TYSLSSTLTL** **SKADYEKHKV** **YACEVTHQGL**
201 **SSPVTKSFNR** **GEC**

Figure Supplementary 15-4: mAB digest with NHSSP-protease and matched protein sequences

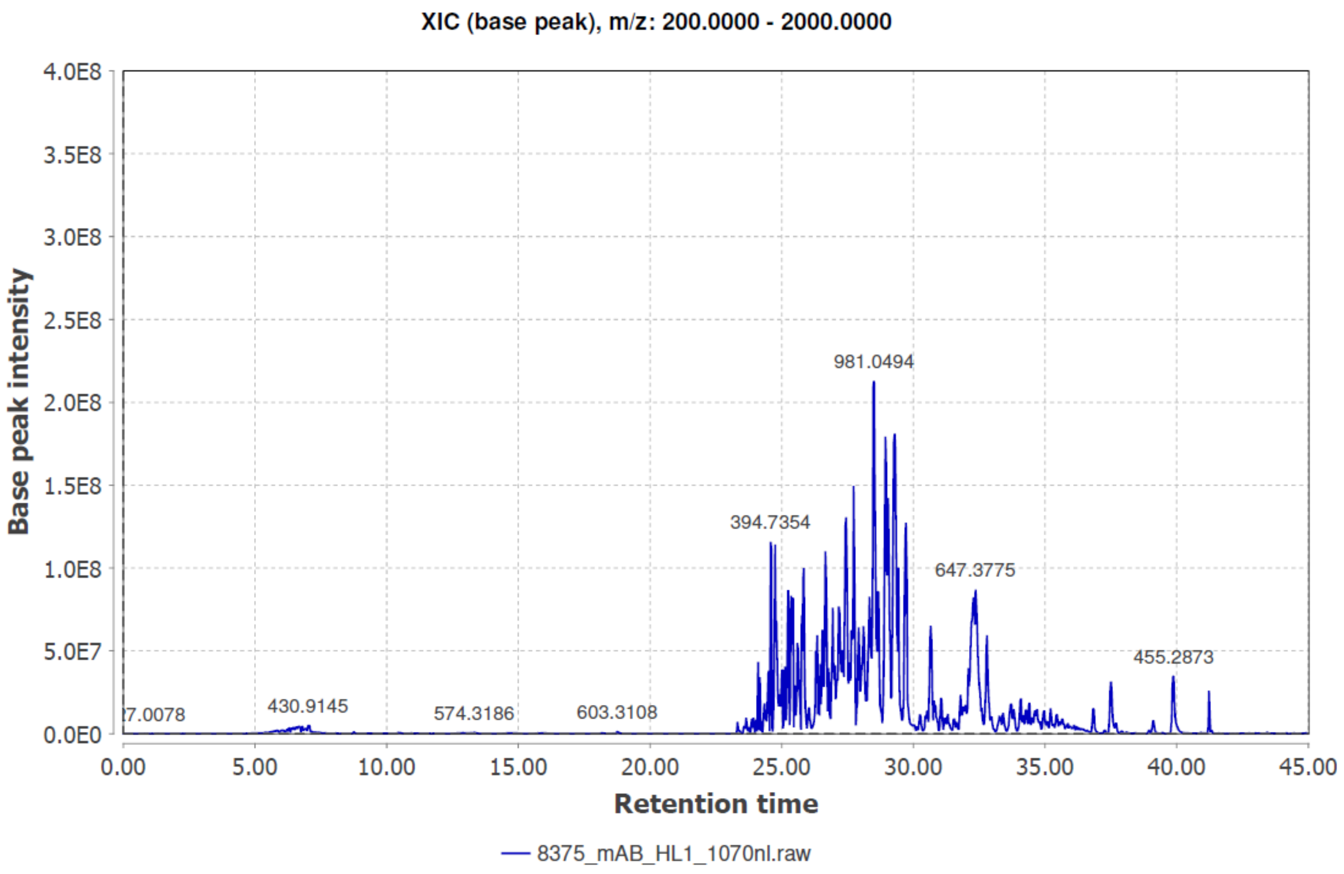

mAB heavy chain:

Sequence Coverage: **84%**

Matched peptides shown in **Bold Red**

1 QVQL**QQPGAE** **LVKPGASVK**M S**CKASGYTFT** **SYNMHWVKQT** **PGRGLEWIGA**
51 **IYPGNGDTSY** **NQKFKGKATL** **TADKSSSTAY** MQLS**SLTSED** **SAVYYCARST**
101 **YYGGDWYFN**V WGAGTTVTVS **AASTKGPSVF** **PLAPSSKSTS** **GGTAALGCLV**
151 **KDYFPEPVTV** **SWNSGALTSG** **VHTFPAVLQS** **SGLYSLSSVV** **TVPSSSLGTQ**
201 **TY**IC**NVNHKP** **S**NTKVDKKVE PK**SCDKTHTC** **PPCPAPELL**G GPSVF**LFPPK**
251 **PKDTLMIS**RT PEVT**CVVVDV** **SHEDPEVKFN** **WYVDGVEVH**N AKTKPREEQY
301 NSTYRVVSVL **TVLHQDWLNG** **KEYKCKVSNK** **ALPAPIEKTI** **SKAKGQPREP**
351 **QVYTLPPSRD** **ELTKNQVSLT** **CLVKGFYPSD** **IAVEWESNGQ** **PENNYKTTPP**
401 **VLDSDGSFF**L Y**SKLTVDKSR** **WQQGNVFSCS** **VMHEALHNHY** **TQKSLSLSPG**
451 **K**

mAB light chain:

Sequence Coverage: **96%**

Matched peptides shown in **Bold Red**

1 QIVL**SQSPAI** **LSASPGEKVT** **MTCRASSSVS** **YIHWFQQKPG** **SSPKPWIYAT**
51 **SNLASGVPVR** **FSGSGSGTSY** **SLTISRVEAE** **DAATYYCQQW** **TSNPPTFGGG**
101 **TKLEIKRTVA** **APSVFIFPPS** **DEQLKSGTAS** **VVCLLNNFYP** **REAKVQWKVD**
151 **NALQSGNSQE** **SVTEQDSKDS** **TYSLS**STL**TL** **SKADYEKHKV** **YACEVTHQGL**
201 **SSPVTKSFNR** **GEC**

Figure Supplementary 15-5: cleavage around bitter peptide region of ß-lactalbumin

START - END OBSERVED	MR(EXPT)	MR(CALC)	DELTA	MISS	SEQUENCE
70 – 82486.6409	1456.9010	1456.9017	0.0007	0	K.KIIAEKTKIPAVF.K (Ions score 38)
70 – 82486.6411	1456.9014 1	456.9017 -	0.0003	0	K.KIIAEKTKIPAVF.K (Ions score 24)
70 – 82 486.6411	1456.9016 1	456.9017 -	0.0001	0	K.KIIAEKTKIPAVF.K (Ions score 27)
70 – 82486.6416	1456.9030 1	456.9017	0.0013	0	K.KIIAEKTKIPAVF.K (Ions score 28)
71 – 82665.4109	1328.8073 1	328.8067	0.0006	0	K.IIAEKTKIPAVF.K (Ions score 38)
73 – 82552.3264	1102.6383 1	102.6386 -	0.0003	0	I.AEKTKIPAVF.K (Ions score 33)
74 – 82516.8077	1031.6009 1	031.6015 -	0.0006	0	A.EKTKIPAVF.K (Ions score 41)
74 – 82516.8078	1031.6010 1	031.6015 -	0.0004	0	A.EKTKIPAVF.K (Ions score 25)
74 – 82516.8080	1031.6014 1	031.6015 -	0.0001	0	A.EKTKIPAVF.K (Ions score 35)
74 – 82516.8081	1031.6017 1	031.6015	0.0002	0	A.EKTKIPAVF.K (Ions score 44)
74 – 82516.8082	1031.6019 1	031.6015	0.0004	0	A.EKTKIPAVF.K (Ions score 43)
74 – 82516.8083	1031.6020 1	031.6015	0.0005	0	A.EKTKIPAVF.K (Ions score 50)
74 – 82516.8083	1031.6020 1	031.6015	0.0005	0	A.EKTKIPAVF.K (Ions score 36)
74 – 82516.8083	1031.6020 1	031.6015	0.0005	0	A.EKTKIPAVF.K (Ions score 22)
74 – 82516.8084	1031.6023 1	031.6015	0.0008	0	A.EKTKIPAVF.K (Ions score 50)
74 – 82516.8085	1031.6024 1	031.6015	0.0009	0	A.EKTKIPAVF.K (Ions score 45)
74 – 82516.8085	1031.6025 1	031.6015	0.0010	0	A.EKTKIPAVF.K (Ions score 43)
74 – 83387.5727	1159.6963 1	159.6964 -	0.0001	0	A.EKTKIPAVFK.I (Ions score 23)
75 – 82452.2862	902.5578	902.5589 -	0.0011	0	E.KTKIPAVF.K (Ions score 29)
75 – 82452.2866	902.5586	902.5589 -	0.0003	0	E.KTKIPAVF.K (Ions score 34)
75 – 82452.2866	902.5587	902.5589 -	0.0002	0	E.KTKIPAVF.K (Ions score 31)
76 – 83301.8600	902.5582	902.5589 -	0.0007	0	K.TKIPAVFK.I (Ions score 28)
76 – 83301.8600	902.5582	902.5589 -	0.0007	0	K.TKIPAVFK.I (Ions score 29)
76 – 83301.8602	902.5589	902.5589 -	0.0000	0	K.TKIPAVFK.I (Ions score 38)
77 – 82337.7151	673.4157	673.4163 -	0.0005	0	T.KIPAVF.K (Ions score 32)
77 – 82337.7153	673.4161	673.4163 -	0.0001	0	T.KIPAVF.K (Ions score 23)
81 – 85311.1838	620.3531	620.3533 -	0.0002	0	A.VFKID.A (Ions score 33)
81 –85 311.1839	620.3533	620.3533 -	0.0000	0	A.VFKID.A (Ions score 33)
83 – 87559.3448	558.3376	558.3377 -	0.0001	0	F.KIDAL (Ions score 29)
