# Supplementary material for: An alternative for proteinase K-heat-sensitive protease from fungus Onygena corvina for biotechnology: cloning, engineering, expression, characterization and special application for protein sequencing
Source: Microb Cell Fact. 2020 Jun 24;19:135. doi: 10.1186/s12934-020-01392-3 (PMC7313183; doi:10.1186/s12934-020-01392-3)
Supplement: Supplementary file 16 — Additional file 16. MASCOT Search Results of NHSSP-cleaved BLG. [file 12934_2020_1392_MOESM16_ESM.pdf]

(MATRIX SCIENCE) Mascot Search Results

Protein View

Match to: **BLG** Score: **5255**

Found in search of \\SERVER3\User\Kunden_Projekte\Koller_8375\Further-characterization-part-I\8375_BLG_HL1_1210nl.mgf

Nominal mass (M_r): **18555**; Calculated pI value: **4.83**
NCBI BLAST search of BLG against nr
Unformatted sequence string for pasting into other applications

Fixed modifications: Carbamidomethyl (C)
Variable modifications: Oxidation (M)
No enzyme cleavage specificity
Sequence Coverage: **100%**

Matched peptides shown in **Bold Red**

```
  1 LIVTQTMKGL DIQKVAGTWY SLAMAASDIS LLDAQSAPLR VYVEELKPTP
 51 EGDLEILLQK WENGECAQKK IIAEKTKIPA VFKIDALNEN KVLVLDTDYK
101 KYLLFCMENS AEPEQSLACQ CLVRTPEVDD EALEKFDKAL KALPMHIRLS
151 FNPTQLEEQC HI
```

Sort Peptides By ◉ Residue Number ○ Increasing Mass ○ Decreasing Mass

Start - End	Observed	Mr(expt)	Mr(calc)	Delta	Miss	Sequence
1 - 5	573.3610	572.3537	572.3533	0.0004	0	-.LIVTQ.T (Ions score 26)
1 - 8	467.2752	932.5358	932.5365	-0.0007	0	-.LIVTQTMK.G (Ions score 21)
1 - 8	467.2754	932.5363	932.5365	-0.0002	0	-.LIVTQTMK.G (Ions score 64)
1 - 8	467.2756	932.5366	932.5365	0.0001	0	-.LIVTQTMK.G (Ions score 61)
1 - 10	552.3282	1102.6418	1102.6420	-0.0002	0	-.LIVTQTMKGL.D (Ions score 34)
1 - 10	552.3285	1102.6424	1102.6420	0.0004	0	-.LIVTQTMKGL.D (Ions score 58)
1 - 10	552.3286	1102.6427	1102.6420	0.0007	0	-.LIVTQTMKGL.D (Ions score 61)
1 - 10	552.3287	1102.6429	1102.6420	0.0009	0	-.LIVTQTMKGL.D (Ions score 58)
1 - 10	560.3242	1118.6339	1118.6369	-0.0030	0	-.LIVTQTMKGL.D Oxidation (M) (Ions score 32)
1 - 10	560.3243	1118.6340	1118.6369	-0.0029	0	-.LIVTQTMKGL.D Oxidation (M) (Ions score 28)
1 - 13	730.4138	1458.8131	1458.8116	0.0015	0	-.LIVTQTMKGLDIQ.K (Ions score 73)
1 - 13	730.4141	1458.8136	1458.8116	0.0020	0	-.LIVTQTMKGLDIQ.K (Ions score 51)
1 - 13	730.4142	1458.8139	1458.8116	0.0024	0	-.LIVTQTMKGLDIQ.K (Ions score 21)
1 - 13	730.4142	1458.8139	1458.8116	0.0024	0	-.LIVTQTMKGLDIQ.K (Ions score 80)
1 - 13	730.4144	1458.8142	1458.8116	0.0026	0	-.LIVTQTMKGLDIQ.K (Ions score 21)
1 - 13	730.4144	1458.8142	1458.8116	0.0026	0	-.LIVTQTMKGLDIQ.K (Ions score 22)
1 - 13	730.4144	1458.8142	1458.8116	0.0026	0	-.LIVTQTMKGLDIQ.K (Ions score 20)
1 - 13	730.4145	1458.8144	1458.8116	0.0029	0	-.LIVTQTMKGLDIQ.K (Ions score 94)
1 - 13	730.4145	1458.8144	1458.8116	0.0029	0	-.LIVTQTMKGLDIQ.K (Ions score 59)
1 - 13	738.4110	1474.8075	1474.8065	0.0010	0	-.LIVTQTMKGLDIQ.K Oxidation (M) (Ions score 73)
1 - 13	738.4113	1474.8080	1474.8065	0.0015	0	-.LIVTQTMKGLDIQ.K Oxidation (M) (Ions score 38)
1 - 13	738.4113	1474.8081	1474.8065	0.0016	0	-.LIVTQTMKGLDIQ.K Oxidation (M) (Ions score 74)
1 - 13	738.4113	1474.8081	1474.8065	0.0016	0	-.LIVTQTMKGLDIQ.K Oxidation (M) (Ions score 72)
1 - 13	738.4114	1474.8082	1474.8065	0.0017	0	-.LIVTQTMKGLDIQ.K Oxidation (M) (Ions score 55)
1 - 13	738.4114	1474.8083	1474.8065	0.0018	0	-.LIVTQTMKGLDIQ.K Oxidation (M) (Ions score 76)
1 - 13	738.4116	1474.8086	1474.8065	0.0021	0	-.LIVTQTMKGLDIQ.K Oxidation (M) (Ions score 57)
1 - 13	738.4116	1474.8086	1474.8065	0.0021	0	-.LIVTQTMKGLDIQ.K Oxidation (M) (Ions score 29)
1 - 13	738.4116	1474.8087	1474.8065	0.0022	0	-.LIVTQTMKGLDIQ.K Oxidation (M) (Ions score 61)
1 - 16	586.6790	1757.0150	1757.0120	0.0030	0	-.LIVTQTMKGLDIQKVA.G (Ions score 42)
1 - 16	592.0098	1773.0075	1773.0070	0.0005	0	-.LIVTQTMKGLDIQKVA.G Oxidation (M) (Ions score 50)
1 - 16	592.0098	1773.0077	1773.0070	0.0007	0	-.LIVTQTMKGLDIQKVA.G Oxidation (M) (Ions score 45)
1 - 16	592.0099	1773.0080	1773.0070	0.0011	0	-.LIVTQTMKGLDIQKVA.G Oxidation (M) (Ions score 54)
1 - 16	592.0099	1773.0080	1773.0070	0.0011	0	-.LIVTQTMKGLDIQKVA.G Oxidation (M) (Ions score 22)
1 - 16	887.5118	1773.0091	1773.0070	0.0022	0	-.LIVTQTMKGLDIQKVA.G Oxidation (M) (Ions score 31)
5 - 10	339.1859	676.3573	676.3578	-0.0005	0	T.QTMKGL.D (Ions score 28)
5 - 13	525.2683	1048.5221	1048.5223	-0.0002	0	T.QTMKGLDIQ.K Oxidation (M) (Ions score 24)
5 - 13	525.2685	1048.5224	1048.5223	0.0002	0	T.QTMKGLDIQ.K Oxidation (M) (Ions score 32)
5 - 16	666.3712	1330.7279	1330.7278	0.0001	0	T.QTMKGLDIQKVA.G (Ions score 27)
6 - 13	453.2414	904.4682	904.4688	-0.0006	0	Q.TMKGLDIQ.K (Ions score 34)
6 - 13	453.2414	904.4683	904.4688	-0.0005	0	Q.TMKGLDIQ.K (Ions score 22)
6 - 13	453.2415	904.4684	904.4688	-0.0004	0	Q.TMKGLDIQ.K (Ions score 40)
6 - 13	453.2416	904.4687	904.4688	-0.0001	0	Q.TMKGLDIQ.K (Ions score 31)
6 - 13	453.2417	904.4688	904.4688	0.0000	0	Q.TMKGLDIQ.K (Ions score 27)
6 - 13	453.2417	904.4688	904.4688	0.0001	0	Q.TMKGLDIQ.K (Ions score 46)
6 - 13	453.2417	904.4688	904.4688	0.0001	0	Q.TMKGLDIQ.K (Ions score 42)
6 - 13	453.2418	904.4690	904.4688	0.0002	0	Q.TMKGLDIQ.K (Ions score 44)
6 - 13	453.2419	904.4693	904.4688	0.0006	0	Q.TMKGLDIQ.K (Ions score 45)
6 - 13	461.2388	920.4630	920.4637	-0.0007	0	Q.TMKGLDIQ.K Oxidation (M) (Ions score 28)
6 - 13	461.2388	920.4631	920.4637	-0.0006	0	Q.TMKGLDIQ.K Oxidation (M) (Ions score 39)
6 - 13	461.2390	920.4634	920.4637	-0.0003	0	Q.TMKGLDIQ.K Oxidation (M) (Ions score 30)
6 - 13	461.2391	920.4637	920.4637	-0.0000	0	Q.TMKGLDIQ.K Oxidation (M) (Ions score 40)
6 - 16	602.3417	1202.6688	1202.6692	-0.0005	0	Q.TMKGLDIQKVA.G (Ions score 39)
6 - 16	602.3419	1202.6692	1202.6692	-0.0001	0	Q.TMKGLDIQKVA.G (Ions score 43)
6 - 16	602.3419	1202.6693	1202.6692	0.0000	0	Q.TMKGLDIQKVA.G (Ions score 41)
6 - 18	454.5865	1360.7376	1360.7384	-0.0008	0	Q.TMKGLDIQKVAGT.W (Ions score 33)
9 - 16	422.2502	842.4858	842.4861	-0.0004	0	K.GLDIQKVA.G (Ions score 35)
9 - 16	422.2502	842.4859	842.4861	-0.0002	0	K.GLDIQKVA.G (Ions score 25)
10 - 16	393.7396	785.4646	785.4647	-0.0001	0	G.LDIQKVA.G (Ions score 30)
11 - 16	673.3876	672.3804	672.3806	-0.0003	0	L.DIQKVA.G (Ions score 38)
11 - 16	337.1975	672.3804	672.3806	-0.0002	0	L.DIQKVA.G (Ions score 26)
11 - 16	337.1976	672.3807	672.3806	0.0001	0	L.DIQKVA.G (Ions score 23)
11 - 18	416.2318	830.4491	830.4498	-0.0007	0	L.DIQKVAGT.W (Ions score 30)
11 - 18	416.2321	830.4496	830.4498	-0.0001	0	L.DIQKVAGT.W (Ions score 38)

11 - 22	690.8621	1379.7096	1379.7085	0.0011	0	L.DIQKVAGTWYSL.A (Ions score 58)
11 - 23	726.3803	1450.7461	1450.7456	0.0005	0	L.DIQKVAGTWYSLA.M (Ions score 56)
11 - 23	726.3803	1450.7461	1450.7456	0.0005	0	L.DIQKVAGTWYSLA.M (Ions score 59)
11 - 24	791.9008	1581.7871	1581.7861	0.0010	0	L.DIQKVAGTWYSLAM.A (Ions score 88)
11 - 24	791.9009	1581.7872	1581.7861	0.0011	0	L.DIQKVAGTWYSLAM.A (Ions score 68)
11 - 25	827.4194	1652.8243	1652.8232	0.0011	0	L.DIQKVAGTWYSLAMA.A (Ions score 75)
14 - 22	512.7766	1023.5385	1023.5389	-0.0003	0	Q.KVAGTWYSL.A (Ions score 27)
14 - 22	512.7768	1023.5390	1023.5389	0.0001	0	Q.KVAGTWYSL.A (Ions score 50)
14 - 22	512.7770	1023.5394	1023.5389	0.0005	0	Q.KVAGTWYSL.A (Ions score 50)
14 - 23	548.2949	1094.5753	1094.5760	-0.0007	0	Q.KVAGTWYSLA.M (Ions score 60)
14 - 23	548.2953	1094.5760	1094.5760	0.0000	0	Q.KVAGTWYSLA.M (Ions score 40)
14 - 23	548.2953	1094.5761	1094.5760	0.0001	0	Q.KVAGTWYSLA.M (Ions score 48)
14 - 24	613.8160	1225.6175	1225.6165	0.0010	0	Q.KVAGTWYSLAM.A (Ions score 62)
14 - 24	613.8171	1225.6197	1225.6165	0.0032	0	Q.KVAGTWYSLAM.A (Ions score 56)
14 - 27	728.3672	1454.7198	1454.7227	-0.0029	0	Q.KVAGTWYSLAMAAS.D (Ions score 34)
17 - 22	726.3460	725.3387	725.3384	0.0003	0	A.GTWYSL.A (Ions score 22)
17 - 23	797.3834	796.3761	796.3755	0.0006	0	A.GTWYSLA.M (Ions score 22)
17 - 25	500.2339	998.4533	998.4531	0.0002	0	A.GTWYSLAMA.A (Ions score 52)
19 - 23	639.3137	638.3064	638.3064	-0.0000	0	T.WYSLA.M (Ions score 21)
19 - 23	639.3138	638.3066	638.3064	0.0002	0	T.WYSLA.M (Ions score 20)
19 - 23	639.3140	638.3067	638.3064	0.0004	0	T.WYSLA.M (Ions score 20)
19 - 24	770.3536	769.3464	769.3469	-0.0005	0	T.WYSLAM.A (Ions score 21)
22 - 31	504.2578	1006.5010	1006.5005	0.0005	0	S.LAMAASDISL.L Oxidation (M) (Ions score 22)
23 - 42	1046.5430	2091.0714	2091.0670	0.0044	0	L.AMAASDISLLDAQSAPLRVY.V (Ions score 79)
24 - 35	617.8024	1233.5903	1233.5911	-0.0008	0	A.MAASDISLLDAQ.S (Ions score 43)
24 - 35	617.8027	1233.5909	1233.5911	-0.0002	0	A.MAASDISLLDAQ.S (Ions score 50)
24 - 35	617.8029	1233.5913	1233.5911	0.0002	0	A.MAASDISLLDAQ.S (Ions score 64)
24 - 35	617.8029	1233.5913	1233.5911	0.0002	0	A.MAASDISLLDAQ.S (Ions score 44)
24 - 42	1011.0228	2020.0310	2020.0299	0.0011	0	A.MAASDISLLDAQSAPLRVY.V (Ions score 61)
24 - 42	1011.0231	2020.0316	2020.0299	0.0017	0	A.MAASDISLLDAQSAPLRVY.V (Ions score 40)
24 - 42	1011.0241	2020.0337	2020.0299	0.0038	0	A.MAASDISLLDAQSAPLRVY.V (Ions score 40)
25 - 32	789.4359	788.4286	788.4280	0.0006	0	M.AASDISLL.D (Ions score 23)
25 - 35	552.2824	1102.5503	1102.5506	-0.0003	0	M.AASDISLLDAQ.S (Ions score 54)
25 - 35	552.2826	1102.5506	1102.5506	0.0000	0	M.AASDISLLDAQ.S (Ions score 43)
25 - 35	552.2828	1102.5510	1102.5506	0.0004	0	M.AASDISLLDAQ.S (Ions score 30)
25 - 42	945.5029	1888.9913	1888.9894	0.0019	0	M.AASDISLLDAQSAPLRVY.V (Ions score 48)
25 - 42	945.5030	1888.9914	1888.9894	0.0020	0	M.AASDISLLDAQSAPLRVY.V (Ions score 40)
25 - 42	945.5035	1888.9925	1888.9894	0.0031	0	M.AASDISLLDAQSAPLRVY.V (Ions score 85)
25 - 42	945.5040	1888.9935	1888.9894	0.0041	0	M.AASDISLLDAQSAPLRVY.V (Ions score 59)
26 - 35	516.7640	1031.5135	1031.5135	0.0000	0	A.ASDISLLDAQ.S (Ions score 21)
26 - 35	516.7647	1031.5149	1031.5135	0.0014	0	A.ASDISLLDAQ.S (Ions score 68)
26 - 39	700.8675	1399.7204	1399.7195	0.0010	0	A.ASDISLLDAQSAPL.R (Ions score 21)
26 - 42	909.9846	1817.9547	1817.9523	0.0024	0	A.ASDISLLDAQSAPLRVY.V (Ions score 86)
26 - 42	909.9853	1817.9560	1817.9523	0.0037	0	A.ASDISLLDAQSAPLRVY.V (Ions score 100)
27 - 42	874.4653	1746.9160	1746.9152	0.0008	0	A.SDISLLDAQSAPLRVY.V (Ions score 60)
27 - 42	874.4654	1746.9162	1746.9152	0.0011	0	A.SDISLLDAQSAPLRVY.V (Ions score 42)
27 - 42	874.4656	1746.9166	1746.9152	0.0014	0	A.SDISLLDAQSAPLRVY.V (Ions score 69)
27 - 42	874.4661	1746.9177	1746.9152	0.0025	0	A.SDISLLDAQSAPLRVY.V (Ions score 74)
28 - 32	560.3292	559.3219	559.3217	0.0002	0	S.DISLL.D (Ions score 20)
28 - 35	437.7290	873.4435	873.4444	-0.0009	0	S.DISLLDAQ.S (Ions score 42)
28 - 35	437.7294	873.4443	873.4444	-0.0000	0	S.DISLLDAQ.S (Ions score 36)
28 - 39	621.8327	1241.6509	1241.6503	0.0005	0	S.DISLLDAQSAPL.R (Ions score 39)
28 - 39	621.8327	1241.6509	1241.6503	0.0005	0	S.DISLLDAQSAPL.R (Ions score 43)
28 - 39	621.8330	1241.6515	1241.6503	0.0012	0	S.DISLLDAQSAPL.R (Ions score 27)
28 - 42	830.9488	1659.8830	1659.8831	-0.0001	0	S.DISLLDAQSAPLRVY.V (Ions score 30)
28 - 42	830.9490	1659.8835	1659.8831	0.0004	0	S.DISLLDAQSAPLRVY.V (Ions score 26)
28 - 42	830.9498	1659.8851	1659.8831	0.0020	0	S.DISLLDAQSAPLRVY.V (Ions score 51)
28 - 42	830.9499	1659.8852	1659.8831	0.0021	0	S.DISLLDAQSAPLRVY.V (Ions score 26)
28 - 42	830.9503	1659.8860	1659.8831	0.0028	0	S.DISLLDAQSAPLRVY.V (Ions score 53)
28 - 42	830.9503	1659.8861	1659.8831	0.0029	0	S.DISLLDAQSAPLRVY.V (Ions score 99)
33 - 42	560.2930	1118.5715	1118.5720	-0.0005	0	L.DAQSAPLRVY.V (Ions score 46)
33 - 42	560.2936	1118.5726	1118.5720	0.0006	0	L.DAQSAPLRVY.V (Ions score 49)
33 - 42	560.2936	1118.5726	1118.5720	0.0006	0	L.DAQSAPLRVY.V (Ions score 20)
33 - 42	560.2936	1118.5726	1118.5720	0.0006	0	L.DAQSAPLRVY.V (Ions score 49)
33 - 42	560.2937	1118.5729	1118.5720	0.0009	0	L.DAQSAPLRVY.V (Ions score 46)
33 - 42	560.2939	1118.5732	1118.5720	0.0012	0	L.DAQSAPLRVY.V (Ions score 46)
33 - 42	560.2941	1118.5737	1118.5720	0.0017	0	L.DAQSAPLRVY.V (Ions score 37)
33 - 42	1119.5812	1118.5739	1118.5720	0.0019	0	L.DAQSAPLRVY.V (Ions score 27)
33 - 42	560.2943	1118.5739	1118.5720	0.0020	0	L.DAQSAPLRVY.V (Ions score 43)
33 - 42	560.2945	1118.5744	1118.5720	0.0024	0	L.DAQSAPLRVY.V (Ions score 39)
34 - 42	502.7796	1003.5447	1003.5450	-0.0003	0	D.AQSAPLRVY.V (Ions score 45)
34 - 42	502.7799	1003.5452	1003.5450	0.0002	0	D.AQSAPLRVY.V (Ions score 22)
36 - 42	403.2317	804.4489	804.4494	-0.0005	0	Q.SAPLRVY.V (Ions score 34)
36 - 42	403.2320	804.4494	804.4494	0.0000	0	Q.SAPLRVY.V (Ions score 26)
36 - 42	403.2320	804.4494	804.4494	0.0001	0	Q.SAPLRVY.V (Ions score 32)
36 - 42	403.2320	804.4494	804.4494	0.0001	0	Q.SAPLRVY.V (Ions score 23)
36 - 42	403.2320	804.4495	804.4494	0.0001	0	Q.SAPLRVY.V (Ions score 22)
36 - 42	403.2320	804.4495	804.4494	0.0001	0	Q.SAPLRVY.V (Ions score 23)
36 - 42	403.2321	804.4496	804.4494	0.0002	0	Q.SAPLRVY.V (Ions score 33)
36 - 42	403.2321	804.4497	804.4494	0.0004	0	Q.SAPLRVY.V (Ions score 23)
36 - 42	403.2321	804.4497	804.4494	0.0004	0	Q.SAPLRVY.V (Ions score 30)
36 - 42	403.2322	804.4499	804.4494	0.0005	0	Q.SAPLRVY.V (Ions score 23)
36 - 42	403.2322	804.4499	804.4494	0.0006	0	Q.SAPLRVY.V (Ions score 35)
36 - 42	403.2323	804.4500	804.4494	0.0006	0	Q.SAPLRVY.V (Ions score 34)
36 - 42	403.2324	804.4502	804.4494	0.0008	0	Q.SAPLRVY.V (Ions score 37)
36 - 42	403.2325	804.4505	804.4494	0.0012	0	Q.SAPLRVY.V (Ions score 34)
37 - 42	359.7155	717.4165	717.4173	-0.0008	0	S.APLRVY.V (Ions score 33)
37 - 42	359.7159	717.4171	717.4173	-0.0002	0	S.APLRVY.V (Ions score 32)
37 - 42	359.7159	717.4172	717.4173	-0.0001	0	S.APLRVY.V (Ions score 36)
38 - 42	324.1967	646.3788	646.3802	-0.0015	0	A.PLRVY.V (Ions score 25)
43 - 51	521.2767	1040.5388	1040.5390	-0.0002	0	Y.VEELKPTPE.G (Ions score 50)
43 - 51	521.2768	1040.5390	1040.5390	0.0001	0	Y.VEELKPTPE.G (Ions score 50)
43 - 54	663.8435	1325.6725	1325.6714	0.0010	0	Y.VEELKPTPEGDL.E (Ions score 29)

43 - 57	841.4316	1680.8486	1680.8821	-0.0335	0	Y.VEELKPTPEGDLEIL.L	(Ions score 31)
43 - 57	841.4316	1680.8487	1680.8821	-0.0334	0	Y.VEELKPTPEGDLEIL.L	(Ions score 30)
43 - 57	841.4471	1680.8797	1680.8821	-0.0024	0	Y.VEELKPTPEGDLEIL.L	(Ions score 30)
43 - 57	841.4478	1680.8811	1680.8821	-0.0010	0	Y.VEELKPTPEGDLEIL.L	(Ions score 39)
43 - 57	841.4480	1680.8814	1680.8821	-0.0007	0	Y.VEELKPTPEGDLEIL.L	(Ions score 26)
43 - 57	841.4481	1680.8817	1680.8821	-0.0004	0	Y.VEELKPTPEGDLEIL.L	(Ions score 43)
43 - 57	841.4481	1680.8817	1680.8821	-0.0004	0	Y.VEELKPTPEGDLEIL.L	(Ions score 20)
43 - 57	841.4482	1680.8819	1680.8821	-0.0002	0	Y.VEELKPTPEGDLEIL.L	(Ions score 25)
43 - 57	841.4483	1680.8821	1680.8821	-0.0001	0	Y.VEELKPTPEGDLEIL.L	(Ions score 39)
43 - 57	841.4484	1680.8823	1680.8821	0.0002	0	Y.VEELKPTPEGDLEIL.L	(Ions score 31)
43 - 57	841.4485	1680.8825	1680.8821	0.0004	0	Y.VEELKPTPEGDLEIL.L	(Ions score 30)
43 - 57	841.4486	1680.8827	1680.8821	0.0005	0	Y.VEELKPTPEGDLEIL.L	(Ions score 25)
43 - 57	841.4487	1680.8828	1680.8821	0.0007	0	Y.VEELKPTPEGDLEIL.L	(Ions score 45)
43 - 57	841.4487	1680.8828	1680.8821	0.0007	0	Y.VEELKPTPEGDLEIL.L	(Ions score 27)
43 - 57	841.4487	1680.8829	1680.8821	0.0008	0	Y.VEELKPTPEGDLEIL.L	(Ions score 34)
43 - 57	841.4487	1680.8829	1680.8821	0.0008	0	Y.VEELKPTPEGDLEIL.L	(Ions score 32)
43 - 57	841.4488	1680.8830	1680.8821	0.0009	0	Y.VEELKPTPEGDLEIL.L	(Ions score 57)
43 - 57	841.4489	1680.8832	1680.8821	0.0010	0	Y.VEELKPTPEGDLEIL.L	(Ions score 24)
43 - 57	841.4489	1680.8832	1680.8821	0.0010	0	Y.VEELKPTPEGDLEIL.L	(Ions score 36)
43 - 57	841.4489	1680.8833	1680.8821	0.0012	0	Y.VEELKPTPEGDLEIL.L	(Ions score 42)
43 - 57	841.4489	1680.8833	1680.8821	0.0012	0	Y.VEELKPTPEGDLEIL.L	(Ions score 43)
43 - 57	841.4489	1680.8833	1680.8821	0.0012	0	Y.VEELKPTPEGDLEIL.L	(Ions score 32)
43 - 57	841.4490	1680.8834	1680.8821	0.0013	0	Y.VEELKPTPEGDLEIL.L	(Ions score 56)
43 - 57	841.4490	1680.8834	1680.8821	0.0013	0	Y.VEELKPTPEGDLEIL.L	(Ions score 48)
43 - 57	841.4490	1680.8834	1680.8821	0.0013	0	Y.VEELKPTPEGDLEIL.L	(Ions score 33)
43 - 57	841.4490	1680.8834	1680.8821	0.0013	0	Y.VEELKPTPEGDLEIL.L	(Ions score 43)
43 - 57	841.4490	1680.8834	1680.8821	0.0013	0	Y.VEELKPTPEGDLEIL.L	(Ions score 35)
43 - 57	841.4490	1680.8834	1680.8821	0.0013	0	Y.VEELKPTPEGDLEIL.L	(Ions score 22)
43 - 57	841.4490	1680.8835	1680.8821	0.0014	0	Y.VEELKPTPEGDLEIL.L	(Ions score 55)
43 - 57	841.4490	1680.8835	1680.8821	0.0014	0	Y.VEELKPTPEGDLEIL.L	(Ions score 27)
43 - 57	841.4490	1680.8835	1680.8821	0.0014	0	Y.VEELKPTPEGDLEIL.L	(Ions score 26)
43 - 57	841.4490	1680.8835	1680.8821	0.0014	0	Y.VEELKPTPEGDLEIL.L	(Ions score 30)
43 - 57	841.4491	1680.8836	1680.8821	0.0015	0	Y.VEELKPTPEGDLEIL.L	(Ions score 64)
43 - 57	841.4492	1680.8839	1680.8821	0.0018	0	Y.VEELKPTPEGDLEIL.L	(Ions score 59)
43 - 57	841.4492	1680.8839	1680.8821	0.0018	0	Y.VEELKPTPEGDLEIL.L	(Ions score 39)
43 - 57	841.4493	1680.8840	1680.8821	0.0019	0	Y.VEELKPTPEGDLEIL.L	(Ions score 51)
43 - 57	841.4493	1680.8840	1680.8821	0.0019	0	Y.VEELKPTPEGDLEIL.L	(Ions score 57)
43 - 57	841.4493	1680.8840	1680.8821	0.0019	0	Y.VEELKPTPEGDLEIL.L	(Ions score 28)
43 - 57	841.4493	1680.8841	1680.8821	0.0020	0	Y.VEELKPTPEGDLEIL.L	(Ions score 21)
43 - 57	841.4493	1680.8841	1680.8821	0.0020	0	Y.VEELKPTPEGDLEIL.L	(Ions score 26)
43 - 57	841.4494	1680.8843	1680.8821	0.0021	0	Y.VEELKPTPEGDLEIL.L	(Ions score 51)
43 - 57	841.4495	1680.8844	1680.8821	0.0022	0	Y.VEELKPTPEGDLEIL.L	(Ions score 66)
43 - 57	841.4495	1680.8844	1680.8821	0.0022	0	Y.VEELKPTPEGDLEIL.L	(Ions score 41)
43 - 57	841.4503	1680.8860	1680.8821	0.0038	0	Y.VEELKPTPEGDLEIL.L	(Ions score 38)
43 - 57	841.4504	1680.8862	1680.8821	0.0041	0	Y.VEELKPTPEGDLEIL.L	(Ions score 64)
43 - 57	841.4504	1680.8863	1680.8821	0.0042	0	Y.VEELKPTPEGDLEIL.L	(Ions score 48)
43 - 57	841.4506	1680.8866	1680.8821	0.0044	0	Y.VEELKPTPEGDLEIL.L	(Ions score 52)
43 - 57	841.4509	1680.8873	1680.8821	0.0052	0	Y.VEELKPTPEGDLEIL.L	(Ions score 46)
43 - 57	841.4512	1680.8879	1680.8821	0.0058	0	Y.VEELKPTPEGDLEIL.L	(Ions score 49)
43 - 57	841.4515	1680.8884	1680.8821	0.0063	0	Y.VEELKPTPEGDLEIL.L	(Ions score 67)
43 - 57	841.4517	1680.8889	1680.8821	0.0068	0	Y.VEELKPTPEGDLEIL.L	(Ions score 55)
43 - 59	962.0208	1922.0270	1922.0248	0.0022	0	Y.VEELKPTPEGDLEILLQ.K	(Ions score 48)
43 - 59	962.0209	1922.0272	1922.0248	0.0024	0	Y.VEELKPTPEGDLEILLQ.K	(Ions score 37)
43 - 59	962.0209	1922.0273	1922.0248	0.0026	0	Y.VEELKPTPEGDLEILLQ.K	(Ions score 23)
43 - 59	962.0211	1922.0277	1922.0248	0.0029	0	Y.VEELKPTPEGDLEILLQ.K	(Ions score 59)
43 - 59	962.0213	1922.0280	1922.0248	0.0033	0	Y.VEELKPTPEGDLEILLQ.K	(Ions score 33)
43 - 59	962.0214	1922.0282	1922.0248	0.0034	0	Y.VEELKPTPEGDLEILLQ.K	(Ions score 60)
43 - 59	962.0214	1922.0283	1922.0248	0.0035	0	Y.VEELKPTPEGDLEILLQ.K	(Ions score 73)
43 - 59	962.0224	1922.0302	1922.0248	0.0055	0	Y.VEELKPTPEGDLEILLQ.K	(Ions score 57)
45 - 57	727.3936	1452.7726	1452.7711	0.0014	0	E.ELKPTPEGDLEIL.L	(Ions score 65)
45 - 57	727.3937	1452.7729	1452.7711	0.0018	0	E.ELKPTPEGDLEIL.L	(Ions score 21)
45 - 57	727.3941	1452.7737	1452.7711	0.0025	0	E.ELKPTPEGDLEIL.L	(Ions score 59)
45 - 57	727.3943	1452.7741	1452.7711	0.0030	0	E.ELKPTPEGDLEIL.L	(Ions score 33)
45 - 57	727.3950	1452.7754	1452.7711	0.0042	0	E.ELKPTPEGDLEIL.L	(Ions score 51)
46 - 57	662.8721	1323.7296	1323.7285	0.0010	0	E.LKPTPEGDLEIL.L	(Ions score 40)
46 - 57	662.8721	1323.7296	1323.7285	0.0010	0	E.LKPTPEGDLEIL.L	(Ions score 39)
46 - 57	662.8721	1323.7297	1323.7285	0.0012	0	E.LKPTPEGDLEIL.L	(Ions score 39)
46 - 59	783.4432	1564.8718	1564.8712	0.0006	0	E.LKPTPEGDLEILLQ.K	(Ions score 34)
46 - 59	783.4434	1564.8723	1564.8712	0.0011	0	E.LKPTPEGDLEILLQ.K	(Ions score 44)
46 - 59	783.4437	1564.8728	1564.8712	0.0016	0	E.LKPTPEGDLEILLQ.K	(Ions score 63)
46 - 59	783.4457	1564.8769	1564.8712	0.0057	0	E.LKPTPEGDLEILLQ.K	(Ions score 57)
52 - 57	659.3608	658.3536	658.3537	-0.0002	0	E.GDLEIL.L	(Ions score 27)
58 - 73	639.3404	1914.9994	1914.9985	0.0008	0	L.LQKWENGECAQKKIIA.E	(Ions score 33)
60 - 73	558.9590	1673.8551	1673.8559	-0.0007	0	Q.KWENGECAQKKIIA.E	(Ions score 22)
62 - 73	454.2345	1359.6816	1359.6816	-0.0000	0	W.ENGECAQKKIIA.E	(Ions score 21)
70 - 82	486.6409	1456.9010	1456.9017	-0.0007	0	K.KIIAEKTKIPAVF.K	(Ions score 38)
70 - 82	486.6411	1456.9014	1456.9017	-0.0003	0	K.KIIAEKTKIPAVF.K	(Ions score 24)
70 - 82	486.6411	1456.9016	1456.9017	-0.0001	0	K.KIIAEKTKIPAVF.K	(Ions score 27)
70 - 82	486.6416	1456.9030	1456.9017	0.0013	0	K.KIIAEKTKIPAVF.K	(Ions score 28)
71 - 82	665.4109	1328.8073	1328.8067	0.0006	0	K.IIAEKTKIPAVF.K	(Ions score 38)
73 - 82	552.3264	1102.6383	1102.6386	-0.0003	0	I.AEKTKIPAVF.K	(Ions score 33)
74 - 82	516.8077	1031.6009	1031.6015	-0.0006	0	A.EKTKIPAVF.K	(Ions score 41)
74 - 82	516.8078	1031.6010	1031.6015	-0.0004	0	A.EKTKIPAVF.K	(Ions score 25)
74 - 82	516.8080	1031.6014	1031.6015	-0.0001	0	A.EKTKIPAVF.K	(Ions score 35)
74 - 82	516.8081	1031.6017	1031.6015	0.0002	0	A.EKTKIPAVF.K	(Ions score 44)
74 - 82	516.8082	1031.6019	1031.6015	0.0004	0	A.EKTKIPAVF.K	(Ions score 43)
74 - 82	516.8083	1031.6020	1031.6015	0.0005	0	A.EKTKIPAVF.K	(Ions score 50)
74 - 82	516.8083	1031.6020	1031.6015	0.0005	0	A.EKTKIPAVF.K	(Ions score 36)
74 - 82	516.8083	1031.6020	1031.6015	0.0005	0	A.EKTKIPAVF.K	(Ions score 22)
74 - 82	516.8084	1031.6023	1031.6015	0.0008	0	A.EKTKIPAVF.K	(Ions score 50)
74 - 82	516.8085	1031.6024	1031.6015	0.0009	0	A.EKTKIPAVF.K	(Ions score 45)
74 - 82	516.8085	1031.6025	1031.6015	0.0010	0	A.EKTKIPAVF.K	(Ions score 43)
74 - 83	387.5727	1159.6963	1159.6964	-0.0001	0	A.EKTKIPAVFK.I	(Ions score 23)

75 - 82	452.2862	902.5578	902.5589	-0.0011	0	E.KTKIPAVF.K	(Ions score 29)
75 - 82	452.2866	902.5586	902.5589	-0.0003	0	E.KTKIPAVF.K	(Ions score 34)
75 - 82	452.2866	902.5587	902.5589	-0.0002	0	E.KTKIPAVF.K	(Ions score 31)
76 - 83	301.8600	902.5582	902.5589	-0.0007	0	K.TKIPAVFK.I	(Ions score 28)
76 - 83	301.8600	902.5582	902.5589	-0.0007	0	K.TKIPAVFK.I	(Ions score 29)
76 - 83	301.8602	902.5589	902.5589	-0.0000	0	K.TKIPAVFK.I	(Ions score 38)
77 - 82	337.7151	673.4157	673.4163	-0.0005	0	T.KIPAVF.K	(Ions score 32)
77 - 82	337.7153	673.4161	673.4163	-0.0001	0	T.KIPAVF.K	(Ions score 23)
81 - 85	311.1838	620.3531	620.3533	-0.0002	0	A.VFKID.A	(Ions score 33)
81 - 85	311.1839	620.3533	620.3533	-0.0000	0	A.VFKID.A	(Ions score 33)
83 - 87	559.3448	558.3376	558.3377	-0.0001	0	F.KIDAL.N	(Ions score 29)
83 - 93	419.5782	1255.7127	1255.7135	-0.0008	0	F.KIDALNENKVL.V	(Ions score 33)
83 - 93	419.5783	1255.7130	1255.7135	-0.0005	0	F.KIDALNENKVL.V	(Ions score 36)
83 - 93	628.8639	1255.7132	1255.7135	-0.0003	0	F.KIDALNENKVL.V	(Ions score 48)
83 - 93	628.8641	1255.7137	1255.7135	0.0002	0	F.KIDALNENKVL.V	(Ions score 37)
83 - 93	419.5786	1255.7138	1255.7135	0.0003	0	F.KIDALNENKVL.V	(Ions score 27)
83 - 93	628.8643	1255.7141	1255.7135	0.0005	0	F.KIDALNENKVL.V	(Ions score 51)
83 - 93	628.8644	1255.7143	1255.7135	0.0008	0	F.KIDALNENKVL.V	(Ions score 41)
83 - 95	490.2960	1467.8662	1467.8660	0.0002	0	F.KIDALNENKVLVL.D	(Ions score 30)
83 - 95	490.2962	1467.8668	1467.8660	0.0008	0	F.KIDALNENKVLVL.D	(Ions score 34)
83 - 95	734.9411	1467.8677	1467.8660	0.0016	0	F.KIDALNENKVLVL.D	(Ions score 77)
86 - 93	450.7610	899.5074	899.5076	-0.0002	0	D.ALNENKVL.V	(Ions score 35)
86 - 93	450.7611	899.5077	899.5076	0.0001	0	D.ALNENKVL.V	(Ions score 37)
86 - 93	450.7613	899.5080	899.5076	0.0004	0	D.ALNENKVL.V	(Ions score 40)
86 - 93	450.7614	899.5082	899.5076	0.0006	0	D.ALNENKVL.V	(Ions score 41)
86 - 93	450.7614	899.5082	899.5076	0.0006	0	D.ALNENKVL.V	(Ions score 39)
86 - 93	450.7614	899.5083	899.5076	0.0008	0	D.ALNENKVL.V	(Ions score 35)
86 - 93	450.7617	899.5088	899.5076	0.0012	0	D.ALNENKVL.V	(Ions score 54)
86 - 95	556.8375	1111.6604	1111.6600	0.0003	0	D.ALNENKVLVL.D	(Ions score 32)
86 - 95	556.8375	1111.6605	1111.6600	0.0004	0	D.ALNENKVLVL.D	(Ions score 30)
88 - 93	358.7002	715.3858	715.3864	-0.0006	0	L.NENKVL.V	(Ions score 21)
88 - 93	358.7003	715.3860	715.3864	-0.0005	0	L.NENKVL.V	(Ions score 23)
88 - 93	358.7003	715.3860	715.3864	-0.0004	0	L.NENKVL.V	(Ions score 24)
88 - 93	358.7003	715.3860	715.3864	-0.0004	0	L.NENKVL.V	(Ions score 20)
88 - 93	358.7004	715.3863	715.3864	-0.0001	0	L.NENKVL.V	(Ions score 21)
88 - 93	358.7007	715.3869	715.3864	0.0005	0	L.NENKVL.V	(Ions score 22)
88 - 95	464.7766	927.5387	927.5389	-0.0002	0	L.NENKVLVL.D	(Ions score 31)
88 - 95	464.7769	927.5393	927.5389	0.0004	0	L.NENKVLVL.D	(Ions score 32)
88 - 95	464.7771	927.5396	927.5389	0.0007	0	L.NENKVLVL.D	(Ions score 42)
88 - 95	464.7772	927.5398	927.5389	0.0010	0	L.NENKVLVL.D	(Ions score 41)
88 - 95	464.7772	927.5399	927.5389	0.0010	0	L.NENKVLVL.D	(Ions score 36)
94 - 102	382.2006	1143.5800	1143.5812	-0.0011	0	L.VLDTDYKKY.L	(Ions score 25)
94 - 102	572.7976	1143.5807	1143.5812	-0.0005	0	L.VLDTDYKKY.L	(Ions score 54)
94 - 102	382.2009	1143.5810	1143.5812	-0.0002	0	L.VLDTDYKKY.L	(Ions score 36)
94 - 102	382.2010	1143.5812	1143.5812	0.0001	0	L.VLDTDYKKY.L	(Ions score 28)
94 - 102	572.7981	1143.5816	1143.5812	0.0005	0	L.VLDTDYKKY.L	(Ions score 21)
94 - 102	572.7982	1143.5818	1143.5812	0.0006	0	L.VLDTDYKKY.L	(Ions score 55)
94 - 102	382.2014	1143.5824	1143.5812	0.0013	0	L.VLDTDYKKY.L	(Ions score 34)
94 - 103	629.3398	1256.6651	1256.6652	-0.0001	0	L.VLDTDYKKYL.L	(Ions score 32)
94 - 103	629.3400	1256.6655	1256.6652	0.0003	0	L.VLDTDYKKYL.L	(Ions score 42)
94 - 103	629.3401	1256.6656	1256.6652	0.0004	0	L.VLDTDYKKYL.L	(Ions score 54)
94 - 103	419.8960	1256.6663	1256.6652	0.0010	0	L.VLDTDYKKYL.L	(Ions score 22)
94 - 103	629.3406	1256.6666	1256.6652	0.0014	0	L.VLDTDYKKYL.L	(Ions score 53)
94 - 103	419.8962	1256.6669	1256.6652	0.0017	0	L.VLDTDYKKYL.L	(Ions score 23)
94 - 103	629.3408	1256.6671	1256.6652	0.0019	0	L.VLDTDYKKYL.L	(Ions score 35)
94 - 103	419.8965	1256.6675	1256.6652	0.0023	0	L.VLDTDYKKYL.L	(Ions score 28)
94 - 103	419.8965	1256.6676	1256.6652	0.0024	0	L.VLDTDYKKYL.L	(Ions score 25)
94 - 103	419.8969	1256.6687	1256.6652	0.0035	0	L.VLDTDYKKYL.L	(Ions score 29)
94 - 104	457.5900	1369.7481	1369.7493	-0.0012	0	L.VLDTDYKKYLL.F	(Ions score 22)
94 - 104	457.5902	1369.7487	1369.7493	-0.0006	0	L.VLDTDYKKYLL.F	(Ions score 24)
94 - 104	457.5902	1369.7488	1369.7493	-0.0005	0	L.VLDTDYKKYLL.F	(Ions score 21)
94 - 104	457.5904	1369.7494	1369.7493	0.0002	0	L.VLDTDYKKYLL.F	(Ions score 25)
94 - 104	457.5905	1369.7495	1369.7493	0.0003	0	L.VLDTDYKKYLL.F	(Ions score 32)
94 - 104	457.5906	1369.7499	1369.7493	0.0006	0	L.VLDTDYKKYLL.F	(Ions score 32)
94 - 104	685.8823	1369.7500	1369.7493	0.0007	0	L.VLDTDYKKYLL.F	(Ions score 32)
94 - 104	685.8824	1369.7502	1369.7493	0.0009	0	L.VLDTDYKKYLL.F	(Ions score 45)
94 - 104	457.5907	1369.7503	1369.7493	0.0010	0	L.VLDTDYKKYLL.F	(Ions score 20)
94 - 104	685.8826	1369.7507	1369.7493	0.0014	0	L.VLDTDYKKYLL.F	(Ions score 43)
94 - 105	506.6129	1516.8169	1516.8177	-0.0008	0	L.VLDTDYKKYLLF.C	(Ions score 20)
94 - 105	506.6130	1516.8172	1516.8177	-0.0005	0	L.VLDTDYKKYLLF.C	(Ions score 23)
94 - 105	506.6130	1516.8173	1516.8177	-0.0004	0	L.VLDTDYKKYLLF.C	(Ions score 24)
94 - 105	506.6131	1516.8176	1516.8177	-0.0001	0	L.VLDTDYKKYLLF.C	(Ions score 23)
94 - 105	506.6131	1516.8176	1516.8177	-0.0001	0	L.VLDTDYKKYLLF.C	(Ions score 24)
94 - 105	759.4165	1516.8185	1516.8177	0.0008	0	L.VLDTDYKKYLLF.C	(Ions score 41)
94 - 105	506.6134	1516.8185	1516.8177	0.0008	0	L.VLDTDYKKYLLF.C	(Ions score 25)
94 - 105	759.4166	1516.8186	1516.8177	0.0009	0	L.VLDTDYKKYLLF.C	(Ions score 22)
94 - 105	759.4167	1516.8188	1516.8177	0.0011	0	L.VLDTDYKKYLLF.C	(Ions score 40)
94 - 105	759.4167	1516.8188	1516.8177	0.0011	0	L.VLDTDYKKYLLF.C	(Ions score 31)
94 - 105	506.6137	1516.8193	1516.8177	0.0016	0	L.VLDTDYKKYLLF.C	(Ions score 24)
94 - 105	759.4169	1516.8193	1516.8177	0.0016	0	L.VLDTDYKKYLLF.C	(Ions score 40)
94 - 105	759.4169	1516.8193	1516.8177	0.0016	0	L.VLDTDYKKYLLF.C	(Ions score 31)
94 - 105	506.6137	1516.8194	1516.8177	0.0017	0	L.VLDTDYKKYLLF.C	(Ions score 20)
94 - 105	759.4173	1516.8200	1516.8177	0.0024	0	L.VLDTDYKKYLLF.C	(Ions score 32)
94 - 105	759.4174	1516.8203	1516.8177	0.0026	0	L.VLDTDYKKYLLF.C	(Ions score 34)
94 - 105	506.6142	1516.8208	1516.8177	0.0031	0	L.VLDTDYKKYLLF.C	(Ions score 22)
94 - 106	559.9572	1676.8496	1676.8483	0.0013	0	L.VLDTDYKKYLLFC.M	(Ions score 30)
94 - 106	559.9572	1676.8496	1676.8483	0.0013	0	L.VLDTDYKKYLLFC.M	(Ions score 27)
94 - 106	839.4338	1676.8530	1676.8483	0.0047	0	L.VLDTDYKKYLLFC.M	(Ions score 24)
94 - 106	839.4357	1676.8569	1676.8483	0.0086	0	L.VLDTDYKKYLLFC.M	(Ions score 34)
96 - 102	466.7215	931.4285	931.4287	-0.0002	0	L.DTDYKKY.L	(Ions score 31)
96 - 103	349.1779	1044.5117	1044.5128	-0.0010	0	L.DTDYKKYL.L	(Ions score 25)
96 - 103	523.2633	1044.5121	1044.5128	-0.0007	0	L.DTDYKKYL.L	(Ions score 22)
96 - 103	523.2634	1044.5122	1044.5128	-0.0006	0	L.DTDYKKYL.L	(Ions score 21)

96 - 103	523.2636	1044.5125	1044.5128	-0.0002	0	L.DTDYKKYL.L	(Ions score 21)
96 - 103	523.2638	1044.5130	1044.5128	0.0003	0	L.DTDYKKYL.L	(Ions score 21)
96 - 103	523.2639	1044.5133	1044.5128	0.0005	0	L.DTDYKKYL.L	(Ions score 27)
96 - 103	523.2639	1044.5133	1044.5128	0.0005	0	L.DTDYKKYL.L	(Ions score 24)
96 - 103	523.2640	1044.5135	1044.5128	0.0008	0	L.DTDYKKYL.L	(Ions score 25)
96 - 103	1045.5209	1044.5136	1044.5128	0.0008	0	L.DTDYKKYL.L	(Ions score 39)
96 - 103	523.2642	1044.5138	1044.5128	0.0010	0	L.DTDYKKYL.L	(Ions score 28)
96 - 103	523.2643	1044.5141	1044.5128	0.0014	0	L.DTDYKKYL.L	(Ions score 28)
96 - 104	579.8058	1157.5971	1157.5968	0.0003	0	L.DTDYKKYLL.F	(Ions score 40)
96 - 104	579.8060	1157.5974	1157.5968	0.0006	0	L.DTDYKKYLL.F	(Ions score 36)
96 - 104	579.8064	1157.5982	1157.5968	0.0014	0	L.DTDYKKYLL.F	(Ions score 39)
96 - 105	653.3173	1304.6200	1304.6652	-0.0453	0	L.DTDYKKYLLF.C	(Ions score 25)
96 - 105	653.3370	1304.6594	1304.6652	-0.0058	0	L.DTDYKKYLLF.C	(Ions score 24)
96 - 105	653.3403	1304.6660	1304.6652	0.0008	0	L.DTDYKKYLLF.C	(Ions score 34)
96 - 105	653.3403	1304.6660	1304.6652	0.0008	0	L.DTDYKKYLLF.C	(Ions score 28)
96 - 105	653.3403	1304.6661	1304.6652	0.0009	0	L.DTDYKKYLLF.C	(Ions score 29)
96 - 105	653.3408	1304.6670	1304.6652	0.0017	0	L.DTDYKKYLLF.C	(Ions score 23)
96 - 105	653.3410	1304.6675	1304.6652	0.0022	0	L.DTDYKKYLLF.C	(Ions score 21)
96 - 105	653.3413	1304.6681	1304.6652	0.0028	0	L.DTDYKKYLLF.C	(Ions score 36)
96 - 105	653.3414	1304.6682	1304.6652	0.0030	0	L.DTDYKKYLLF.C	(Ions score 36)
96 - 105	653.3441	1304.6737	1304.6652	0.0085	0	L.DTDYKKYLLF.C	(Ions score 25)
96 - 106	733.3554	1464.6963	1464.6959	0.0004	0	L.DTDYKKYLLFC.M	(Ions score 42)
96 - 106	733.3557	1464.6968	1464.6959	0.0009	0	L.DTDYKKYLLFC.M	(Ions score 37)
96 - 106	733.3557	1464.6969	1464.6959	0.0010	0	L.DTDYKKYLLFC.M	(Ions score 38)
99 - 105	487.7894	973.5642	973.5636	0.0006	0	D.YKKYLLF.C	(Ions score 41)
99 - 105	487.7894	973.5642	973.5636	0.0006	0	D.YKKYLLF.C	(Ions score 29)
99 - 105	487.7894	973.5643	973.5636	0.0007	0	D.YKKYLLF.C	(Ions score 28)
105 - 118	806.8350	1611.6554	1611.6545	0.0009	0	L.FCMENSAEPEQSLA.C	(Ions score 42)
105 - 118	806.8350	1611.6555	1611.6545	0.0010	0	L.FCMENSAEPEQSLA.C	(Ions score 45)
105 - 118	806.8353	1611.6561	1611.6545	0.0016	0	L.FCMENSAEPEQSLA.C	(Ions score 28)
105 - 118	806.8356	1611.6567	1611.6545	0.0022	0	L.FCMENSAEPEQSLA.C	(Ions score 23)
106 - 118	733.3010	1464.5874	1464.5861	0.0013	0	F.CMENSAEPEQSLA.C	(Ions score 46)
106 - 118	733.3011	1464.5876	1464.5861	0.0016	0	F.CMENSAEPEQSLA.C	(Ions score 36)
106 - 118	733.3018	1464.5890	1464.5861	0.0029	0	F.CMENSAEPEQSLA.C	(Ions score 54)
106 - 118	733.3019	1464.5893	1464.5861	0.0033	0	F.CMENSAEPEQSLA.C	(Ions score 63)
106 - 120	877.3458	1752.6771	1752.6753	0.0018	0	F.CMENSAEPEQSLACQ.C	(Ions score 40)
107 - 118	653.2844	1304.5542	1304.5554	-0.0012	0	C.MENSAEPEQSLA.C	(Ions score 28)
107 - 118	653.2847	1304.5548	1304.5554	-0.0006	0	C.MENSAEPEQSLA.C	(Ions score 28)
108 - 118	587.7646	1173.5147	1173.5149	-0.0002	0	M.ENSAEPEQSLA.C	(Ions score 47)
108 - 118	587.7651	1173.5157	1173.5149	0.0008	0	M.ENSAEPEQSLA.C	(Ions score 51)
108 - 118	587.7653	1173.5160	1173.5149	0.0010	0	M.ENSAEPEQSLA.C	(Ions score 39)
108 - 118	587.7653	1173.5160	1173.5149	0.0010	0	M.ENSAEPEQSLA.C	(Ions score 52)
108 - 118	587.7656	1173.5166	1173.5149	0.0017	0	M.ENSAEPEQSLA.C	(Ions score 43)
111 - 118	422.7058	843.3969	843.3974	-0.0004	0	S.AEPEQSLA.C	(Ions score 38)
111 - 118	422.7058	843.3971	843.3974	-0.0003	0	S.AEPEQSLA.C	(Ions score 42)
119 - 123	679.2902	678.2829	678.2829	0.0000	0	A.CQCLV.R	(Ions score 25)
119 - 136	1105.0151	2208.0157	2208.0191	-0.0033	0	A.CQCLVRTPEVDDEALEKF.D	(Ions score 64)
119 - 136	737.0134	2208.0183	2208.0191	-0.0008	0	A.CQCLVRTPEVDDEALEKF.D	(Ions score 48)
119 - 136	1105.0167	2208.0189	2208.0191	-0.0002	0	A.CQCLVRTPEVDDEALEKF.D	(Ions score 66)
119 - 136	737.0139	2208.0197	2208.0191	0.0007	0	A.CQCLVRTPEVDDEALEKF.D	(Ions score 59)
119 - 136	737.0141	2208.0205	2208.0191	0.0014	0	A.CQCLVRTPEVDDEALEKF.D	(Ions score 29)
119 - 136	737.0142	2208.0208	2208.0191	0.0018	0	A.CQCLVRTPEVDDEALEKF.D	(Ions score 52)
119 - 136	737.0143	2208.0210	2208.0191	0.0020	0	A.CQCLVRTPEVDDEALEKF.D	(Ions score 45)
119 - 136	737.0143	2208.0212	2208.0191	0.0021	0	A.CQCLVRTPEVDDEALEKF.D	(Ions score 47)
119 - 136	737.0144	2208.0214	2208.0191	0.0023	0	A.CQCLVRTPEVDDEALEKF.D	(Ions score 29)
119 - 136	737.0147	2208.0223	2208.0191	0.0032	0	A.CQCLVRTPEVDDEALEKF.D	(Ions score 51)
119 - 136	737.0162	2208.0269	2208.0191	0.0078	0	A.CQCLVRTPEVDDEALEKF.D	(Ions score 60)
120 - 136	683.6693	2047.9861	2047.9884	-0.0023	0	C.QCLVRTPEVDDEALEKF.D	(Ions score 45)
120 - 136	683.6707	2047.9901	2047.9884	0.0017	0	C.QCLVRTPEVDDEALEKF.D	(Ions score 22)
120 - 136	1025.0035	2047.9925	2047.9884	0.0041	0	C.QCLVRTPEVDDEALEKF.D	(Ions score 45)
121 - 136	960.9714	1919.9282	1919.9298	-0.0016	0	Q.CLVRTPEVDDEALEKF.D	(Ions score 66)
121 - 136	640.9840	1919.9302	1919.9298	0.0004	0	Q.CLVRTPEVDDEALEKF.D	(Ions score 70)
121 - 136	640.9844	1919.9315	1919.9298	0.0017	0	Q.CLVRTPEVDDEALEKF.D	(Ions score 67)
121 - 136	640.9846	1919.9318	1919.9298	0.0020	0	Q.CLVRTPEVDDEALEKF.D	(Ions score 41)
121 - 136	640.9847	1919.9324	1919.9298	0.0026	0	Q.CLVRTPEVDDEALEKF.D	(Ions score 58)
121 - 136	960.9737	1919.9328	1919.9298	0.0030	0	Q.CLVRTPEVDDEALEKF.D	(Ions score 62)
121 - 136	640.9850	1919.9331	1919.9298	0.0033	0	Q.CLVRTPEVDDEALEKF.D	(Ions score 37)
121 - 136	640.9852	1919.9337	1919.9298	0.0039	0	Q.CLVRTPEVDDEALEKF.D	(Ions score 52)
121 - 136	640.9857	1919.9353	1919.9298	0.0055	0	Q.CLVRTPEVDDEALEKF.D	(Ions score 66)
122 - 133	678.8545	1355.6944	1355.6932	0.0012	0	C.LVRTPEVDDEAL.E	(Ions score 61)
122 - 136	587.6404	1759.8995	1759.8992	0.0003	0	C.LVRTPEVDDEALEKF.D	(Ions score 48)
122 - 136	587.6406	1759.9000	1759.8992	0.0009	0	C.LVRTPEVDDEALEKF.D	(Ions score 50)
122 - 136	880.9578	1759.9011	1759.8992	0.0019	0	C.LVRTPEVDDEALEKF.D	(Ions score 74)
122 - 136	880.9583	1759.9020	1759.8992	0.0028	0	C.LVRTPEVDDEALEKF.D	(Ions score 70)
122 - 136	587.6413	1759.9021	1759.8992	0.0029	0	C.LVRTPEVDDEALEKF.D	(Ions score 57)
122 - 136	880.9584	1759.9023	1759.8992	0.0031	0	C.LVRTPEVDDEALEKF.D	(Ions score 68)
122 - 136	587.6417	1759.9032	1759.8992	0.0040	0	C.LVRTPEVDDEALEKF.D	(Ions score 57)
122 - 141	772.7538	2315.2395	2315.2372	0.0023	0	C.LVRTPEVDDEALEKFDKALK.A	(Ions score 25)
123 - 136	549.9456	1646.8148	1646.8151	-0.0003	0	L.VRTPEVDDEALEKF.D	(Ions score 34)
123 - 136	824.4152	1646.8159	1646.8151	0.0008	0	L.VRTPEVDDEALEKF.D	(Ions score 92)
123 - 136	549.9464	1646.8172	1646.8151	0.0021	0	L.VRTPEVDDEALEKF.D	(Ions score 59)
123 - 136	549.9464	1646.8174	1646.8151	0.0023	0	L.VRTPEVDDEALEKF.D	(Ions score 49)
123 - 136	824.4160	1646.8175	1646.8151	0.0024	0	L.VRTPEVDDEALEKF.D	(Ions score 62)
124 - 136	516.9225	1547.7456	1547.7467	-0.0011	0	V.RTPEVDDEALEKF.D	(Ions score 46)
124 - 136	516.9228	1547.7465	1547.7467	-0.0002	0	V.RTPEVDDEALEKF.D	(Ions score 52)
124 - 136	774.8810	1547.7475	1547.7467	0.0008	0	V.RTPEVDDEALEKF.D	(Ions score 65)
124 - 136	774.8812	1547.7478	1547.7467	0.0011	0	V.RTPEVDDEALEKF.D	(Ions score 51)
125 - 136	696.8303	1391.6460	1391.6456	0.0004	0	R.TPEVDDEALEKF.D	(Ions score 61)
125 - 136	696.8303	1391.6461	1391.6456	0.0005	0	R.TPEVDDEALEKF.D	(Ions score 81)
125 - 136	696.8306	1391.6467	1391.6456	0.0011	0	R.TPEVDDEALEKF.D	(Ions score 81)
125 - 136	696.8308	1391.6471	1391.6456	0.0015	0	R.TPEVDDEALEKF.D	(Ions score 81)
134 - 140	425.7372	849.4599	849.4596	0.0004	0	L.EKFDKAL.K	(Ions score 26)
137 - 145	493.7881	985.5617	985.5630	-0.0013	0	F.DKALKALPM.H	(Ions score 39)

137 - 145	493.7888	985.5631	985.5630	0.0001	0	F.DKALKALPM.H	(Ions score 45)
137 - 145	493.7889	985.5633	985.5630	0.0003	0	F.DKALKALPM.H	(Ions score 45)
137 - 145	493.7889	985.5633	985.5630	0.0003	0	F.DKALKALPM.H	(Ions score 20)
137 - 145	493.7889	985.5633	985.5630	0.0003	0	F.DKALKALPM.H	(Ions score 41)
137 - 145	493.7889	985.5633	985.5630	0.0003	0	F.DKALKALPM.H	(Ions score 44)
137 - 145	493.7889	985.5633	985.5630	0.0003	0	F.DKALKALPM.H	(Ions score 42)
137 - 145	501.7860	1001.5573	1001.5579	-0.0006	0	F.DKALKALPM.H Oxidation (M)	(Ions score 48)
137 - 145	501.7860	1001.5574	1001.5579	-0.0005	0	F.DKALKALPM.H Oxidation (M)	(Ions score 34)
137 - 148	464.9430	1391.8072	1391.8071	0.0002	0	F.DKALKALPMHIR.L	(Ions score 25)
137 - 148	464.9432	1391.8078	1391.8071	0.0007	0	F.DKALKALPMHIR.L	(Ions score 29)
139 - 145	372.2278	742.4411	742.4411	0.0000	0	K.ALKALPM.H	(Ions score 33)
139 - 145	372.2279	742.4413	742.4411	0.0002	0	K.ALKALPM.H	(Ions score 22)
141 - 148	322.5281	964.5624	964.5640	-0.0016	0	L.KALPMHIR.L	(Ions score 22)
141 - 148	322.5282	964.5627	964.5640	-0.0013	0	L.KALPMHIR.L	(Ions score 37)
141 - 148	322.5284	964.5634	964.5640	-0.0006	0	L.KALPMHIR.L	(Ions score 22)
141 - 148	322.5285	964.5637	964.5640	-0.0003	0	L.KALPMHIR.L	(Ions score 31)
141 - 148	483.2892	964.5639	964.5640	-0.0001	0	L.KALPMHIR.L	(Ions score 28)
141 - 148	322.5288	964.5645	964.5640	0.0005	0	L.KALPMHIR.L	(Ions score 31)
142 - 148	419.2421	836.4696	836.4691	0.0005	0	K.ALPMHIR.L	(Ions score 31)
146 - 150	313.1921	624.3696	624.3707	-0.0011	0	M.HIRLS.F	(Ions score 23)
146 - 162	708.0221	2121.0445	2121.0425	0.0019	0	M.HIRLSFNPTQLEEQCHI.-	(Ions score 54)
149 - 154	678.3461	677.3389	677.3384	0.0004	0	R.LSFNPT.Q	(Ions score 29)
149 - 162	858.4049	1714.7953	1714.7984	-0.0032	0	R.LSFNPTQLEEQCHI.-	(Ions score 41)
149 - 162	858.4056	1714.7967	1714.7984	-0.0017	0	R.LSFNPTQLEEQCHI.-	(Ions score 92)
149 - 162	858.4056	1714.7967	1714.7984	-0.0017	0	R.LSFNPTQLEEQCHI.-	(Ions score 29)
149 - 162	858.4063	1714.7981	1714.7984	-0.0004	0	R.LSFNPTQLEEQCHI.-	(Ions score 45)
149 - 162	858.4071	1714.7997	1714.7984	0.0012	0	R.LSFNPTQLEEQCHI.-	(Ions score 46)
149 - 162	858.4078	1714.8010	1714.7984	0.0026	0	R.LSFNPTQLEEQCHI.-	(Ions score 80)
149 - 162	858.4079	1714.8012	1714.7984	0.0028	0	R.LSFNPTQLEEQCHI.-	(Ions score 81)
149 - 162	858.4080	1714.8014	1714.7984	0.0029	0	R.LSFNPTQLEEQCHI.-	(Ions score 52)
149 - 162	858.4086	1714.8027	1714.7984	0.0043	0	R.LSFNPTQLEEQCHI.-	(Ions score 79)
149 - 162	858.4088	1714.8030	1714.7984	0.0045	0	R.LSFNPTQLEEQCHI.-	(Ions score 81)
149 - 162	858.4090	1714.8034	1714.7984	0.0050	0	R.LSFNPTQLEEQCHI.-	(Ions score 88)
150 - 162	801.8647	1601.7149	1601.7144	0.0006	0	L.SFNPTQLEEQCHI.-	(Ions score 66)
151 - 159	553.2614	1104.5083	1104.5087	-0.0005	0	S.FNPTQLEEQ.C	(Ions score 35)
151 - 159	553.2616	1104.5086	1104.5087	-0.0001	0	S.FNPTQLEEQ.C	(Ions score 42)
151 - 162	758.3489	1514.6833	1514.6824	0.0010	0	S.FNPTQLEEQCHI.-	(Ions score 57)
151 - 162	758.3490	1514.6834	1514.6824	0.0011	0	S.FNPTQLEEQCHI.-	(Ions score 59)
151 - 162	758.3496	1514.6847	1514.6824	0.0023	0	S.FNPTQLEEQCHI.-	(Ions score 57)
151 - 162	758.3499	1514.6853	1514.6824	0.0029	0	S.FNPTQLEEQCHI.-	(Ions score 60)
152 - 162	684.8141	1367.6136	1367.6140	-0.0003	0	F.NPTQLEEQCHI.-	(Ions score 40)
152 - 162	684.8149	1367.6153	1367.6140	0.0014	0	F.NPTQLEEQCHI.-	(Ions score 35)
153 - 162	627.7930	1253.5715	1253.5710	0.0005	0	N.PTQLEEQCHI.-	(Ions score 47)
153 - 162	627.7933	1253.5720	1253.5710	0.0010	0	N.PTQLEEQCHI.-	(Ions score 47)
153 - 162	627.7934	1253.5722	1253.5710	0.0012	0	N.PTQLEEQCHI.-	(Ions score 50)
155 - 162	528.7423	1055.4701	1055.4706	-0.0005	0	T.QLEEQCHI.-	(Ions score 33)
155 - 162	528.7426	1055.4707	1055.4706	0.0001	0	T.QLEEQCHI.-	(Ions score 34)
155 - 162	528.7426	1055.4707	1055.4706	0.0001	0	T.QLEEQCHI.-	(Ions score 21)
155 - 162	528.7427	1055.4709	1055.4706	0.0003	0	T.QLEEQCHI.-	(Ions score 30)
155 - 162	528.7427	1055.4709	1055.4706	0.0003	0	T.QLEEQCHI.-	(Ions score 28)
155 - 162	528.7432	1055.4718	1055.4706	0.0012	0	T.QLEEQCHI.-	(Ions score 29)
155 - 162	528.7433	1055.4720	1055.4706	0.0014	0	T.QLEEQCHI.-	(Ions score 29)

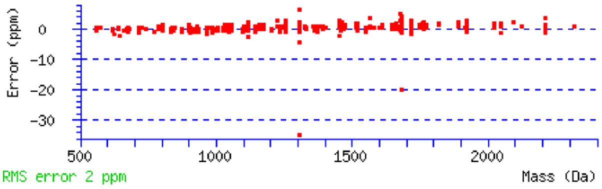

Mascot: http://www.matrixscience.com/
