# Supplementary material for: An alternative for proteinase K-heat-sensitive protease from fungus Onygena corvina for biotechnology: cloning, engineering, expression, characterization and special application for protein sequencing
Source: Microb Cell Fact. 2020 Jun 24;19:135. doi: 10.1186/s12934-020-01392-3 (PMC7313183; doi:10.1186/s12934-020-01392-3)
Supplement: Supplementary file 17 — Additional file 17. MASCOT Search Results of NHSSP-cleaved monoclonal mAb heavy chain. [file 12934_2020_1392_MOESM17_ESM.pdf]

Mascot Search Results

Protein View

Match to: **mAb_HC** Score: **4625**

Found in search of \\SERVER3\User\Kunden_Projekte\Koller_8375\Further-characterization-part-I\8375_mAB_HL1_1070nl.mgf

Nominal mass (M_r): **49839**; Calculated pI value: **8.67**
NCBI BLAST search of mAb_HC against nr
Unformatted sequence string for pasting into other applications

Fixed modifications: Carbamidomethyl (C)
Variable modifications: Oxidation (M)
No enzyme cleavage specificity
Sequence Coverage: **84%**

Matched peptides shown in **Bold Red**

```
  1 QVQLQQPGAE LVKPGASVKM SCKASGYTFT SYNMHWVKQT PGRGLEWIGA
 51 IYPGNGDTSY NQKFKGKATL TADKSSSTAY MQLSSLTSED SAVYYCARST
101 YYGGDWYFNV WGAGTTVTVS AASTKGPSVF PLAPSSKSTS GGTAALGCLV
151 KDYFPEPVTV SWNSGALTSG VHTFPAVLQS SGLYSLSSVV TVPSSSLGTQ
201 TYICNVNHKP SNTKVDKKVE PKSCDKTHTC PPCPAPELLG GPSVFLFPPK
251 PKDTLMISRT PEVTCVVVDV SHEDPEVKFN WYVDGVEVHN AKTKPREEQY
301 NSTYRVVSVL TVLHQDWLNG KEYKCKVSNK ALPAPIEKTI SKAKGQPREP
351 QVYTLPPSRD ELTKNQVSLT CLVKGFYPSD IAVEWESNGQ PENNYKTTPP
401 VLDSDGSFFL YSKLTVDKSR WQQGNVFSCS VMHEALHNHY TQKSLSLSPG
451 K
```

Sort Peptides By ◉ Residue Number ○ Increasing Mass ○ Decreasing Mass

Start - End	Observed	Mr(expt)	Mr(calc)	Delta	Miss	Sequence
5 - 15	562.3089	1122.6032	1122.6033	-0.0000	0	L.QQPGAELVKPG.A (Ions score 43)
5 - 19	503.6189	1507.8348	1507.8358	-0.0010	0	L.QQPGAELVKPGASVK.M (Ions score 25)
6 - 15	498.2774	994.5403	994.5447	-0.0044	0	Q.QPGAELVKPG.A (Ions score 40)
6 - 19	460.9336	1379.7790	1379.7772	0.0018	0	Q.QPGAELVKPGASVK.M (Ions score 23)
22 - 30	517.7344	1033.4543	1033.4539	0.0005	0	S.CKASGYTFT.S (Ions score 22)
31 - 38	532.7529	1063.4912	1063.4909	0.0003	0	T.SYNMHWVK.Q (Ions score 31)
31 - 38	532.7531	1063.4917	1063.4909	0.0008	0	T.SYNMHWVK.Q (Ions score 40)
33 - 38	407.7101	813.4056	813.3956	0.0100	0	Y.NMHWVK.Q (Ions score 27)
39 - 49	607.3203	1212.6260	1212.6251	0.0009	0	K.QTPGRGLEWIG.A (Ions score 30)
39 - 50	642.8381	1283.6616	1283.6622	-0.0006	0	K.QTPGRGLEWIGA.I (Ions score 25)
39 - 50	642.8386	1283.6627	1283.6622	0.0005	0	K.QTPGRGLEWIGA.I (Ions score 27)
39 - 50	642.8391	1283.6635	1283.6622	0.0014	0	K.QTPGRGLEWIGA.I (Ions score 26)
39 - 54	857.9503	1713.8861	1713.8838	0.0023	0	K.QTPGRGLEWIGAIYPG.N (Ions score 28)
39 - 55	914.9715	1827.9284	1827.9267	0.0017	0	K.QTPGRGLEWIGAIYPGN.G (Ions score 52)
39 - 55	914.9720	1827.9294	1827.9267	0.0027	0	K.QTPGRGLEWIGAIYPGN.G (Ions score 29)
39 - 62	865.4149	2593.2227	2593.2197	0.0031	0	K.QTPGRGLEWIGAIYPGNGDTSYNQ.K (Ions score 51)
39 - 62	865.4149	2593.2229	2593.2197	0.0033	0	K.QTPGRGLEWIGAIYPGNGDTSYNQ.K (Ions score 59)
39 - 62	1297.6193	2593.2240	2593.2197	0.0043	0	K.QTPGRGLEWIGAIYPGNGDTSYNQ.K (Ions score 47)
39 - 62	1297.6194	2593.2242	2593.2197	0.0046	0	K.QTPGRGLEWIGAIYPGNGDTSYNQ.K (Ions score 53)
46 - 50	575.2818	574.2745	574.2751	-0.0006	0	L.EWIGA.I (Ions score 25)
46 - 50	575.2823	574.2751	574.2751	-0.0000	0	L.EWIGA.I (Ions score 20)
63 - 67	304.1998	606.3850	606.3853	-0.0003	0	Q.KFKGK.A (Ions score 22)
63 - 80	635.3424	1903.0054	1903.0050	0.0004	0	Q.KFKGKATLTADKSSSTAY.M (Ions score 47)
68 - 73	591.3138	590.3065	590.2912	0.0154	0	K.ATLTAD.K (Ions score 20)
68 - 80	658.3226	1314.6307	1314.6303	0.0004	0	K.ATLTADKSSSTAY.M (Ions score 74)
72 - 78	348.1635	694.3125	694.3133	-0.0008	0	T.ADKSSST.A (Ions score 25)
72 - 80	465.2138	928.4130	928.4138	-0.0008	0	T.ADKSSSTAY.M (Ions score 48)
85 - 95	617.7777	1233.5409	1233.5401	0.0008	0	S.SLTSEDSAVYY.C (Ions score 44)
85 - 95	617.7778	1233.5411	1233.5401	0.0010	0	S.SLTSEDSAVYY.C (Ions score 55)
95 - 102	542.2307	1082.4469	1082.4491	-0.0022	0	Y.YCARSTYY.G (Ions score 30)
99 - 109	686.7780	1371.5415	1371.5407	0.0007	0	R.STYYGGDWYFN.V (Ions score 66)
99 - 109	686.7780	1371.5415	1371.5407	0.0007	0	R.STYYGGDWYFN.V (Ions score 59)
99 - 109	1372.5493	1371.5420	1371.5407	0.0013	0	R.STYYGGDWYFN.V (Ions score 29)
101 - 109	592.7376	1183.4607	1183.4610	-0.0004	0	T.YYGGDWYFN.V (Ions score 31)
121 - 132	587.8264	1173.6383	1173.6393	-0.0010	0	S.AASTKGPSVFPL.A (Ions score 33)
121 - 132	587.8265	1173.6385	1173.6393	-0.0008	0	S.AASTKGPSVFPL.A (Ions score 42)
121 - 132	587.8268	1173.6390	1173.6393	-0.0003	0	S.AASTKGPSVFPL.A (Ions score 50)
121 - 132	587.8269	1173.6393	1173.6393	-0.0001	0	S.AASTKGPSVFPL.A (Ions score 41)
121 - 132	587.8270	1173.6394	1173.6393	0.0001	0	S.AASTKGPSVFPL.A (Ions score 49)
121 - 132	587.8273	1173.6401	1173.6393	0.0008	0	S.AASTKGPSVFPL.A (Ions score 44)
121 - 132	587.8275	1173.6404	1173.6393	0.0010	0	S.AASTKGPSVFPL.A (Ions score 52)
121 - 132	587.8278	1173.6410	1173.6393	0.0016	0	S.AASTKGPSVFPL.A (Ions score 53)
121 - 135	715.3865	1428.7584	1428.7612	-0.0028	0	S.AASTKGPSVFPLAPS.S (Ions score 37)
121 - 135	715.3891	1428.7636	1428.7612	0.0024	0	S.AASTKGPSVFPLAPS.S (Ions score 41)
121 - 137	548.9688	1643.8846	1643.8882	-0.0036	0	S.AASTKGPSVFPLAPSSK.S (Ions score 38)
121 - 146	797.4141	2389.2204	2389.2488	-0.0285	0	S.AASTKGPSVFPLAPSSKSTSGGTAAL.G (Ions score 46)
121 - 146	797.4184	2389.2334	2389.2488	-0.0155	0	S.AASTKGPSVFPLAPSSKSTSGGTAAL.G (Ions score 50)
121 - 146	797.4256	2389.2550	2389.2488	0.0061	0	S.AASTKGPSVFPLAPSSKSTSGGTAAL.G (Ions score 45)
121 - 146	1195.6366	2389.2586	2389.2488	0.0098	0	S.AASTKGPSVFPLAPSSKSTSGGTAAL.G (Ions score 50)
123 - 132	516.7892	1031.5638	1031.5651	-0.0013	0	A.STKGPSVFPL.A (Ions score 28)
123 - 132	516.7892	1031.5639	1031.5651	-0.0012	0	A.STKGPSVFPL.A (Ions score 28)
123 - 132	516.7892	1031.5639	1031.5651	-0.0012	0	A.STKGPSVFPL.A (Ions score 28)
123 - 132	516.7899	1031.5652	1031.5651	0.0001	0	A.STKGPSVFPL.A (Ions score 27)
123 - 132	516.7899	1031.5652	1031.5651	0.0001	0	A.STKGPSVFPL.A (Ions score 33)
123 - 132	516.7902	1031.5658	1031.5651	0.0007	0	A.STKGPSVFPL.A (Ions score 36)
123 - 132	516.7903	1031.5660	1031.5651	0.0009	0	A.STKGPSVFPL.A (Ions score 27)

123 - 135	644.3497	1286.6849	1286.6870	-0.0021	0	A.STKGPSVFPLAPS.S	(Ions score 47)
123 - 135	644.3505	1286.6864	1286.6870	-0.0006	0	A.STKGPSVFPLAPS.S	(Ions score 49)
123 - 135	644.3505	1286.6865	1286.6870	-0.0005	0	A.STKGPSVFPLAPS.S	(Ions score 42)
123 - 135	644.3510	1286.6874	1286.6870	0.0003	0	A.STKGPSVFPLAPS.S	(Ions score 40)
123 - 135	644.3517	1286.6888	1286.6870	0.0018	0	A.STKGPSVFPLAPS.S	(Ions score 44)
123 - 137	501.6111	1501.8113	1501.8140	-0.0027	0	A.STKGPSVFPLAPSSK.S	(Ions score 29)
123 - 137	751.9130	1501.8114	1501.8140	-0.0026	0	A.STKGPSVFPLAPSSK.S	(Ions score 78)
123 - 146	750.0565	2247.1477	2247.1746	-0.0269	0	A.STKGPSVFPLAPSSKSTSGGTAAL.G	(Ions score 60)
123 - 146	750.0670	2247.1790	2247.1746	0.0044	0	A.STKGPSVFPLAPSSKSTSGGTAAL.G	(Ions score 45)
123 - 146	1124.5977	2247.1808	2247.1746	0.0062	0	A.STKGPSVFPLAPSSKSTSGGTAAL.G	(Ions score 56)
123 - 146	750.0679	2247.1820	2247.1746	0.0074	0	A.STKGPSVFPLAPSSKSTSGGTAAL.G	(Ions score 44)
124 - 132	473.2735	944.5324	944.5331	-0.0006	0	S.TKGPSVFPL.A	(Ions score 27)
133 - 146	617.8128	1233.6111	1233.6201	-0.0090	0	L.APSSKSTSGGTAAL.G	(Ions score 76)
133 - 146	617.8245	1233.6344	1233.6201	0.0143	0	L.APSSKSTSGGTAAL.G	(Ions score 73)
133 - 146	617.8245	1233.6344	1233.6201	0.0143	0	L.APSSKSTSGGTAAL.G	(Ions score 77)
133 - 146	617.8254	1233.6363	1233.6201	0.0163	0	L.APSSKSTSGGTAAL.G	(Ions score 60)
136 - 146	490.2564	978.4982	978.4982	0.0001	0	S.SKSTSGGTAAL.G	(Ions score 28)
147 - 158	712.3506	1422.6866	1422.6853	0.0013	0	L.GCLVKDYFPEPV.T	(Ions score 54)
147 - 158	712.3513	1422.6881	1422.6853	0.0028	0	L.GCLVKDYFPEPV.T	(Ions score 60)
147 - 160	812.4092	1622.8038	1622.8014	0.0024	0	L.GCLVKDYFPEPVTV.S	(Ions score 42)
147 - 160	812.4092	1622.8039	1622.8014	0.0026	0	L.GCLVKDYFPEPVTV.S	(Ions score 49)
147 - 161	855.9241	1709.8337	1709.8334	0.0003	0	L.GCLVKDYFPEPVTVS.W	(Ions score 60)
147 - 161	855.9246	1709.8346	1709.8334	0.0012	0	L.GCLVKDYFPEPVTVS.W	(Ions score 49)
147 - 161	855.9247	1709.8349	1709.8334	0.0015	0	L.GCLVKDYFPEPVTVS.W	(Ions score 56)
147 - 161	855.9249	1709.8352	1709.8334	0.0018	0	L.GCLVKDYFPEPVTVS.W	(Ions score 49)
147 - 161	855.9250	1709.8354	1709.8334	0.0020	0	L.GCLVKDYFPEPVTVS.W	(Ions score 20)
147 - 161	855.9250	1709.8355	1709.8334	0.0021	0	L.GCLVKDYFPEPVTVS.W	(Ions score 47)
147 - 161	855.9250	1709.8355	1709.8334	0.0021	0	L.GCLVKDYFPEPVTVS.W	(Ions score 45)
147 - 161	855.9252	1709.8359	1709.8334	0.0025	0	L.GCLVKDYFPEPVTVS.W	(Ions score 28)
147 - 161	855.9254	1709.8363	1709.8334	0.0029	0	L.GCLVKDYFPEPVTVS.W	(Ions score 39)
147 - 161	855.9255	1709.8364	1709.8334	0.0030	0	L.GCLVKDYFPEPVTVS.W	(Ions score 25)
147 - 161	855.9260	1709.8375	1709.8334	0.0041	0	L.GCLVKDYFPEPVTVS.W	(Ions score 38)
147 - 161	855.9262	1709.8379	1709.8334	0.0045	0	L.GCLVKDYFPEPVTVS.W	(Ions score 27)
147 - 161	855.9268	1709.8390	1709.8334	0.0056	0	L.GCLVKDYFPEPVTVS.W	(Ions score 44)
147 - 162	948.9644	1895.9142	1895.9127	0.0014	0	L.GCLVKDYFPEPVTVSW.N	(Ions score 48)
147 - 162	948.9652	1895.9159	1895.9127	0.0031	0	L.GCLVKDYFPEPVTVSW.N	(Ions score 29)
149 - 161	747.3968	1492.7790	1492.7813	-0.0023	0	C.LVKDYFPEPVTVS.W	(Ions score 50)
149 - 161	747.3986	1492.7826	1492.7813	0.0013	0	C.LVKDYFPEPVTVS.W	(Ions score 35)
149 - 161	747.3991	1492.7837	1492.7813	0.0024	0	C.LVKDYFPEPVTVS.W	(Ions score 68)
150 - 161	690.8553	1379.6961	1379.6972	-0.0011	0	L.VKDYFPEPVTVS.W	(Ions score 49)
150 - 161	690.8554	1379.6963	1379.6972	-0.0010	0	L.VKDYFPEPVTVS.W	(Ions score 52)
150 - 161	690.8558	1379.6970	1379.6972	-0.0002	0	L.VKDYFPEPVTVS.W	(Ions score 31)
152 - 161	577.2741	1152.5337	1152.5339	-0.0002	0	K.DYFPEPVTVS.W	(Ions score 50)
152 - 161	577.2742	1152.5339	1152.5339	0.0000	0	K.DYFPEPVTVS.W	(Ions score 53)
152 - 161	577.2744	1152.5342	1152.5339	0.0003	0	K.DYFPEPVTVS.W	(Ions score 63)
152 - 161	577.2744	1152.5342	1152.5339	0.0003	0	K.DYFPEPVTVS.W	(Ions score 53)
152 - 161	577.2745	1152.5344	1152.5339	0.0005	0	K.DYFPEPVTVS.W	(Ions score 59)
152 - 161	577.2746	1152.5346	1152.5339	0.0008	0	K.DYFPEPVTVS.W	(Ions score 53)
152 - 161	577.2747	1152.5348	1152.5339	0.0009	0	K.DYFPEPVTVS.W	(Ions score 50)
152 - 161	577.2747	1152.5349	1152.5339	0.0010	0	K.DYFPEPVTVS.W	(Ions score 53)
152 - 161	577.2747	1152.5349	1152.5339	0.0010	0	K.DYFPEPVTVS.W	(Ions score 45)
162 - 172	564.7763	1127.5381	1127.5359	0.0021	0	S.WNSGALTSGVH.T	(Ions score 70)
162 - 172	564.7765	1127.5384	1127.5359	0.0025	0	S.WNSGALTSGVH.T	(Ions score 73)
162 - 172	564.7769	1127.5392	1127.5359	0.0032	0	S.WNSGALTSGVH.T	(Ions score 64)
162 - 178	878.9551	1755.8957	1755.8944	0.0014	0	S.WNSGALTSGVHTFPAVL.Q	(Ions score 68)
162 - 178	878.9556	1755.8966	1755.8944	0.0022	0	S.WNSGALTSGVHTFPAVL.Q	(Ions score 65)
162 - 178	878.9556	1755.8966	1755.8944	0.0022	0	S.WNSGALTSGVHTFPAVL.Q	(Ions score 41)
162 - 179	942.9847	1883.9549	1883.9529	0.0020	0	S.WNSGALTSGVHTFPAVLQ.S	(Ions score 62)
163 - 178	785.9149	1569.8153	1569.8150	0.0002	0	W.NSGALTSGVHTFPAVL.Q	(Ions score 83)
163 - 178	785.9152	1569.8158	1569.8150	0.0007	0	W.NSGALTSGVHTFPAVL.Q	(Ions score 48)
168 - 178	564.8049	1127.5953	1127.5975	-0.0022	0	L.TSGVHTFPAVL.Q	(Ions score 47)
168 - 178	564.8055	1127.5965	1127.5975	-0.0009	0	L.TSGVHTFPAVL.Q	(Ions score 30)
170 - 178	470.7657	939.5169	939.5178	-0.0009	0	S.GVHTFPAVL.Q	(Ions score 22)
170 - 178	470.7659	939.5172	939.5178	-0.0005	0	S.GVHTFPAVL.Q	(Ions score 24)
170 - 178	470.7661	939.5176	939.5178	-0.0002	0	S.GVHTFPAVL.Q	(Ions score 20)
170 - 178	470.7662	939.5179	939.5178	0.0001	0	S.GVHTFPAVL.Q	(Ions score 29)
170 - 178	940.5266	939.5193	939.5178	0.0015	0	S.GVHTFPAVL.Q	(Ions score 30)
173 - 178	647.3764	646.3691	646.3690	0.0001	0	H.TFPAVL.Q	(Ions score 22)
173 - 178	647.3768	646.3696	646.3690	0.0006	0	H.TFPAVL.Q	(Ions score 23)
173 - 178	647.3775	646.3702	646.3690	0.0012	0	H.TFPAVL.Q	(Ions score 28)
173 - 179	388.2208	774.4270	774.4276	-0.0005	0	H.TFPAVLQ.S	(Ions score 33)
173 - 179	388.2209	774.4272	774.4276	-0.0003	0	H.TFPAVLQ.S	(Ions score 32)
173 - 179	388.2211	774.4276	774.4276	0.0001	0	H.TFPAVLQ.S	(Ions score 37)
173 - 179	388.2211	774.4276	774.4276	0.0001	0	H.TFPAVLQ.S	(Ions score 35)
173 - 179	388.2211	774.4277	774.4276	0.0001	0	H.TFPAVLQ.S	(Ions score 29)
173 - 179	775.4390	774.4317	774.4276	0.0042	0	H.TFPAVLQ.S	(Ions score 22)
173 - 184	641.8373	1281.6601	1281.6605	-0.0003	0	H.TFPAVLQSSGLY.S	(Ions score 24)
173 - 184	641.8377	1281.6609	1281.6605	0.0004	0	H.TFPAVLQSSGLY.S	(Ions score 23)
173 - 184	641.8379	1281.6612	1281.6605	0.0008	0	H.TFPAVLQSSGLY.S	(Ions score 30)
173 - 184	641.8380	1281.6614	1281.6605	0.0009	0	H.TFPAVLQSSGLY.S	(Ions score 23)
185 - 197	631.8458	1261.6770	1261.6765	0.0005	0	Y.SLSSVVTVPSSSL.G	(Ions score 35)
185 - 197	631.8462	1261.6778	1261.6765	0.0013	0	Y.SLSSVVTVPSSSL.G	(Ions score 42)
185 - 197	631.8464	1261.6782	1261.6765	0.0017	0	Y.SLSSVVTVPSSSL.G	(Ions score 34)
185 - 200	774.9111	1547.8076	1547.8042	0.0034	0	Y.SLSSVVTVPSSSLGTQ.T	(Ions score 22)
187 - 200	674.8537	1347.6928	1347.6881	0.0047	0	L.SSVVTVPSSSLGTQ.T	(Ions score 47)
187 - 200	674.8544	1347.6942	1347.6881	0.0060	0	L.SSVVTVPSSSLGTQ.T	(Ions score 69)
187 - 200	1348.7015	1347.6943	1347.6881	0.0061	0	L.SSVVTVPSSSLGTQ.T	(Ions score 43)
188 - 200	631.3360	1260.6574	1260.6561	0.0013	0	S.SVVTVPSSSLGTQ.T	(Ions score 50)
188 - 200	631.3376	1260.6607	1260.6561	0.0046	0	S.SVVTVPSSSLGTQ.T	(Ions score 80)
188 - 202	763.3914	1524.7682	1524.7671	0.0010	0	S.SVVTVPSSSLGTQTY.I	(Ions score 56)
188 - 202	763.3917	1524.7688	1524.7671	0.0017	0	S.SVVTVPSSSLGTQTY.I	(Ions score 47)
188 - 202	763.3917	1524.7688	1524.7671	0.0017	0	S.SVVTVPSSSLGTQTY.I	(Ions score 26)

191 - 200	488.7506	975.4867	975.4873	-0.0006	0	V.TVPSSSLGTQ.T	(Ions score 33)
191 - 200	488.7509	975.4872	975.4873	-0.0001	0	V.TVPSSSLGTQ.T	(Ions score 54)
191 - 200	488.7509	975.4872	975.4873	-0.0001	0	V.TVPSSSLGTQ.T	(Ions score 26)
191 - 200	976.5012	975.4939	975.4873	0.0067	0	V.TVPSSSLGTQ.T	(Ions score 27)
205 - 211	398.2090	794.4035	794.4035	0.0000	0	C.NVNHKPS.N	(Ions score 27)
223 - 239	991.9439	1981.8733	1981.8696	0.0037	0	K.SCDKTHTCPPCPAPELL.G	(Ions score 33)
223 - 239	661.6321	1981.8746	1981.8696	0.0050	0	K.SCDKTHTCPPCPAPELL.G	(Ions score 24)
223 - 239	661.6322	1981.8748	1981.8696	0.0052	0	K.SCDKTHTCPPCPAPELL.G	(Ions score 21)
223 - 239	661.6326	1981.8759	1981.8696	0.0063	0	K.SCDKTHTCPPCPAPELL.G	(Ions score 34)
223 - 239	661.6328	1981.8764	1981.8696	0.0068	0	K.SCDKTHTCPPCPAPELL.G	(Ions score 20)
223 - 239	661.6332	1981.8779	1981.8696	0.0083	0	K.SCDKTHTCPPCPAPELL.G	(Ions score 24)
223 - 239	991.9462	1981.8779	1981.8696	0.0083	0	K.SCDKTHTCPPCPAPELL.G	(Ions score 44)
223 - 239	661.6340	1981.8801	1981.8696	0.0105	0	K.SCDKTHTCPPCPAPELL.G	(Ions score 21)
223 - 239	991.9479	1981.8812	1981.8696	0.0116	0	K.SCDKTHTCPPCPAPELL.G	(Ions score 44)
225 - 239	579.2779	1734.8119	1734.8069	0.0049	0	C.DKTHTCPPCPAPELL.G	(Ions score 20)
225 - 239	579.2783	1734.8129	1734.8069	0.0060	0	C.DKTHTCPPCPAPELL.G	(Ions score 36)
225 - 239	868.4147	1734.8148	1734.8069	0.0079	0	C.DKTHTCPPCPAPELL.G	(Ions score 50)
225 - 239	868.4150	1734.8154	1734.8069	0.0085	0	C.DKTHTCPPCPAPELL.G	(Ions score 65)
227 - 239	746.8442	1491.6738	1491.6850	-0.0112	0	K.THTCPPCPAPELL.G	(Ions score 37)
227 - 239	746.8452	1491.6759	1491.6850	-0.0092	0	K.THTCPPCPAPELL.G	(Ions score 50)
227 - 239	746.8456	1491.6766	1491.6850	-0.0084	0	K.THTCPPCPAPELL.G	(Ions score 52)
227 - 239	746.8498	1491.6850	1491.6850	0.0000	0	K.THTCPPCPAPELL.G	(Ions score 47)
229 - 239	627.7966	1253.5787	1253.5784	0.0003	0	H.TCPPCPAPELL.G	(Ions score 30)
229 - 239	627.7972	1253.5799	1253.5784	0.0015	0	H.TCPPCPAPELL.G	(Ions score 41)
229 - 239	627.7974	1253.5802	1253.5784	0.0017	0	H.TCPPCPAPELL.G	(Ions score 62)
229 - 239	627.7974	1253.5802	1253.5784	0.0017	0	H.TCPPCPAPELL.G	(Ions score 57)
229 - 239	627.7977	1253.5808	1253.5784	0.0024	0	H.TCPPCPAPELL.G	(Ions score 63)
229 - 239	627.7977	1253.5808	1253.5784	0.0024	0	H.TCPPCPAPELL.G	(Ions score 40)
246 - 252	413.7647	825.5149	825.5112	0.0036	0	F.LFPPKPK.D	(Ions score 25)
246 - 255	385.8943	1154.6611	1154.6699	-0.0088	0	F.LFPPKPKDTL.M	(Ions score 29)
246 - 255	385.8963	1154.6670	1154.6699	-0.0029	0	F.LFPPKPKDTL.M	(Ions score 23)
246 - 255	578.3408	1154.6671	1154.6699	-0.0028	0	F.LFPPKPKDTL.M	(Ions score 22)
246 - 255	578.3408	1154.6671	1154.6699	-0.0028	0	F.LFPPKPKDTL.M	(Ions score 28)
246 - 255	578.3409	1154.6672	1154.6699	-0.0027	0	F.LFPPKPKDTL.M	(Ions score 22)
246 - 255	385.8965	1154.6677	1154.6699	-0.0022	0	F.LFPPKPKDTL.M	(Ions score 28)
246 - 255	578.3412	1154.6678	1154.6699	-0.0021	0	F.LFPPKPKDTL.M	(Ions score 25)
246 - 255	578.3413	1154.6681	1154.6699	-0.0019	0	F.LFPPKPKDTL.M	(Ions score 31)
246 - 255	578.3414	1154.6683	1154.6699	-0.0016	0	F.LFPPKPKDTL.M	(Ions score 27)
246 - 255	578.3416	1154.6687	1154.6699	-0.0012	0	F.LFPPKPKDTL.M	(Ions score 27)
246 - 255	578.3419	1154.6692	1154.6699	-0.0008	0	F.LFPPKPKDTL.M	(Ions score 30)
246 - 255	385.8970	1154.6693	1154.6699	-0.0006	0	F.LFPPKPKDTL.M	(Ions score 26)
246 - 255	385.8971	1154.6694	1154.6699	-0.0006	0	F.LFPPKPKDTL.M	(Ions score 25)
246 - 255	578.3420	1154.6694	1154.6699	-0.0005	0	F.LFPPKPKDTL.M	(Ions score 22)
246 - 255	385.8971	1154.6695	1154.6699	-0.0005	0	F.LFPPKPKDTL.M	(Ions score 31)
246 - 255	578.3421	1154.6697	1154.6699	-0.0003	0	F.LFPPKPKDTL.M	(Ions score 21)
246 - 255	385.8973	1154.6701	1154.6699	0.0002	0	F.LFPPKPKDTL.M	(Ions score 25)
246 - 255	578.3423	1154.6701	1154.6699	0.0002	0	F.LFPPKPKDTL.M	(Ions score 25)
246 - 255	578.3423	1154.6701	1154.6699	0.0002	0	F.LFPPKPKDTL.M	(Ions score 39)
246 - 255	578.3424	1154.6703	1154.6699	0.0003	0	F.LFPPKPKDTL.M	(Ions score 23)
246 - 255	385.8974	1154.6703	1154.6699	0.0004	0	F.LFPPKPKDTL.M	(Ions score 28)
246 - 255	385.8974	1154.6704	1154.6699	0.0004	0	F.LFPPKPKDTL.M	(Ions score 33)
246 - 255	385.8974	1154.6704	1154.6699	0.0004	0	F.LFPPKPKDTL.M	(Ions score 24)
246 - 255	578.3425	1154.6705	1154.6699	0.0006	0	F.LFPPKPKDTL.M	(Ions score 21)
246 - 255	385.8975	1154.6706	1154.6699	0.0006	0	F.LFPPKPKDTL.M	(Ions score 22)
246 - 255	385.8975	1154.6706	1154.6699	0.0007	0	F.LFPPKPKDTL.M	(Ions score 24)
246 - 255	578.3428	1154.6710	1154.6699	0.0011	0	F.LFPPKPKDTL.M	(Ions score 21)
246 - 255	385.8978	1154.6716	1154.6699	0.0016	0	F.LFPPKPKDTL.M	(Ions score 20)
246 - 255	578.3431	1154.6717	1154.6699	0.0018	0	F.LFPPKPKDTL.M	(Ions score 37)
246 - 255	385.8979	1154.6718	1154.6699	0.0019	0	F.LFPPKPKDTL.M	(Ions score 29)
246 - 255	385.8988	1154.6746	1154.6699	0.0047	0	F.LFPPKPKDTL.M	(Ions score 22)
246 - 258	743.9157	1485.8169	1485.8265	-0.0096	0	F.LFPPKPKDTLMIS.R	(Ions score 37)
246 - 258	496.2799	1485.8180	1485.8265	-0.0085	0	F.LFPPKPKDTLMIS.R	(Ions score 20)
246 - 258	743.9165	1485.8185	1485.8265	-0.0080	0	F.LFPPKPKDTLMIS.R	(Ions score 30)
246 - 258	743.9171	1485.8197	1485.8265	-0.0068	0	F.LFPPKPKDTLMIS.R	(Ions score 37)
246 - 258	743.9193	1485.8239	1485.8265	-0.0025	0	F.LFPPKPKDTLMIS.R	(Ions score 32)
246 - 258	743.9217	1485.8288	1485.8265	0.0023	0	F.LFPPKPKDTLMIS.R	(Ions score 34)
246 - 258	743.9222	1485.8299	1485.8265	0.0034	0	F.LFPPKPKDTLMIS.R	(Ions score 28)
246 - 258	743.9225	1485.8304	1485.8265	0.0039	0	F.LFPPKPKDTLMIS.R	(Ions score 37)
246 - 258	743.9231	1485.8316	1485.8265	0.0051	0	F.LFPPKPKDTLMIS.R	(Ions score 36)
265 - 278	806.3873	1610.7601	1610.7610	-0.0009	0	T.CVVVDVSHEDPEVK.F	(Ions score 71)
265 - 278	806.3883	1610.7621	1610.7610	0.0011	0	T.CVVVDVSHEDPEVK.F	(Ions score 70)
265 - 278	537.9283	1610.7632	1610.7610	0.0022	0	T.CVVVDVSHEDPEVK.F	(Ions score 30)
265 - 278	537.9283	1610.7632	1610.7610	0.0022	0	T.CVVVDVSHEDPEVK.F	(Ions score 36)
265 - 280	624.9657	1871.8753	1871.8723	0.0030	0	T.CVVVDVSHEDPEVKFN.W	(Ions score 53)
269 - 280	472.5527	1414.6364	1414.6364	-0.0001	0	V.DVSHEDPEVKFN.W	(Ions score 35)
269 - 280	472.5529	1414.6370	1414.6364	0.0006	0	V.DVSHEDPEVKFN.W	(Ions score 23)
269 - 280	708.3270	1414.6395	1414.6364	0.0031	0	V.DVSHEDPEVKFN.W	(Ions score 33)
270 - 278	347.1733	1038.4980	1038.4982	-0.0001	0	D.VSHEDPEVK.F	(Ions score 25)
270 - 278	520.2583	1038.5021	1038.4982	0.0039	0	D.VSHEDPEVK.F	(Ions score 43)
270 - 278	347.1747	1038.5022	1038.4982	0.0041	0	D.VSHEDPEVK.F	(Ions score 30)
270 - 280	434.2101	1299.6084	1299.6095	-0.0011	0	D.VSHEDPEVKFN.W	(Ions score 47)
270 - 280	650.8120	1299.6095	1299.6095	-0.0000	0	D.VSHEDPEVKFN.W	(Ions score 56)
270 - 280	434.2105	1299.6098	1299.6095	0.0003	0	D.VSHEDPEVKFN.W	(Ions score 46)
270 - 280	434.2106	1299.6100	1299.6095	0.0005	0	D.VSHEDPEVKFN.W	(Ions score 35)
270 - 280	650.8127	1299.6108	1299.6095	0.0013	0	D.VSHEDPEVKFN.W	(Ions score 49)
271 - 280	401.1879	1200.5419	1200.5411	0.0008	0	V.SHEDPEVKFN.W	(Ions score 45)
271 - 280	601.2786	1200.5426	1200.5411	0.0015	0	V.SHEDPEVKFN.W	(Ions score 55)
272 - 280	557.7604	1113.5063	1113.5090	-0.0027	0	S.HEDPEVKFN.W	(Ions score 49)
272 - 280	557.7612	1113.5079	1113.5090	-0.0011	0	S.HEDPEVKFN.W	(Ions score 46)
272 - 280	557.7615	1113.5084	1113.5090	-0.0006	0	S.HEDPEVKFN.W	(Ions score 49)
272 - 280	557.7621	1113.5096	1113.5090	0.0006	0	S.HEDPEVKFN.W	(Ions score 35)
272 - 280	557.7632	1113.5118	1113.5090	0.0028	0	S.HEDPEVKFN.W	(Ions score 39)

275 - 280	367.1965	732.3785	732.3806	-0.0021	0	D.PEVKFN.W	(Ions score 26)
279 - 289	682.8171	1363.6197	1363.6196	0.0001	0	K.FNWYVDGVEVH.N	(Ions score 70)
279 - 289	682.8174	1363.6202	1363.6196	0.0006	0	K.FNWYVDGVEVH.N	(Ions score 36)
279 - 289	682.8175	1363.6205	1363.6196	0.0008	0	K.FNWYVDGVEVH.N	(Ions score 72)
281 - 286	738.3460	737.3387	737.3384	0.0003	0	N.WYVDGV.E	(Ions score 37)
281 - 286	738.3467	737.3395	737.3384	0.0011	0	N.WYVDGV.E	(Ions score 41)
281 - 289	552.2599	1102.5053	1102.5083	-0.0030	0	N.WYVDGVEVH.N	(Ions score 48)
281 - 289	552.2606	1102.5067	1102.5083	-0.0016	0	N.WYVDGVEVH.N	(Ions score 45)
281 - 289	552.2610	1102.5075	1102.5083	-0.0008	0	N.WYVDGVEVH.N	(Ions score 48)
281 - 289	552.2611	1102.5077	1102.5083	-0.0007	0	N.WYVDGVEVH.N	(Ions score 48)
281 - 289	552.2612	1102.5079	1102.5083	-0.0004	0	N.WYVDGVEVH.N	(Ions score 58)
281 - 289	552.2614	1102.5082	1102.5083	-0.0002	0	N.WYVDGVEVH.N	(Ions score 48)
281 - 289	552.2614	1102.5082	1102.5083	-0.0002	0	N.WYVDGVEVH.N	(Ions score 48)
281 - 289	552.2614	1102.5083	1102.5083	-0.0000	0	N.WYVDGVEVH.N	(Ions score 48)
281 - 289	552.2615	1102.5085	1102.5083	0.0002	0	N.WYVDGVEVH.N	(Ions score 48)
281 - 289	552.2615	1102.5085	1102.5083	0.0002	0	N.WYVDGVEVH.N	(Ions score 49)
281 - 289	552.2616	1102.5086	1102.5083	0.0003	0	N.WYVDGVEVH.N	(Ions score 48)
281 - 289	552.2616	1102.5086	1102.5083	0.0003	0	N.WYVDGVEVH.N	(Ions score 48)
281 - 289	552.2616	1102.5086	1102.5083	0.0003	0	N.WYVDGVEVH.N	(Ions score 48)
281 - 289	552.2617	1102.5088	1102.5083	0.0004	0	N.WYVDGVEVH.N	(Ions score 48)
281 - 289	552.2617	1102.5089	1102.5083	0.0006	0	N.WYVDGVEVH.N	(Ions score 52)
281 - 289	552.2617	1102.5089	1102.5083	0.0006	0	N.WYVDGVEVH.N	(Ions score 48)
281 - 289	552.2618	1102.5090	1102.5083	0.0007	0	N.WYVDGVEVH.N	(Ions score 42)
281 - 289	552.2621	1102.5096	1102.5083	0.0013	0	N.WYVDGVEVH.N	(Ions score 45)
282 - 289	459.2223	916.4301	916.4290	0.0011	0	W.YVDGVEVH.N	(Ions score 43)
311 - 319	563.2888	1124.5631	1124.5614	0.0016	0	L.TVLHQDWLN.G	(Ions score 23)
311 - 319	563.2895	1124.5644	1124.5614	0.0030	0	L.TVLHQDWLN.G	(Ions score 51)
311 - 319	563.2896	1124.5647	1124.5614	0.0032	0	L.TVLHQDWLN.G	(Ions score 58)
311 - 321	437.5673	1309.6801	1309.6779	0.0023	0	L.TVLHQDWLNGK.E	(Ions score 21)
320 - 328	366.8583	1097.5532	1097.5539	-0.0007	0	N.GKEYKCKVS.N	(Ions score 23)
320 - 328	549.7848	1097.5550	1097.5539	0.0011	0	N.GKEYKCKVS.N	(Ions score 29)
329 - 341	461.2696	1380.7870	1380.7976	-0.0106	0	S.NKALPAPIEKTIS.K	(Ions score 40)
329 - 341	691.4021	1380.7896	1380.7976	-0.0080	0	S.NKALPAPIEKTIS.K	(Ions score 36)
329 - 341	461.2729	1380.7968	1380.7976	-0.0008	0	S.NKALPAPIEKTIS.K	(Ions score 56)
329 - 341	461.2732	1380.7978	1380.7976	0.0002	0	S.NKALPAPIEKTIS.K	(Ions score 46)
329 - 341	691.4064	1380.7982	1380.7976	0.0006	0	S.NKALPAPIEKTIS.K	(Ions score 31)
329 - 341	461.2733	1380.7982	1380.7976	0.0006	0	S.NKALPAPIEKTIS.K	(Ions score 28)
329 - 341	691.4065	1380.7984	1380.7976	0.0008	0	S.NKALPAPIEKTIS.K	(Ions score 33)
329 - 341	691.4066	1380.7986	1380.7976	0.0009	0	S.NKALPAPIEKTIS.K	(Ions score 32)
329 - 341	691.4066	1380.7987	1380.7976	0.0011	0	S.NKALPAPIEKTIS.K	(Ions score 35)
329 - 341	691.4066	1380.7987	1380.7976	0.0011	0	S.NKALPAPIEKTIS.K	(Ions score 33)
329 - 341	691.4068	1380.7990	1380.7976	0.0014	0	S.NKALPAPIEKTIS.K	(Ions score 28)
329 - 341	691.4072	1380.7999	1380.7976	0.0023	0	S.NKALPAPIEKTIS.K	(Ions score 36)
329 - 341	691.4075	1380.8004	1380.7976	0.0028	0	S.NKALPAPIEKTIS.K	(Ions score 39)
329 - 341	691.4078	1380.8010	1380.7976	0.0034	0	S.NKALPAPIEKTIS.K	(Ions score 36)
329 - 341	691.4091	1380.8036	1380.7976	0.0059	0	S.NKALPAPIEKTIS.K	(Ions score 39)
331 - 341	570.3286	1138.6427	1138.6597	-0.0171	0	K.ALPAPIEKTIS.K	(Ions score 28)
331 - 341	570.3334	1138.6523	1138.6597	-0.0074	0	K.ALPAPIEKTIS.K	(Ions score 27)
331 - 341	570.3378	1138.6611	1138.6597	0.0014	0	K.ALPAPIEKTIS.K	(Ions score 41)
331 - 341	570.3387	1138.6628	1138.6597	0.0031	0	K.ALPAPIEKTIS.K	(Ions score 26)
331 - 341	570.3394	1138.6643	1138.6597	0.0045	0	K.ALPAPIEKTIS.K	(Ions score 34)
331 - 341	570.3401	1138.6657	1138.6597	0.0060	0	K.ALPAPIEKTIS.K	(Ions score 48)
331 - 353	841.1426	2520.4061	2520.4063	-0.0002	0	K.ALPAPIEKTISKAKGQPREPQVY.T	(Ions score 21)
342 - 353	467.5886	1399.7439	1399.7571	-0.0133	0	S.KAKGQPREPQVY.T	(Ions score 23)
342 - 353	467.5926	1399.7560	1399.7571	-0.0011	0	S.KAKGQPREPQVY.T	(Ions score 23)
342 - 353	467.5931	1399.7574	1399.7571	0.0003	0	S.KAKGQPREPQVY.T	(Ions score 22)
342 - 353	467.5932	1399.7577	1399.7571	0.0005	0	S.KAKGQPREPQVY.T	(Ions score 21)
342 - 353	700.8862	1399.7579	1399.7571	0.0008	0	S.KAKGQPREPQVY.T	(Ions score 23)
342 - 353	467.5945	1399.7615	1399.7571	0.0044	0	S.KAKGQPREPQVY.T	(Ions score 21)
343 - 353	636.8416	1271.6687	1271.6622	0.0065	0	K.AKGQPREPQVY.T	(Ions score 29)
345 - 353	537.2710	1072.5274	1072.5301	-0.0027	0	K.GQPREPQVY.T	(Ions score 38)
345 - 353	537.2719	1072.5292	1072.5301	-0.0010	0	K.GQPREPQVY.T	(Ions score 33)
345 - 353	537.2719	1072.5293	1072.5301	-0.0008	0	K.GQPREPQVY.T	(Ions score 30)
345 - 353	537.2722	1072.5299	1072.5301	-0.0002	0	K.GQPREPQVY.T	(Ions score 39)
345 - 353	537.2726	1072.5306	1072.5301	0.0005	0	K.GQPREPQVY.T	(Ions score 43)
354 - 365	685.8605	1369.7065	1369.7201	-0.0136	0	Y.TLPPSRDELTKN.Q	(Ions score 42)
354 - 365	685.8618	1369.7091	1369.7201	-0.0110	0	Y.TLPPSRDELTKN.Q	(Ions score 35)
354 - 365	685.8646	1369.7146	1369.7201	-0.0055	0	Y.TLPPSRDELTKN.Q	(Ions score 40)
354 - 365	685.8660	1369.7174	1369.7201	-0.0027	0	Y.TLPPSRDELTKN.Q	(Ions score 43)
354 - 365	685.8660	1369.7175	1369.7201	-0.0026	0	Y.TLPPSRDELTKN.Q	(Ions score 39)
354 - 365	685.8662	1369.7179	1369.7201	-0.0023	0	Y.TLPPSRDELTKN.Q	(Ions score 55)
354 - 365	685.8662	1369.7179	1369.7201	-0.0023	0	Y.TLPPSRDELTKN.Q	(Ions score 24)
354 - 365	685.8671	1369.7196	1369.7201	-0.0005	0	Y.TLPPSRDELTKN.Q	(Ions score 42)
354 - 365	685.8672	1369.7198	1369.7201	-0.0003	0	Y.TLPPSRDELTKN.Q	(Ions score 45)
354 - 365	685.8672	1369.7198	1369.7201	-0.0003	0	Y.TLPPSRDELTKN.Q	(Ions score 44)
354 - 365	685.8672	1369.7198	1369.7201	-0.0003	0	Y.TLPPSRDELTKN.Q	(Ions score 37)
354 - 365	685.8672	1369.7198	1369.7201	-0.0003	0	Y.TLPPSRDELTKN.Q	(Ions score 40)
354 - 365	685.8674	1369.7203	1369.7201	0.0002	0	Y.TLPPSRDELTKN.Q	(Ions score 34)
354 - 365	685.8676	1369.7206	1369.7201	0.0004	0	Y.TLPPSRDELTKN.Q	(Ions score 41)
354 - 365	685.8686	1369.7226	1369.7201	0.0025	0	Y.TLPPSRDELTKN.Q	(Ions score 36)
354 - 365	685.8687	1369.7229	1369.7201	0.0028	0	Y.TLPPSRDELTKN.Q	(Ions score 42)
354 - 365	685.8690	1369.7235	1369.7201	0.0034	0	Y.TLPPSRDELTKN.Q	(Ions score 50)
354 - 365	685.8701	1369.7256	1369.7201	0.0054	0	Y.TLPPSRDELTKN.Q	(Ions score 45)
354 - 365	685.8713	1369.7280	1369.7201	0.0079	0	Y.TLPPSRDELTKN.Q	(Ions score 35)
354 - 365	685.8742	1369.7339	1369.7201	0.0137	0	Y.TLPPSRDELTKN.Q	(Ions score 39)
354 - 365	685.8743	1369.7340	1369.7201	0.0139	0	Y.TLPPSRDELTKN.Q	(Ions score 37)
354 - 365	685.8751	1369.7356	1369.7201	0.0154	0	Y.TLPPSRDELTKN.Q	(Ions score 43)
354 - 365	685.8756	1369.7367	1369.7201	0.0165	0	Y.TLPPSRDELTKN.Q	(Ions score 41)
354 - 365	685.8759	1369.7373	1369.7201	0.0172	0	Y.TLPPSRDELTKN.Q	(Ions score 39)
354 - 365	685.8759	1369.7373	1369.7201	0.0172	0	Y.TLPPSRDELTKN.Q	(Ions score 42)
354 - 365	685.8762	1369.7379	1369.7201	0.0178	0	Y.TLPPSRDELTKN.Q	(Ions score 42)
354 - 370	950.0071	1897.9996	1898.0109	-0.0113	0	Y.TLPPSRDELTKNQVSLT.C	(Ions score 25)

354 - 370	950.0112	1898.0078	1898.0109	-0.0031	0	Y.TLPPSRDELTKNQVSLT.C		(Ions score 22)
354 - 370	633.6772	1898.0099	1898.0109	-0.0010	0	Y.TLPPSRDELTKNQVSLT.C		(Ions score 25)
354 - 370	950.0124	1898.0102	1898.0109	-0.0007	0	Y.TLPPSRDELTKNQVSLT.C		(Ions score 32)
354 - 370	950.0127	1898.0108	1898.0109	-0.0000	0	Y.TLPPSRDELTKNQVSLT.C		(Ions score 23)
354 - 370	950.0131	1898.0117	1898.0109	0.0008	0	Y.TLPPSRDELTKNQVSLT.C		(Ions score 25)
354 - 370	633.6784	1898.0134	1898.0109	0.0025	0	Y.TLPPSRDELTKNQVSLT.C		(Ions score 40)
354 - 370	633.6788	1898.0145	1898.0109	0.0036	0	Y.TLPPSRDELTKNQVSLT.C		(Ions score 30)
354 - 370	950.0146	1898.0146	1898.0109	0.0037	0	Y.TLPPSRDELTKNQVSLT.C		(Ions score 45)
354 - 370	633.6793	1898.0160	1898.0109	0.0051	0	Y.TLPPSRDELTKNQVSLT.C		(Ions score 24)
354 - 370	633.6793	1898.0161	1898.0109	0.0053	0	Y.TLPPSRDELTKNQVSLT.C		(Ions score 23)
354 - 370	633.6796	1898.0169	1898.0109	0.0060	0	Y.TLPPSRDELTKNQVSLT.C		(Ions score 24)
354 - 370	950.0160	1898.0174	1898.0109	0.0066	0	Y.TLPPSRDELTKNQVSLT.C		(Ions score 23)
354 - 370	633.6806	1898.0200	1898.0109	0.0091	0	Y.TLPPSRDELTKNQVSLT.C		(Ions score 24)
356 - 365	578.8130	1155.6114	1155.5884	0.0230	0	L.PPSRDELTKN.Q		(Ions score 21)
371 - 384	799.4008	1596.7870	1596.7857	0.0012	0	T.CLVKGFYPSDIAVE.W		(Ions score 45)
375 - 384	549.2610	1096.5075	1096.5076	-0.0001	0	K.GFYPSDIAVE.W		(Ions score 30)
375 - 384	549.2611	1096.5077	1096.5076	0.0000	0	K.GFYPSDIAVE.W		(Ions score 42)
375 - 384	549.2612	1096.5079	1096.5076	0.0003	0	K.GFYPSDIAVE.W		(Ions score 56)
375 - 384	549.2614	1096.5083	1096.5076	0.0006	0	K.GFYPSDIAVE.W		(Ions score 64)
375 - 384	549.2615	1096.5084	1096.5076	0.0008	0	K.GFYPSDIAVE.W		(Ions score 58)
375 - 384	549.2616	1096.5086	1096.5076	0.0010	0	K.GFYPSDIAVE.W		(Ions score 34)
385 - 408	1341.6031	2681.1917	2681.1881	0.0037	0	E.WESNGQPENNYKTTPPVLDSDGSF.F		(Ions score 32)
385 - 409	1415.1372	2828.2599	2828.2565	0.0034	0	E.WESNGQPENNYKTTPPVLDSDGSFF.L		(Ions score 32)
385 - 409	943.7608	2828.2606	2828.2565	0.0041	0	E.WESNGQPENNYKTTPPVLDSDGSFF.L		(Ions score 40)
385 - 409	1415.1378	2828.2611	2828.2565	0.0046	0	E.WESNGQPENNYKTTPPVLDSDGSFF.L		(Ions score 47)
385 - 409	943.7612	2828.2619	2828.2565	0.0054	0	E.WESNGQPENNYKTTPPVLDSDGSFF.L		(Ions score 64)
389 - 409	1157.0391	2312.0636	2312.0596	0.0039	0	N.GQPENNYKTTPPVLDSDGSFF.L		(Ions score 56)
389 - 409	1157.0396	2312.0646	2312.0596	0.0049	0	N.GQPENNYKTTPPVLDSDGSFF.L		(Ions score 51)
395 - 409	837.4068	1672.7990	1672.7984	0.0006	0	N.YKTTPPVLDSDGSFF.L		(Ions score 82)
395 - 409	837.4075	1672.8005	1672.7984	0.0021	0	N.YKTTPPVLDSDGSFF.L		(Ions score 46)
395 - 409	837.4077	1672.8008	1672.7984	0.0023	0	N.YKTTPPVLDSDGSFF.L		(Ions score 78)
397 - 409	691.8276	1381.6406	1381.6401	0.0005	0	K.TTPPVLDSDGSFF.L		(Ions score 39)
397 - 409	691.8281	1381.6417	1381.6401	0.0016	0	K.TTPPVLDSDGSFF.L		(Ions score 87)
397 - 409	691.8282	1381.6419	1381.6401	0.0018	0	K.TTPPVLDSDGSFF.L		(Ions score 58)
397 - 409	691.8284	1381.6422	1381.6401	0.0020	0	K.TTPPVLDSDGSFF.L		(Ions score 67)
398 - 409	641.3041	1280.5936	1280.5925	0.0011	0	T.TPPVLDSDGSFF.L		(Ions score 41)
398 - 409	641.3042	1280.5938	1280.5925	0.0014	0	T.TPPVLDSDGSFF.L		(Ions score 26)
404 - 409	659.2677	658.2604	658.2598	0.0006	0	D.SDGSFF.L		(Ions score 21)
412 - 417	331.6904	661.3662	661.3646	0.0016	0	Y.SKLTVD.K		(Ions score 34)
412 - 423	492.6095	1474.8066	1474.7892	0.0174	0	Y.SKLTVDKSRWQQ.G		(Ions score 24)
412 - 423	492.6131	1474.8174	1474.7892	0.0282	0	Y.SKLTVDKSRWQQ.G		(Ions score 24)
412 - 425	549.6312	1645.8717	1645.8536	0.0181	0	Y.SKLTVDKSRWQQGN.V		(Ions score 31)
412 - 427	947.0016	1891.9886	1891.9904	-0.0018	0	Y.SKLTVDKSRWQQGNVF.S		(Ions score 50)
412 - 427	631.6711	1891.9914	1891.9904	0.0010	0	Y.SKLTVDKSRWQQGNVF.S		(Ions score 25)
412 - 427	947.0034	1891.9922	1891.9904	0.0018	0	Y.SKLTVDKSRWQQGNVF.S		(Ions score 49)
412 - 429	714.0251	2139.0534	2139.0531	0.0004	0	Y.SKLTVDKSRWQQGNVFSC.S		(Ions score 43)
412 - 429	714.0267	2139.0582	2139.0531	0.0051	0	Y.SKLTVDKSRWQQGNVFSC.S		(Ions score 39)
415 - 427	522.2678	1563.7816	1563.7793	0.0023	0	L.TVDKSRWQQGNVF.S		(Ions score 23)
415 - 427	782.8986	1563.7826	1563.7793	0.0032	0	L.TVDKSRWQQGNVF.S		(Ions score 32)
418 - 427	625.3267	1248.6388	1248.6363	0.0025	0	D.KSRWQQGNVF.S		(Ions score 33)
430 - 436	393.6942	785.3738	785.3741	-0.0003	0	C.SVMHEAL.H		(Ions score 29)
430 - 436	393.6942	785.3739	785.3741	-0.0003	0	C.SVMHEAL.H		(Ions score 26)
430 - 436	393.6944	785.3742	785.3741	0.0000	0	C.SVMHEAL.H		(Ions score 26)
430 - 436	401.6913	801.3680	801.3691	-0.0010	0	C.SVMHEAL.H	Oxidation (M)	(Ions score 32)
430 - 436	401.6960	801.3775	801.3691	0.0085	0	C.SVMHEAL.H	Oxidation (M)	(Ions score 28)
430 - 443	565.6074	1693.8003	1693.7994	0.0008	0	C.SVMHEALHNHYTQK.S		(Ions score 34)
430 - 450	779.3880	2335.1422	2335.1379	0.0043	0	C.SVMHEALHNHYTQKSLSLSPG.K		(Ions score 39)
433 - 443	459.8932	1376.6576	1376.6585	-0.0009	0	M.HEALHNHYTQK.S		(Ions score 22)
433 - 445	526.5985	1576.7737	1576.7746	-0.0009	0	M.HEALHNHYTQKSL.S		(Ions score 34)
433 - 445	526.5989	1576.7750	1576.7746	0.0004	0	M.HEALHNHYTQKSL.S		(Ions score 29)
433 - 450	673.6819	2018.0238	2017.9969	0.0269	0	M.HEALHNHYTQKSLSLSPG.K		(Ions score 25)
437 - 443	464.2251	926.4357	926.4359	-0.0001	0	L.HNHYTQK.S		(Ions score 22)
437 - 443	464.2255	926.4365	926.4359	0.0006	0	L.HNHYTQK.S		(Ions score 22)
444 - 451	394.7284	787.4423	787.4439	-0.0016	0	K.SLSLSPGK.-		(Ions score 32)
446 - 450	460.2446	459.2373	459.2329	0.0044	0	L.SLSPG.K		(Ions score 27)

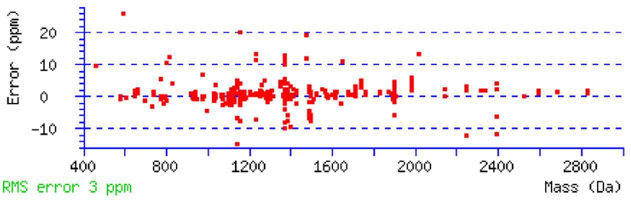

RMS error 3 ppm

Mascot: http://www.matrixscience.com/
