# Supplementary material for: An alternative for proteinase K-heat-sensitive protease from fungus Onygena corvina for biotechnology: cloning, engineering, expression, characterization and special application for protein sequencing
Source: Microb Cell Fact. 2020 Jun 24;19:135. doi: 10.1186/s12934-020-01392-3 (PMC7313183; doi:10.1186/s12934-020-01392-3)
Supplement: Supplementary file 18 — Additional file 18. MASCOT Search Results of NHSSP-cleaved monoclonal mAb light chain. [file 12934_2020_1392_MOESM18_ESM.pdf]

Mascot Search Results

Protein View

Match to: **mAb_LC** Score: **4682**

Found in search of \\SERVER3\User\Kunden_Projekte\Koller_8375\Further-characterization-part-I\8375_mAB_HL1_1070nl.mgf

Nominal mass (M_r): **23327**; Calculated pI value: **8.26**
NCBI BLAST search of mAb_LC against nr
Unformatted sequence string for pasting into other applications

Fixed modifications: Carbamidomethyl (C)
Variable modifications: Oxidation (M)
No enzyme cleavage specificity
Sequence Coverage: **96%**

Matched peptides shown in **Bold Red**

```
  1 QIVLSQSPAI LSASPGEKVT MTCRASSSVS YIHWFQQKPG SSPKPWIYAT
 51 SNLASGVPVR FSGSGSGTSY SLTISRVEAE DAATYYCQQW TSNPPTFGGG
101 TKLEIKRTVA APSVFIFPPS DEQLKSGTAS VVCLLNNFYP REAKVQWKVD
151 NALQSGNSQE SVTEQDSKDS TYSLSSTLTL SKADYEKHKV YACEVTHQGL
201 SSPVTKSFNR GEC
```

Sort Peptides By ◉ Residue Number ○ Increasing Mass ○ Decreasing Mass

Start - End	Observed	Mr(expt)	Mr(calc)	Delta	Miss	Sequence
5 - 11	715.3978	714.3906	714.3912	-0.0006	0	L.SQSPAIL.S (Ions score 22)
5 - 11	715.3981	714.3909	714.3912	-0.0003	0	L.SQSPAIL.S (Ions score 22)
5 - 11	715.3982	714.3909	714.3912	-0.0003	0	L.SQSPAIL.S (Ions score 28)
5 - 11	715.3988	714.3915	714.3912	0.0003	0	L.SQSPAIL.S (Ions score 21)
7 - 11	500.3072	499.2999	499.3006	-0.0007	0	Q.SPAIL.S (Ions score 26)
7 - 11	500.3076	499.3004	499.3006	-0.0002	0	Q.SPAIL.S (Ions score 29)
12 - 20	438.2267	874.4389	874.4396	-0.0007	0	L.SASPGEKVT.M (Ions score 40)
12 - 20	438.2270	874.4394	874.4396	-0.0001	0	L.SASPGEKVT.M (Ions score 47)
12 - 21	503.7473	1005.4801	1005.4801	0.0001	0	L.SASPGEKVTM.T (Ions score 56)
12 - 21	503.7478	1005.4811	1005.4801	0.0010	0	L.SASPGEKVTM.T (Ions score 54)
12 - 21	503.7530	1005.4914	1005.4801	0.0114	0	L.SASPGEKVTM.T (Ions score 61)
12 - 21	511.7443	1021.4740	1021.4750	-0.0009	0	L.SASPGEKVTM.T Oxidation (M) (Ions score 38)
12 - 21	511.7445	1021.4744	1021.4750	-0.0006	0	L.SASPGEKVTM.T Oxidation (M) (Ions score 48)
12 - 21	511.7445	1021.4745	1021.4750	-0.0005	0	L.SASPGEKVTM.T Oxidation (M) (Ions score 36)
12 - 21	511.7447	1021.4748	1021.4750	-0.0001	0	L.SASPGEKVTM.T Oxidation (M) (Ions score 51)
12 - 22	554.2710	1106.5274	1106.5277	-0.0003	0	L.SASPGEKVTMT.C (Ions score 54)
12 - 22	554.2710	1106.5274	1106.5277	-0.0003	0	L.SASPGEKVTMT.C (Ions score 62)
12 - 22	554.2720	1106.5294	1106.5277	0.0017	0	L.SASPGEKVTMT.C (Ions score 67)
12 - 22	554.2745	1106.5345	1106.5277	0.0068	0	L.SASPGEKVTMT.C (Ions score 58)
12 - 22	554.2776	1106.5406	1106.5277	0.0129	0	L.SASPGEKVTMT.C (Ions score 51)
12 - 22	554.2776	1106.5406	1106.5277	0.0129	0	L.SASPGEKVTMT.C (Ions score 52)
12 - 22	554.2781	1106.5416	1106.5277	0.0139	0	L.SASPGEKVTMT.C (Ions score 46)
12 - 22	562.2636	1122.5127	1122.5227	-0.0100	0	L.SASPGEKVTMT.C Oxidation (M) (Ions score 44)
12 - 22	562.2646	1122.5147	1122.5227	-0.0079	0	L.SASPGEKVTMT.C Oxidation (M) (Ions score 30)
12 - 22	562.2681	1122.5216	1122.5227	-0.0011	0	L.SASPGEKVTMT.C Oxidation (M) (Ions score 25)
12 - 22	562.2688	1122.5230	1122.5227	0.0004	0	L.SASPGEKVTMT.C Oxidation (M) (Ions score 64)
12 - 22	562.2690	1122.5235	1122.5227	0.0009	0	L.SASPGEKVTMT.C Oxidation (M) (Ions score 67)
12 - 22	562.2690	1122.5235	1122.5227	0.0009	0	L.SASPGEKVTMT.C Oxidation (M) (Ions score 46)
12 - 22	562.2692	1122.5238	1122.5227	0.0011	0	L.SASPGEKVTMT.C Oxidation (M) (Ions score 37)
12 - 22	562.2697	1122.5249	1122.5227	0.0022	0	L.SASPGEKVTMT.C Oxidation (M) (Ions score 37)
12 - 22	562.2747	1122.5348	1122.5227	0.0121	0	L.SASPGEKVTMT.C Oxidation (M) (Ions score 70)
12 - 22	562.2751	1122.5356	1122.5227	0.0130	0	L.SASPGEKVTMT.C Oxidation (M) (Ions score 52)
12 - 26	791.3683	1580.7220	1580.7286	-0.0066	0	L.SASPGEKVTMTCRAS.S (Ions score 39)
12 - 26	527.9177	1580.7313	1580.7286	0.0027	0	L.SASPGEKVTMTCRAS.S (Ions score 46)
27 - 33	396.6972	791.3799	791.3813	-0.0014	0	S.SSVSYIH.W (Ions score 47)
27 - 33	396.6979	791.3812	791.3813	-0.0001	0	S.SSVSYIH.W (Ions score 54)
31 - 35	383.1892	764.3639	764.3646	-0.0007	0	S.YIHWF.Q (Ions score 21)
31 - 35	383.1895	764.3645	764.3646	-0.0000	0	S.YIHWF.Q (Ions score 26)
31 - 35	383.1899	764.3652	764.3646	0.0006	0	S.YIHWF.Q (Ions score 27)
31 - 35	383.1900	764.3655	764.3646	0.0009	0	S.YIHWF.Q (Ions score 26)
31 - 36	447.2186	892.4226	892.4232	-0.0005	0	S.YIHWFQ.Q (Ions score 31)
31 - 36	447.2188	892.4229	892.4232	-0.0002	0	S.YIHWFQ.Q (Ions score 26)
31 - 36	447.2189	892.4233	892.4232	0.0001	0	S.YIHWFQ.Q (Ions score 28)
34 - 40	445.7297	889.4448	889.4446	0.0002	0	H.WFQQKPG.S (Ions score 26)
34 - 46	524.9373	1571.7901	1571.7885	0.0017	0	H.WFQQKPGSSPKPW.I (Ions score 23)
34 - 46	524.9377	1571.7912	1571.7885	0.0028	0	H.WFQQKPGSSPKPW.I (Ions score 21)
34 - 46	786.9030	1571.7915	1571.7885	0.0030	0	H.WFQQKPGSSPKPW.I (Ions score 40)
34 - 46	786.9036	1571.7926	1571.7885	0.0041	0	H.WFQQKPGSSPKPW.I (Ions score 46)
34 - 46	786.9039	1571.7932	1571.7885	0.0047	0	H.WFQQKPGSSPKPW.I (Ions score 60)
34 - 46	786.9040	1571.7934	1571.7885	0.0050	0	H.WFQQKPGSSPKPW.I (Ions score 53)
34 - 46	524.9385	1571.7936	1571.7885	0.0051	0	H.WFQQKPGSSPKPW.I (Ions score 32)
34 - 48	924.9767	1847.9388	1847.9359	0.0030	0	H.WFQQKPGSSPKPWIY.A (Ions score 27)
34 - 48	616.9871	1847.9395	1847.9359	0.0037	0	H.WFQQKPGSSPKPWIY.A (Ions score 30)
34 - 48	616.9879	1847.9419	1847.9359	0.0061	0	H.WFQQKPGSSPKPWIY.A (Ions score 23)
34 - 48	924.9785	1847.9425	1847.9359	0.0066	0	H.WFQQKPGSSPKPWIY.A (Ions score 21)
35 - 46	462.9090	1385.7053	1385.7092	-0.0039	0	W.FQQKPGSSPKPW.I (Ions score 22)
36 - 46	620.3335	1238.6524	1238.6407	0.0117	0	F.QQKPGSSPKPW.I (Ions score 20)
49 - 61	659.8599	1317.7052	1317.7040	0.0011	0	Y.ATSNLASGVPVRF.S (Ions score 95)
49 - 62	703.3755	1404.7364	1404.7361	0.0004	0	Y.ATSNLASGVPVRFS.G (Ions score 64)
49 - 62	703.3772	1404.7398	1404.7361	0.0038	0	Y.ATSNLASGVPVRFS.G (Ions score 65)
49 - 64	775.4026	1548.7906	1548.7896	0.0011	0	Y.ATSNLASGVPVRFSGS.G (Ions score 42)

49 - 64	775.4031	1548.7917	1548.7896	0.0022	0	Y.ATSNLASGVPVRFSGS.G	(Ions score 88)
49 - 70	1051.5138	2101.0130	2101.0075	0.0055	0	Y.ATSNLASGVPVRFSGSGSGTSY.S	(Ions score 58)
49 - 70	1051.5150	2101.0155	2101.0075	0.0080	0	Y.ATSNLASGVPVRFSGSGSGTSY.S	(Ions score 29)
49 - 72	1151.5658	2301.1170	2301.1236	-0.0066	0	Y.ATSNLASGVPVRFSGSGSGTSYSL.T	(Ions score 78)
49 - 72	1151.5706	2301.1266	2301.1236	0.0030	0	Y.ATSNLASGVPVRFSGSGSGTSYSL.T	(Ions score 33)
49 - 72	1151.5706	2301.1266	2301.1236	0.0030	0	Y.ATSNLASGVPVRFSGSGSGTSYSL.T	(Ions score 45)
49 - 72	1151.5712	2301.1278	2301.1236	0.0042	0	Y.ATSNLASGVPVRFSGSGSGTSYSL.T	(Ions score 66)
51 - 55	491.2456	490.2384	490.2387	-0.0003	0	T.SNLAS.G	(Ions score 32)
51 - 61	573.8172	1145.6198	1145.6192	0.0006	0	T.SNLASGVPVRF.S	(Ions score 62)
51 - 61	573.8188	1145.6230	1145.6192	0.0038	0	T.SNLASGVPVRF.S	(Ions score 63)
51 - 62	617.3318	1232.6490	1232.6513	-0.0023	0	T.SNLASGVPVRFS.G	(Ions score 60)
51 - 62	617.3334	1232.6522	1232.6513	0.0009	0	T.SNLASGVPVRFS.G	(Ions score 72)
51 - 62	617.3347	1232.6548	1232.6513	0.0035	0	T.SNLASGVPVRFS.G	(Ions score 71)
51 - 64	689.3556	1376.6966	1376.7048	-0.0081	0	T.SNLASGVPVRFSGS.G	(Ions score 69)
51 - 64	689.3560	1376.6974	1376.7048	-0.0074	0	T.SNLASGVPVRFSGS.G	(Ions score 74)
51 - 70	965.4713	1928.9281	1928.9227	0.0053	0	T.SNLASGVPVRFSGSGSGTSY.S	(Ions score 78)
51 - 70	965.4728	1928.9311	1928.9227	0.0084	0	T.SNLASGVPVRFSGSGSGTSY.S	(Ions score 58)
51 - 72	1065.5240	2129.0335	2129.0388	-0.0053	0	T.SNLASGVPVRFSGSGSGTSYSL.T	(Ions score 77)
51 - 72	1065.5254	2129.0362	2129.0388	-0.0026	0	T.SNLASGVPVRFSGSGSGTSYSL.T	(Ions score 100)
51 - 72	1065.5269	2129.0392	2129.0388	0.0003	0	T.SNLASGVPVRFSGSGSGTSYSL.T	(Ions score 48)
51 - 72	1065.5272	2129.0399	2129.0388	0.0011	0	T.SNLASGVPVRFSGSGSGTSYSL.T	(Ions score 66)
51 - 72	1065.5277	2129.0409	2129.0388	0.0021	0	T.SNLASGVPVRFSGSGSGTSYSL.T	(Ions score 67)
52 - 61	530.3010	1058.5874	1058.5872	0.0002	0	S.NLASGVPVRF.S	(Ions score 50)
52 - 72	1022.0081	2042.0017	2042.0068	-0.0051	0	S.NLASGVPVRFSGSGSGTSYSL.T	(Ions score 84)
54 - 61	416.7380	831.4615	831.4602	0.0013	0	L.ASGVPVRF.S	(Ions score 20)
54 - 62	460.2523	918.4901	918.4923	-0.0021	0	L.ASGVPVRFS.G	(Ions score 43)
54 - 62	460.2525	918.4904	918.4923	-0.0019	0	L.ASGVPVRFS.G	(Ions score 51)
54 - 62	460.2527	918.4909	918.4923	-0.0014	0	L.ASGVPVRFS.G	(Ions score 58)
54 - 70	808.3915	1614.7684	1614.7637	0.0047	0	L.ASGVPVRFSGSGSGTSY.S	(Ions score 74)
54 - 72	908.4500	1814.8855	1814.8798	0.0057	0	L.ASGVPVRFSGSGSGTSYSL.T	(Ions score 21)
55 - 62	424.7340	847.4534	847.4552	-0.0018	0	A.SGVPVRFS.G	(Ions score 26)
56 - 61	337.7033	673.3920	673.3911	0.0009	0	S.GVPVRF.S	(Ions score 32)
56 - 62	381.2183	760.4220	760.4231	-0.0011	0	S.GVPVRFS.G	(Ions score 27)
56 - 62	381.2185	760.4224	760.4231	-0.0007	0	S.GVPVRFS.G	(Ions score 29)
56 - 62	381.2193	760.4240	760.4231	0.0009	0	S.GVPVRFS.G	(Ions score 34)
56 - 64	453.2457	904.4768	904.4766	0.0002	0	S.GVPVRFSGS.G	(Ions score 29)
56 - 70	729.3566	1456.6986	1456.6946	0.0040	0	S.GVPVRFSGSGSGTSY.S	(Ions score 77)
56 - 70	729.3569	1456.6992	1456.6946	0.0046	0	S.GVPVRFSGSGSGTSY.S	(Ions score 58)
56 - 70	729.3571	1456.6997	1456.6946	0.0051	0	S.GVPVRFSGSGSGTSY.S	(Ions score 81)
56 - 72	829.4143	1656.8141	1656.8107	0.0034	0	S.GVPVRFSGSGSGTSYSL.T	(Ions score 69)
58 - 62	303.1731	604.3317	604.3333	-0.0016	0	V.PVRFS.G	(Ions score 24)
58 - 62	303.1733	604.3320	604.3333	-0.0013	0	V.PVRFS.G	(Ions score 23)
73 - 78	352.7003	703.3861	703.3864	-0.0003	0	L.TISRVE.A	(Ions score 26)
73 - 85	713.3457	1424.6769	1424.6783	-0.0014	0	L.TISRVEAEDAATY.Y	(Ions score 67)
73 - 85	713.3461	1424.6777	1424.6783	-0.0006	0	L.TISRVEAEDAATY.Y	(Ions score 45)
73 - 85	713.3470	1424.6794	1424.6783	0.0011	0	L.TISRVEAEDAATY.Y	(Ions score 55)
76 - 80	302.1578	602.3011	602.3024	-0.0012	0	S.RVEAE.D	(Ions score 23)
76 - 80	302.1579	602.3013	602.3024	-0.0010	0	S.RVEAE.D	(Ions score 29)
76 - 80	302.1580	602.3014	602.3024	-0.0010	0	S.RVEAE.D	(Ions score 25)
76 - 80	302.1581	602.3017	602.3024	-0.0006	0	S.RVEAE.D	(Ions score 26)
76 - 84	481.2323	960.4501	960.4512	-0.0011	0	S.RVEAEDAAT.Y	(Ions score 26)
76 - 85	562.7623	1123.5101	1123.5145	-0.0044	0	S.RVEAEDAATY.Y	(Ions score 36)
76 - 85	562.7631	1123.5117	1123.5145	-0.0028	0	S.RVEAEDAATY.Y	(Ions score 38)
76 - 85	562.7638	1123.5130	1123.5145	-0.0015	0	S.RVEAEDAATY.Y	(Ions score 33)
76 - 85	562.7639	1123.5133	1123.5145	-0.0012	0	S.RVEAEDAATY.Y	(Ions score 31)
76 - 85	562.7645	1123.5145	1123.5145	-0.0000	0	S.RVEAEDAATY.Y	(Ions score 34)
76 - 85	562.7648	1123.5150	1123.5145	0.0005	0	S.RVEAEDAATY.Y	(Ions score 53)
76 - 86	644.2966	1286.5786	1286.5778	0.0007	0	S.RVEAEDAATYY.C	(Ions score 33)
76 - 86	644.2976	1286.5807	1286.5778	0.0028	0	S.RVEAEDAATYY.C	(Ions score 47)
76 - 86	644.2979	1286.5813	1286.5778	0.0034	0	S.RVEAEDAATYY.C	(Ions score 44)
86 - 106	804.7280	2411.1621	2411.1579	0.0041	0	Y.YCQQWTSNPPTFGGGTKLEIK.R	(Ions score 57)
86 - 106	1206.5900	2411.1654	2411.1579	0.0075	0	Y.YCQQWTSNPPTFGGGTKLEIK.R	(Ions score 59)
86 - 106	804.7295	2411.1666	2411.1579	0.0087	0	Y.YCQQWTSNPPTFGGGTKLEIK.R	(Ions score 60)
87 - 106	750.3656	2248.0750	2248.0946	-0.0196	0	Y.CQQWTSNPPTFGGGTKLEIK.R	(Ions score 44)
87 - 106	750.3659	2248.0759	2248.0946	-0.0187	0	Y.CQQWTSNPPTFGGGTKLEIK.R	(Ions score 43)
89 - 103	795.8997	1589.7849	1589.7838	0.0011	0	Q.QWTSNPPTFGGGTKL.E	(Ions score 49)
89 - 103	795.9018	1589.7890	1589.7838	0.0053	0	Q.QWTSNPPTFGGGTKL.E	(Ions score 50)
89 - 103	795.9023	1589.7900	1589.7838	0.0062	0	Q.QWTSNPPTFGGGTKL.E	(Ions score 63)
89 - 106	654.3384	1959.9933	1960.0054	-0.0121	0	Q.QWTSNPPTFGGGTKLEIK.R	(Ions score 36)
89 - 106	981.0040	1959.9935	1960.0054	-0.0119	0	Q.QWTSNPPTFGGGTKLEIK.R	(Ions score 63)
89 - 106	654.3387	1959.9942	1960.0054	-0.0111	0	Q.QWTSNPPTFGGGTKLEIK.R	(Ions score 22)
89 - 106	654.3387	1959.9942	1960.0054	-0.0111	0	Q.QWTSNPPTFGGGTKLEIK.R	(Ions score 47)
89 - 106	981.0051	1959.9957	1960.0054	-0.0097	0	Q.QWTSNPPTFGGGTKLEIK.R	(Ions score 52)
89 - 106	981.0056	1959.9967	1960.0054	-0.0087	0	Q.QWTSNPPTFGGGTKLEIK.R	(Ions score 58)
89 - 106	654.3430	1960.0072	1960.0054	0.0019	0	Q.QWTSNPPTFGGGTKLEIK.R	(Ions score 39)
89 - 106	654.3432	1960.0078	1960.0054	0.0024	0	Q.QWTSNPPTFGGGTKLEIK.R	(Ions score 23)
91 - 103	638.8293	1275.6440	1275.6459	-0.0019	0	W.TSNPPTFGGGTKL.E	(Ions score 53)
91 - 103	638.8302	1275.6458	1275.6459	-0.0000	0	W.TSNPPTFGGGTKL.E	(Ions score 55)
91 - 106	823.9409	1645.8673	1645.8675	-0.0002	0	W.TSNPPTFGGGTKLEIK.R	(Ions score 47)
91 - 106	823.9421	1645.8696	1645.8675	0.0021	0	W.TSNPPTFGGGTKLEIK.R	(Ions score 24)
91 - 106	823.9421	1645.8696	1645.8675	0.0021	0	W.TSNPPTFGGGTKLEIK.R	(Ions score 58)
94 - 106	672.8788	1343.7431	1343.7448	-0.0017	0	N.PPTFGGGTKLEIK.R	(Ions score 61)
94 - 106	448.9219	1343.7439	1343.7448	-0.0010	0	N.PPTFGGGTKLEIK.R	(Ions score 46)
94 - 106	448.9221	1343.7445	1343.7448	-0.0003	0	N.PPTFGGGTKLEIK.R	(Ions score 32)
94 - 106	448.9224	1343.7453	1343.7448	0.0004	0	N.PPTFGGGTKLEIK.R	(Ions score 35)
107 - 113	351.2003	700.3861	700.3868	-0.0007	0	K.RTVAAPS.V	(Ions score 22)
107 - 113	351.2004	700.3863	700.3868	-0.0005	0	K.RTVAAPS.V	(Ions score 29)
107 - 115	474.2694	946.5243	946.5236	0.0007	0	K.RTVAAPSVF.I	(Ions score 46)
107 - 115	474.2699	946.5252	946.5236	0.0017	0	K.RTVAAPSVF.I	(Ions score 41)
107 - 115	474.2700	946.5254	946.5236	0.0018	0	K.RTVAAPSVF.I	(Ions score 50)
107 - 115	474.2706	946.5266	946.5236	0.0030	0	K.RTVAAPSVF.I	(Ions score 23)
107 - 115	474.2709	946.5273	946.5236	0.0037	0	K.RTVAAPSVF.I	(Ions score 37)

107 - 115	474.2713	946.5281	946.5236	0.0045	0	K.RTVAAPSVF.I	(Ions score 48)
107 - 115	474.2715	946.5284	946.5236	0.0048	0	K.RTVAAPSVF.I	(Ions score 42)
114 - 126	753.8961	1505.7777	1505.7766	0.0011	0	S.VFIFPPSDEQLKS.G	(Ions score 40)
114 - 126	753.8963	1505.7781	1505.7766	0.0015	0	S.VFIFPPSDEQLKS.G	(Ions score 39)
114 - 126	753.8964	1505.7782	1505.7766	0.0016	0	S.VFIFPPSDEQLKS.G	(Ions score 67)
114 - 126	753.8965	1505.7784	1505.7766	0.0019	0	S.VFIFPPSDEQLKS.G	(Ions score 43)
114 - 129	868.4493	1734.8841	1734.8828	0.0013	0	S.VFIFPPSDEQLKSGTA.S	(Ions score 66)
114 - 129	868.4496	1734.8847	1734.8828	0.0019	0	S.VFIFPPSDEQLKSGTA.S	(Ions score 50)
114 - 129	868.4499	1734.8852	1734.8828	0.0024	0	S.VFIFPPSDEQLKSGTA.S	(Ions score 53)
114 - 129	868.4500	1734.8854	1734.8828	0.0026	0	S.VFIFPPSDEQLKSGTA.S	(Ions score 53)
114 - 129	868.4501	1734.8856	1734.8828	0.0028	0	S.VFIFPPSDEQLKSGTA.S	(Ions score 61)
114 - 129	868.4501	1734.8857	1734.8828	0.0029	0	S.VFIFPPSDEQLKSGTA.S	(Ions score 41)
114 - 129	868.4502	1734.8858	1734.8828	0.0030	0	S.VFIFPPSDEQLKSGTA.S	(Ions score 74)
114 - 129	868.4503	1734.8860	1734.8828	0.0032	0	S.VFIFPPSDEQLKSGTA.S	(Ions score 48)
114 - 129	868.4504	1734.8863	1734.8828	0.0035	0	S.VFIFPPSDEQLKSGTA.S	(Ions score 76)
114 - 129	868.4504	1734.8863	1734.8828	0.0035	0	S.VFIFPPSDEQLKSGTA.S	(Ions score 57)
114 - 129	868.4505	1734.8864	1734.8828	0.0037	0	S.VFIFPPSDEQLKSGTA.S	(Ions score 33)
114 - 129	868.4506	1734.8867	1734.8828	0.0039	0	S.VFIFPPSDEQLKSGTA.S	(Ions score 37)
116 - 126	630.8268	1259.6391	1259.6397	-0.0006	0	F.IFPPSDEQLKS.G	(Ions score 27)
116 - 126	630.8279	1259.6413	1259.6397	0.0016	0	F.IFPPSDEQLKS.G	(Ions score 21)
116 - 126	630.8281	1259.6417	1259.6397	0.0020	0	F.IFPPSDEQLKS.G	(Ions score 36)
116 - 126	630.8283	1259.6421	1259.6397	0.0023	0	F.IFPPSDEQLKS.G	(Ions score 20)
116 - 126	630.8284	1259.6423	1259.6397	0.0026	0	F.IFPPSDEQLKS.G	(Ions score 48)
116 - 126	630.8284	1259.6423	1259.6397	0.0026	0	F.IFPPSDEQLKS.G	(Ions score 34)
116 - 126	630.8287	1259.6429	1259.6397	0.0032	0	F.IFPPSDEQLKS.G	(Ions score 30)
116 - 126	630.8289	1259.6432	1259.6397	0.0034	0	F.IFPPSDEQLKS.G	(Ions score 39)
116 - 126	630.8290	1259.6434	1259.6397	0.0037	0	F.IFPPSDEQLKS.G	(Ions score 24)
116 - 126	630.8292	1259.6439	1259.6397	0.0042	0	F.IFPPSDEQLKS.G	(Ions score 28)
116 - 126	630.8292	1259.6439	1259.6397	0.0042	0	F.IFPPSDEQLKS.G	(Ions score 46)
116 - 126	630.8293	1259.6440	1259.6397	0.0043	0	F.IFPPSDEQLKS.G	(Ions score 38)
116 - 126	630.8294	1259.6443	1259.6397	0.0045	0	F.IFPPSDEQLKS.G	(Ions score 47)
116 - 126	630.8294	1259.6443	1259.6397	0.0045	0	F.IFPPSDEQLKS.G	(Ions score 36)
116 - 126	630.8295	1259.6445	1259.6397	0.0048	0	F.IFPPSDEQLKS.G	(Ions score 25)
116 - 126	630.8305	1259.6465	1259.6397	0.0067	0	F.IFPPSDEQLKS.G	(Ions score 34)
116 - 129	745.3702	1488.7258	1488.7460	-0.0202	0	F.IFPPSDEQLKSGTA.S	(Ions score 48)
116 - 129	745.3752	1488.7358	1488.7460	-0.0102	0	F.IFPPSDEQLKSGTA.S	(Ions score 38)
116 - 129	745.3766	1488.7387	1488.7460	-0.0072	0	F.IFPPSDEQLKSGTA.S	(Ions score 40)
116 - 129	745.3800	1488.7455	1488.7460	-0.0005	0	F.IFPPSDEQLKSGTA.S	(Ions score 47)
116 - 129	745.3804	1488.7463	1488.7460	0.0003	0	F.IFPPSDEQLKSGTA.S	(Ions score 68)
116 - 129	745.3805	1488.7464	1488.7460	0.0004	0	F.IFPPSDEQLKSGTA.S	(Ions score 50)
116 - 129	745.3807	1488.7469	1488.7460	0.0009	0	F.IFPPSDEQLKSGTA.S	(Ions score 25)
116 - 129	745.3810	1488.7474	1488.7460	0.0014	0	F.IFPPSDEQLKSGTA.S	(Ions score 22)
116 - 129	745.3812	1488.7478	1488.7460	0.0018	0	F.IFPPSDEQLKSGTA.S	(Ions score 43)
116 - 129	745.3818	1488.7490	1488.7460	0.0030	0	F.IFPPSDEQLKSGTA.S	(Ions score 53)
116 - 129	745.3823	1488.7500	1488.7460	0.0040	0	F.IFPPSDEQLKSGTA.S	(Ions score 49)
116 - 129	745.3825	1488.7505	1488.7460	0.0045	0	F.IFPPSDEQLKSGTA.S	(Ions score 47)
116 - 129	745.3826	1488.7506	1488.7460	0.0046	0	F.IFPPSDEQLKSGTA.S	(Ions score 52)
116 - 129	745.3829	1488.7513	1488.7460	0.0053	0	F.IFPPSDEQLKSGTA.S	(Ions score 51)
116 - 129	745.3830	1488.7514	1488.7460	0.0055	0	F.IFPPSDEQLKSGTA.S	(Ions score 54)
116 - 129	745.3831	1488.7516	1488.7460	0.0056	0	F.IFPPSDEQLKSGTA.S	(Ions score 33)
116 - 129	745.3832	1488.7519	1488.7460	0.0059	0	F.IFPPSDEQLKSGTA.S	(Ions score 52)
116 - 129	745.3834	1488.7522	1488.7460	0.0062	0	F.IFPPSDEQLKSGTA.S	(Ions score 44)
116 - 129	745.3837	1488.7529	1488.7460	0.0069	0	F.IFPPSDEQLKSGTA.S	(Ions score 41)
116 - 129	745.3841	1488.7536	1488.7460	0.0076	0	F.IFPPSDEQLKSGTA.S	(Ions score 43)
116 - 129	745.3845	1488.7544	1488.7460	0.0084	0	F.IFPPSDEQLKSGTA.S	(Ions score 47)
116 - 129	745.3845	1488.7544	1488.7460	0.0084	0	F.IFPPSDEQLKSGTA.S	(Ions score 29)
116 - 129	745.3848	1488.7550	1488.7460	0.0090	0	F.IFPPSDEQLKSGTA.S	(Ions score 56)
116 - 132	887.9646	1773.9146	1773.9148	-0.0002	0	F.IFPPSDEQLKSGTASVV.C	(Ions score 53)
116 - 132	887.9648	1773.9151	1773.9148	0.0003	0	F.IFPPSDEQLKSGTASVV.C	(Ions score 48)
116 - 132	887.9649	1773.9153	1773.9148	0.0004	0	F.IFPPSDEQLKSGTASVV.C	(Ions score 27)
116 - 132	887.9655	1773.9164	1773.9148	0.0015	0	F.IFPPSDEQLKSGTASVV.C	(Ions score 42)
116 - 132	887.9656	1773.9167	1773.9148	0.0019	0	F.IFPPSDEQLKSGTASVV.C	(Ions score 42)
116 - 132	887.9657	1773.9168	1773.9148	0.0020	0	F.IFPPSDEQLKSGTASVV.C	(Ions score 36)
116 - 132	887.9658	1773.9170	1773.9148	0.0021	0	F.IFPPSDEQLKSGTASVV.C	(Ions score 34)
116 - 132	887.9662	1773.9178	1773.9148	0.0030	0	F.IFPPSDEQLKSGTASVV.C	(Ions score 33)
116 - 132	887.9664	1773.9182	1773.9148	0.0034	0	F.IFPPSDEQLKSGTASVV.C	(Ions score 30)
116 - 132	887.9672	1773.9198	1773.9148	0.0049	0	F.IFPPSDEQLKSGTASVV.C	(Ions score 31)
116 - 132	887.9680	1773.9215	1773.9148	0.0067	0	F.IFPPSDEQLKSGTASVV.C	(Ions score 38)
116 - 132	887.9684	1773.9223	1773.9148	0.0075	0	F.IFPPSDEQLKSGTASVV.C	(Ions score 22)
133 - 137	633.3044	632.2972	632.2952	0.0020	0	V.CLLNN.F	(Ions score 26)
133 - 141	598.7975	1195.5805	1195.5808	-0.0002	0	V.CLLNNFYPR.E	(Ions score 46)
133 - 141	598.7978	1195.5810	1195.5808	0.0003	0	V.CLLNNFYPR.E	(Ions score 41)
134 - 141	518.7793	1035.5440	1035.5501	-0.0061	0	C.LLNNFYPR.E	(Ions score 62)
134 - 141	518.7826	1035.5506	1035.5501	0.0005	0	C.LLNNFYPR.E	(Ions score 51)
134 - 141	518.7830	1035.5514	1035.5501	0.0012	0	C.LLNNFYPR.E	(Ions score 57)
135 - 141	462.2394	922.4643	922.4661	-0.0018	0	L.LNNFYPR.E	(Ions score 46)
135 - 141	462.2397	922.4649	922.4661	-0.0011	0	L.LNNFYPR.E	(Ions score 39)
135 - 141	462.2402	922.4659	922.4661	-0.0002	0	L.LNNFYPR.E	(Ions score 45)
136 - 141	405.6971	809.3797	809.3820	-0.0023	0	L.NNFYPR.E	(Ions score 25)
136 - 141	405.6976	809.3806	809.3820	-0.0014	0	L.NNFYPR.E	(Ions score 27)
136 - 141	405.6976	809.3806	809.3820	-0.0014	0	L.NNFYPR.E	(Ions score 27)
136 - 141	405.6977	809.3809	809.3820	-0.0011	0	L.NNFYPR.E	(Ions score 27)
136 - 141	405.6978	809.3810	809.3820	-0.0010	0	L.NNFYPR.E	(Ions score 29)
136 - 141	405.6979	809.3812	809.3820	-0.0008	0	L.NNFYPR.E	(Ions score 22)
136 - 141	405.6983	809.3820	809.3820	-0.0000	0	L.NNFYPR.E	(Ions score 30)
136 - 141	405.6983	809.3821	809.3820	0.0001	0	L.NNFYPR.E	(Ions score 31)
136 - 141	405.6985	809.3824	809.3820	0.0004	0	L.NNFYPR.E	(Ions score 25)
136 - 146	683.3500	1364.6854	1364.6836	0.0018	0	L.NNFYPREAKVQ.W	(Ions score 39)
136 - 146	683.3506	1364.6866	1364.6836	0.0030	0	L.NNFYPREAKVQ.W	(Ions score 40)
136 - 146	455.9030	1364.6871	1364.6836	0.0035	0	L.NNFYPREAKVQ.W	(Ions score 31)
137 - 141	348.6732	695.3318	695.3391	-0.0073	0	N.NFYPR.E	(Ions score 27)
137 - 141	348.6739	695.3332	695.3391	-0.0059	0	N.NFYPR.E	(Ions score 25)

137 - 141	348.6754	695.3363	695.3391	-0.0027	0	N.NFYPR.E	(Ions score 27)
137 - 141	348.6760	695.3374	695.3391	-0.0016	0	N.NFYPR.E	(Ions score 28)
137 - 141	348.6761	695.3376	695.3391	-0.0015	0	N.NFYPR.E	(Ions score 30)
137 - 141	348.6762	695.3378	695.3391	-0.0013	0	N.NFYPR.E	(Ions score 20)
137 - 141	348.6765	695.3384	695.3391	-0.0007	0	N.NFYPR.E	(Ions score 30)
138 - 146	569.3127	1136.6108	1136.5978	0.0130	0	N.FYPREAKVQ.W	(Ions score 29)
147 - 153	423.2233	844.4320	844.4443	-0.0122	0	Q.WKVDNAL.Q	(Ions score 35)
147 - 153	423.2257	844.4369	844.4443	-0.0073	0	Q.WKVDNAL.Q	(Ions score 41)
147 - 153	423.2263	844.4380	844.4443	-0.0063	0	Q.WKVDNAL.Q	(Ions score 37)
147 - 153	423.2298	844.4450	844.4443	0.0008	0	Q.WKVDNAL.Q	(Ions score 47)
147 - 154	487.2601	972.5057	972.5029	0.0029	0	Q.WKVDNALQ.S	(Ions score 44)
147 - 154	487.2609	972.5073	972.5029	0.0044	0	Q.WKVDNALQ.S	(Ions score 56)
147 - 156	559.2878	1116.5611	1116.5563	0.0048	0	Q.WKVDNALQSG.N	(Ions score 39)
147 - 157	616.3092	1230.6039	1230.5993	0.0046	0	Q.WKVDNALQSGN.S	(Ions score 79)
147 - 168	817.3867	2449.1381	2449.1357	0.0025	0	Q.WKVDNALQSGNSQESVTEQDSK.D	(Ions score 95)
147 - 172	972.7779	2915.3119	2915.3056	0.0062	0	Q.WKVDNALQSGNSQESVTEQDSKDSTY.S	(Ions score 59)
147 - 172	972.7805	2915.3197	2915.3056	0.0141	0	Q.WKVDNALQSGNSQESVTEQDSKDSTY.S	(Ions score 72)
147 - 172	972.7806	2915.3199	2915.3056	0.0143	0	Q.WKVDNALQSGNSQESVTEQDSKDSTY.S	(Ions score 82)
147 - 175	1068.4891	3202.4456	3202.4538	-0.0082	0	Q.WKVDNALQSGNSQESVTEQDSKDSTYSLS.S	(Ions score 77)
154 - 168	812.3589	1622.7032	1622.7019	0.0013	0	L.QSGNSQESVTEQDSK.D	(Ions score 49)
154 - 172	1045.4436	2088.8727	2088.8719	0.0007	0	L.QSGNSQESVTEQDSKDSTY.S	(Ions score 70)
154 - 172	1045.4459	2088.8773	2088.8719	0.0054	0	L.QSGNSQESVTEQDSKDSTY.S	(Ions score 124)
154 - 172	1045.4460	2088.8775	2088.8719	0.0056	0	L.QSGNSQESVTEQDSKDSTY.S	(Ions score 104)
155 - 172	981.4172	1960.8198	1960.8134	0.0064	0	Q.SGNSQESVTEQDSKDSTY.S	(Ions score 91)
157 - 172	909.3902	1816.7658	1816.7599	0.0060	0	G.NSQESVTEQDSKDSTY.S	(Ions score 83)
158 - 172	852.3672	1702.7199	1702.7169	0.0030	0	N.SQESVTEQDSKDSTY.S	(Ions score 95)
158 - 172	852.3680	1702.7214	1702.7169	0.0045	0	N.SQESVTEQDSKDSTY.S	(Ions score 91)
160 - 172	744.8219	1487.6292	1487.6263	0.0029	0	Q.ESVTEQDSKDSTY.S	(Ions score 38)
166 - 172	408.1746	814.3346	814.3345	0.0002	0	Q.DSKDSTY.S	(Ions score 21)
179 - 191	527.9471	1580.8194	1580.8198	-0.0004	0	L.TLSKADYEKHKVY.A	(Ions score 22)
179 - 191	527.9479	1580.8218	1580.8198	0.0020	0	L.TLSKADYEKHKVY.A	(Ions score 33)
180 - 191	494.2646	1479.7718	1479.7721	-0.0003	0	T.LSKADYEKHKVY.A	(Ions score 34)
181 - 191	456.5698	1366.6876	1366.6881	-0.0005	0	L.SKADYEKHKVY.A	(Ions score 33)
181 - 191	456.5699	1366.6879	1366.6881	-0.0001	0	L.SKADYEKHKVY.A	(Ions score 34)
182 - 191	427.5596	1279.6569	1279.6560	0.0008	0	S.KADYEKHKVY.A	(Ions score 21)
184 - 191	541.2688	1080.5230	1080.5240	-0.0009	0	A.DYEKHKVY.A	(Ions score 24)
192 - 196	579.2446	578.2374	578.2370	0.0004	0	Y.ACEVT.H	(Ions score 32)
192 - 198	422.6842	843.3538	843.3545	-0.0007	0	Y.ACEVTHQ.G	(Ions score 56)
192 - 198	422.6850	843.3554	843.3545	0.0009	0	Y.ACEVTHQ.G	(Ions score 52)
194 - 198	307.1500	612.2854	612.2867	-0.0014	0	C.EVTHQ.G	(Ions score 21)
194 - 198	307.1501	612.2856	612.2867	-0.0011	0	C.EVTHQ.G	(Ions score 20)
194 - 198	307.1501	612.2857	612.2867	-0.0010	0	C.EVTHQ.G	(Ions score 23)
197 - 206	527.2875	1052.5604	1052.5614	-0.0010	0	T.HQGLSSPVTK.S	(Ions score 21)
197 - 206	527.2880	1052.5614	1052.5614	-0.0001	0	T.HQGLSSPVTK.S	(Ions score 35)
197 - 208	644.3384	1286.6623	1286.6619	0.0005	0	T.HQGLSSPVTKSF.N	(Ions score 57)
199 - 206	394.7290	787.4435	787.4439	-0.0005	0	Q.GLSSPVTK.S	(Ions score 33)
199 - 206	394.7304	787.4463	787.4439	0.0023	0	Q.GLSSPVTK.S	(Ions score 41)
199 - 206	394.7332	787.4519	787.4439	0.0079	0	Q.GLSSPVTK.S	(Ions score 38)
199 - 206	394.7336	787.4525	787.4439	0.0086	0	Q.GLSSPVTK.S	(Ions score 38)
199 - 206	394.7340	787.4535	787.4439	0.0095	0	Q.GLSSPVTK.S	(Ions score 37)
199 - 206	394.7340	787.4535	787.4439	0.0096	0	Q.GLSSPVTK.S	(Ions score 35)
199 - 206	394.7342	787.4538	787.4439	0.0098	0	Q.GLSSPVTK.S	(Ions score 39)
199 - 208	511.7788	1021.5431	1021.5444	-0.0012	0	Q.GLSSPVTKSF.N	(Ions score 41)
199 - 208	511.7795	1021.5445	1021.5444	0.0001	0	Q.GLSSPVTKSF.N	(Ions score 58)
199 - 208	511.7803	1021.5461	1021.5444	0.0017	0	Q.GLSSPVTKSF.N	(Ions score 63)
199 - 213	546.9342	1637.7808	1637.7831	-0.0023	0	Q.GLSSPVTKSFNRGEC.-	(Ions score 39)
199 - 213	546.9347	1637.7822	1637.7831	-0.0008	0	Q.GLSSPVTKSFNRGEC.-	(Ions score 37)
199 - 213	819.8990	1637.7834	1637.7831	0.0003	0	Q.GLSSPVTKSFNRGEC.-	(Ions score 65)
201 - 206	309.6762	617.3378	617.3384	-0.0006	0	L.SSPVTK.S	(Ions score 38)
207 - 213	435.1819	868.3492	868.3497	-0.0005	0	K.SFNRGEC.-	(Ions score 42)
207 - 213	435.1823	868.3501	868.3497	0.0004	0	K.SFNRGEC.-	(Ions score 45)

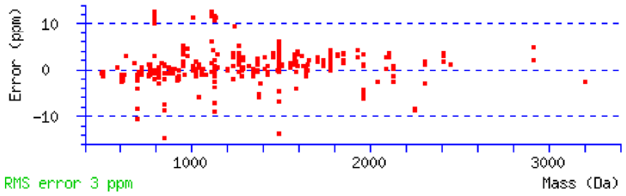

Mascot: http://www.matrixscience.com/
